# Concentration-Dependent Activity of Hydromethylthionine on Clinical Decline and Brain Atrophy in a Randomized Controlled Trial in Behavioral Variant Frontotemporal Dementia

**DOI:** 10.3233/JAD-191173

**Published:** 2020-05-19

**Authors:** Helen Shiells, Bjoern O. Schelter, Peter Bentham, Thomas C. Baddeley, Christopher M. Rubino, Harish Ganesan, Jeffrey Hammel, Vesna Vuksanovic, Roger T. Staff, Alison D. Murray, Luc Bracoud, Damon J. Wischik, Gernot Riedel, Serge Gauthier, Jianping Jia, Hans J. Moebius, Jiri Hardlund, Christopher M. Kipps, Karin Kook, John M.D. Storey, Charles R. Harrington, Claude M. Wischik

**Affiliations:** a TauRx Therapeutics Ltd., Aberdeen, UK; b Institute for Complex Systems and Mathematical Biology, University of Aberdeen, Aberdeen, UK; c Department of Chemistry, University of Aberdeen, Aberdeen, UK; d Institute of Clinical Pharmacodynamics, Schenectady, NY, USA; e Aberdeen Biomedical Imaging Centre, School of Medicine, Medical Sciences and Nutrition, University of Aberdeen, Aberdeen, UK; f Aberdeen Royal Infirmary, NHS Grampian, Aberdeen, UK; g Bioclinica, Lyon, France; h Department of Computer Science and Technology, University of Cambridge, Cambridge, UK; i School of Medicine, Medical Sciences and Nutrition, University of Aberdeen, Foresterhill, Aberdeen, UK; j McGill Centre for Studies in Aging, Alzheimer’s Disease Research Unit, and Douglas Mental Health University Institute, Montreal, QC, Canada; k Beijing Institute for Brain Disorders Alzheimer’s Disease Centre, Beijing, China; l Moebius-Consult, Baar, Switzerland; m University Hospital Southampton and University of Southampton, UK; n Salamandra LLC, Bethesda, MD, USA

**Keywords:** Behavioral variant frontotemporal dementia, clinical trials, hydromethylthionine, leucomethylthioninium, tau protein, TDP-43

## Abstract

**Background:**

Hydromethylthionine is a potent inhibitor of pathological aggregation of tau and TDP-43 proteins.

**Objective:**

To compare hydromethylthionine treatment effects at two doses and to determine how drug exposure is related to treatment response in bvFTD.

**Methods:**

We undertook a 52-week Phase III study in 220 bvFTD patients randomized to compare hydromethylthionine at 200 mg/day and 8 mg/day (intended as a control). The principal outcomes were change on the Addenbrookes Cognitive Examination – Revised (ACE-R), the Functional Activities Questionnaire (FAQ), and whole brain volume. Secondary outcomes included Modified Clinical Global Impression of Change (Modified-CGIC). A population pharmacokinetic exposure-response analysis was undertaken in 175 of the patients with available blood samples and outcome data using a discriminatory plasma assay for the parent drug.

**Results:**

There were no significant differences between the two doses as randomized. There were steep concentration-response relationships for plasma levels in the range 0.3–0.6 ng/ml at the 8 mg/day dose on clinical and MRI outcomes. There were significant exposure-dependent differences at 8 mg/day for FAQ, Modified-CGIC, and whole brain atrophy comparing patients with plasma levels greater than 0.346 ng/ml with having minimal drug exposure. The exposure-response is biphasic with worse outcomes at the high concentrations produced by 200 mg/day.

**Conclusions:**

Hydromethylthionine has a similar concentration-response profile for effects on clinical decline and brain atrophy at the 8 mg/day dose in bvFTD as recently reported in AD. Treatment responses in bvFTD are predicted to be maximal at doses in the range 20–60 mg/day. A confirmatory placebo-controlled trial is now planned.

## INTRODUCTION

Behavioral variant frontotemporal dementia (bvFTD) is a clinical syndrome characterized by progressive deterioration of personality, social comportment, and cognition [1]. Although a rare disorder, bvFTD is the second most common cause of dementia under age 65; there are also a significant number of cases in older people [2, 3]. The diagnosis of bvFTD is primarily clinical, with insidious onset and progressive deterioration, the core symptoms being disinhibition, apathy, lack of empathy, compulsions, hyperorality, and impairment of executive function. There are also patients who exhibit similar symptoms, but who do not suffer from a neurodegenerative condition [4, 5]. This phenocopy is characterized by preservation of functional ability [6], absence of a genetic abnormality [3], and normal imaging [5]. Patients with bvFTD decline significantly more rapidly on cognitive and functional measures than patients with AD [7], with mean survival from symptom onset approximately 8 years [8]. For a given level of severity, prevalence of significant burden in carers is much higher in bvFTD compared with AD [9], and patients with neuropathologically confirmed bvFTD have a significantly higher prevalence of criminal and socially inappropriate behavior compared with AD [10]. Because the typical age of onset is earlier than for AD [11, 12], the condition has a direct impact on working life and on household income. At 2016 US costs, bvFTD was found to reduce income from $75,000–$99,000 before diagnosis to $50,000 [13].

There are no treatments licensed for any form of FTD. There is no reliable evidence of benefit for acetylcholinesterase inhibitors [14, 15] and some suggestion that they may make FTD symptoms worse [16, 17]. Memantine is better tolerated but is also ineffective [18, 19]. There have been numerous small studies of other drugs targeting the behavioral symptoms, with some evidence of a small beneficial effect of trazadone on Neuropsychiatric Inventory (NPI) score [20]. However, the NPI is unsuitable as an outcome measure because the increasing passivity that accompanies disease progression is scored as a better outcome [21].

bvFTD and AD are both members of a class of progressive neurodegenerative disorders characterized by pathological aggregation and prion-like spread of otherwise normal proteins [22]. The largest consecutive bvFTD autopsy study published to date showed there is high confidence of a clinical diagnosis of bvFTD; characteristic pathology is present in 94% of cases, with 84% having pathological aggregation of tau protein (27%) or transactive response DNA binding protein 43 (TDP-43; 57%) [23]. There is increasing recognition of the importance of tau aggregation pathology as a substrate of clinical dementia and as a target for therapy in AD [24], and by implication in prion-like neurodegenerative disorders involving other proteins such as TDP-43.

The most advanced late-stage program targeting pathological protein aggregation currently in development is based on leuco-methylthioninium bis(hydromethanesulphonate) (LMTM) [25]. LMTM has recently been assigned the International Nonproprietary Name (INN) ‘hydromethylthionine’, recognizing it as chemically and pharmacologically distinct from methylthioninium chloride (MTC, methylene blue). The methylthioninium (MT) moiety can exist in oxidized (MT^+^) and reduced (LMT) forms. LMTM is a stabilized salt of LMT which has better brain uptake and tolerability than the oxidized MT^+^ form [26]. We have retained the LMTM abbreviation in contexts that require technical discussion of the distinctive properties of LMT, but otherwise we now use the INN more generally. We have reported recently that LMT rather than MT^+^ is the active species blocking tau aggregation *in vitro* and that it acts at a tau:LMT molar ratio of 1 : 0.1 [27]. Its site of action is within the proteolytically stable core tau unit of the tau aggregates found in both bvFTD and AD [28–30]. LMT blocks aggregation of the core tau unit in cell-based assays [25] and reduces tau aggregation pathology and associated behavioral deficits in a tau transgenic mouse model of bvFTD at clinically relevant doses [31]. There was increased clearance of pathological tau via enhancement of autophagy at the 10–20 nM concentration range in a mouse model of bvFTD [32] and reversal of resistance of filamentous tau to proteases [25, 33]. The MT moiety inhibits aggregation of TDP-43 in cell models with an EC_50_ of 0.05*μ*M [34], although not in a TDP-43 mouse model at a dose unlikely to have been sufficient for MTC activity [35].

The MT moiety has a range of other properties that affect cellular metabolism. It has been known for some time that it enhances mitochondrial activity at low concentrations (10–100 nM) [36, 37] by acting as a supplementary electron carrier in the electron transfer chain. This has been confirmed recently in an AD-like tau transgenic mouse model in which hydromethylthionine was found to increase Complex IV activity in the brain at clinically relevant doses [38]. It is able to induce mitochondrial biogenesis and to activate Nrf2-mediated oxidative stress response elements *in vivo* [39]. Other potentially beneficial activities include neuroprotective effects in the brain by inhibiting microglial activation and increasing autophagy [40]. Therefore, in addition to its actions on tau and TDP-43 aggregation, the MT moiety has complementary actions which address many of the pathways currently advocated as having potential for the treatment of neurodegenerative diseases [41–43].

We have previously reported the results of two Phase III trials using hydromethylthionine in AD [44, 45]. Both trials were designed as dose comparison studies, comparing doses in the range 150–250 mg/day with a low dose (8 mg/day) that was intended as a control to mask the urinary discoloration that occurs variably when urine from patients taking any form of MT is exposed to air [46]. The expectation was that this low dose would have no effect on brain structure or function, an expectation based on the results of an earlier placebo-controlled dose-finding Phase II trial using MTC which identified 138 mg/day as the minimum effective dose in AD [47], and early comparative Phase I pharmacokinetic studies showing similar plasma levels of total MT measured after acid extraction of samples [48]. However, we have found that this assay is dominated by an acid-labile inactive conjugate in plasma which is not distinguished from the active parent form of the drug following acid extraction.

We have developed a sensitive assay which can measure parent drug levels in plasma and which has been found to be reliable and accurate in preclinical and Phase I studies. Using this assay in a population pharmacokinetic (PK) study in 1,162 patients participating in the AD trials, we recently reported [49] that there is a steep concentration-response relationship on all clinical and brain magnetic resonance imaging (MRI) outcomes in patients receiving the 8 mg/day dose. Hydromethylthionine therefore has pharmacological activity on brain structure and function in the majority of AD patients at this dose. We also found that there is a predicted plateau in response at theoretical doses above 16 mg/day, consistent with the lack of dose-response at much higher doses in AD [49].

The design of the present Phase III study in bvFTD (TRx-237-007) was based on the same underlying premise as the AD trials, comparing a high dose of hydromethylthionine (200 mg/day given in divided doses twice daily) with a low dose (8 mg/day given in divided doses twice daily). We now report that, as in AD, there was no overall difference on any endpoint between these two doses in bvFTD. We also report the results of the embedded population PK analysis of clinical and MRI biomarker outcomes similar to that recently reported in AD [49], to determine how drug exposure is related to treatment response in bvFTD.

## MATERIALS AND METHODS

### Study design and participants, randomization and masking, and outcomes

The study was designed as a 52-week Phase III, randomized, controlled, double-blind, parallel-group trial conducted between May 2013 and February 2016 at 70 sites in Canada, United States, Australia, Asia, and Europe. Eligible patients had to be younger than 80 years of age with a diagnosis of bvFTD according to criteria revised by the International bvFTD Criteria Consortium [1, 50], with Mini-Mental Status Examination (MMSE) score greater than or equal to 20 at screening. In order to limit inclusion of bvFTD phenocopy cases, there was an additional requirement that patients had to meet the criterion of having definite brain atrophy in frontal and/or temporal lobes scoring 2 or more on a scale previously developed by Kipps et al. [51]. Concomitant use of acetylcholinesterase inhibitors (AChEIs) or memantine (or both) was permitted provided this was at a stable dose for at least 18 weeks before randomization to minimize any potential early symptomatic effects of these treatments. Concomitant use of antidepressant, antipsychotic (except clozapine or olanzapine), and sedative medications was also permitted at stable doses where clinically feasible. Each patient had one or more study partners participate with them in the trial as informants. Patients were excluded from the study if they had a significant CNS disorder other than bvFTD. A detailed list of inclusion and exclusion criteria is in the protocol provided in the Supplementary Material.

Patients were randomly assigned to receive hydromethylthionine 100 mg twice a day or hydromethylthionine 4 mg twice a day (*n* = 220). The randomization was stratified according to geographical region (three levels: North America, Europe, Asia/Australia). The randomization file and investigational medicinal product kit list were unavailable to personnel involved in conducting the study prior to final analysis after database lock. Study participants, their informant(s), and all assessors remained masked to treatment assignment throughout the study, and safety assessors were not permitted to be involved in efficacy assessments.

The two doses were provided in identical blister packages as visually identical oral tablets to be taken for up to 52 weeks. The primary outcome was the Addenbrookes Cognitive Examination – Revised (ACE-R) [52]. The Functional Activities Questionnaire (FAQ) [53] and reduction in progression of whole brain atrophy were alternative co-primary outcomes. Other outcomes included: Modified Alzheimer’s Disease Cooperative Study – Clinical Global Impression of Change (Modified-CGIC) [21] determined by a third independent rater; Frontotemporal Dementia Rating Scale (FRS) [54]; MMSE (from MMSE items incorporated into ACE-R); Addenbrookes Cognitive Examination – III (a revised version of ACE-R which excludes MMSE items subject to copyright restrictions [55]); change in brain volume measured by MRI in frontal and temporal lobes (FTV) and lateral ventricles (LVV); and the effect of hydromethylthionine in patients with known genetic mutations associated with bvFTD. Blood was collected prospectively for the purpose of population PK analyses.

MRI scans were obtained at screening and weeks 16, 32, and 52. The acquisition protocol was standardized across sites and all data were centrally collected, quality-controlled, and analyzed by the imaging core laboratory (Bioclinica). MRI data included a 3D sagittal T1-weighted sequence (using parameters compatible with the ADNI protocol) which was used for all volumetric analyses. For data passing quality-control, baseline volume was automatically assessed using FreeSurfer v5.3, while volume changes were assessed using Boundary Shift Integral (whole brain, lateral ventricles) [56] and Tensor-Based Morphometry (frontal and temporal lobes) [57].

Patients were monitored throughout the study for adverse events, including use of clinical laboratory tests (including measurement of methaemoglobin by pulse CO-oximetry), physical and neurological examinations, and 12-lead electrocardiograms (ECG) at all clinic visits (screening, baseline, and weeks 2, 16, 32, 52, and 56). The Unified Parkinson’s Disease Rating Scale Part III (UPDRS) [58] was included as a motor safety outcome measure requested by a regulatory agency, as was the Columbia-Suicide Severity Rating Scale (C-SSRS) [59], which were assessed at all visits. Patients were also systematically monitored for potential serotonin syndrome using a rating scale derived from four published diagnostic criteria [60], because of a theoretical potential for serotonin syndrome [61]. By protocol, amyloid related imaging abnormalities (ARIA), serotonin toxicity, and suicidality were to be reported as serious adverse events for expedited reporting.

### Statistical analysis

The primary analyses were conducted in the Modified Intent-to-Treat (MITT) population (all randomized patients who took at least one dose of study drug and had both a Baseline and at least one post-Baseline efficacy assessment; *n* = 214). The MRI imaging population comprised all MITT population patients with a Screening/Baseline and at least one valid post-baseline volumetric assessment (*n* = 209).

The primary analyses were specified as a mixed model, repeated-measures analysis with an unstructured covariance matrix and no imputation for missing data. The model included visit (three levels corresponding to assessments at weeks 16, 32, and 52), treatment group (two levels, 4 mg or 100 mg twice a day), a visit by treatment group interaction term, use of AD-labelled medications (two levels, using or not using), geographic region (three levels: North America, Asia/Australia, Europe), and baseline value of the variable analyzed. The individual comparisons were implemented through contrasts. The Bonferroni-Holm correction was used to take account of multiplicity arising from alternative co-primary outcomes (FAQ and whole brain atrophy). We used the same method for all secondary analyses in predefined gated sequences such that no further adjustment of alpha (0.05) was needed.

A sensitivity analysis was conducted to compare baseline MRI scans with those available from the parallel study in mild AD (TRx-237-005) [44] using the Statistical Parametric Mapping (SPM12) software package (http://www.fil.ion.ucl.ac.uk/spm/)) in analyses controlled for age, sex, and total intracranial volume.

Safety analyses were based on the Safety Population comprising 218 patients who received at least one dose of study drug, with summaries presented according to dose.

Data analyses specified in the Statistical Analysis Plan were undertaken independently of the funder by SynteractHCR (Carlsbad, CA, USA) using SAS 9.4 (Enterprise Guide v7.1). The results were verified and additional exploratory analyses were provided by two of the co-authors (HS, BOS) using R version 3.3.0 (2016-05-03). Additional voxel-based morphometric analyses (VBM) were provided by VV, TA, and RTS using the SPM12 software package. This trial is registered at http://www.clinicaltrials.gov (NCT01626378) and the European Union Clinical Trials Registry (EudraCT 2011-005529-34).

### Population PK analysis

Blood samples for assessment of parent MT, *N*-desmethyl MT, and total MT (sum of parent MT and a labile LMT conjugate), were collected from each patient on the first treatment visit (two samples: pre-dose and approximately 3.5 h after the dose) and at each subsequent on-treatment visit. The protocol specified that PK plasma sampling was to be conducted only at sites with adequate facilities (i.e., a refrigerated centrifuge and adequate capability to freeze samples reliably). Blinded analyses were conducted at the University of Aberdeen GLP Test Facility. MT levels in plasma were measured using liquid chromatography-tandem mass spectrometry assay. The method was validated for use in the Phase III studies over the range 0.2 to 10 ng/ml. Extrapolated MT concentrations were available below the lower limit of quantitation (but above the lower limit of detection) in approximately 35% of the Day 1 patients randomized to the 8 mg/day dose. Model development and estimation of steady state maximum plasma concentration (C_max,ss_) were determined independently by the Institute of Clinical Pharmacodynamics (ICPD, NY, USA). C_max,ss_ estimates using a model validated in Phase I studies were based on in-clinic plasma concentration data obtained on Day 1 when the dose-sampling interval was accurately recorded. The percentage of patients with Day 1 exposure levels below the lower calibration limit of the assay was used to define a proxy for placebo in *post-hoc* binary statistical comparisons of patients with low and high exposure to the drug with a view to informing the design of a future placebo-controlled trial. Change in ACE-R, ACE-III, FAQ, MMSE, Modified-CGIC, FRS, whole brain volume (WBV), lateral ventricular volume (LVV), and frontotemporal volume (FTV), as well as UPDRS Part III, were expressed as a function of C_max,ss_ estimates grouped into four groups described in the results section. C_max,ss_ values outside 3x the interquartile range were excluded from determination of concentration-response relationships, but were included in inferential statistical analyses.

The concentration-response analyses used a Mixed-effect Model Repeat Measurement (MMRM) with per-subject correction and an unstructured covariance matrix according to the following formula:

*Treatment effect*∼*plasma-level x visit* + *plasma-level x co-medication-status* + *co-medication-status x visit* + *MMSE-class x visit* + *sex x visit* + *age-class x visit* + *geographical-region* + *baseline-score*

The following terms were categorical variables in the models used for concentration-response analyses: *plasma level* (five levels), *visit* (three levels), *co-medication status* with AD drugs (two levels), *geographical-region* (three levels), *MMSE-class* (two levels, ≤20 and >21), and *age-class* (two levels, ≤65 and ≥66). For the longitudinal analyses, plasma level was described by two levels (above or below threshold). The *sex x visit* and *age x visit* terms were included because of the significant differences in baseline severity between males and females and the likely influence of age given that the age range of patients in this study was wide (42–79 years). Since severity was shown to be a significant parameter in the primary ACE-R and FAQ analyses, severity as determined by MMSE score was used as a rate term in the model as *MMSE-class x visit*. There were too few cases taking LMTM in combination with AD-labelled drugs in whom plasma concentration and efficacy data were both available to permit separate analyses according to co-medication status to be conducted. Pharmacodynamic analyses were undertaken independently by ICPD and were confirmed by HS and BOS.

A further analysis was undertaken (HS, BOS) using a modified form of the Hill equation which is commonly used in the analysis of concentration-response data [62] in order to estimate the minimum and maximum plasma concentrations for expected treatment response over 52 weeks. The Hill equation was applied under the assumption of non-cooperativity and used imposed zero values where the no-effect level was taken as -12 ACE-R units, 8 FAQ units or –30 cm^3^ for whole brain volume at a C_max,ss_ concentration of 0.29 ng/ml based on visual inspection of the data. Use of different limiting values did not meaningfully change the results. In addition, a linear term was added to permit trends occurring at high concentrations to be included in the model. For decline in whole brain volume at this dose, patients were split further into terciles to permit estimation of the maximum limiting concentration at which the treatment effect was lost. The modified Hill equation was applied to the data in the form: 

*change* *in* *parameter* = *E*_*min*_ - (*E*_*max*_ *x* ([*C*]- 0.29))/(*EC*_50_ + ([*C*] -0.29)) + (*A* *x*  ([*C*] -0.29))

where *E_*min*_* and *C_*min*_* are the imposed zero values, where *E* is the mean treatment response for any given C_max,ss_ subgroup; *E_*max*_* is the maximum treatment effect as estimated from a standard Hill equation without the additional linear term; *EC_50_* is the C_max,ss_ at which the treatment effect is 50% of the maximum response as estimated from a standard Hill equation without the additional linear term; *A x (C_*max*,*ss*_* – *C_*min*_)* is a further linear term in which *A* is estimated by the model to take account of the trends seen at high concentrations. In order to relate C_max,ss_ values to theoretical doses in a future trial, a linear model was fitted to the mean plasma concentrations at the 8, 150, 200, and 250 mg/day doses using data from both AD patients, and 8 mg/day and 200 mg/day from bvFTD patients:

*estimated dose* = *22.22 x (C_*max*,*ss*_* – *0.016)*

where dose is in mg/day and C_max,ss_ is in ng/ml units.

### Role of the funding source

The funder of the study (TauRx) took the lead in study design, undertaking the study, data interpretation, and initial drafting of the report.

## RESULTS

### Study disposition and population characteristics at baseline

**Fig.1:**
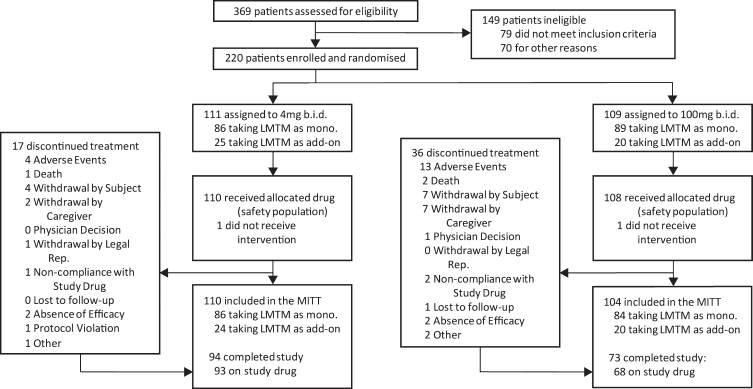
Trial profile.

The disposition of patients randomized to Study 007 is shown in Fig. 1.

Of 1,030 patients who were initially thought to be suitable for inclusion in the trial by investigators, 810 (78.6%) were found to be ineligible either during preliminary assessment prior to screening (661, 64.2%) or at formal screening (149, 14.5%), with only 220 (21.4%) enrolled and randomized. Reasons relating to diagnosis (clinical and MRI imaging) and severity accounted for the majority exclusions (309, 30.0%). Only 18 (1.7%) exclusions were due to meeting DSM IV criteria for other psychiatric conditions, whereas 71 (6.6%) were due to other neurological disorders and a further 81 (7.9%) were found not to meet criteria for probable bvFTD, with the majority of these excluded prior to formal screening. As expected, the majority of medical exclusions (38 from a total of 47 [5.8%]) emerged only during formal screening. Operational reasons represented the second largest group of exclusions overall (227, 28.0%), including unwillingness to participate (86, 8.3%), inability to comply with study procedures (64, 6.2%), and residence in continuous care facility (26, 2.5%) as the largest categories. Overall, therefore, only 20% of patients that investigators initially thought might be suitable met the criteria for enrolment.

The demographic characteristics of the 220 patients randomized are summarized in Table 1. Mean age was 63.3 years, ranging from 42 to 79 years, with more men (63%) than women (37%). Patients were distributed geographically between Europe (46%), North America (41%), and Australia/Asia (13%).

**Table 1:** Demographic characteristics of the randomized Intent to Treat Population

Characteristic	Hydromethylthionine 8 mg/day (*n* = 111)	Hydromethylthionine 200 mg/day (*n* = 109)	Total
Age (y)
Mean (SD)	63.1 (7.35)	63.6 (7.52)	63.3 (7.42)
Median (range)	63.0 (43–78)	64.0 (42–79)	63.0 (42–79)
*n* (%) <60 y	32 (28.8%)	27 (24.8%)	59 (26.8%)
*n* (%) ≥60 y	79 (71.2%)	82 (75.2%)	161 (73.2%)
Sex *n* (%)
Male	67 (60.4%)	71 (65.1%)	138 (62.7%)
Female	44 (39.6%)	38 (34.9%)	82 (37.3%)
Race *n* (%)
American Indian or Alaska Native	4 (3.6%)	4 (3.7%)	8 (3.6%)
Asian	4 (3.6%)	3 (2.8%)	7 (3.2%)
Black or African American	0	1 (0.9%)	1 (0.5%)
White	102 (91.9%)	99 (90.8%)	201 (91.4%)
Other	1 (0.9%)	2 (1.8%)	3 (1.4%)

Patients had been diagnosed with bvFTD for almost 2 years on average (median, 1.1 years; ranging up to 17.6 years). The mean MMSE (SD) at baseline was 24.6 (3.1) with an almost equal distribution of patients with a score of 22–26 (85 patients, 39%) and greater than 26 (81 patients, 37%). Females were more impaired than males on the MMSE scale (males 25.4 (3.5), females 22.9 (4.0), *p* < 0.001) and ACE-R (males 72 (16), females 62 (14), *p* < 0.001) scales, although not on the FAQ or FRS scales. Severity of frontotemporal atrophy was predominantly Kipps stages 2 or 3 (82.3%) with 17.7% at Kipps stage 4 [51] (Table 2). Baseline disease characteristics were similar in distribution across treatment groups, regions, and centers. Comparative summaries of baseline biological characteristics of patients in the present bvFTD study and the AD study populations reported previously [44, 45] are provided in Supplementary Tables 1–3.

**Table 2:** Clinical characteristics of patients at baseline

Characteristic	Hydromethylthionine 8 mg/day (*n* = 111)	Hydromethylthionine 200 mg/day (*n* = 109)	Total
Years since bvFTD diagnosis
*n*	107	106	213
Mean (SD)	1.9 (2.4)	1.9 (2.4)	1.9 (2.4)
Median (range)	0.9 (0.0–14.9)	1.1 (0.0–17.6)	1.1 (0.0–17.6)
MMSE score
Mean (SD)	24.6 (3.1)	24.7 (3.0)	24.6 (3.1)
≤21	28 (25.2%)	26 (23.9%)	54 (24.5%)
22–26	46 (41.4%)	39 (35.8%)	85 (38.6%)
>26	37 (33.3%)	44 (40.4%)	81 (36.8%)
Kipps stage, *n* (%)
2	38 (34.2%)	42 (38.5%)	80 (36.4%)
3	55 (49.5%)	46 (42.2%)	101 (45.9%)
4	18 (16.2%)	21 (19.3%)	39 (17.7%)

Co-medication utilization is summarized in Table 3. Approximately one-fifth of all patients (21% overall) were receiving an AChEI and/or memantine therapy at screening, while almost half (46%) were using medical food or alternative pharmacotherapy. Antidepressant serotonergic reuptake inhibitors were used by 29%, and drugs with potential serotonergic activity were taken by 50%.

**Table 3:** Co-medication status of patients in study

Characteristic	Hydromethylthionine 8 mg/day (*n* = 111)	Hydromethylthionine 200 mg/day (*n* = 109)	Total
Use of AChEI/memantine (Concomitant medication) *n* (%)
AChEI and/or memantine	25 (22.5%)	20 (18.3%)	45 (20.5%)
Both AChEI and memantine	7 (6.3%)	3 (2.8%)	10 (4.5%)
AChEI only	10 (9.0%)	7 (6.4%)	17 (7.7%)
Memantine only	8 (7.2%)	10 (9.2%)	18 (8.2%)
Use of Medical Food or Alternative Pharmacotherapy for Dementia *n* (%)
Yes	54 (48.6%)	49 (45.0%)	103 (46.8%)
No	57 (51.4%)	60 (55.0%)	117 (53.2%)
Use of Selective Serotonin Reuptake Inhibitor (SSRI) *n* (%)
Yes	30 (27.0%)	33 (30.3%)	63 (28.6%)
No	81 (73.0%)	76 (69.7%)	157 (71.4%)
Use of Drugs of Serotonergic Potential *n* (%)
Yes	49 (44.1%)	62 (56.9%)	111 (50.5%)
No	62 (55.9%)	47 (43.1%)	109 (49.5%)

### Validation of diagnosis and genetic mutations

**Fig.2:**
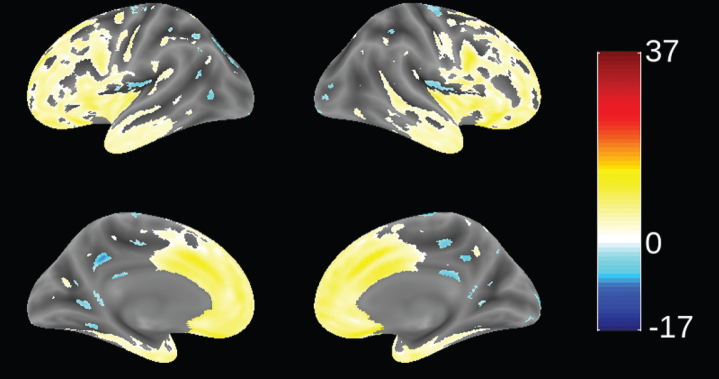
Voxel-based morphometric comparison showing regions of greater atrophy (yellow) in patients with bvFTD compared to AD patients with mild AD (*n* = 713), from study TRx-237-005, controlled for age, sex, and total intracranial volume of each individual. Blue color indicates greater atrophy in AD patients. Data are displayed at a significance threshold corrected for family-wise error at the whole brain level at *p* < 0.05.

Since patients in this study were recruited from 70 trial sites in 13 countries (not necessarily from FTD specialist centers), we sought to determine the extent to which patients meeting consensus clinical criteria [1] and the further requirement for evidence of definite frontal and/or temporal lobe atrophy on MRI scan [51] were distinct from mild AD. This was examined by using VBM to compare the distribution of brain atrophy in patients in this study with those enrolled in a parallel study of mild AD (TRx-237-005) in which similar MRI procedures were used. This comparison is shown in Fig. 2. bvFTD patients in this study had significantly more atrophy in frontal cortex and anterior temporal cortex, and significantly less atrophy in hippocampus, middle temporal gyrus, cuneus, and insula. We have also reported recently that bvFTD patients in this study had significantly more atrophy in frontal cortex than the other lobes. This contrasts with the AD group, which showed more distributed patterns of atrophy and with more atrophy in temporal, parietal, and occipital lobes than the bvFTD group [63]. The MRI differentiation between bvFTD and AD patients is consistent with the high degree of confidence of a clinical diagnosis of bvFTD previously reported in a clinicopathological study [23].

Of 159 cases analyzed genetically, seven were found to have mutations in coding regions for either tau (6, 3.8%) or TDP-43 (1). This is similar to the frequency previously reported for bvFTD patients having tau mutations (3%) [23]. For the one case with a TDP-43 mutation, the mutation was I383V. There were four cases with a P301L mutation in the tau coding region, one with G272V, and one with R406W. Treatment effects were not analyzed separately in these cases.

### Prespecified efficacy analyses

There were no significant differences on any of the primary or secondary outcomes at 52 weeks between patients receiving hydromethylthionine 200 mg/day and those receiving 8 mg/day as randomized and as prespecified in the Statistical Analysis Plan. These results are shown in Table 4.

**Table 4:** Modeled difference in change from baseline for the respective endpoints comparing patients receiving hydromethylthionine 8 mg/day and 200 mg/day on primary and secondary outcomes. (UPDRS, although a safety outcome, is also included.)

	Decline±SEM for LMTM 8 mg/day group (*n* = 110)	Difference±SEM from LMTM 8 mg/day for 200 mg/day (*n* = 104)	CI	*p*
ACE-R	–9.98±1.39	–0.49±2.10	–4.64, 3.66	0.8170
FAQ	5.51±0.55	–0.39±0.84	–2.05, 1.26	0.6410
WBV (cm^3^)	–21.64±1.56	–1.35±2.32	–5.94, 3.23	0.5614
MMSE	–3.41±0.53	–0.12±0.80	–1.69, 1.42	0.8836
CGIC	–1.05±0.11	–0.04±0.16	–0.35, 0.28	0.8252
FRS	–0.1240±0.0136	0.0075±0.0206	–0.0332, 0.0482	0.7176
ACE-III	–10.70±1.55	0.44±2.27	–4.05, 4.93	0.8469
UPDRS – part III	3.90±1.06	–1.40±1.61	–4.59, 1.78	0.3850

### Analyses based on population PK data

Data for plasma concentration and time from taking the first dose were available for 171 patients out of 220. Patients receiving hydromethylthionine at a dose of 8 mg/day were split into four groups based on C_max,ss_ to provide an independent biological classification according to drug exposure at this dose. The plasma concentration ranges used for this purpose are as indicated in Table 5. The cut-off that defined the upper limit of the lowest 35% group (corresponding to the percentage of patients with plasma levels below the validated limit of quantitation on Day 1; 32 patients) was 0.346 ng/ml. The remainder with Day 1 plasma levels within the validated calibration range of the assay were distributed into terciles having approximately 20 patients (22%) each. Small changes in the group boundaries did not meaningfully change the results. As expected, plasma levels in the 80 patients receiving the 200 mg/day dose were 16-fold higher than the highest level seen at 8 mg/day.

**Table 5:** Plasma-modeled parent MT C_max,ss_ for hydromethylthionine groups

Dose groups	C_max,ss_ (ng/ml)		
	*n* (%)	Mean (SD)	Range
8 mg/day
8 mg/day – Group 1	32 (35%)	0.321 (0.0198)	0.281–0.346
8 mg/day – Group 2	20 (22%)	0.355 (0.0082)	0.346–0.372
8 mg/day – Group 3	19 (21%)	0.387 (0.0121)	0.373–0.409
8 mg/day – Group 4	20 (22%)	0.470 (0.0537)	0.413–0.583
200 mg/day	80	9.040 (1.6259)	6.800–14.235

**Fig.3:**
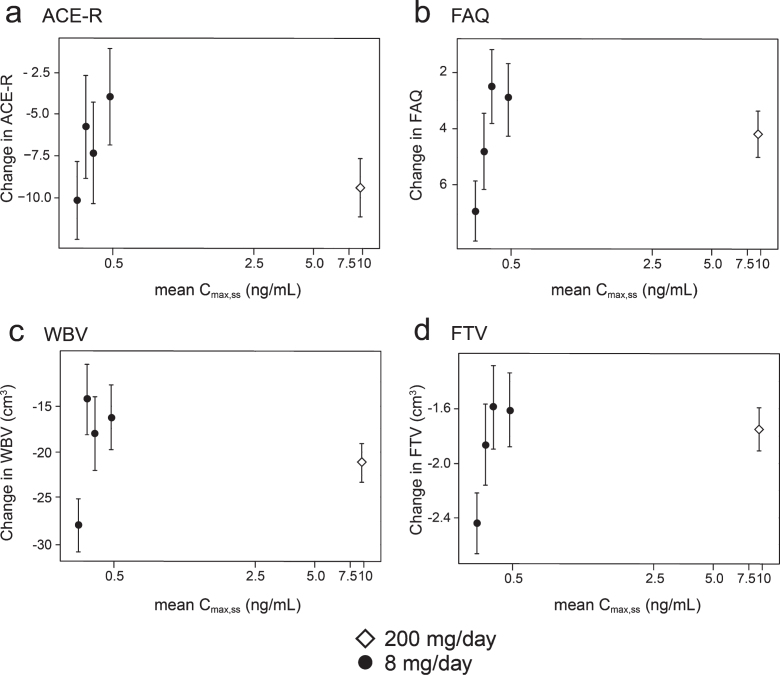
Model-derived least square mean and standard error estimates of change of 52 weeks for clinical (a, b) and MRI volumetric endpoints (c, d) according to plasma concentration groups (8 mg/day) or dose (200 mg/day).

Concentration-response relationships are shown (Fig. 3) for change in ACE-R, FAQ, WBV, and FTV over 52 weeks for all patients in whom data were available for estimated C_max,ss_ of parent MT in plasma.

As can be seen, there are steep concentration-response relationships for plasma levels in the range 0.3–0.6 ng/ml at the 8 mg/day dose on clinical and MRI outcomes. The patients with plasma C_max,ss_ levels less than 0.346 ng/ml have worse outcomes than those with levels above this threshold at the 8 mg/day dose. At substantially higher plasma concentrations, in the range 6.8–14.2 ng/ml seen in patients receiving the 200 mg/day dose, treatment effects are also generally worse.

**Fig.4:**
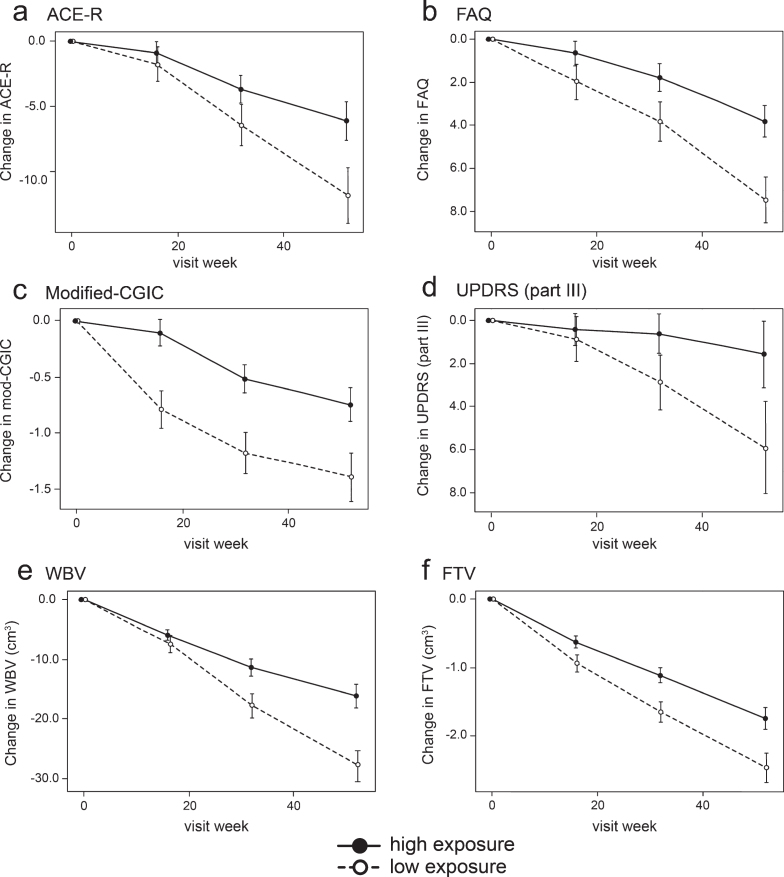
Model-derived least square mean and standard error estimates of change of 52 weeks for clinical (a, b, c, d) and MRI volumetric endpoints (e, f) in patients taking hydromethylthionine 8 mg/day categorized by plasma levels above or below the C_max,ss_ threshold of 0.346 ng/ml.

In order to inform a future confirmatory study, binary outcome analyses were performed in which the group of patients with minimal systemic exposure to the drug was used as a proxy for placebo. These are shown in Table 6 and illustrated in Fig. 4.

**Table 6:** Binary pharmacological activity analysis based on a plasma parent MT threshold of 0.346 ng/ml to define a proxy for placebo; modelled difference in change from baseline for the respective endpoints

		All patients					Patients receiving hydromethylthionine 8 mg/day				
	Decline±SEM for C_max,ss_ ≤0.346 ng/ml	Difference±SEM for C_max,ss_ > 0.346 ng/ml	CI	*p*	Nlow	Nhigh	Difference±SEM for C_max,ss_ > 0.346 ng/ml	CI	*p*	Nlow	Nhigh
ACE-R	–11.33 ± 2.09	1.37±2.60	–3.73, 6.47	0.5973	30	130	5.06±2.62	–0.08, 10.21	0.0536	30	60
FAQ	7.13 ± 1.06	–2.98±1.10	–5.15, –0.82	0.0069	30	129	–3.27±1.32	–5.85, –0.69	0.0131	30	60
WBV (cm^3^)	–27.72 ± 2.73	9.05±3.06	3.06, 15.04	0.0031	28	115	11.67±3.41	5.00, 18.36	0.0006	28	52
LVV (cm^3^)	9.13 ± 0.82	–3.41±–0.95	–5.27, –1.55	0.0003	28	107	–4.12±1.06	–6.19, –2.05	<0.0001	28	46
FTV (cm^3^)	–2.47 ± 0.22	0.73±0.24	0.26, 1.19	0.0023	28	115	0.72±0.27	0.19, 1.26	0.0076	28	52
FRS	–0.14 ± 0.03	0.04±0.03	–0.02, 0.10	0.1527	30	130	0.04±0.03	–0.03, 0.10	0.2519	30	60
CGIC	–1.34 ± 0.20	0.42±0.22	–0.02, 0.85	0.0623	30	130	0.64±0.27	0.12, 1.17	0.0157	30	60
MMSE	–2.95 ± 0.93	–0.43±1.05	–2.48, 1.62	0.6788	30	130	0.41±1.04	–1.64, 2.45	0.6974	29	58
ACE-III	–9.47 ± 2.40	1.27±2.73	–4.08, 6.61	0.6418	26	122	4.36±2.94	–1.40, 10.13	0.1380	29	58
UPDRS-part III	5.71 ± 1.85	–3.27±2.09	–7.37, 0.81	0.1163	29	123	–4.31±2.69	–9.58, 0.95	0.1085	31	62

Compared to patients with subthreshold plasma concentrations, patients with plasma C_max,ss_ above the 0.346 ng/ml threshold at the 8 mg/day dose have significantly better outcomes on FAQ, Modified-CGIC, WBV, LVV, and FTV, and near-significant differences on ACE-R and UPDRS. There are no exposure-dependent differences in MMSE or FRS. Treatment differences are generally larger for comparisons restricted to patients receiving the 8 mg/day dose. This is due to inclusion of patients receiving the 200 mg/day dose who have worse outcomes than patients with above-threshold plasma levels at the 8 mg/day dose.

**Fig.5:**
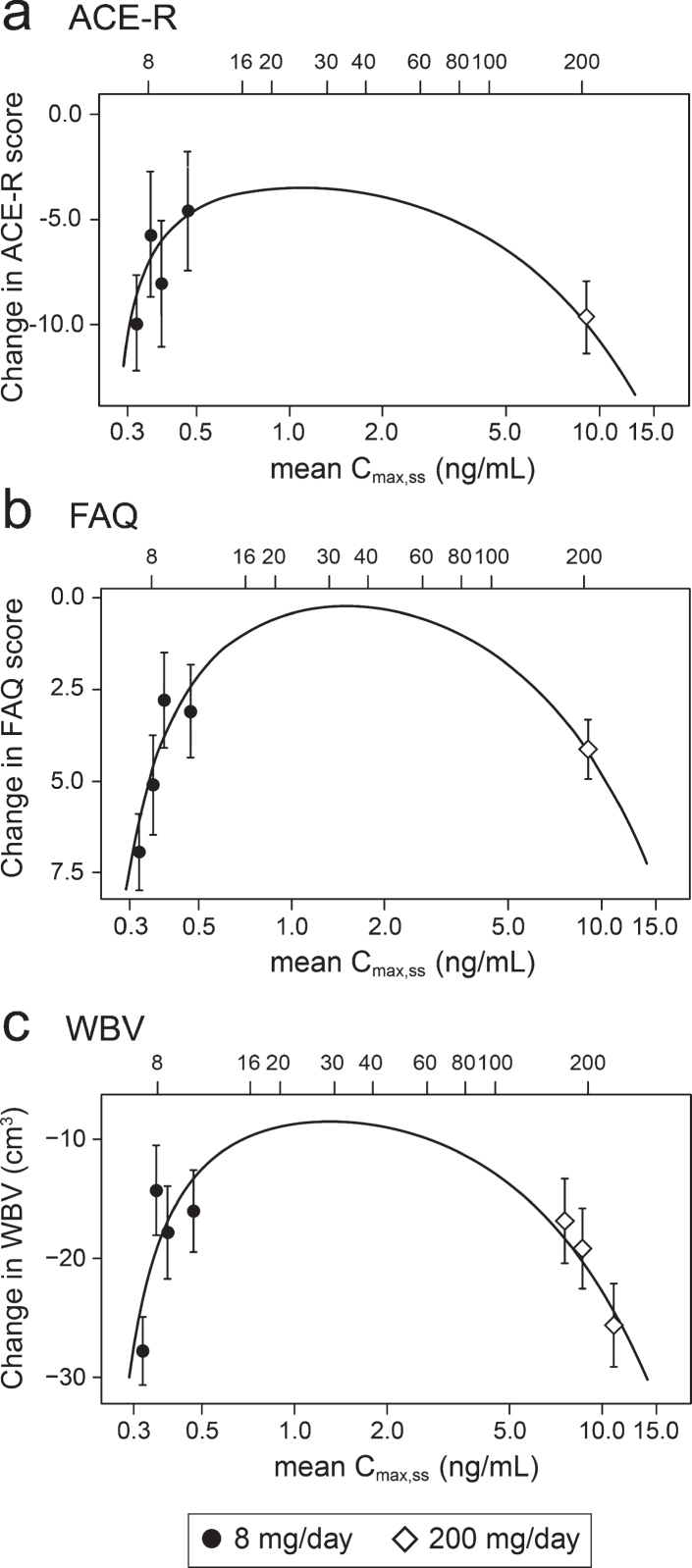
Expanded Hill equation analysis of pharmacological activity of hydromethylthionine on change in ACE-R, FAQ, and WBV using model-derived least squares mean and standard error estimates of change over 52 weeks for clinical (a,b) and MRI volumetric (c) endpoints according to plasma concentration group (8 mg/day) or dose (200 mg/day). Scales at the top of each panel indicate actual doses or calculated theoretical intermediate doses.

In order to understand the exposure-response relationship better, the data were explored further using an expanded version of the Hill equation [62]. This provided a robust fit to the mean concentration-response for change in ACE-R, FAQ, and WBV over 52 weeks (Fig. 5). The model fit for these outcomes is consistent with the assumption that the lower limiting plasma concentration required for treatment response is 0.29 ng/ml in patients receiving the 8 mg/day dose. The whole brain volume data in patients receiving the 200 mg/day dose were sufficiently homogeneous to permit further subgrouping into terciles (Fig. 5). This made it possible to derive an estimate of the upper limiting concentration (13.6 ng/ml, corresponding to a theoretical dose of 301 mg/day) at which the treatment effect would be lost (Fig. 5).

Using data from the combined AD and bvFTD populations, we have reported previously that a dose of 16 mg/day would be sufficient to ensure that all patients have plasma levels above the threshold required for pharmacological activity [49]. However, the Hill equation analyses suggests that pharmacological activity is predicted to peak in the vicinity of a 30 mg/day dose (Fig. 5). At this dose, the treatment effect on the FAQ scale is predicted to be 7.73 [CI 5.50, 12.81] units (mean and confidence interval based on bootstrap analysis), and the treatment effect on whole brain atrophy is predicted to be 18.52 cm^3^ [CI 14.29 cm^3^, 23.70 cm^3^]. A simulation of the plasma concentration profile for 30 mg/day, given as single daily dose, is shown in the Supplementary Figure 1.

### Safety outcomes

The safety evaluation was based on the 218 patients who enrolled in this study and took at least one dose of hydromethylthionine (Safety Population). Of the 218 patients in the Safety Population, 110 (50.5%) were randomized to 8 mg/day and 108 (49.5%) were randomized to 200 mg/day. The compliance was high, 97% for 8 mg/day and 94% for 200 mg/day patients meeting a criterion defined as between 80% and 120% of planned doses based on tablet returns. The 8 mg/day dose was better tolerated, with a withdrawal rate of 15% over 12 months compared with 38% for the 200 mg/day dose.

Treatment emergent adverse events (TEAEs) were reported by 88 (80%) patients in the 8 mg/day group and 103 (95.4%) patients in the 200 mg/day group. TEAEs were judged by the Investigator to be related to study drug in 30% of patients in the 8 mg/day group and 56% of patients taking 200 mg/day. The majority of TEAEs were mild to moderate in intensity. A summary of TEAEs with an incidence of ≥5% of patients in any treatment group is provided in Table 7. TEAEs that showed an increase in incidence with hydromethylthionine 200 mg/day relative to 8 mg/day include anemia, diarrhea, dysuria, nausea, pollakiuria, insomnia, and vomiting. Renal and urinary disorders were reported with greater frequency in patients receiving hydromethylthionine 8 mg/day with above-threshold plasma levels (26%) compared with those with below-threshold levels (18%). At least one severe TEAE was reported in 13 (12%) hydromethylthionine 8 mg/day patients and 17 (16%) in 200 mg/day patients. More patients (35 [32%]) treated with hydromethylthionine 200 mg/day experienced a TEAE resulting in a dose reduction, interruption, or withdrawal of study drug as compared to patients treated with hydromethylthionine 8 mg/day (15 [14%]). The UPDRS was included to ensure there was no adverse effect on motor function. Patients did not have evidence of impaired motor function at the start of treatment and there was no worsening over time.

**Table 7:** Number (%) of patients with at least one TEAE with an incidence of ≥5% of patients in any treatment group – Safety Population

MedDRA System Organ Class/Preferred Term	Hydromethylthionine 8 mg/day (*n* = 110)	Hydromethylthionine 200 mg/day (*n* = 108)
Patients Reporting at Least One Adverse Event	88 (80.0%)	103 (95.4%)
**Gastrointestinal disorders**	**28 (25.5%)**	**42 (38.9%)**
Diarrhea	9 (8.2%)	24 (22.2%)
Vomiting	5 (4.5%)	9 (8.3%)
**Infections and infestations**	**38 (34.5%)**	**34 (31.5%)**
Nasopharyngitis	8 (7.3%)	7 (6.5%)
Urinary tract infection	13 (11.8%)	14 (13.0%)
**Injury, poisoning and procedural complications**	**18 (16.4%)**	**16 (14.8%)**
Fall	10 (9.1%)	13 (12.0%)
**Investigations**	**17 (15.5%)**	**34 (31.5%)**
Blood folate decreased	6 (5.5%)	9 (8.3%)
White blood cells urine positive	1 (0.9%)	6 (5.6%)
**Metabolism and nutrition disorders**	**12 (10.9%)**	**14 (13.0%)**
Folate deficiency	2 (1.8%)	6 (5.6%)
**Nervous system disorders**	**33 (30.5%)**	**27 (25.0%)**
Headache	8 (7.3%)	5 (4.6%)
**Psychiatric disorders**	**29 (26.4%)**	**31 (28.7%)**
Agitation	9 (8.2%)	9 (8.3%)
Insomnia	3 (2.7%)	7 (6.5%)
**Renal and urinary disorders**	**25 (22.7%)**	**28 (25.9%)**
Dysuria	0	6 (5.6%)
Pollakiuria	3 (2.7%)	10 (9.3%)
Urinary incontinence	13 (11.8%)	9 (8.3%)
**Respiratory, thoracic and mediastinal disorders**	**12 (10.9%)**	**8 (7.4%)**
Cough	7 (6.4%)	2 (1.9%)

Protocol-specified adverse events of special interest included significant hematological findings (methemoglobinemia, hemolytic anemia, presence of Heinz bodies), serotonin syndrome, and ARIA. The frequency of affirmative C-SSRS responses prior to treatment was the same for both dosage groups (8 mg/day, 7 (6.4%); 200 mg/day, 6 (5.6%)) and remained unchanged following treatment. There was one aborted suicide attempt in a patient who had a prior history of an attempt that was not reported as a TEAE and the patient continued with treatment. There was 1 death among patients receiving hydromethylthionine at the 8 mg/day dose, and 3 in patients exposed to the 200 mg/day dose. None of these events was judged by the investigator as being related to treatment. Patients were closely monitored for evidence of possible serotonin syndrome/toxicity using a 20-item structured examination based on published criteria [64, 65]. No subject had serotonin syndrome reported as an AE. Three subjects (2 randomized to 8 mg/day and 1 randomized to 200 mg/day) had myoclonus reported as an AE. However, none of these was considered by investigators to be due to serotonin toxicity after clinical review.

## DISCUSSION

We report the results of the largest therapeutic trial conducted in bvFTD to date along with an embedded population PK analysis. The study was designed to compare a high dose of hydromethylthionine (200 mg/day) with a low dose (8 mg/day) intended as a control. As in two similarly designed trials in AD [44, 45], there were no significant differences between these two doses on any efficacy outcome. In AD, we have reported recently that there are steep concentration-response relationships on cognitive and MRI outcomes for steady state plasma levels in the range 0.3–0.8 ng/ml at the 8 mg/day dose, and that plasma concentrations in the range 4–21 ng/ml produced by doses in the range 150–250 mg/day are not associated with any additional benefit [49]. We now report a similar exposure-response profile in bvFTD. There are steep concentration-response relationships on clinical and MRI outcomes for steady state plasma levels in the range 0.3–0.6 ng/ml on the clinical ACE-R and FAQ scales and whole brain atrophy measured by MRI. High plasma concentrations, in the range 7–14 ng/ml for subjects receiving the 200 mg/day dose, produce worse outcomes.

The exposure-response space we have now been able to define permits a better understanding of the relationship between dose and pharmacological activity. The lack of dose-response comparing hydromethylthionine at 8 mg/day with 200 mg/day is due to two main factors. First, there is a steep exposure-response relationship at the 8 mg/day dose such that the majority of patients have blood levels sufficient for pharmacological activity on clinical and MRI volumetric outcomes. Second, there is a biphasic response at concentrations substantially higher than required for pharmacological activity such the pharmacological activity at 200 mg/day is either no greater or less compared with that seen in patients with adequate blood levels at the 8 mg/day dose. Modelling using an expanded version of the Hill equation produced robust fits to the clinical and MRI data. This analysis suggests that 0.29 ng/ml is the minimum limiting plasma concentration of the drug required for pharmacological activity, and that 13.6 ng/ml (corresponding to a theoretical dose of 301 mg/day) is the upper limiting concentration for activity.

We have reported recently that variability in exposure at the 8 mg/day dose is determined primarily by renal function as measured by creatinine clearance [49]. Patients with high creatinine clearance have relatively lower steady state plasma levels of the drug and vice versa. Since the bvFTD population is younger and the mean creatinine clearance is higher, a higher dose of hydromethylthionine may be required than in AD. Whereas the predicted optimal dose for treatment of AD is about 16 mg/day, we estimate that a dose in the vicinity of 30 mg/day is required in bvFTD. The Hill equation analysis suggests that a broad range of doses (20–60 mg/day) are likely to produce roughly comparable treatment effects and would provide an adequate range for dosing flexibility in clinical practice.

The doses of hydromethylthionine required for pharmacological activity are substantially lower than that previously identified for MTC in AD [47]. Hydromethylthionine has better brain delivery of LMT than does MTC following oral dosing [31, 49]. This is due in part to 20-fold more efficient uptake into red blood cells which is needed for the drug to escape first-pass metabolism which leads to loss of activity [26], and a corresponding 60-fold or greater brain:plasma ratio compared to MTC [49]. The plasma concentration threshold of 0.346 ng/ml required for clinical pharmacological activity in bvFTD is very close to that identified in mild/moderate AD (C_max,ss_ 0.373 ng/ml). Likewise, the non-linear concentration-response profile is similar to that seen in AD [49], although the biphasic response profile is more pronounced in bvFTD. Both diseases are characterized by pathology of protein aggregation. The protein involved is TDP-43 in more than half of bvFTD cases, whereas in AD it is tau protein in the majority of cases, with an unknown contribution of cases with limbic TDP-43 pathology [66]. The estimated brain concentration of hydromethylthionine required for pharmacological activity (0.02–0.06*μ*M) [49] is consistent with its activity as an inhibitor of both tau [27] and TDP-43 [65] aggregation [34] *in vitro*, and with the doses required for activity in a tau transgenic mouse model of bvFTD [31]. In addition to different clinical manifestations and response to symptomatic treatments, the underlying pathology affects different classes of brain cells with a different neuroanatomical distribution in AD and bvFTD [67–69]. It is therefore striking that hydromethylthionine has similar pharmacological activity with respect to clinical decline and brain atrophy over similar concentration ranges of the drug in two distinct neurodegenerative disorders.

In addition to effects on pathological protein aggregation, the MT moiety has been reported to have effects on dopamine and serotonin. Bhurtel and colleagues reported that MTC (given in the oxidized MT^+^ form as MTC at a dose of 20 mg/kg intraperitoneally) restores dopamine levels in a mouse model of Parkinson’s disease using 1-methyl-4-phenyl-1,2,3,6-tetrahydropyridine (MPTP) [70]. MPTP is not itself toxic, but produces toxicity in dopaminergic neurons via a metabolite produced by monoamine oxidase in the brain [71]. However, no effect was seen when MTC was given alone, suggesting that the primary mechanism is via an interaction with monoamine oxidase. It has been reported that MT^+^ inhibits monoamine oxidase (IC_50_ 5.5*μ*M; [72]) and serotonin transporter (IC_50_ 1.2*μ*M; [73]) activity *in vitro*. Since the estimated brain concentrations of hydromethylthionine required for clinical pharmacological activity in bvFTD are more than an order of magnitude below these values it is unlikely that the treatment effects seen clinically are mediated by a direct effect on serotonin neurotransmission. Using a tau transgenic mouse model, we have recently reported that hydromethylthionine increases acetylcholine levels in the hippocampus and normalizes glutamate release in synaptosomes prepared from brain tissues at clinically relevant doses. It is therefore likely that, in addition to direct effects on protein aggregation pathology, there are secondary effects in neurotransmitter systems compromised by pathology.

We have used the results from this study to inform the design of a planned placebo-controlled trial. For this purpose, we defined a subthreshold patient group with minimal drug exposure as a proxy for placebo. The study was powered to detect a 50% reduction in the expected annual decline over 52 weeks for patients with definite evidence of fronto-temporal atrophy estimated to be 13.4±2.1 ACE-R units based on an observational study [51]. The decline observed in patients with minimal drug exposure as defined by the 0.346 ng/ml threshold is 11.3±2.1 ACE-R units, and is therefore comparable, although a more recent study in patients meeting only clinical diagnostic criteria reported a smaller decline [4]. Other than the Kipps et al. study [51], which included MRI evidence of fronto-temporal atrophy to exclude phenocopies, other available studies had only clinical requirements using either the Neary et al. [74] or Rascovsky et al. [1] criteria. In the comparisons which follow, we have used the published data to calculate standard errors for comparison. The annualized decline on the FAQ scale was reported as 5.81±1.11 units in bvFTD [21], 5.6 to 8.8 units (extrapolated from 26-week data in patients randomized to either placebo or memantine [18]), and 3.73±0.39 [75]. The decline observed in patients with subthreshold plasma levels was 7.13±1.06 units. The ADCS-CGIC score after 12 months was reported as 5.27 (SD 0.88) [21], corresponding to a change score of –1.27±1.11 units. The decline on the Modified-CGIC in patients with subthreshold plasma levels was –1.34±0.20 units. The annualized decline on the MMSE scale has been reported as –2.45±0.79 [21] and –3.98±0.54 [75]. The MMSE decline observed in patients with subthreshold plasma levels was –2.95±0.93 units. Finally, the decline in whole brain volume reported in a bvFTD cohort was 20.8 cm^3^ [76]. The corresponding decline in patients with subthreshold plasma levels was 27.7±2.7 cm^3^. Therefore, allowing for differences in patient selection criteria, the decline observed across multiple metrics in patients with minimal drug exposure is comparable to that reported for patients in historical studies. Compared against patients with subthreshold plasma levels, the treatment differences for patients receiving 8 mg/day with plasma levels above the 0.346 ng/ml threshold are 5.1 units (*p* = 0.0536, 45% reduction) on the ACE-R scale, –3.3 units (*p* = 0.0131, 46% reduction) on the FAQ scale, 0.64 Modified-CGIC units (*p* = 0.0157, 48% reduction), –4.3 UPDRS units (*p* = 0.0711, 75% reduction) and 11.7 cm^3^ (*p* = 0.0006, 42% reduction) in whole brain atrophy. These binary exposure-dependent differences cannot be accounted for by differences in severity, sex, age, co-medication status with AD drugs, or geography since these variables are corrected for in the concentration-response analysis model.

There have been several proposals regarding selection of primary and secondary outcomes for studies in bvFTD, based on historical observational data, theoretical treatment effect sizes and other theoretical considerations [21, 75–78]. Unlike AD, where trial methodology has evolved over a 40-year period and a consensus has emerged as to the domains which should be assessed as primary outcomes, there is no general agreement as to the best ways to measure treatment effects in bvFTD. In a recent review, 50 different primary and secondary outcome scales are listed for clinical trials reported in bvFTD between 2003-2015 [78]. Based on the present results, FAQ and Modified-CGIC appear to have the greatest power as clinical outcomes in practice, consistent with previous suggestions [75, 76], and change in whole brain [76] or lateral ventricular volume have the greatest power as supportive biomarker outcomes. In contrast to AD, the functional outcome measure (FAQ) performs better than the cognitive outcome measures (ACE-R and ACE-III) we have used. It also has greater clinical relevance and face validity [53]. An apparent effect on the UPDRS motor assessment scale is surprising, although involvement of basal regions early in the disease process has been reported [79, 80].

The safety profile of hydromethylthionine seen in the present study is very similar to that seen in the two AD studies we have reported. Comparing 200 mg/day with 8 mg/day, there is a dose-dependent increase in adverse events recorded as anemia, dysuria, pollakiuria, diarrhea, nausea, insomnia, and vomiting. In patients receiving the 8 mg/day dose, the higher frequency of renal and urinary adverse events in patients with above- versus below-threshold plasma levels (26% versus 18%) is consistent with higher plasma levels. At a dose of 8 mg/day, the safety profile is generally benign. This dose is well tolerated, with a withdrawal rate of 15% over 12 months, compared with 38% for the 200 mg/day dose.

There are important limitations in the inferences which can be drawn from the present study. A *post-hoc* exposure-response analysis of the kind we have undertaken does not prove efficacy. It provides a means of determining how pharmacological activity is related to plasma concentration, and hence dose. However, pharmacological activity is not the same thing as efficacy. Efficacy can be established only by demonstrating a statistically significant effect on prespecified outcomes in a suitable randomized placebo-controlled trial. The analyses presented here are hypothesis generating and serve the purpose of informing the design of a confirmatory placebo-controlled trial which is now planned. The group sizes in the analyses comparing exposure-dependent differences at the 8 mg/day dose are small (46–62 with high exposure against 28–31 with low exposure), although comparable with other studies conducted in bvFTD [14–19]. The fact that there are consistent exposure-dependent differences achieving nominal significance on a range of clinical and MRI outcomes based on relatively small numbers of subjects suggests that the effects, if confirmed, are likely to be clinically meaningful.

### Conclusion

We report the results of the largest clinical trial in bvFTD to have been conducted to date, combined with a population PK analysis in 80% of the participating patients in whom drug concentration and efficacy outcome data were available. The randomized comparisons between hydromethylthionine at a dose of 200 mg/day and a low dose of 8 mg/day, thought to be inactive, did not show any difference on the intended primary or secondary outcomes. However, the population PK analyses conducted *post-hoc* imply that 8 mg/day produces significantly better outcomes in patients with adequate exposure to the drug compared to those with minimal exposure at the same dose. Hydromethylthionine shows a biphasic concentration-response profile such that the high plasma levels associated with the 200 mg/day dose produce worse outcomes. In patients with adequate systemic exposure at the 8 mg/day dose, hydromethylthione has pharmacological activity with respect to clinical decline and brain atrophy, reducing decline by almost half compared with patients with minimal drug exposure. If confirmed in a further trial, these would represent potentially meaningful clinical gains in a severely debilitating condition for which no effective treatment options exist at present. The similarity in the concentration-response profiles at the 8 mg/day dose in AD and bvFTD is consistent with a common molecular mechanism of action linked to inhibition of pathological protein aggregation. A placebo-controlled trial in bvFTD aiming to determine whether hydromethylthionine is efficacious in bvFTD is now planned.

### Availability of data and materials

The datasets and analyses used during the current study are available from the corresponding author on reasonable request.

## Supplementary Material

Supplementary Material

Supplementary Material
