# Supplementary material for: Concentration-Dependent Activity of Hydromethylthionine on Clinical Decline and Brain Atrophy in a Randomized Controlled Trial in Behavioral Variant Frontotemporal Dementia
Source: J Alzheimers Dis. 2020 May 19;75(2):501–19. doi: 10.3233/JAD-191173 (PMC7306898; doi:10.3233/JAD-191173)
Supplement: Supplementary Material [file jad-75-jad191173-s001.pdf]

Supplementary Material

Concentration-Dependent Activity of Hydromethylthionine on Clinical Decline and Brain Atrophy in a Randomized Controlled Trial in Behavioral Variant Frontotemporal Dementia

Supplementary Table 1. STUDY TRx-237-007 PopPK Population Variables

Characteristic	Randomized Treatment		Total (N=175)
	LMTM 4 mg/day (N=93)	LMTM 100 mg/day (N=82)	
Weight (kg)			
Mean (SD)	82.7 (18.8%)	81.7 (20.1%)	82.2 (19.4%)
Median (range)	82.5 (44.7-128)	82.4 (42.1-135)	82.5 (42.1-135)
BMI (kg/m^2)			
Mean (SD)	28.5 (17.7%)	27.5 (15.9%)	28.0(16.9%)
Median (range)	27.5 (20.1-46.1)	27.1 (17.1-40.6)	27.3(17.1-46.1)
Creatinine Clearance (mL/min)			
Mean (SD)	74.8 (23.4%)	76.6 (21.0%)	75.6 (22.2%)
Median (range)	72.7 (42.3-121)	76.5 (41.9-127)	73.5 (41.9-127)
eGFR ($mL/min/1.73m^2$)			
Mean (SD)	83.7 (17.7%)	86.1 (18.2%)	84.8 (18.0%)
Median (range)	81.7 (52.9-128)	84.0 (51.6-122)	82.4 (51.6-128)

Supplementary Table 2. STUDY TRx-237-005 PopPK Population Variable

Characteristic	Randomized Treatment		Total (N=674)
	LMTM 4 mg/day (N=344)	LMTM 100 mg/day (N=330)	
Weight (kg)			
Mean (SD)	74.4 (22.8%)	71.7 (21.1%)	73.1 (22.1%)
Median	72.4 (43.5-134)	70.7 (36.8-123)	71.4 (36.8-134)
BMI (kg/m^2)			
Mean (SD)	26.8 (32.7%)	26.2 (17.6%)	26.5 (26.6%)
Median	25.9 (17.4-162)	25.4 (14.4-41.2)	25.5 (14.4-162)
Creatinine Clearance (mL/min)			
Mean (SD)	58.6 (28.8%)	58.3 (28.3%)	58.5 (28.5%)
Median	57.4 (22.2-117)	56.4 (11.9-134)	56.7 (11.9-134)
Creatinine Clearance (mL/min/1.73 m^2)			
Mean (SD)	55.6 (26.4%)	56.6 (25.8%)	56.1 (26.1%)
Median	53.4 (23.9-106)	55.6 (10.1-118)	54.5 (10.1-118)
eGFR (mL/min/1.73 m^2)			
Mean (SD)	74.3 (22.5%)	75.8 (21.4%)	75.1 (22.0%)
Median	72.3 (34.2-134)	76.2 (12.6-128)	74.2 (12.6-134)

Supplementary Table 3. STUDY TRx-237-015 PopPK Population Variables

Characteristic	Randomized Treatment			Total (N=626)
	LMTM 8 mg/day (N=256)	LMTM 150 mg/day (N=187)	LMTM 250 mg/day (N=183)	
Weight (kg)				
Mean (CV)	69.8 (20.0%)	68.7 (19.1%)	69.8 (22.4%)	69.5 (20.5%)
Median (min – max)	69.0 (41.0-132)	68.0 (36.2-110)	68.5 (39.0-108)	68.6 (36.2-132)
BMI (kg/m^2)				
Mean (CV)	26.0 (15.7%)	26.4 (22.5%)	25.5 (18.3%)	26.0 (18.7%)
Median (min – max)	26.0 (15.4-37.6)	25.9 (15.3-82.1)	24.7 (14.9-43.3)	25.6 (14.9-82.1)
Creatinine Clearance (mL/min)				
Mean (CV)	56.8 (29.2%)	58.4 (27.3%)	61.3 (29.2%)	58.6 (28.8%)
Median (min – max)	55.4 (23.0-115)	57.3 (26.5-104)	57.8 (25.7-118)	56.7 (23.0-118)
Creatinine Clearance (mL/min/1.73 m^2)[b]				
Mean (CV)	55.9 (25.4%)	58.6 (25.5%)	60.1 (24.6%)	58.0 (25.3%)
Median (min – max)	55.4 (20.3-94.7)	56.6 (26.3-106)	59.5 (27.3-106)	56.6 (20.3-106)
eGFR (mL/min/1.73 m^2)				
Mean (CV)	74.6 (22.1%)	78.2 (20.2%)	79.7 (20.7%)	77.2 (21.3%)
Median (min – max)	73. 7(30.6-150)	78.0 (34.0-122)	78.9 (37.8-123)	76.5 (30.6-150)

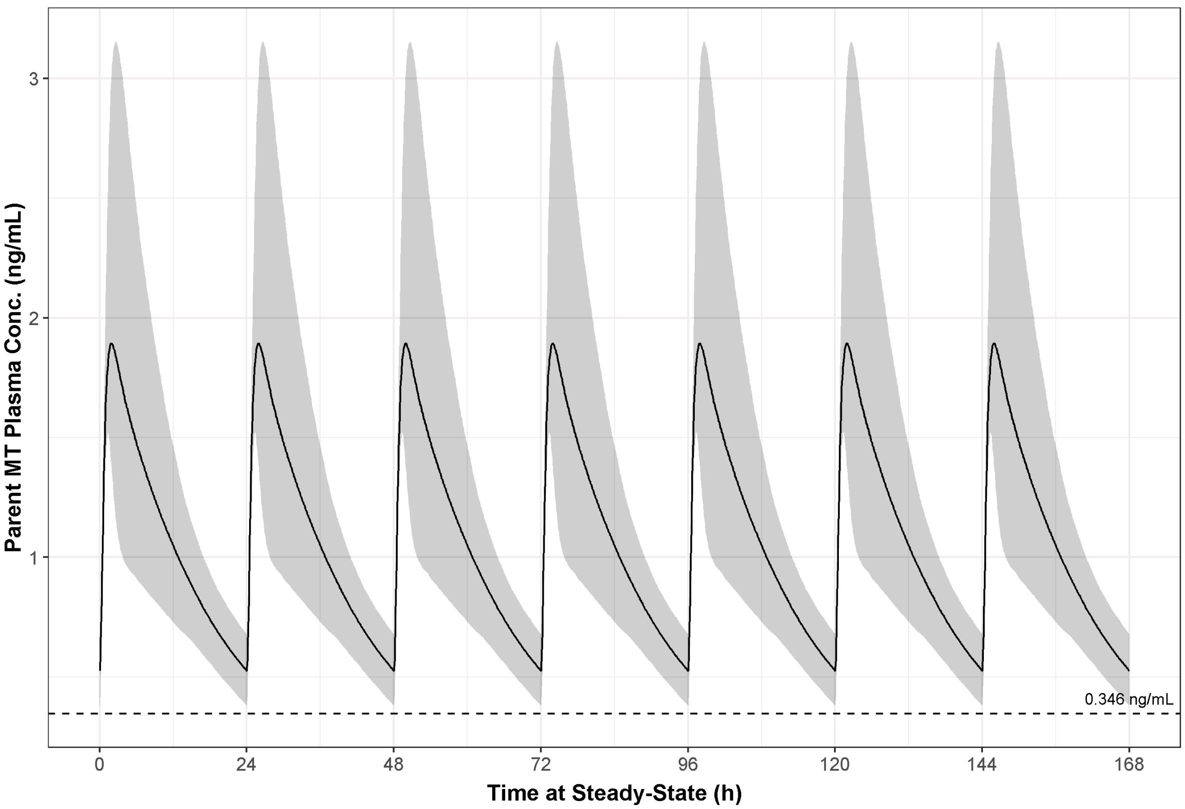

Supplementary Figure 1. Predicted parent MT concentration-time profiles for one week of dosing of 30 mg PO QD at steady state. Demographics and post-hoc PK parameters for the simulation were derived from the pooled AD/bvFTD Phase III population, truncated to the lower limit of CLcr in the bvFTD cohort (40 mL/min/1.73 m^2) (n = 1326). Solid, black line is the median predicted concentration; the grey shaded region is the 90% prediction interval. The horizontal, dashed line is the plasma concentration threshold of 0.346 ng/ml, required for clinical pharmacological activity in bvFTD.
