# Supplementary material for: Concentration-Dependent Activity of Hydromethylthionine on Clinical Decline and Brain Atrophy in a Randomized Controlled Trial in Behavioral Variant Frontotemporal Dementia
Source: J Alzheimers Dis. 2020 May 19;75(2):501–19. doi: 10.3233/JAD-191173 (PMC7306898; doi:10.3233/JAD-191173)
Supplement: Supplementary Material [file jad-75-jad191173-s002.pdf]

CLINICAL STUDY PROTOCOL

A Double-Blind, Placebo-Controlled, Randomized, Parallel Group, 12-Month Safety and Efficacy Trial of Leuco-methylthioninium bis(hydromethanesulfonate) in Subjects with Behavioral Variant Frontotemporal Dementia (bvFTD)

STUDY CODE: TRx-237-007

STUDY PHASE: 3

VERSION 8.0 DATED 7 JULY 2015

Previous Versions:

VERSION 7.0 DATED 29 JUNE 2015

VERSION 6.0 DATED 18 JUNE 2014

VERSION 5.1 DATED 15 MAY 2014

VERSION 5.0 DATED 20 NOVEMBER 2013

VERSION 4.1 DATED 9 SEPTEMBER 2013

VERSION 4.0 DATED 21 AUGUST 2013

VERSION 3.0 DATED 12 DECEMBER 2012

VERSION 2.0 DATED 20 SEPTEMBER 2012

VERSION 1.1 DATED 17 SEPTEMBER 2012

VERSION 1.0 DATED 17 MAY 2012

TauRx Therapeutics Ltd.
Liberty Building
Foresterhill Road
Aberdeen AB25 2ZP
Scotland, UK
Tel: +44 1224 438550
Fax: +44 1224 555173

TauRx Therapeutics Ltd.
3 Shenton Way, #21-04
Shenton House
Singapore 068805
Republic of Singapore

THIS PROTOCOL IS A CONFIDENTIAL DOCUMENT

This Protocol is the property of TauRx Therapeutics Limited. The information within it is confidential and is provided to you, for review by you, your staff, and applicable Ethics Committees/Institutional Review Boards. The Protocol must be kept in a confidential manner and must be returned to TauRx upon request. No part of this document may be reproduced in any form without permission from TauRx. By accepting this document, you agree that the information contained therein will not be disclosed to a third party without written authorization from TauRx.

1. PROTOCOL APPROVAL, RESPONSIBLE PERSONNEL, AND INVESTIGATOR SIGNATURES

1.1. Protocol Approval

Sponsor Signatory:

Claude Wischik, MD, PhD
Executive Chairman

TauRx Therapeutics Ltd
Liberty Building
Foresterhill
Aberdeen
AB25 2ZP
Telephone: + 44 1224 555191
Mobile: + 44 7779 114429

Claude Wischik 8/7/15

Signature Date

Pharmaceutical Physician:

Jiri Hardlund, MD
Chief Medical Officer
TauRx Therapeutics Ltd
Liberty Building
Foresterhill
Aberdeen
AB25 2ZP
Telephone: + 44 1224 438543
Mobile: + 44 7825 280632

[illegible] 08 JUL 2015

Signature Date

Statistician:

Charles S. Davis, PhD

CSD Biostatistics, Inc.
1005 W. Soft Wind Place
Tucson, AZ 85737
United States
Telephone: +1 520 544 6098
Mobile: +1 858 345 7250

Charles S. Davis 08 July 2015

Signature Date

1.2. Responsible Personnel

**TauRx Global Project Lead for TRx-237-007**<br>Gavin Ryan, PhD<br>Liberty Building<br>Foresterhill Road<br>Aberdeen AB25 2ZP<br>Scotland, UK<br>Tel: +44 1224 438548<br>Fax: +44 1224 555173<br>E-mail: g.ryan@taurx.com	**Global Lead Medical Monitor**<br>Manolo Beelke, MD, PhD<br>Worldwide Clinical Trials<br>Hausener Str. 7, 82131<br>Gauting, Germany<br>Tel: +49 (0) 89 89 05 8422<br>Fax: +49 (0) 89 89 05 8423<br>Mobile: +49 173 61 89 707<br>E-mail: manolo.beelke@wwctrials.com
**TauRx Head of Safety and Medical Monitoring**<br>Dr Jiri Hardlund<br>Liberty Building<br>Foresterhill Road<br>Aberdeen AB25 2ZP<br>Scotland, UK<br>Tel: +44 1224 438543<br>Fax: +44 1224 555173<br>Mobile: +44 7825 280632<br>E-mail: JHH@taurx.com	**Pharmacovigilance**<br>Nafisat Umar MSc, BSc<br>Senior Drug Safety Associate Global Drug Safety Operations<br>Worldwide Clinical Trials<br>Isaac Newton Centre, Nottingham Science Park<br>Nottingham, NG7 2RH, UK<br>Tel: +44 (0)115 951 7122<br>Fax: +44 (0)115 922 0960<br>Email: nafisat.umar@wwctrials.com
**Central Laboratory**<br>Covance, Inc.<br>*Americas*<br>8211 SciCor Drive<br>Indianapolis, IN 46214-2985<br>United States<br>Tel: +1 317 271 1200<br>Fax: +1 317 273 4030<br><br>*Europe*<br>7 rue Marcinhes<br>1217 Geneva<br>Meyrin Switzerland<br>Tel: +41 58 822 7000<br>Fax: +41 58 822 6999<br><br>*Asia and Australia*<br>1 International Business Park<br>#05-12A/B The Synergy<br>Singapore 609917<br>Tel: 65 6560 8793<br>Fax: 65 6565 5901	**Imaging**<br>*MRI Acquisition and Site Interaction*<br>BioClinica Inc.<br>100 Overlook Center<br>Princeton, NJ 08540<br>United States<br>Tel: +1 415-817-8945<br><br>*Oversight and Central Blinded Readers*<br>RadMD, LLC<br>4920 York Rd, Suite 2EE<br>Buckingham, PA 18912<br>Tel: +1 215 348 5644<br>Fax: +1 610 482 9332<br>E-mail: kshamsi@rad-md.net<br><br>*Analyses of MRI Data*<br>BioClinica, Inc.<br>100 Overlook Center<br>Princeton, NJ 08540<br>United States<br>Tel: +1 415-817-8945
**MT Concentrations**<br>University of Aberdeen GLP Test Facility<br>Meston Building<br>Old Aberdeen<br>Aberdeen AB24 3UE<br>United Kingdom<br>Tel: +44 (0) 1224 272945<br>Fax: +44 (0) 1224 272921	**Study Monitor and Project Management**<br>Worldwide Clinical Trials Limited<br>2nd Floor, 172 Tottenham Court Rd<br>London Q1T 7NS, UK<br>Tel: +44 20 7121 6160
**Data Management and Statistics**<br>SynteractHCR, Inc.<br>5759 Fleet Street, Suite 100<br>Carlsbad, CA 92008<br>United States<br>Tel: + 1 760 268 8200	**ECG**<br>BioClinica, Inc.<br>100 Overlook Center<br>Princeton, NJ 08540<br>United States<br>Tel: +1 301 795 2500

<table>
<tr><td>IWRS
BioClinica, Inc.
800 Adams Ave
Audubon, PA 19403
United States
Tel: +1 484 928 6736</td><td rowspan="2">Secondary Monitoring of Data and Documentation
Institute for Complex Systems and Mathematical Biology
University of Aberdeen
King’s College
Old Aberdeen AB24 3UE, UK

Exp-e-Data (UK) Limited
Wedale House
Church Wynd
Stow
Selkirkshire
TD1 2QU UK</td></tr>
<tr><td>Statistical Analysis of Pharmacokinetic Data
Institute for Clinical Pharmacodynamics
43 British American Blvd.
Latham, NY 12110
United States</td></tr>
</table>

1.3. Investigator Signature Sheet

By signing below, I agree to the conditions relating to this study as set out in this protocol (TRx-237-007 dated 7 July 2015).

I agree to conduct this study according to Good Clinical Practice (ICH GCP) and applicable regulatory requirements.

I fully understand that any changes instituted by me without previous discussion with TauRx or their designated representative constitute a violation of the protocol.

I agree to adhere to the protocol in all circumstances other than where necessary to protect the well-being of the subject.

I will ensure that the drugs supplied by TauRx will be used only for administration to subjects enrolled in this study and for no other purpose.

Study Site Principal Investigator’s Name, Title, Address and Contact Information:

____

____

____

____

____

____

Signature: ____ Date: ________________

SYNOPSIS

**Name of Sponsor / Company:**<br>**TauRx Therapeutics Ltd (TauRx)**	
**Name of Finished Product:**<br>**Leuco-methylthioninium bis(hydromethanesulfonate) (LMTM, TRx0237) Tablets, 100 mg**	
**Name of Active Ingredient:**<br>**Methylthioninium (MT)**	
**Number and Title of Study:** TRx-237-007: A Double-Blind, Placebo-Controlled, Randomized, Parallel Group, 12-Month Safety and Efficacy Trial of Leuco-methylthioninium bis(hydromethanesulfonate) in Subjects with Behavioral Variant Frontotemporal Dementia (bvFTD)	
**Study Site(s):** Approximately 55 - 80 study sites in the Americas, Europe, Asia, and Australia	
**Study Duration:**<br>The total duration of participation for an individual subject will be up to 62 weeks, including a Screening period of up to 42 days, a double-blind treatment study of 52 weeks, and a post-treatment assessment 28 days after completion of randomized treatment. It is anticipated that the study will have an overall duration of approximately 28 months, assuming an enrollment period of approximately 1 year.<br>In addition, a separate open-label extension study is planned.	**Phase of Development:**<br>Phase 3

Objectives:

Primary:

1. To demonstrate the efficacy of LMTM as assessed by the change from Baseline on:
   - Addenbrooke's Cognitive Examination-Revised (ACE-R)
   - Symptomatic effect as reflected by the Functional Activities Questionnaire (FAQ)
   - Disease-modifying effect based on reduction in decline in whole brain volume (WBV), using change from Baseline as measured by the Brain Boundary Shift Integral (BBSI) by MRI imaging

Secondary:

2. To evaluate the effect of LMTM as measured by the following additional global, disease severity, and motor impairment scales:
   - Modified Alzheimer's Disease Cooperative Study – Clinical Global Impression of Change (Modified ADCS-CGIC) – independently rated
   - Frontotemporal Dementia Rating Scale (FRS)
   - Unified Parkinson's Disease Rating Scale (UPDRS Parts II and III)
3. To evaluate the safety and tolerability of LMTM

Exploratory:

4. To evaluate the effect of LMTM as assessed by the change from Baseline on Addenbrooke's Cognitive Examination-III (ACE-III)
5. To evaluate an early effect on Modified ADCS-CGIC (after 8 weeks of treatment)
6. To determine the effects of LMTM on disease modification by reduction in the rate of atrophy in frontal and temporal lobes as well as ventricular volume as evaluated by MRI
7. To evaluate the effect of LMTM on the Mini-Mental Status Examination (MMSE)
8. To determine the effect of LMTM in subjects with known genetic mutations associated with bvFTD (mutations in the coding regions of Tau and TDP-43 genes)

Blood will be collected for purposes of population pharmacokinetic and genetic analyses (the latter analyses to be performed only for those subjects by or for whom separate legally acceptable informed consent is provided). The pharmacokinetic analysis will be detailed in a separate Statistical Analysis Plan (SAP) and will be reported separately (together with data from other studies).

Name of Sponsor / Company:
TauRx Therapeutics Ltd (TauRx)

Name of Finished Product:
Leuco-methylthioninium bis(hydromethanesulfonate) (LMTM, TRx0237) Tablets, 100 mg

Name of Active Ingredient:
Methylthioninium (MT)

Study Design:
Multinational, randomized, placebo-controlled, double-blind, parallel-group, 52-week, outpatient study with seven post-baseline on-treatment visits planned and an off-treatment follow-up visit for subjects who either discontinue early or complete treatment but do not enter a separate open-label extension study. Unscheduled visits and housing may occur as needed for assessment.

Number of Subjects:
The target recruitment number is approximately 180 subjects (90 per arm). Subjects who drop out after starting study treatment will not be replaced. Based on published data, the assumed decline and standard deviation of the ACE-R change from Baseline to Week 52 is 13.4 ± 13.8 units in untreated/placebo-treated subjects with bvFTD. Under this assumption, the study will have 90% power to detect a treatment difference of 6.7 units (or 50% reduction in assumed decline) on the ACE-R at a two-sided significance level of 0.05.

Subject Population:

Inclusion Criteria.

1. Diagnosis of probable bvFTD according to the International Consensus Criteria for bvFTD (Rascovsky *et al.*, 2007; Section 24.1)
2. Centrally rated frontotemporal atrophy score of 2 or greater, taken as the maximum of right or left frontal or anterior temporal lobes (Kipps *et al.*, 2007) on brain MRI of sufficient quality obtained at Screening or within a maximum of 42 days before Baseline, irrespective of pre-existing structural or functional imaging evidence supporting a diagnosis of bvFTD
3. MMSE ≥20 at the Screening visit
4. Age <80 years at the Screening visit
5. Modified Hachinski ischemic score of ≤4 at the Screening visit
6. Females must meet one of the following:
    - Surgically sterile (hysterectomy, bilateral salpingectomy / oophorectomy) for at least 6 months minimum
    - Have undergone bilateral tubal occlusion / ligation at least 6 months prior
    - Post-menopausal for at least 1 year
    - Using adequate contraception (a barrier method [such as condom, diaphragm, or cervical/vault cap] with spermicidal foam, gel, film, cream, or suppository; intrauterine device [IUD] or system, or oral or long-acting injected or implanted hormonal contraceptives for at least 3 months prior to Baseline; or vasectomized partner [with the appropriate post-vasectomy documentation of the absence of spermatozoa in the ejaculate]), or true abstinence (when this is in line with the preferred and usual lifestyle of the subject); subjects must be competent to use adequate contraception and to agree to continue to maintain adequate contraception throughout participation in the study
      OR
      In Italy, have avoided a pregnancy for at least 3 months prior to Baseline and accept to avoid a pregnancy throughout participation in the study
7. Subject and/or, in the case of reduced decision-making capacity, legally acceptable representative(s) consistent with national law is/are able to read, understand, and provide written informed consent in the designated language of the study site
8. Has one or more identified adult caregivers who meet the following criteria:
    - Either lives with the subject or sees the subject on average for ≥ 2 hours/day ≥ 3 days/week, or in the investigator’s opinion, the extent of contact is sufficient to provide meaningful assessment of changes in subject behavior and function over time and provide information on safety and tolerability
    - Is willing to provide written informed consent for his/her own participation

**Name of Sponsor / Company:**<br>**TauRx Therapeutics Ltd (TauRx)**
**Name of Finished Product:**<br>**Leuco-methylthioninium bis(hydromethanesulfonate) (LMTM, TRx0237) Tablets, 100 mg**
**Name of Active Ingredient:**<br>**Methylthioninium (MT)**

- Is able to read, understand, and speak the designated language at the study site
- Agrees to accompany the subject to each study visit
- Is able to verify daily compliance with study drug

9. If currently taking an AChEI (*i.e*., donepezil, galantamine, or rivastigmine) and/or memantine, at the time of Screening:
   - The subject must have been taking such medication(s) for ≥ 3 months
   - The current dosage regimen and dosage form must be within the locally approved dose range and must have remained stable for ≥ 6 weeks
   - It must be planned that the dosage regimen will remain stable throughout participation in the study

   Subjects not being treated with an AChEI or memantine (for ≥ 6 weeks before Screening) may also be enrolled if initiation of an AChEI or memantine is not planned for the time period during which the subject will be participating in this study
10. Able to comply with the study procedures in the view of the investigator

Exclusion Criteria.

1. Significant CNS disorder other than bvFTD, *e.g*., Alzheimer's disease, Lewy body dementia, Parkinson's disease, multiple sclerosis, progressive supranuclear palsy, hydrocephalus, Huntington's disease, any condition directly or indirectly caused by Transmissible Spongiform Encephalopathy (TSE), Creutzfeldt-Jakob Disease (CJD), variant Creutzfeldt-Jakob Disease (vCJD), or new variant Creutzfeldt-Jakob Disease (nvCJD)
2. Other significant intracranial pathology seen on brain MRI scan that would lead to a diagnosis other than probable bvFTD or that puts the subject at risk of Amyloid Related Imaging Abnormalities (ARIA), including:
   - Large confluent white matter hyperintense lesions (*i.e.*, Fazekas score of 3)
   - Other focal brain lesion(s) judged clinically relevant by the investigator
   - A single area of superficial siderosis
   - > 4 Cerebral microhemorrhages (regardless of their anatomical location or diagnostic characterization as "possible" or "definite")
   - Evidence of a prior macrohemorrhage
3. Biomarker evidence of underlying AD pathology as etiology of dementia
4. Expressive language deficits such that the subject is too severely impaired to allow testing at Baseline
5. Meets research criteria (El Escorial) for Amyotrophic Lateral Sclerosis or motor neuron disease (Section 24.2); evidence of mild motor neuron disease on examination is allowed if not expected in investigator's opinion to interfere with subject's completion of study but prominent bulbar symptoms, indicating high risk for respiratory compromise, would be exclusionary
6. Meets diagnostic criteria for probable bvFTD but has a proven mutation producing non-tau, non-TDP-43 pathology (*e.g*., FUS CHMP2B)
7. Clinical evidence or history of any of the following within specified period:
   - Cerebrovascular accident (2 years)
   - Transient ischemic attack (6 months)
   - Significant head injury with associated loss of consciousness, skull fracture or persisting cognitive impairment (2 years)
   - Other unexplained or recurrent loss of consciousness ≥ 15 minutes (2 years)

**Name of Sponsor / Company:**<br>**TauRx Therapeutics Ltd (TauRx)**
**Name of Finished Product:**<br>**Leuco-methylthioninium bis(hydromethanesulfonate) (LMTM, TRx0237) Tablets, 100 mg**
**Name of Active Ingredient:**<br>**Methylthioninium (MT)**

8. Epilepsy (a single prior seizure is considered acceptable)

9. Rapid eye movement sleep behavior disorder

10. DSM IV-TR criteria met (and not subsequently revised) for any of the following (within specified period):
    - Major depressive disorder (current)
    - Schizophrenia (lifetime)
    - Other psychotic disorders, bipolar disorder (within the past 5 years), or substance (including alcohol) related disorders (within the past 2 years)

11. Metal implants in the head (except dental), pacemaker, cochlear implants, or any other non-removable items that are contraindications to MR imaging; MR compatible prosthetics, clips, stents, or any other device proven to be compatible will be allowed

12. Resides in hospital or moderate to high dependency continuous care facility (residence in low grade assisted living facility where there is sufficient autonomy to permit evaluation of activities of behavior and general functioning is allowed so long as it is not mandated by an order issued either by the judicial or the administrative authorities)

13. History of swallowing difficulties (note: study drug should be swallowed whole and MUST NOT be broken, crushed, chewed, or dissolved in fluids prior to ingestion)

14. Pregnant or breastfeeding

15. Glucose-6-phosphate dehydrogenase (G6PD) deficiency

16. History of significant hematological abnormality or current acute or chronic clinically significant abnormality, including:
    - History of hereditary or acquired methemoglobinemia or Baseline measurement of MetHb > 2.0% (confirmed on repeat)
    - History of hemoglobinopathy, myelodysplastic syndrome, hemolytic anemia, or splenectomy
    - Screening hemoglobin value (confirmed upon repeat) below age/sex appropriate lower limit of the central laboratory normal range.

    Subjects in whom folate is < 4.0 ng/mL may be entered into the study provided folate supplementation (approximately 1 mg/day) is initiated and maintained for the duration of the study (see Section 4.7.8).

    Subjects in whom Vitamin B_{12} is < 150 pg/mL should be evaluated and supplemented as appropriate prior to the initiation of study drug (see Section 4.7.8). If review and correction can be achieved within the 42-day screening window, the subject may be entered into the study; otherwise the subject must be reconsented and rescreened after the deficit has been corrected.

17. Abnormal serum chemistry laboratory value at Screening deemed to be clinically relevant by the investigator (*e.g.*, those considered to have the potential to increase the risk associated with study participation or administration of investigational product and, in the judgment of the investigator, would make the subject inappropriate for entry into this study). In addition, subjects with either of the following abnormalities must be excluded:
    - Creatinine clearance < 50 mL/min at Screening, estimated by the central laboratory according to the Cockcroft and Gault equation
    - Thyroid stimulating hormone (TSH) above laboratory normal range (subject may be treated and rescreened after 3 months)

**Name of Sponsor / Company:**<br>**TauRx Therapeutics Ltd (TauRx)**
**Name of Finished Product:**<br>**Leuco-methylthioninium bis(hydromethanesulfonate) (LMTM, TRx0237) Tablets, 100 mg**
**Name of Active Ingredient:**<br>**Methylthioninium (MT)**

18. Clinically significant cardiovascular disease or abnormal assessments (in the opinion of the investigator) such as:
    - Hospitalization for acute coronary syndrome (acute myocardial infarction or unstable angina) or symptoms consistent with angina pectoris, within the 12 months preceding Baseline
    - Signs or symptoms of clinical heart failure within the 12 months preceding Baseline
    - Evidence of uncontrolled atrial fibrillation on Screening ECG or history of atrial fibrillation that is not currently controlled (heart rate ≥ 85 bpm and/or inappropriate anticoagulation) or where the QT interval cannot in the opinion of the investigator be assessed by triplicate ECGs taken within an approximate 2- to 5-minute interval (if better control of the heart rate and/or of anticoagulation can be achieved after adequate treatment, subject may be entered into the study if still within the 42-day window, or else the subject must be reconsented and rescreened). A cardiology consult should be sought for further ECG evaluation (especially in subjects with left bundle branch block) if deemed necessary by the investigator.
    - QTcF (based on mean of three triplicate ECGs, QT corrected for heart rate using Fridericia’s formula) at Screening > 460 msec in males or > 470 msec in females, or low or flat T waves making measurement of QT interval unreliable
    - Recent history of poorly controlled hypertension, systolic blood pressure > 160 mmHg, or diastolic blood pressure > 100 mmHg, after 5 minutes in a seated position at Screening
    - Hypotension: systolic blood pressure < 100 mmHg after 5 minutes in a seated position at Screening
    - Heart rate < 48 bpm or > 96 bpm by measurement of vital signs (after 5 minutes in a seated position) or by ECG at Screening

19. Preexisting or current signs or symptoms of respiratory failure (*e.g*., caused by chronic obstructive pulmonary disease, bronchial asthma, lung fibrosis, or other disease)
    - Subjects with previously diagnosed moderate to severe sleep apnea not adequately controlled (in the opinion of the investigator) should be excluded
20. Concurrent acute or chronic clinically significant (in the opinion of the investigator) immunologic, hepatic (such as presence of encephalopathy or ascites), or endocrine disease (not adequately treated) and/or other unstable or major disease other than bvFTD
    - Subjects with hepatitis or primary biliary cirrhosis should be excluded
    - Human T-Cell Lymphocytic Virus Type III (HTLV-III), Lymphadenopathy Associated Virus (LAV), any mutants or derivatives of HLTV-III or LAV, any condition associated with Acquired Immunodeficiency Syndrome or similar condition however named
21. Diagnosis of cancer within the past 2 years prior to Baseline (other than basal cell or squamous cell skin cancer or Stage 1 prostate cancer) unless treatment has resulted in complete freedom from disease for at least 2 years
22. Prior intolerance or hypersensitivity to MT-containing drug, similar organic dyes, or any of the excipients
23. Treatment currently or within 3 months before Baseline with any of the following medications (unless otherwise noted; see Section 4.7.4):
    - Tacrine
    - Amphetamine or dexamphetamine
    - Antipsychotics
        - Clozapine, olanzapine (and there is no intent to initiate therapy during the course of the study)
        - Other antipsychotics are allowable provided they have not been initiated within 3 months before Baseline and are used in a stable dose and regimen
    - Carbamazepine, primidone
    - Drugs for which there is a warning or precaution in the labeling about methemoglobinemia at approved

Name of Sponsor / Company:
TauRx Therapeutics Ltd (TauRx)

Name of Finished Product:
Leuco-methylthioninium bis(hydromethanesulfonate) (LMTM, TRx0237) Tablets, 100 mg

Name of Active Ingredient:
Methylthioninium (MT)

doses (*e.g*., dapsone, local anesthetics such as benzocaine used chronically, primaquine and related antimalarials)

24. Current or prior participation in a clinical trial as follows:
    - Clinical trial of a product for cognition prior to Screening in which the last dose was received within 90 days prior to Screening unless confirmed to have been randomized to placebo
    - A clinical trial of a drug, biologic, device, or medical food in which the last dose was received within 28 days prior to Baseline

Dose/Route/Regimen:
Subjects will be randomized 1:1 (stratified by geographical region, comprising three levels: the Americas, Europe, and Asia/Australia) to one of the following oral treatment groups to be given for 52 weeks:

- LMTM 200 mg/day group: LMTM 100 mg twice daily (*b.i.d.*) (one 100-mg tablet in the morning and one 100-mg tablet in the evening) [n=90]
- Placebo group: Placebo twice daily (*b.i.d.*) (one LMTM 4-mg tablet in the morning and one LMTM 4-mg tablet in the evening) [n=90]

The placebo group will receive low dose LMTM as a urinary and fecal colorant to maintain blinding. All tablets are of matching appearance.

Interruption of dosing for up to (maximum duration on any one occasion) 30 days during the first 6 months of treatment and/or 14 days during the second 6 months of treatment or reduction of dose by omitting the morning or evening dose may be allowed if the investigator determines this is indicated, *e.g*., tolerability concerns or laboratory abnormalities. In the event of continued poor tolerance subjects should be withdrawn from treatment (but encouraged to return for the balance of the scheduled study visits).

Methodology:
Following provision of written informed consent by the subject and/or his/her legal representative(s), consistent with national law, and his/her caregiver, eligibility for enrollment will be assessed initially at one or more Screening visits, which will occur no earlier than 42 days before the Baseline visit. MRI images acquired will be sent to an imaging core laboratory where they will be evaluated by a trained technologist for acceptable quality and then reviewed by an independent neuroradiologist (reader) who is trained in the evaluations and is not involved in the clinical conduct of the study to confirm eligibility of the subject. The reader's assessment will be communicated to the site within 5 business days of image transfer to the imaging core laboratory (and resolution of any quality issues).

All Baseline safety assessments will be completed by Baseline (Visit 2, Day 1) (designated pre-dose). Staging and baseline efficacy assessments can be made on the day before randomization and dosing (including ratings of ACE-R, additional items for the ACE-III, MMSE, Modified ADCS-CGIC, FRS, FAQ, and UPDRS Parts II and III). Subjects will be randomized and the first dose of study drug will be administered under supervised conditions (dosing may be held subject to eligibility review based on the local interpretation of triplicate ECGs by the investigator and on cardiology consultation if deemed necessary by the investigator). All subjects will be observed for at least 4 hours.

**Name of Sponsor / Company:**<br>**TauRx Therapeutics Ltd (TauRx)**
**Name of Finished Product:**<br>**Leuco-methylthioninium bis(hydromethanesulfonate) (LMTM, TRx0237) Tablets, 100 mg**
**Name of Active Ingredient:**<br>**Methylthioninium (MT)**

During this time, oral temperature (aural temperature is an acceptable, but less preferable, alternative) and respiratory rate will be measured hourly until discharge. Seated and standing blood pressure and pulse will be measured after approximately 2 hours, methemoglobin and oxygen saturation will be measured after approximately 2.5 hours, a triplicate ECG recording will be made after approximately 3 hours, and immediately thereafter, targeted physical and neurological examinations will be performed. Potential signs and symptoms of serotonin toxicity will be rated and potential cases of serotonin syndrome identified for all subjects (see Section 24.7); an assessment is to be completed just prior to discharge from the clinic. Blood will be collected pre-dose and approximately 3.5 hours post-dose for analysis of MT (restricted to sites that have a refrigerated centrifuge and appropriate freezer capacity). Subjects will be discharged from the clinic provided there are no signs or symptoms of impending serotonin toxicity; otherwise, they will continue to be monitored in the clinic and managed as medically appropriate. Upon discharge, all subjects will receive a supply of study drug to take home for use until the visit 8 weeks later (Visit 4); a compliance check will be made after 4 weeks (Visit 3).

Subjects who are receiving serotonergic medication and their caregivers are to remain in close proximity to a local hospital and remain in telephone contact with the clinic for at least 12 to 14 hours post-dose and reimbursement for accommodations will be made available if requested by the subject and/or his/her caregiver. They will be provided with monitoring instructions, a thermometer, and diary. Temperature should be monitored three times a day (in the morning, afternoon, and evening) until 72 hours after the first dose and recorded in the diary, to be returned at Visit 3 (Week 4). Caregivers will be contacted by telephone 5–7, >7–14, >14–24, 44–52, and 68–76 hours post-dose (with a minimum of 1 hour between contacts) and queried for the presence of signs and symptoms of serotonin toxicity in the subjects (see Section 24.8); they will be instructed to have the temperature diary available during these telephone contacts. If indicated, more frequent contacts will be made and the site will assume responsibility for clinical review and hospital referral if indicated.

Brain MRI will be performed at Screening/Baseline and evaluated for whole brain and ventricular volumes and atrophy in frontal and temporal lobes at Weeks 16, 32, and 52 (or upon early termination) to determine whether there is retardation of expected rate of atrophy over the period of the study. Change in whole brain and ventricular volumes as well as atrophy in frontal and temporal lobes will be quantified at the imaging core laboratory.

Subjects will be treated with study drug for 1 year (52 weeks) on an outpatient basis. On-treatment post-Baseline study visits will occur at time points approximately 4, 8, 16, 24, 32, 42, and 52 weeks after Baseline. Efficacy and safety assessments are described below. Caregivers will be contacted by telephone at approximately 12, 20, and 28 weeks. In the event of tolerability problems subjects and/or caregivers will be asked to contact the investigator and an extra visit will be arranged to assess the subject.

Subjects who complete the treatment period may be offered an opportunity to subsequently receive treatment with LMTM in a separate open-label extension study. Otherwise, a follow-up visit will occur approximately 28 days after the last dose of study drug for those subjects discontinuing early or not entering the open-label extension. The trial will be monitored for safety by a Data Safety Monitoring Board (DSMB) throughout its duration.

Name of Sponsor / Company:
TauRx Therapeutics Ltd (TauRx)

Name of Finished Product:
Leuco-methylthioninium bis(hydromethanesulfonate) (LMTM, TRx0237) Tablets, 100 mg

Name of Active Ingredient:
Methylthioninium (MT)

Assessments:

Efficacy/Imaging:

Efficacy will be evaluated after 4 months (16 weeks), 8 months (32 weeks), and 12 months (52 weeks) by assessors/raters who are not involved in the assessment of safety (with the exception of the C-SSRS, Serotonin Toxicity Assessment, and UPDRS) using the following instruments:

- ACE-R (incorporates the MMSE)
- FAQ
- ACE-III (in an exploratory fashion; this version does not incorporate the MMSE and includes 6 additional items)
- Modified ADCS-CGIC (also after 8 weeks in an exploratory fashion)
- FRS

Whole brain volume (MRI) will be evaluated centrally on images obtained after 4 months (16 weeks), 8 months (32 weeks), and 12 months (52 weeks) (or upon early termination).

If positron emission tomography (PET) is obtained as part of the site's usual practice, the data will be captured in the eCRF.

Safety and Tolerability:

Safety assessments will be performed during Screening to assess subject eligibility for enrollment. For enrolled subjects, safety assessments will be performed at Baseline (pre-dose and post-dose during the 4-hour observation), at 4, 8, 16, 24, 32, 42, and 52 weeks after Baseline, and approximately 4 weeks after the last dose of study drug (if applicable). Caregivers will be contacted by telephone at approximately 12, 20, and 28 weeks. All adverse events (AEs), vital signs, 12-lead ECGs, methemoglobin and oxygen saturation, clinical laboratory findings, physical and neurological examinations, serotonin toxicity, brain MRI data, and the potential for suicide or self-harm will be assessed according to the following:

- Adverse events will be recorded from the time informed consent is signed and recording will continue throughout the study, including the follow-up visit if applicable (Visit 10). Adverse events with an onset after the first dose of study drug or that worsen in intensity or treatment relationship after the first dose will be considered treatment-emergent.
- Temperature and respiratory rate will be recorded at Screening and at each clinic visit thereafter (or upon early termination), including the follow-up visit if applicable (Visit 10). Oral (sublingual) temperature measurements are preferable; aural temperature is an acceptable alternative (the use of this alternate means of measuring temperature is to be recorded in the eCRF). At Visit 2, temperature and respiratory rate will be recorded within 1 hour prior to dosing and hourly following the first dose while subjects are in the clinic (*i.e.*, for at least 4 hours). Upon discharge, if receiving a product with serotonergic potential, subjects/caregivers will continue to monitor temperature three times a day (in the morning, afternoon, and evening) until 72 hours after the first dose of study drug.
- Blood pressure and pulse will be obtained at Screening, within 1 hour before and approximately 2 hours after administration of the first dose of study drug (Visit 2), and at each visit thereafter (or upon early termination), including the follow-up visit if applicable (Visit 10). At Screening and on Day 1, two sets of blood pressure and pulse measurements will be made, the first set after the subject has been at rest in a seated position for approximately 5 minutes, and the second set approximately 2 minutes after the subject rises to a standing position. Otherwise, blood pressure and pulse will be measured with the subject in a seated position for at least 5 minutes.
- Height will be measured at Screening only. Body weight will be measured at Screening, Baseline (Visit 2; Pre-dose), and Visits 4 through 9 while on treatment (or upon early termination), and at the follow-up visit if applicable (Visit 10).

**Name of Sponsor / Company:**<br>**TauRx Therapeutics Ltd (TauRx)**
**Name of Finished Product:**<br>**Leuco-methylthioninium bis(hydromethanesulfonate) (LMTM, TRx0237) Tablets, 100 mg**
**Name of Active Ingredient:**<br>**Methylthioninium (MT)**
• A 12-lead ECG will be obtained in triplicate at Screening; the site may base eligibility determinations on the central interpretation or the results of the local interpretation in subjects with atrial fibrillation or with intraventricular conduction blocks. In subjects with atrial fibrillation and in subjects with intraventricular conduction blocks, a read should be undertaken locally and a local cardiology consult should be sought to decide on eligibility. Data derived from any triplicate ECG will be documented on the eCRF as the average of the three readings. At Visit 2, the ECG will be obtained in triplicate pre-dose and at approximately 3 hours after the first dose (timed to occur at the anticipated time of the peak MT concentration after initial dose); dosing decisions should be made on the basis of interpretations of the Screening ECG and local interpretations of the pre-dose ECG, with a cardiology consult if deemed necessary by the investigator, in particular in subjects with controlled atrial fibrillation (heart rate ≤ 84 bpm and appropriate anticoagulation) and in subjects with intraventricular conduction blocks. At all other visits (or upon early termination), the ECG measurement will be obtained as a single recording, unless there are emergent abnormalities deemed clinically significant by the Investigator, in which case triplicate ECGs should be obtained. For subjects with well controlled atrial fibrillation (heart rate ≤ 84 bpm and appropriate anticoagulation) at baseline, monitoring by triplicate ECGs at all subsequent visits is mandatory and data documented on the eCRF will be averages of the three readings.<br>• Methemoglobin and oxygen saturation will be measured using a pulse co-oximeter at Screening, within 1 hour before and approximately 2.5 hours after administration of the first dose of study drug (Visit 2), and at each visit thereafter (or upon early termination), including the follow-up visit if applicable (Visit 10).<br>• UPDRS Parts II and III will be evaluated at Baseline (Visit 2; pre-dose) and after 4 months (16 weeks), 8 months (32 weeks), and 12 months (52 weeks).<br>• Standard clinical laboratory testing, including hematology, blood chemistry, and urinalysis will be performed at Screening, Baseline (Visit 2; Pre-dose), Visits 3 through 9 while on treatment (or upon early termination), and at the follow-up visit if applicable (Visit 10). Testing for Vitamin B_{12} and folate will be included at each timepoint. TSH will be measured at Screening and Weeks 24 and 52. Clinical laboratory testing at the Screening visit will additionally include G6PD and haptoglobin. A serum pregnancy test will be collected for all women of childbearing potential at Screening, Baseline and at each visit beginning with Visit 3 (Week 4), including the post-treatment follow-up visit if applicable (Visit 10).<br>• Complete physical (PE) and neurological examinations will be performed at Screening. Targeted examinations will be performed to assess potential signs of serotonin toxicity and, as needed, to evaluate adverse events or change in medical history at pre-dose and approximately 3 hours after administration of the first dose of study drug (Visit 2) (following the ECG measurements); these are to be repeated as needed for subjects who remain in the clinic longer than 4 hours. Thereafter, targeted examinations are to be performed at each subsequent visit (or upon early termination), including the 4-week post-treatment follow-up if applicable (Visit 10).<br>• Signs and symptoms of potential serotonin toxicity will be rated in the clinic for all subjects at Baseline (Visit 2) and each subsequent visit (or upon early termination), including the follow-up visit if applicable (Visit 10). These will be rated using a 20-item Serotonin Toxicity (Syndrome) Diagnostic Interview and Rating Guide (Section 24.7). This will be conducted pre-dose and for at least 4 hours after the first dose of study drug while the subject is in the clinic (Visit 2); an assessment is to be completed just prior to discharge from the clinic. Caregivers of subjects receiving serotonergic medication will be contacted 5–7, >7–14, >14–24, 44–52, and 68–76 hours post-dose (with a minimum of 1 hour between contacts) and queried for the presence of signs and symptoms of serotonin toxicity using the Serotonin Toxicity Telephone Assessment (Section 24.8); if indicated, more frequent contacts will be made.<br>• The Columbia-Suicide Severity Rating Scale (C-SSRS) will be applied at Screening (Visit 1), after the first dose at Baseline (Visit 2; prior to discharge from the clinic) and at each visit thereafter (or upon early

**Name of Sponsor / Company:**<br>**TauRx Therapeutics Ltd (TauRx)**
**Name of Finished Product:**<br>**Leuco-methylthioninium bis(hydromethanesulfonate) (LMTM, TRx0237) Tablets, 100 mg**
**Name of Active Ingredient:**<br>**Methylthioninium (MT)**

termination), including the follow-up visit if applicable (Visit 10), to capture any respective changes.

- At Screening, medications administered within the last 3 months will be recorded and changes in concomitant medication will be recorded at all visits, including the follow-up visit if applicable (Visit 10) as well as at each telephone contact.
- Brain MRI will be performed at Screening/Baseline and evaluated by an independent neuroradiologist (reader) for confirmation of eligibility. Information about the eligibility will be communicated to the site by the imaging core laboratory within 5 business days of image transfer (and resolution of any imaging queries). Post-Baseline MRI scans will not be independently reviewed for ARIA at regular intervals; however, if ARIA is suspected and the site submits images to the imaging core laboratory for review, an independent review for ARIA may take place. All investigators should be aware of the imaging manifestations of ARIA and the clinical signs and symptoms. The study drug shall be permanently discontinued in any identified case of ARIA (guidance on follow up is provided).

Other Assessments:
Provided the site has a refrigerated centrifuge and adequate capability to reliably freeze samples, blood will also be collected at Baseline (Visit 2; pre-dose and approximately 3.5 hours post-dose) and, to the extent possible, at each subsequent on-treatment visit through Week 52 (approximately 20 minutes after the ECG recording) for purposes of population pharmacokinetic (PK) analysis of MT concentrations. The time of the prior dose and the time of the blood sample will be recorded.

A single blood sample for genotyping will be obtained only for subjects by or for whom legally acceptable informed consent for this is provided; the blood sample may be collected any time after eligibility for randomization and continued participation in the study has been confirmed.

Primary Statistical Analyses:
The primary efficacy analyses will be performed on a Modified Intent-to-Treat (MITT) population which will include all randomized subjects who took at least one dose of study drug and have a Baseline and at least one post-Baseline efficacy measurement. Subjects will be analyzed in the treatment group to which they were randomized.

- Two clinical co-primary efficacy endpoints will be analyzed to support a symptomatic / delay of disability claim: the ACE-R and the Functional Activities Questionnaire (FAQ).
- One clinical and one imaging efficacy endpoint will be analyzed to support a disease-modifying claim: the ACE-R and the whole brain volume (WBV).

The testing of the imaging and primary clinical outcomes will be conducted as follows:

Step 1: The ACE-R endpoint will be analyzed using a two-sided test at the alpha=0.05 level of significance.

Step 2: If the analysis of ACE-R is statistically significant, then the WBV and FAQ will be analyzed using the Bonferroni-Holm method. Thus, if the smallest of the two p-values is <0.025 (based on the use of a two-sided test), then the endpoint with the larger p-value will be tested at the alpha=0.05 level of significance (two-sided).

This approach will permit the ACE-R and WBV outcomes to support a disease-modifying claim in the event that the ACE-R and FAQ do not support a symptomatic / delay of disability claim or *vice versa*.

The primary analyses of the ACE-R and the FAQ will be carried out using restricted maximum likelihood based repeated measures linear mixed models with an unstructured covariance matrix in which no data will be imputed. Fixed effects will be included for treatment group (two levels), time (three levels, corresponding to Weeks 16, 32, and 52), the treatment group by time interaction, and geographic region (three levels consisting of the Americas, Europe, and Asia/Australia). In addition, the corresponding baseline quantity (ACE-R or FAQ) will be included as a covariate.

**Name of Sponsor / Company:**<br>**TauRx Therapeutics Ltd (TauRx)**
**Name of Finished Product:**<br>**Leuco-methylthioninium bis(hydromethanesulfonate) (LMTM, TRx0237) Tablets, 100 mg**
**Name of Active Ingredient:**<br>**Methylthioninium (MT)**

The primary comparisons will be based on the modeled change from Baseline at Week 52. Marginal means (least-square means in SAS) for change from Baseline at Week 52 will be presented for each treatment group.

Change in whole brain volume (WBV) as measured by the Brain Boundary Shift Integral (BBSI) will be evaluated to demonstrate a potential disease modification effect on an Imaging MITT population which will include all randomized subjects who took at least one dose of study drug and have a Baseline and at least one post-Baseline MRI imaging measurement of adequate imaging quality. The analysis will be carried out using a restricted maximum likelihood based repeated measures linear mixed model with an unstructured covariance matrix in which no data will be imputed. The dependent variable will be the change in WBV (as measured by the BBSI) from Baseline. The model will include fixed effects for treatment group (two levels), time (three levels, corresponding to Weeks 16, 32, and 52), and the treatment group by time interaction. The Baseline WBV will be included as a covariate. The primary comparison will be based on the modeled change from Baseline at Week 52. Marginal means (least-square means in SAS) for change from Baseline at Week 52 will be presented for each treatment group. The difference between these marginal means, with 95% confidence interval and p-value, will be provided for the LMTM treatment group *versus* placebo.

Sensitivity analyses, additional analyses of the primary variables (such as responder analyses and subgroup analyses), and the analyses of secondary and exploratory endpoints are described in the body of the protocol.

As an exploratory analysis, the association between the change in WBV and change in ACE-R at Week 52 will be investigated using Pearson's chi-squared test. In addition, the association between the change in WBV at Week 32 and change in ACE-R at Week 52 will also be explored using Pearson's chi-squared test to determine whether the former is predictive of the latter. The thresholds that separate the groups into decliners and non-decliners based on the change in ACE-R and the change in WBV is based on the corresponding mixed effects models as detailed in the statistical analysis section of this protocol.

TABLE OF CONTENTS

Page

1. PROTOCOL APPROVAL, RESPONSIBLE PERSONNEL, AND INVESTIGATOR SIGNATURES......2
1.1. PROTOCOL APPROVAL......2
1.2. RESPONSIBLE PERSONNEL......3
1.3. INVESTIGATOR SIGNATURE SHEET......5
SYNOPSIS......6
ABBREVIATIONS......22
2. BACKGROUND......27
2.1. INVESTIGATIONAL PRODUCT......27
2.2. NONCLINICAL DATA......28
2.3. CLINICAL DATA......29
2.3.1. Pharmacokinetics......29
2.3.2. Efficacy......30
2.3.3. Safety......31
2.4. RATIONALE FOR STUDY......33
3. OBJECTIVES......35
3.1. PRIMARY......35
3.2. SECONDARY......35
3.3. EXPLORATORY......35
4. STUDY DESIGN......36
4.1. GENERAL DESCRIPTION......36
4.2. POPULATION......37
4.3. DURATION......38
4.4. SCHEDULE OF ASSESSMENTS......38
4.5. RANDOMIZATION AND BLINDING......42
4.6. STUDY TREATMENT......43
4.7. CONCOMITANT MEDICATION......43
4.7.1. Dementia Medication......44
4.7.2. Drugs with Serotonergic Potential......45
4.7.3. CYP and P-gp Substrates......47
4.7.4. Drugs Used to Manage Behavioral Disturbance......47
4.7.5. Other Medications......48
4.7.6. Dietary Tyramine......48
4.7.7. Contraceptive Measures......48
4.7.8. Folate and Vitamin B_{12}......49

4.8. DATA AND SAFETY MONITORING BOARD / INDEPENDENT DATA MONITORING COMMITTEE 50

5. SELECTION OF SUBJECTS AND CRITERIA FOR WITHDRAWAL 50

5.1. INCLUSION CRITERIA 50

5.2. EXCLUSION CRITERIA 51

5.3. DISCONTINUATION/WITHDRAWAL 55

5.4. TERMINATION OF THE STUDY 56

6. TREATMENTS ADMINISTERED 56

6.1. STUDY DRUG 56

6.1.1. Active Ingredient 56

6.1.2. Inactive Ingredients 57

6.2. STUDY REGIMENS 57

6.2.1. Double-Blind Study Drug (LMTM or Placebo) 57

6.2.2. Dose Interruption/ Reduction/ Discontinuation 58

6.3. PACKAGING, LABELING, AND STORAGE 62

6.4. DISPENSING 63

6.5. COMPLIANCE 63

6.6. ACCOUNTABILITY 64

7. ASSESSMENT OF EFFICACY 64

7.1. RATERS 64

7.2. INSTRUMENTS 65

7.2.1. Addenbrooke's Cognitive Examination 66

7.2.2. Functional Activities Questionnaire (FAQ) 69

7.2.3. Modified Alzheimer's Disease Cooperative Study - Clinical Global Impression of Change (Modified ADCS-CGIC) 70

7.2.4. Frontotemporal Dementia Rating Scale (FRS) 71

8. ASSESSMENT OF SAFETY 71

8.1. ADVERSE EVENTS 71

8.1.1. Intensity 72

8.1.2. Relationship to Study Drug 72

8.1.3. Adverse Events of Special Interest 73

8.1.4. Serious Adverse Events 73

8.1.5. Pregnancy 74

8.1.6. Reporting Requirements and Timeframes 75

8.2. URGENT SAFETY MEASURES 77

8.3. CLINICAL LABORATORY TESTS 77

8.3.1. Serum Chemistry 78

8.3.2. Hematology 78

8.3.3. Urinalysis *79*
8.3.4. Other Laboratory Tests *79*
8.3.5. Review of Laboratory Results *80*
8.4. PULSE CO-OXIMETRY 80
8.5. VITAL SIGN MEASUREMENTS 81
8.5.1. Temperature and Respiratory Rate *81*
8.5.2. Blood Pressure and Pulse *82*
8.5.3. Weight and Height *82*
8.6. ELECTROCARDIOGRAPHY 82
8.7. PHYSICAL AND NEUROLOGICAL EXAMINATIONS 83
8.8. UNIFIED PARKINSON'S DISEASE RATING SCALE (UPDRS PARTS II AND III) 84
8.9. SEROTONIN TOXICITY (SYNDROME) 84
8.10. COLUMBIA-SUICIDE SEVERITY RATING SCALE (C-SSRS) 85
9. OTHER ASSESSMENTS 85
9.1. MAGNETIC RESONANCE IMAGING 85
9.1.1. Site Selection and Qualification *85*
9.1.2. Brain MRI Acquisition *86*
9.1.3. Image Transfer and Quality Assurance *87*
9.1.4. Image Evaluation Procedure *87*
9.1.5. Site Review *88*
9.2. MT CONCENTRATIONS 89
9.2.1. Procedure for Blood Sample Collection *89*
9.2.2. Packaging, Labeling, and Shipping of Blood Samples *89*
9.2.3. Analytical Laboratory *90*
9.3. GENOTYPING 90
10. STATISTICAL PLAN 90
10.1. EFFICACY ENDPOINTS 90
10.1.1. Primary Efficacy Endpoints *90*
10.1.2. Secondary Endpoints *91*
10.1.3. Exploratory Endpoints *91*
10.2. SAMPLE SIZE CALCULATIONS 91
10.2.1. ACE-R *91*
10.2.2. FAQ *91*
10.2.3. Modified ADCS-CGIC *92*
10.2.4. MRI *92*
10.3. STATISTICAL ANALYSIS 92
10.3.1. Analysis Populations *92*

10.3.2. Baseline Characteristics and Concomitant Medications 93
10.3.3. Dosing and Exposure 93
10.3.4. Efficacy Analyses 93
10.3.5. Safety Analysis 98
10.3.6. Concentration of MT 102
10.3.7. Genotyping 102
10.4. INTERIM ANALYSIS 102
11. REGULATORY 103
12. APPROVAL OF THE PROTOCOL AND AMENDMENTS 103
13. SERIOUS BREACHES 103
14. INFORMED CONSENT 104
15. INVESTIGATOR RESPONSIBILITIES 105
16. CONFIDENTIALITY AND DATA PROTECTION 106
17. QUALITY ASSURANCE AND CLINICAL MONITORING 106
18. DOCUMENTATION 107
19. PUBLICATION 108
20. INDEMNITY, INSURANCE AND COMPENSATION 109
21. ADMINISTRATIVE AND FINANCIAL AGREEMENT 109
22. STUDY ADMINISTRATION 109
23. REFERENCES 110
24. APPENDICES 114
24.1. INTERNATIONAL CONSENSUS CRITERIA FOR PROBABLE BVFTD (RASCOVSKY *ET AL*., 2007) 115
24.2. EL ESCORIAL RESEARCH CRITERIA FOR DIAGNOSIS OF AMYOTROPHIC LATERAL SCLEROSIS (BROOKS *ET AL*., 2000) 116
24.3. ASSESSMENTS BY VISIT 117
24.4. ADDENBROOKE'S COGNITIVE EXAMINATION-REVISED (ACE-R) 131
24.5. ADDENBROOKE'S COGNITIVE EXAMINATION-III (ACE-III) 138
24.6. MODIFIED ADCS-CGIC 144
24.7. SEROTONIN TOXICITY (SYNDROME) DIAGNOSTIC INTERVIEW AND RATING GUIDE (IN-CLINIC) 152
24.8. SEROTONIN TOXICITY TELEPHONE ASSESSMENT 160
24.9. SUMMARY OF CHANGES 161
24.9.1. Protocol Version 1.1 161
24.9.2. Protocol Version 2.0 162
24.9.3. Protocol Version 3.0 165
24.9.4. Protocol Version 4.0 168
24.9.5. Protocol Version 4.1 175
24.9.6. Protocol Version 5.0 176

24.9.7. Protocol Version 5.1 ... 177
24.9.8. Protocol Version 6.0 ... 179
24.9.9. Protocol Version 7.0 ... 180
24.9.10. Protocol Version 8.0 ... 182

ABBREVIATIONS

Abbreviation	Definition
ACE-III	Addenbrooke's Cognitive Examination-III
ACE-R	Addenbrooke's Cognitive Examination-Revised
AChEI	acetylcholinesterase inhibitor
AD	Alzheimer's disease
$ADAS\text{-}Cog_{11}$	Alzheimer's Disease Assessment Scale – cognitive subscale (11-item)
ADCS-CGIC	Alzheimer's Disease Cooperative Study – Clinical Global Impression of Change
AE	adverse event
AESI	adverse event of special interest
ALT	alanine transaminase
AMG	Arzneimittelgesetz (German Drug Law)
ANCOVA	analysis of covariance
ARIA	Amyloid Related Imaging Abnormality
AST	aspartate aminotransferase
ATC	Anatomical Therapeutic Classification
AUC	area under the curve
β-hCG	Beta subunit of human chorionic gonadotropin
BADLS	Bristol Activities of Daily Living Scale
BBSI	Brain Boundary Shift Integral
b.i.d.	twice daily
bpm	beats per minute
bvFTD	Behavioral variant frontotemporal dementia
ºC	degrees Celsius
C-CASA	Columbia Classification Algorithm of Suicide Assessment
CDR-SB-FTLD	Clinical Dementia Rating Scale-Sum of Boxes-Frontotemporal Lobar Degeneration
CFR	Code of Federal Regulations
CJD	Creutzfeldt-Jakob Disease
CK	creatine kinase
C_{max}	peak plasma concentration as observed
CMH	Cochran-Mantel-Haenszel
CNS	central nervous system
CPMP	Committee for Proprietary Medicinal Products
CRF	case report form
C-SSRS	Columbia-Suicide Severity Rating Scale
CT	computed tomography scan

Abbreviation	Definition
CV	curriculum vitae
CYP	Cytochrome P450
DICOM	Digital Imaging and Communications in Medicine
DNA	deoxyribonucleic acid
DSM-IV-TR	Diagnostic and Statistical Manual of Mental Disorders-4th Edition-Text Revision
DSMB	Data and Safety Monitoring Board
EC	Ethics Committee
ECG	electrocardiogram
eCRF	electronic case report form
eGFR	estimated glomerular filtration rate
EMA	European Medicines Agency
EOS	end of study
ET	early termination
EU	European Union
FAQ	Functional Activities Questionnaire
FD&C	Federal Food, Drug, and Cosmetic Act
FDA	Food and Drug Administration
FLAIR	fluid attenuated inversion recovery
FRS	Frontotemporal Dementia Rating Scale
FTLD	frontotemporal lobar degeneration
FTD	frontotemporal dementia
FTDC	International bvFTD Criteria Consortium
g, kg, mg	gram, kilogram, milligram
GCP	Good Clinical Practice
GEE	Generalized Estimating Equation
GGT	gamma-glutamyl transpeptidase
GI	gastrointestinal
GLP	Good Laboratory Practice
G6PD	glucose-6-phosphate dehydrogenase
HIPAA	Health Insurance Portability Accountabilty Act
HR	heart rate
HTLV-III	Human T-Cell Lymphocytic Virus Type III
ICF	informed consent form
ICH	International Conference on Harmonisation
IEC	Independent Ethics Committee

Abbreviation	Definition
IND	Investigational New Drug Application
IRB	Institutional Review Board
IRC	Independent Review Charter
IRIS	International Remote Imaging Systems, Inc.
ITT	Intent to treat
IUD	intrauterine device
IWRS/IVRS	interactive web/voice response system
L, mL	liter, milliliter
LAV	Lymphadenopathy Associated Virus
LDH	lactate dehydrogenase
LMT	leuco-methylthioninium
LMTB	leuco-methylthioninium-dihydrobromide
LMTM	leuco-methylthioninium bis(hydromethanesulfonate)
LOCF	last observation carried forward
LZCF	last z-score carried forward
MAO	monoamine oxidase
MAPT	microtubule-associated protein tau
MCH	mean corpuscular hemoglobin
MCHC	mean corpuscular hemoglobin concentration
MCV	mean cell volume
MDRD	Modification of Diet in Renal Disease
MedDRA	Medical Dictionary for Regulatory Activities
MetHb	methemoglobin
MHRA	Medicines and Healthcare products Regulatory Agency
min	minute(s)
MITT	Modified Intent-to-Treat
mmHg	millimeters of mercury
MMSE	Mini-Mental State Examination
MP RAGE	magnetization-prepared rapid acquisition with gradient echo
MRI	magnetic resonance imaging
msec	millisecond(s)
MT	methylthioninium
MTC	methylthioninium chloride
NHS	National Health Service
NPI	Neuropsychiatric Inventory

Abbreviation	Definition
nvCJD	new variant Creutzfeldt-Jakob Disease
PE	physical examination
PET	positron emission tomography
P-gp	P-glycoprotein
PGRN	progranulin
PhEur	European Pharmacopoeia
PHFs	paired helical filaments
PK	Pharmacokinetic
QA	quality assurance
QTcF	Fridericia's corrected QT interval
RBC	red blood cell
rpm	revolutions per minute
SAE	serious adverse event
SAP	statistical analysis plan
SD	standard deviation
SEM	standard error of the mean
SNRI	serotonin-norepinephrine reuptake inhibitor
SOC	system organ class
SOP	standard operating procedure
SPECT	single-photon emission computed tomography
SPGR	spoiled gradient echo
SSRI	selective serotonin reuptake inhibitor
SSS	symptom severity rating score
SUSAR	suspected unexpected serious adverse reaction
$T_{½}$	apparent first-order terminal elimination half-life
TEAE	treatment-emergent adverse event
T2*GRE	T2*-weighted gradient-echo imaging
t.i.d.	three times daily
T_{max}	time to peak plasma concentration
TSE	Transmissible Spongiform Encephalopathy
TSH	thyroid stimulating hormone
UCSF	University of California, San Francisco
UK	United Kingdom
UPDRS	Unified Parkinson's Disease Rating Scale
U.S.	United States

Abbreviation	Definition
USP	United States Pharmacopeia
VBSI	Ventricular Boundary Shift Integral
vCJD	variant Creutzfeldt-Jakob Disease
WBC	white blood cell
WBV	whole brain volume
WCT	Worldwide Clinical Trials
WHO	World Health Organization

2. BACKGROUND

Methylthioninium (MT) is proposed for treatment of Alzheimer's disease (AD) and other dementias that involve pathology of the microtubule associated protein tau, and with potential for benefit in other progressive neurodegenerative diseases characterized by pathological protein aggregation. Behavioral variant frontotemporal dementia (bvFTD) is one of the other dementias under investigation. It is a rare, progressive neurodegenerative disease characterized by progressive deterioration of behavior and language, associated with atrophy of the frontal and temporal lobes.

Onset of bvFTD typically occurs sometime in the 50s, though it can occur as early as age 20 or as late as age 80. Diagnosis of bvFTD is primarily on the basis of clinical signs and symptoms, informed by imaging of selected regions of the brain. Character change and disordered social conduct are the dominant features initially and throughout the disease course. Instrumental functions of perception, spatial skills, praxis and memory are intact or relatively well preserved.

There are currently no licensed treatments for any form of FTD. Though there are numerous published treatment studies in FTD, all of these are small and mainly of poor quality. There is some evidence to support both trazodone and dexamphetamine as having a beneficial effect on the behavioral symptoms of FTD; however, this evidence comes from very small studies and awaits replication. There are no studies that show benefits in cognition or function. Although no medications have been proven effective specifically in FTD, many clinicians look to the medications and treatment that alleviate the symptoms. Medical management includes treatment of concomitant medical conditions such as infections, parkinsonian symptoms, seizures (rare), and pain. Cholinergic drugs approved for the symptomatic treatment of AD are reported to worsen the condition. Disease progression in bvFTD is on average faster than AD and invariably leads to death (generally caused by pneumonia or other complications of severe debility, such as sepsis or pulmonary embolism). Survival from onset of symptoms is approximately 8 to 9 years.

Recent developments in molecular genetics and molecular neuropathology have confirmed that the diverse histopathological appearances can actually be understood at the molecular level to be based on deposition within various types of brain cells of aggregated forms of either Tau protein or TDP-43 protein. It is now generally agreed that 90% of FTLD syndromes are associated with pathological aggregations of either Tau protein or TDP-43 protein (Mackenzie *et al*., 2011). It is estimated that about half of bvFTD subjects have underlying pathology related to the protein Tau (Hodges *et al*., 2004; Shi *et al*., 2005), with approximately 10% having tau gene (MAPT) mutations (Rademakers *et al*., 2004). MT has been shown *in vitro* to prevent and dissolve neurotoxic Tau protein aggregates (Wischik *et al*., 1996; Harrington *et al*., 2015), to reduce Tau pathology in the brain in Tau transgenic mouse models (Melis *et al*., 2015), and to reduce TDP-43 aggregates in cell models (Yamashita *et al.,* 2009). LMTM therefore has the potential to be developed as a disease-modifying agent for bvFTD.

2.1. Investigational Product

The investigational product LMTM (also referred to as leuco-methylthioninium dihydromesylate and LMT.2MSOH, as well as by its code name, TRx0237) is a crystalline form of the reduced form of the active MT moiety. Chemically, the drug is named *N*,*N*,*N'*,*N'*-tetramethyl-10*H*-phenothiazine-3,7-diaminium bis(methanesulfonate). It is provided as a solid oral immediate-release dosage form. As a dihydromethanesulfonate salt (also known as mesylate), LMTM

stabilizes the reduced form of the MT moiety in the solid state; further, a matching placebo is provided that includes 4 mg LMTM as a urinary and fecal colorant.

The chloride salt of MT, methylthioninium chloride (MTC), is a diaminophenothiazine dye commonly known as methylene blue. It is the main ingredient in a number of proprietary preparations worldwide used at oral doses of up to 65 mg 3 times daily (*t.i.d.*) (50 mg MT *t.i.d.*) or as a component in various multi-ingredient formulations at doses up to 10 mg *t.i.d.* (8 mg MT *t.i.d.*). The most common use is as a urinary antiseptic in chronic urinary tract infection; other uses include treatment of subjects with manic-depressive psychosis, uncomplicated *Plasmodium falciparum* malaria, and urolithiasis. Methylthioninium chloride is approved in the European Union for parenteral administration in the treatment of drug-induced methemoglobinemia; MTC is usually given intravenously at 0.8 to 1.5 mg/kg body weight, administered over 5 minutes. It is also used parenterally in the management of ifosfamide encephalopathy and refractory shock syndromes of various causes, as a tracer in sentinel lymph node biopsy or visualization in parathyroid surgery, as well as a photosensitizing agent for the sterilization of blood products. While documented extensively in the published literature and various pharmacopeia (USP, PhEur), to TauRx's knowledge, all uses other than the intravenous treatment of methemoglobinemia remain experimental and have not been subject to regulatory review.

LMTM, LMTB (leuco-methylthioninium bis[hydrobromide], an earlier reduced salt form under investigation), and MTC all deliver the same active moiety, *i.e.*, MT, following dissociation of the counter ions. The absorption of the reduced leuco-MT (LMT) forms is favored at low pH such as in the stomach. When the MT moiety is dosed as MTC, it is presented in its charged or oxidized form (*i.e.*, MT^+). It is postulated that there is a requirement for an active reduction step at the low pH in the stomach to permit absorption of MT as the uncharged LMT form (which has one additional covalently bound hydrogen). Within cells, the active moiety MT is postulated to be present in an equilibrium between the reduced or oxidized forms, depending on the intracellular milieu, *i.e.*, pH and reductive capacity within that cell. As salts of MT have high solubility in aqueous media and deliver the same active moiety, the nonclinical and clinical experience with MTC and LMTB are relevant to LMTM. MTC and the reduced salts have been shown to result in comparable exposure when compared at equivalent MT doses in humans in the fasting state.

2.2. Nonclinical Data

In cell models, MTC, LMTM, and LMTB have been shown to prevent aggregation and enhance clearance of aggregation-dependent proteolytic products of tau. In transgenic mouse models, facilitation of clearance of tau aggregates and associated improvements in cognitive and motor learning abilities have been demonstrated. Tau pathology in these transgenic mouse models has been shown to be ameliorated in certain brain regions (*e.g.*, entorhinal cortex, hippocampus, and neocortex) following treatment with MTC, LMTB, and LMTM.

The primary toxicity of MT is hematological, manifesting as methemoglobinemia and a regenerative hemolytic anemia. Depending on the species, Heinz body formation also occurs. A no-observed-adverse effect-level has not been clearly established in any species other than rats (3 to 5 mg MT/kg/day regardless of salt or duration, depending on the low dose in a given study). Hematological toxicity was seen in the other species even at the lowest doses studied (0.9 mg MT/kg/day in monkeys and 3 mg MT/kg/day in minipigs). At higher doses, myeloid generation is affected and extramedullary hematopoeisis is seen; pigment (hemosiderin) deposition becomes

evident in liver and renal cortical tubules (some lipofuscin also present in minipigs) with no adverse effect on either liver or kidney function evident.

The other potential significant toxicities observed include effects on the heart, described as myocardial necrosis, generally occurring at rapidly lethal doses that exceed the maximum tolerated doses for chronic administration. In the minipig, no treatment-related cardiac toxicity has been observed until supra-lethal doses are achieved. Gastric irritation is seen in rodents and urinary bladder irritation is seen in mice and minipigs (with single cell necrosis in the latter species).

MT and some of its metabolites are genotoxic *in vitro* and damage DNA but are not genotoxic *in vivo.*

As reviewed by the U.S. National Toxicology Program, there is “some evidence of carcinogenic activity”[1] of MTC in male rats based on increased incidences of pancreatic islet cell adenoma and adenoma or carcinoma (combined). There is “some evidence of carcinogenic activity” in male mice based on increased incidences of carcinoma and of adenoma or carcinoma (combined) in the small intestine. The increased incidence of malignant lymphoma in 19 mg MT/kg/day males may be related to the administration of MTC whereas there is “equivocal evidence of carcinogenic activity” in female mice based on marginally increased incidences of malignant lymphoma.

MT has no effect on mating performance or pregnancy rate in Sprague-Dawley rats when administered orally. MT results in embryofetal toxicity when administered orally as evidenced by increased fetal resorptions in rats and (non-statistically) in rabbits; in rats the effects occur at doses that are maternally toxic. Following a single subcutaneous dose of 27 mg MT/kg and above, axial skeletal malformations are observed; neural tube defects, other fetal effects, and post-implantation losses occur at a threshold dose of 38 mg MT/kg.

In a standard study performed in male Long-Evans pigmented rats, MT does not cause phototoxicity.

Additional details regarding findings from nonclinical studies with various salt forms (LMTM, LMTB, and MTC) are described in the Investigator’s Brochure.

2.3. Clinical Data

2.3.1. Pharmacokinetics

To date, MT concentrations have been detected using an assay that includes heat- and acid-labile metabolites (conjugates).

LMTM, MTC, and LMTB have been shown to result in comparable exposure when given orally at equivalent MT doses in humans in the fasting state.

In humans, MT is rapidly absorbed when dosed in the fasted state. Median time of the peak plasma concentration (T_{max}) ranges from 1 to 2 hours post-dose when administered as single doses up to 300 mg; T_{max} occurs later at higher doses, up to an average of 2.8 hours post-dose. Peak plasma concentration (C_{max}) and area under the plasma concentration *versus* time curve

[1] Text in quotation marks refers to the interpretation of results provided by the U.S. National Toxicology Program, the sponsoring organization.

(AUC) increase linearly with dose, but in a less than proportional fashion at doses greater than 100 mg. Secondary peaks are evident on review of individual plasma *versus* concentration profiles. Based on published information and excretion of radiolabel in urine, absolute bioavailability is estimated to be 72%. Coadministration of the reduced leuco (LMT) form with food does not alter the extent of absorption significantly (peak concentrations are 8% lower); however, peak plasma concentrations are delayed by about 1.5 hours. Steady state is achieved within 3 days of dosing. The volume of distribution of total MT (*i.e.*, including acid- and heat-labile metabolites) administered orally approximates total body volume (mean 71 L). Apparent volume of distribution increases with increase in dose. MT is not highly protein bound (70 to 75%). Across studies, MT concentrations in whole blood samples are approximately half those in plasma. Study of MT metabolism in humans is ongoing; the primary metabolite appears to be a conjugate. Following a single dose, plasma concentrations decline with a mean elimination half-life ($T_{½}$) of 14 hours in younger healthy volunteers and 16 to 22 hours in healthy elderly volunteers. The $T_{½}$ of elimination of radiolabel from plasma ranges from 12.3 to 31.2 hours; elimination from whole blood is slower, ranging from 20.6 to 58.8 hours. An average of 95% of administered radioactivity is recovered over the 216 hours of urine and feces collection, approximately half within 24 hours of dosing. A total of 72% is recovered from the urine (42% within the first 24 hours) and 23% from the feces (5% within the first 24 hours). Renal clearance is less than glomerular filtration rate (GFR). Renal clearance of total radioactivity is 29 mL/min, approximately 71% of total clearance.

2.3.2. Efficacy

In a double-blind, placebo-controlled Phase 2 study (TRx-014-001) of male and female subjects with mild or moderate AD (acetylcholinesterase inhibitors [AChEIs] and memantine excluded), MTC was administered orally at doses of 30 mg *t.i.d.*, 60 mg *t.i.d.*, and 100 mg *t.i.d.* (total nominal doses of 69 mg/day, 138 mg/day, and 228 mg/day MT base equivalents, respectively). MT 138 mg/day appeared to slow the clinical rate of decline on the Alzheimer’s Disease Assessment Scale – cognitive subscale (ADAS-Cog_{11}) measured over 1 year, an effect that was already evident in patients with moderate disease at baseline within the first 6 months in an analysis of the whole population with severity as an interaction term. The 6-month analysis and methodology were prespecified as part of the primary analysis with the severity term as an interaction covariate. The result in patients with moderate disease severity at 6 months remained significant after correction for multiple comparisons. Longer term efficacy was confirmed in mixed mild/moderate subjects by *post hoc* analyses at 50 weeks and 102 weeks, and efficacy was also confirmed in analyses of a variety of secondary endpoints and was supported by imaging data at 24 weeks. The lower dose (69 mg/day) was below the minimally effective dose. The capsule formulation used limited the absorption of MT at the 228 mg/day dose due to a combination of dose-dependent delay in dissolution of the 100 mg MTC capsules used in the study and a formulation-independent limitation in the ability to absorb MT at the highest dose in the presence of food when administered as MTC. When both of these factors were taken into account, the clinically effective dose available in subjects receiving 100 mg MTC capsules either twice or three times daily was 72 – 109 mg/day, from nominal doses of 152-228 mg/day (Baddeley *et al*., 2015; Wischik *et al.*, 2015).

Building on this experience, a total of 7 subjects with a clinical diagnosis within the FTLD spectrum, 6 meeting Knopman *et al*. (2008) criteria for bvFTD, have thus far been treated with MTC at a dose of 138 mg/day MT and followed up 3 monthly for a period of up to 18 months.

Cognitive response to treatment was evaluated with the Addenbrooke's Cognitive Examination – Revised (ACE-R) (Mioshi *et al*., 2006) and Mini-Mental State Examination (MMSE). In order to quantify the treatment effect at 12 months, a non-linear conversion of MMSE to ACE-R was applied in 1 subject, based on independent data from 53 subjects provided by Kipps, and last observation carried forward (LOCF) imputation was used for missing data. ACE-R scores improved by a mean of 4.5 ± 2.4 (mean ± SEM) units at 12 months *versus* an expected decline of 13.4 ± 4.2 (mean ± SEM) units over this period (Kipps *et al*., 2008), implying a notional effect size relative to historically expected decline of 15.7 units on the 100-point ACE-R scale. The treatment benefit was sustained over 15 months. MMSE scores also remained improved at 12 months, mean 1.6 units above Baseline *versus* an expected decline of 2.5 ± 4.3 (mean ± SEM) units over the same period (Knopman *et al*., 2008). Outcomes on Bristol Activities of Daily Living Scale (BADLS) and Neuropsychiatric Inventory (NPI) were variable and no consistent treatment benefit was detected on these scales. The NPI has not been found to have useful trial metric properties (Knopman *et al*., 2008).

2.3.3. Safety

A total of 284 healthy volunteers have been studied in completed single and multiple dose studies of MT as of October 2014. These include single and multiple doses (expressed as MT base) of MTC 46 to 76 mg and 207 mg/day for 14 days, respectively; LMTB up to 800 mg as a single dose and up to 350 mg/day for 14 days; and LMTM up to 1000 mg as a single dose and up to 450 mg/day for 25.5 days. The most common adverse events (AEs) were gastrointestinal, renal and urinary disorders and headache.

A total of 321 subjects with AD participated in the 2-year Phase 2 study, TRx-014-001, of whom 307 were exposed to at least one dose of MTC. Diarrhea and related adverse events occurred in 48% at the highest dose, 228 mg MT/day, resulting in discontinuation of 16% overall. Nausea, vomiting, and retching were also common in MTC-treated subjects (8 to 14%), without a dose-response relationship; 3% of subjects discontinued due to vomiting. Dysuria and urgency were more common in MTC-treated subjects as compared to placebo, without a dose-response relationship (22 to 29% of subjects on MTC as compared to 13% of subjects on placebo). Urinary adverse events, including urinary discoloration and staining, resulted in the discontinuation of 6% of the subjects. Other common adverse events seen in ≥ 5% of MTC-treated subjects, overall and more common in MTC-treated subjects compared to placebo, were falls and related AEs (not correlated with change in blood pressure but correlated statistically with diarrhea), and rashes (none exfoliative).

Less common, but significant, adverse events that have been associated with MTC given parenterally include anaphylaxis and serotonin syndrome (the latter when MTC has been used intravenously in combination with products that increase serotonin). No cases have been reported to date for oral administration, including in the Phase 2 study where 17% of subjects received oral MTC in combination with products that increase serotonin.

As in animal studies, the primary laboratory abnormality is hematologic. Small, dose-dependent increases in methemoglobin are seen (mean overall increase was from 0.4% in controls to 0.8% in those treated with MTC). The highest value observed in the Phase 2 study has been 8%, not clinically significant; mean methemoglobin levels have remained below 3.5%, a conservative pre-specified threshold. Small reductions in hemoglobin and hematocrit values and compensatory increases in reticulocyte counts are seen, generally not clinically significant.

Possible liver function disturbance was evidenced by a single instance of jaundice and a single case of increase in gamma-glutamyl transpeptidase (GGT) and alkaline phosphatase, both of which were reported as possibly related.

In a completed Phase 1 study with LMTM (TRx-237-011), several subjects had protein and blood in the urine shown by dipstick analysis, not correlated with the reports of dysuria. Colorimetric interference by MT on the dipstick tests used confounds interpretation of these results; nonetheless, in some male subjects the presence of blood in the urine was confirmed by microscopy. (Urinalysis was not included in the Phase 2 trial.)

MT has not been associated with changes in vital signs or electrocardiogram (ECG) intervals.

MTC has been shown to be an inhibitor of monoamine oxidase (MAO), with greater potency *in vitro* (*i.e.*, in the oxidized MT^+ form) for the A isoform than the B isoform. The Medicines and Healthcare products Regulatory Agency (MHRA) in the UK, Health Canada, and, most recently, the US Food and Drug Administration (FDA) have issued safety alerts concluding that the concomitant administration of a serotonergic psychiatric medication with intravenous administration of MTC has the potential for a drug interaction causing serotonin syndrome. These are based on spontaneously reported serious adverse events as well as published cases, when MTC was used in intravenous doses of 1 to 8 mg/kg mostly for gland visualization in parathyroidectomy. As a result, MTC should generally not be given intravenously to subjects taking serotonergic drugs unless the benefit is deemed to outweigh the risk (FDA advice) or subjects are observed for at least 4 hours after the MTC dose (MHRA advice). TauRx is aware of only one[2] reported case of serotonin syndrome with MTC administered non-parenterally.

Of the 1713 subjects with AD or bvFTD randomized to receive LMTM (or matching placebo) in the ongoing double-blind Phase 3 studies as of 30 September 2014[3], 383 subjects (22.3%) were concomitantly receiving a selective serotonin reuptake inhibitor (SSRI) and 540 subjects (31.5%) were concomitantly receiving drugs with serotonergic potential. With extensive monitoring implemented in the ongoing double-blind Phase 3 studies using the Serotonin Toxicity (Syndrome) Diagnostic Interview and Rating Guide, there have been 2 cases of possible serotonin toxicity reported as AEs (as of 14 October 2014) based on the Serotonin Toxicity Diagnostic Interview score. Both were mild in intensity and resolved with treatment interruption; the symptoms did not reappear with restarting study drug. Neither subject reported concomitant use of a serotonergic medication or substance. For one of these cases, after 23 days of follow-up, the Investigator concluded that the case did not represent true clinical serotonin toxicity. Although 17% of subjects exposed to MTC in the 2-year Phase 2 study, TRx-014-001, were taking SSRIs or venlafaxine (a serotonin-norepinephrine reuptake inhibitor, SNRI) concomitantly at some point during the study, there was no report of an episode of serotonin syndrome. This may reflect the difference in predominant form of MT (*i.e.*, LMT rather than MT^+) in circulation following oral *versus* intravenous administration. The risk with oral LMTM is unknown. Nonetheless, there is a theoretical risk of increased serotonin levels following administration of LMTM or following coadministration of LMTM with a serotonergic medication. Close monitoring will continue in the ongoing double-blind Phase 3 studies.

---

[2] Izdes *et al*., 2014 (administration as a solution *via* nasogastric tube).
[3] Data available at the time of the Developmental Safety Update Report #3 cut-off date of 14 October 2014.

In addition to warning subjects and their caregivers of this risk, they are also to be informed that based on this pharmacological profile there is a theoretical potential for a tyramine reaction; TauRx is not aware of any reports of diet-associated hypertensive crisis.

Additional details regarding clinical findings from studies with LMTM, LMTB, and MTC are described in the Investigator's Brochure.

2.4. Rationale for Study

As described above, nonclinical and clinical evidence supports the further study of LMTM in subjects with bvFTD. Subjects will be diagnosed with criteria revised by the International bvFTD Criteria Consortium (FTDC, led by Dr. Rascovsky from the UCSF Memory and Aging Center; Rascovsky *et al*., 2007). These have been developed based on accumulated experience with established criteria that have been the standard for over a decade, namely those published by Neary and colleagues (Neary *et al.*, 1998). The revised criteria have been shown in a validation process to have better sensitivity and the required specificity to differentiate from other dementias (Rascovsky *et al.*, 2011).

bvFTD is a clinical diagnosis based on symptom profile and evidence of progression on caregiver report or functional assessment. Imaging evidence of neurodegeneration, either structural or functional, is required for a diagnosis of probable bvFTD. This ensures that clinically similar, yet non-progressive subjects, also known as 'bvFTD phenocopies' are excluded from a diagnosis of 'probable bvFTD'. It has been shown that subjects with a clinical diagnosis of bvFTD who do not have visible atrophy on imaging have a prolonged, much more stable course. Those with scans rated on a visual rating scale (Kipps *et al*., 2008) declined at a more rapid rate. A centrally rated visual atrophy score of 2 or greater in at least one of the following areas (left or right anterior temporal, left or right frontal lobes) ensures that subjects expected to decline sufficiently on the outcome measure (ACE-R) are included.

In this study, change in whole brain volume (WBV) as measured by the Brain Boundary Shift Integral (BBSI; Fox and Freeborough, 1997) will be used as an imaging outcome to support a disease-modifying effect of treatment with LMTM by showing a reduction in the overall rate of brain atrophy at 52 weeks. In addition, exploratory analyses will be conducted to further support an effect on reduction of brain atrophy by analysis of change in ventricular volume using the Ventricular Boundary Shift Integral (VBSI) as well as change in the rate of atrophy in frontal and temporal lobes.

Previous studies (Kipps *et al*., 2008; Knopman *et al*., 2008) found over 87% of bvFTD patients have an MMSE at baseline of 21 points or above with a mean MMSE ranging from 24.2 (SD 4.8) and 25.1 (SD 4.3). A baseline MMSE of ≥20 would be expected to include a majority of subjects capable of managing the trial assessments. Subjects must be less than 80 years of age at Screening to be eligible for enrollment in this study. Given that onset is typically in the 50's and survival is approximately 8 to 9 years, the diagnosis of bvFTD would be questionable in someone presenting with an age over 80 years.

As an exploratory objective, this study will include a genotyping evaluation to determine the effect of LMTM in subjects with known genetic mutations associated with bvFTD (mutations in the coding regions of Tau and TDP-43 genes). The true prevalence of genetic forms of frontotemporal dementia is unknown and methodologically the question is difficult to answer. There are at least seven different gene mutations that can cause FTD (Mackenzie *et al.*, 2010) but

mutations in the tau and progranulin (PGRN) genes constitute the vast majority and the other mutations are rare. Only a minority of FTD subjects has a known mutation and the common mutations result in either aggregated tau or TDP-43 protein.

The overall design (placebo-controlled, parallel group), duration of treatment (12 months), and primary endpoints (ACE-R as an objective endpoint and the FAQ indicative of functional clinical benefit) are considered appropriate for Phase 3 evaluation. With respect to the endpoints, the scales chosen address the key characteristics of bvFTD, namely cognitive and functional impairment. Although motor impairment is seen in only a minority of early sporadic cases, a motor endpoint is also included, primarily as a safety parameter. The efficacy evaluations are further discussed in Section 7.2.

The dose level (200 mg/day, expressed as MT base equivalents) and dosage regimen (twice daily) of orally administered LMTM were selected based on available nonclinical and clinical data for LMTM, LMTB, and MTC. In the Phase 2 study of MTC (see Section 2.3), potential efficacy in the treatment of subjects with AD was consistently associated with the oral dose of 138 mg/day, expressed as MT base equivalent, and a higher dose, 228 mg/day, expressed as MT base equivalent, was tolerated. There is also preliminary evidence in a small case series that the 138 mg/day (given as 48 mg/MT *t.i.d.*) dose is effective in subjects with bvFTD. Based on clinical pharmacokinetic data, the dose of 200 mg/day administered as two divided doses of LMTM is expected to produce a steady-state plasma level similar to that achieved at the highest dose of MTC studied to date, 228 mg/day, administered as three divided doses of MTC. The twice daily regimen is consistent with the approximate 16- to 22-hour plasma elimination half-life observed in healthy elderly volunteers following single doses of LMTB.

A small amount of LMTM (4 mg) has been included in the placebo tablets, sufficient to color urine and feces, thereby maintaining the study blind. The low 8 mg/day dose of LMTM to be administered to subjects in the placebo group in this study is believed to be unlikely to result in efficacy and in Phase 1 trials has not been associated with adverse events.

Anticipated risks of participation in this study include the potential of experiencing AEs similar to those observed in a previously-conducted Phase 2 study with MTC in subjects with mild or moderate Alzheimer’s disease (see Section 2.3). Standard safety assessments are included at frequent intervals. Subjects will be monitored for any signs or symptoms of possible methemoglobin increases and monitoring guidelines for dosing are provided; pulse co-oximetry will be used to avoid the potential interference by other colorimetric approaches. ECGs were not obtained in the Phase 2 study with MTC and a study of the potential for MT to alter the QT interval has recently completed; however, data analysis is still ongoing. Therefore, ECGs will continue to be monitored in the study (including approximately 3 hours after the initial dose which is to be administered in the clinic). An independent Data and Safety Monitoring Board will be established (see Section 4.8).

Concomitant medications are allowed consistent with the subject population provided there are no safety grounds for exclusion. AChEI and memantine use are allowed; to avoid interference with the primary study objectives, the dose and regimen must have been stable as described in Section 4.7.1. Subjects with bvFTD are also often treated with antidepressant medication; however, because MT is a MAO A and, to a lesser extent MAO B, inhibitor *in vitro*, drugs with serotonergic potential should be used with caution due to the theoretical risk of serotonin

syndrome (see Section 4.7.2) based on intravenous usage. The risk with oral LMTM is unknown. Subjects will be allowed to enter the study on such medication and will be closely monitored, including in the clinic for at least 4 hours after the first dose. They and their caregiver(s) are to remain in close proximity to a local hospital and remain in telephone contact with the clinic for at least 12 – 14 hours after the first dose and reimbursement for accommodations will be made available if requested by subjects and/or their caregivers. They will be instructed in the signs and symptoms of serotonin toxicity and given a thermometer and instructed in its use and diary recording. In the intervening time before the next clinic visit, caregivers will be contacted by telephone 5–7, >7–14, >14–24, 44–52, and 68–76 hours post-dose (with a minimum of 1 hour between contacts) and queried for any signs or symptoms of serotonin toxicity in the subject.

Because MT has activity on the central nervous system (CNS), subjects will also be evaluated for suicidal ideation and intent using the Columbia-Suicide Severity Rating Scale (C-SSRS). This is consistent with (U.S.) regulatory expectations for CNS-active medications. While not validated for use in this subject population, it has been used and provides a systematic means of collecting data in dementing syndromes for future potential regulatory use.

3. OBJECTIVES

The primary, secondary and exploratory objectives of this double-blind, placebo-controlled, randomized, parallel-group, 12-month study of LMTM (leuco-methylthioninium bis(hydromethanesulfonate) [TRx0237, LMTM] 200 mg/day) in subjects with probable bvFTD are stated below.

3.1. Primary

1. To demonstrate the efficacy of LMTM as assessed by the change from Baseline on:
    - Addenbrooke's Cognitive Examination-Revised (ACE-R)
    - Symptomatic effect as reflected by the Functional Activities Questionnaire (FAQ)
    - Disease-modifying effect based on reduction in decline in whole brain volume (WBV), using change from Baseline as measured by the Brain Boundary Shift Integral (BBSI) by MRI imaging

3.2. Secondary

2. To evaluate the effect of LMTM as measured by the following additional global, disease severity, and motor impairment scales:
    - Modified Alzheimer's Disease Cooperative Study – Clinical Global Impression of Change (Modified ADCS-CGIC) – independently rated
    - Frontotemporal Dementia Rating Scale (FRS)
    - Unified Parkinson's Disease Rating Scale (UPDRS Parts II and III)
3. To evaluate the safety and tolerability of LMTM

3.3. Exploratory

4. To evaluate the effect of LMTM as assessed by the change from Baseline on ACE-III
5. To evaluate an early effect on Modified ADCS-CGIC (after 8 weeks of treatment)

6. To determine the effects of LMTM on disease modification by reduction in the rate of atrophy in frontal and temporal lobes as well as ventricular volume as evaluated by MRI
7. To evaluate the effect of LMTM on the MMSE
8. To determine the effect of LMTM in subjects with known genetic mutations associated with bvFTD (mutations in the coding regions of Tau and TDP-43 genes)

Blood will be collected for purposes of population pharmacokinetic and genetic analyses (the latter analyses to be performed only for those subjects by or for whom separate legally acceptable informed consent is provided). The pharmacokinetic analysis will be detailed in a separate Statistical Analysis Plan (SAP) and reported separately (together with data from other studies).

4. STUDY DESIGN

4.1. General Description

This study is a multinational, double-blind, randomized, placebo-controlled, 12-month study in subjects who meet International Consensus Criteria for probable bvFTD (Rascovsky *et al.*, 2007). Eligibility for enrollment will be assessed initially at a Screening visit, which is to occur within 42 days of Baseline. Independent verification that the Screening MRI is consistent with subject eligibility must be obtained prior to randomization. One hundred eighty (180) male and female subjects will participate (see Section 4.2 for further description of the population and Section 5 for inclusion and exclusion criteria).

Eligible subjects will be randomized in a 1:1 ratio at the Baseline visit to one of the following two treatment groups: the LMTM 200 mg/day group or the placebo group. The randomization will be stratified by region. Randomization is further described in Section 4.5.

The first dose will be administered in the clinic, with subjects remaining for at least 4 hours (for safety observation, vital signs, physical and neurological examination, pulse co-oximetry, and ECG). Thereafter, dosing will be on an outpatient basis and subjects will receive study drug twice daily (*b.i.d.*) for 12 months (LMTM 100 mg *b.i.d.* or placebo *b.i.d.*). The placebo tablets include LMTM 4 mg as a urinary and fecal colorant to maintain blinding (see Section 6.1 and Section 6.2.1); hence, the placebo regimen contains LMTM 8 mg/day.

During the treatment period, interruption of dosing for up to (maximum duration on any one occasion) 30 days during the first 6 months of treatment and/or 14 days during the second 6 months of treatment or reduction of dose will be allowed if indicated (*e.g.*, tolerability concerns or clinical laboratory abnormalities) (see Section 6.2.2). Adverse events requiring discontinuation are also detailed in Section 6.2.2. In the event of early withdrawal, subjects will be encouraged to continue to attend visits in accordance with the study visit schedule.

Study visits during the treatment period will occur at time points approximately 4, 8, 16, 24, 32, 42, and 52 weeks after Baseline. Assessments to be made at these visits are described in Section 4.4 and Table 4-1. During intervening times, caregivers will be contacted by telephone at approximately 12, 20, and 28 weeks. Additional telephone contacts will occur in subjects entering the study on serotonergic medication. In addition to in-clinic observation, caregivers

will be contacted by telephone 5-7, >7-14, >14-24, 44-52, and 68-76 hours after the first dose of study drug (with a minimum of 1 hour between contacts).

Subjects who complete treatment through to the final Week 52 visit may be offered an opportunity to subsequently receive treatment with LMTM in an open-label extension study (separate protocol). For subjects who do not continue treatment in the open-label extension study, a follow-up visit will occur approximately 28 days after the last dose of study drug.

Assessments to be performed at each visit are described in Section 4.4. Efficacy and safety assessments will be performed at Baseline and at designated visits throughout the treatment period. Separate efficacy and safety raters will be used to maintain blinding; further, the Modified ADCS-CGIC will be rated by a completely independent rater (see further detail in Section 7, Section 8, and Section 9 for efficacy, safety, and imaging and other assessments, respectively).

A schematic representation of the study design is provided in Figure 4-1.

Figure 4-1 Schematic Representation of Study Design

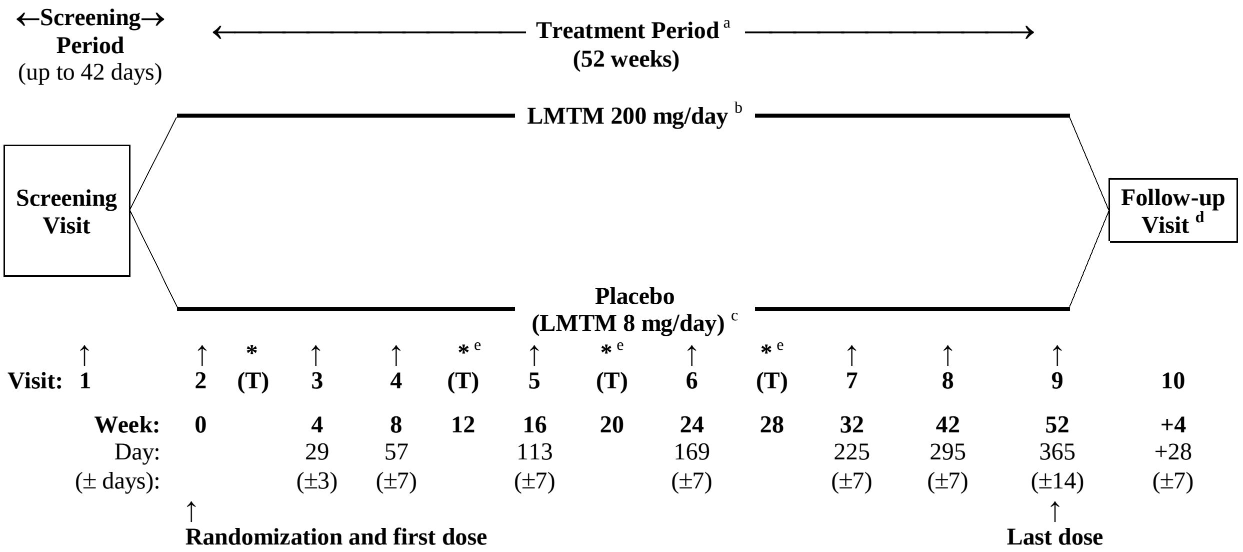

[a] During the treatment period, study drug will be administered under double-blind conditions. All subjects will receive one tablet twice each day (*b.i.d.*; one tablet each morning and one tablet each evening), unless the dose is held or reduced.
[b] LMTM 200 mg/day group subjects will receive an LMTM total daily dose of 200 mg, administered as one LMTM 100-mg tablet *b.i.d.*
[c] Placebo group subjects will receive an LMTM total daily dose of 8 mg, administered as one LMTM 4-mg tablet *b.i.d.*; this low dose is included to maintain blinding because LMTM is associated with coloration of urine and potentially feces.
[d] Subjects who complete treatment through the final Week 52 visit may be offered an opportunity to take part in the open-label extension study. Otherwise, a safety follow-up visit will occur approximately 28 days after the last dose of study drug.
[e] Subjects will be contacted by telephone (*) (T) at selected intervals between clinic visits; the frequent contacts to be made over the first 3 days after the first dose apply only to subjects receiving drugs with serotonergic potential.

4.2. Population

Subjects enrolled into this study will have a diagnosis of probable bvFTD according to International Consensus Criteria (Rascovsky *et al.*, 2007; Section 24.1), including behavioral decline with or without progressive cognitive decline and independently confirmed imaging

evidence of frontotemporal atrophy (see Inclusion Criteria No. 1 and 2 in Section 5.1). Subjects are to have a mini-mental state examination (MMSE) score ≥ 20 at the Screening visit. The subjects will not have any other primary neurodegenerative disorder other than bvFTD.

A total of 180 male and female subjects are planned to be enrolled (90 subjects per arm). Presuming a withdrawal rate of approximately 20%, it is anticipated that approximately 144 subjects will complete the study (72 subjects in LMTM and 72 subjects in the placebo group, assuming unbiased withdrawal). Subjects who drop out after starting study treatment will not be replaced. The basis for this sample size is further discussed in Section 10.2.

The study will be conducted at approximately 55 to 80 study sites, each anticipated to enroll 10 or fewer subjects. The study will be multinational and will include study sites in the Americas, Europe, Asia, and Australia.

4.3. Duration

The total duration of participation in this study for an individual subject is planned to be up to approximately 62 weeks, including a Screening period of up to 42 days, a double-blind treatment period of 52 weeks, and a post-treatment assessment approximately 28 days after completion of randomized treatment for subjects who either discontinue early or complete treatment but do not enter a separate open-label extension study. Additional follow-up visits may be scheduled as needed to monitor the resolution or stabilization of adverse effects.

It is anticipated that the study will have an overall duration of approximately 28 months, assuming an enrollment period of 1 year. The current study will be concluded after the last visit for the last subject under this protocol.

4.4. Schedule of Assessments

Subjects will be screened for eligibility on the basis of diagnostic evaluations, mental state assessments, and safety assessments (clinical laboratory testing, vital sign and pulse co-oximetry, complete physical and neurological examinations, and ECG). An MRI of the brain will be performed at Screening or within a maximum of 42 days before Baseline (results of sufficient quality must be available at the Baseline visit). If positron emission tomography (PET) is obtained as a part of the site's usual practice, the data will be captured in the eCRF (*i.e.*, both existence of a prior diagnostic PET scan and a text field into which the prior diagnostic radiological findings are transcribed).

The ACE-R (which incorporates the MMSE), Modified ADCS-CGIC, FRS, FAQ, and UPDRS (Parts II and III) will be rated at Baseline for enrolled subjects. At the completion of the ACE-R, additional items will be rated to allow for a calculation of an ACE-III score. In view of the high correlation between ACE-R and ACE-III (correlation coefficient 0.99; Hsieh *et al*., 2013), in those few subjects for whom only ACE-III scores are available at Baseline, the ACE-III score will be used as the Baseline score for computation of change from Baseline using subsequent ACE-R scores. (Baseline efficacy assessments can be made on the day before randomization and dosing.) On-treatment, efficacy assessments will be made using these scales at Week 16, Week 32, and Week 52 (or earlier, upon study discontinuation); the Modified ADCS-CGIC will also be rated after 8 weeks in an exploratory fashion. These instruments and rater restrictions are further described in Section 7.2.

Brain MRI will be evaluated for whole brain and ventricular volumes and atrophy in frontal and temporal lobes at Weeks 16, 32 and 52 (or upon early termination) to determine whether there is

reduction in rate of brain atrophy. Change in whole brain and ventricular volumes as well as atrophy in frontal and temporal lobes will be quantified at the imaging core laboratory.

Safety assessments (described in Section 8) will be performed throughout study participation, including at Baseline and during the treatment period. Study visits during the treatment period will occur at time points approximately 4, 8, 16, 24, 32, 42, and 52 weeks after Baseline. At each visit, adverse events (AEs) will be recorded, vital signs measured, ECGs obtained, targeted physical and neurological examinations performed, clinical laboratory testing (*e.g.*, hematology, serum chemistry panels, urinalysis, Vitamin B_{12}, and folate) performed, methemoglobin and oxygen saturation measured by pulse co-oximetry, serum pregnancy testing performed (women of childbearing potential only), potential for serotonin toxicity assessed, and Columbia-Suicide Severity Rating Scale (C-SSRS) rated. Examinations will be repeated at the post-treatment follow-up visit, if applicable. TSH will be measured after 24 and 52 weeks of treatment, with a thyroid hormone panel obtained in the event of abnormality. Unscheduled visits and housing may occur as needed for assessment.

During intervening times, caregivers will be contacted by telephone at approximately 12, 20, and 28 weeks. Additional telephone contacts will occur in subjects entering the study on serotonergic medication; in addition to in-clinic observation, caregivers will be contacted by telephone at 5–7, >7-14, >14–24, 44–52, and 68–76 hours relative to the first dose (with a minimum of 1 hour between contacts). The telephone interview is presented in Section 24.8.

Blood will be collected for determination of MT concentrations prior to dosing (Visit 2) and approximately 3.5 hours after the first dose. Blood will also be collected at all subsequent visits during the treatment period (after ECG recording). These collections are restricted to those sites with a refrigerated centrifuge and appropriate freezer capacity.

In addition, a single blood sample for genotyping will be obtained only for subjects by or for whom legally acceptable informed consent for this is provided. The blood sample may be collected any time after eligibility for randomization and continued participation in the study has been confirmed at Baseline (Visit 2).

A schedule of assessments is shown in Table 4-1. Assessments are listed by visit (including unscheduled visits) in Appendix 24.3.

Table 4-1 Schedule of Assessments

Visit Name:	Screening	Baseline [a] *Day 1*		Treatment Period (52 Weeks) [b]						EOS [c] (ET)	*Safety Follow-up* [d]
Overall Visit Number:	1	2 [e]		3	4	5	6	7	8	9	10
Weeks Relative to Baseline Day:	≤ 42	*Pre-Dose*	*Post-Dose*	4	8	16	24	32	42	52	+4
Scheduled Study Day:				29	57	113	169	225	295	365	+28
(Allowable Time Window in days):				(± 3)	(± 7)	(± 7)	(± 7)	(± 7)	(± 7)	(± 14)	(± 7)
Informed Consent (Subject and Caregiver[s]) (M)	X										
**Medical History**											
Demographics	X										
Medical History (M) [f]	X										
Confirmation of Probable bvFTD diagnosis (M) [g]	X										
MRI (M) [h]	←X→					X		X		X	
MMSE [i]	X^1									X^1	
**Inclusion/Exclusion Criteria**											
Inclusion/Exclusion Criteria Review (M)	X	X									
Modified Hachinski Ischemic Score (M)	X										
El Escorial Research Criteria	X										
Randomization		X									
**Medication**											
In Clinic First Dose			X								
Study Drug Dispensing			X		X	X	X	X	X		
Compliance Assessment (tablet count)				X	X	X	X	X	X	X	
Concomitant Medication Recording/Review (M) [j]	X	X	X	X	X	X	X	X	X	X	X
**Safety Assessments**											
Adverse Events Review (M) [k]	X	X^3	X^3	X^3	X^3	X^3	X^3	X^3	X^3	X^3	X^3
Serotonin Toxicity Assessment (M) [l]		X	X	X	X	X	X	X	X	X	X
Physical and Neurological Examinations (M) [m]	X	X	X	X	X	X	X	X	X	X	X
UPDRS (Parts II and III)		X				X		X		X	
Blood and Urine Samples for Laboratory Tests [n]	X	X^3		X^3	X^3	X^3	X^3	X^3	X^3	X^3	X^3
Blood Sample for Vitamin B_{12} and Folate [n]	X	X^3		X^3	X^3	X^3	X^3	X^3	X^3	X^3	X^3
Blood Sample for G6PD, Haptoglobin [n]	X										
Thyroid Stimulating Hormone [o]	X						X			X	
Pulse co-oximetry [p]	X	X^3	X^3	X^3	X^3	X^3	X^3	X^3	X^3	X^3	X^3
Temperature, Respiratory Rate [q]	X	X	X	X	X	X	X	X	X	X	X
Blood Pressure and Pulse [r]	X	X	X	X	X	X	X	X	X	X	X
Height	X										
Weight	X	X			X	X	X	X	X	X	X
ECG [s]	X	X	X	X	X	X	X	X	X	X	X
C-SSRS [t]	X		X	X	X	X	X	X	X	X	X
**Efficacy Assessments**											
ACE-R (plus additional items for ACE-III)		X^1				X^1		X^1		X^1	
Modified ADCS-CGIC		X^2			X^2	X^2		X^2		X^2	
FRS		X^1				X^1		X^1		X^1	
FAQ		X^1				X^1		X^1		X^1	
**Other**											
Blood Sample for Population PK [u]		X	X	X	X	X	X	X	X	X	
Blood Sample for Genotyping (optional) [v]		X									

Notes: (M) = requires medical assessor

X = any study rater including X^1, X^2, or X^3 (Modified ADCS-CGIC rater cannot also rate UPDRS; see X^2 below)

X^1 = metrics rater (cannot rate X^2 or X^3)

X^2 = independent ADCS-CGIC rater (cannot rate X^1, X^3 or UPDRS)

X^3 = independent adverse events reviewer (cannot rate X^1 or X^2)

[a] The Baseline visit (Visit 2) may span over 2 days if necessary; Day 1 will commence with the administration of the first in-clinic dose

[b] Study visits during the treatment period are to occur within stipulated time windows. For example, if a visit is brought forward or delayed, the next visit should occur at a time to ensure return to the original schedule. If assessments/visits are performed outside of the stipulated time windows, these should be categorized/labeled according to the intended visit designation, regardless of being out of window (with the exception of an assessment/visit that is significantly delayed such that it falls into the stipulated time window for the following visit; in these cases, the label to be applied should be that of the following visit). An unscheduled visit is to take place if needed in response to a safety concern; an assessment/visit should only be labeled as unscheduled if it is a visit which was not planned and which was performed in addition to the protocol-defined visits. After the Week 4 visit (Visit 3), a documented telephone contact will be scheduled to occur at Weeks 12, 20, and 28 (± 7 days) and will include AE and concomitant medication review. Additional telephone contacts will occur in subjects entering the study on serotonergic medication at 5–7, >7–14, >14–24, 44–52, and 68–76 hours relative to the first dose (with a minimum of 1 hour between contacts).

[c] The end of study (EOS) is defined as completion of the Week 52 visit (Visit 9), or last dose of study drug for subjects who terminate early. If a subject withdraws from drug, the subject should be encouraged to continue with the study schedule in order to minimize missing data. If a subject withdraws from the study prematurely (before completion of Visit 9), then an early termination (ET) visit should be conducted at which time all assessments identified for Visit 9 should be performed.

[d] After completion of the study and the EOS assessments at Week 52 (Visit 9), subjects may be considered for open-label treatment with LMTM in an extension study (separate protocol). For subjects who enter the open-label extension study, the EOS visit may serve as the first visit for the extension study, and the follow-up visit for the current study will not be required. For subjects who do not enter the open-label extension study, the follow-up visit (Visit 10) should occur approximately 28 (± 7) days after the Week 52 or ET visit.

[e] At Visit 2 (Day 1), with the exception of the Efficacy assessments, Baseline assessments (assessments in *Pre-Dose* column) will be performed before administration of the first dose of study drug; assessments in the *Post-Dose* column will be performed after administration of the first dose, with timing as specified for each parameter. Efficacy assessments may be made on the prior day if necessary.

[f] Medical history within the past 10 years and any other previous medical history considered clinically relevant by the investigator will be recorded.

[g] International Consensus Criteria for probable bvFTD (Rascovsky *et al.*, 2007; Section 24.1)

[h] Magnetic resonance imaging (MRI) scans must be of sufficient quality and performed as described in a separate MRI protocol (provided to study sites). The Screening MRI must be obtained at Screening or within 42 days before Baseline; results (of sufficient quality) must be available at the Baseline visit for the purpose of inclusion/exclusion review and confirmation of compliance with inclusion and exclusion criteria by an independent neuroradiologist. If the initial Screening/Baseline MRI scan is not of sufficient quality, then a repeat Screening/Baseline MRI may be performed (within 42 days before Visit 2). If the repeat scan cannot be accomplished within the 42-day window, then the subject must be reconsented and rescreened. For subjects who are rescreened for other reasons and an acceptable MRI scan was already completed during the original screening window, the scan should only be repeated if the original MRI occurred > 42 days prior to Visit 2. For subjects who terminate early, if the subject's last MRI scan was performed < 90 days prior to the early termination date, no additional scan is required. If the subject's MRI scan was performed ≥ 90 days prior to the early termination date, the MRI scan must be repeated as part of the early termination visit assessments within the time window of the early termination visit (*i.e.*, ± 14 days of the last dose of study drug). Allowable time window extensions are discussed further in Section 9.1.2. Change in whole brain and ventricular volumes as well as atrophy in frontal and temporal lobes will be quantified at the imaging core laboratory at Weeks 16, 32, and 52 (or upon early termination); a time window of ± 14 days will be allowed for these visits.

[i] At Screening, a Mini-Mental Status Examination (MMSE) score will be obtained for eligibility determination; thereafter it is implemented as a part of the ACE-R (score reported separately at Week 52).

[j] At the Screening visit (Visit 1), medications administered within the past 3 months will be recorded; concomitant medication use will be reviewed and recorded throughout the study.

[k] Adverse events will be evaluated and recorded on an ongoing basis starting after signing of informed consent, including at each scheduled visit and at each scheduled telephone contact.

[l] Serotonin toxicity assessments are to be performed for all subjects pre-dose and for at least 4 hours after the first dose of study drug while subjects are in clinic using the Serotonin Toxicity (Syndrome) Diagnostic Interview and Rating Guide (In-Clinic). Caregivers of subjects receiving serotonergic medication will be contacted by telephone 5–7, >7–14, >14–24, 44–52, and 68–76 hours after the first dose of study drug (with a minimum of 1 hour between contacts) and interviewed using the Serotonin Toxicity Telephone Assessment; if indicated, more frequent contacts will be made.

[m] Complete physical and neurological examinations will be performed at Screening. Targeted examinations will be performed pre-dose and approximately 3 hours after administration of the first dose of study drug (Visit 2) and after 4 weeks. Targeted examinations are to be performed at Weeks 8, 16, 24, 32, 42, and 52 weeks. A targeted examination is to be performed at the off-treatment follow-up visit if applicable.

[n] At each identified time point, blood and urine samples will be obtained for hematology, serum chemistry, Vitamin B_{12} levels, folate levels, and urinalysis. At Screening only, haptoglobin levels will be measured and subjects will be screened for G6PD deficiency. For women of childbearing potential, a serum pregnancy test will be performed at Screening, each subsequent study visit during the treatment period (or upon early termination), and at the 4-week follow-up visit if applicable.

[o] Thyroid panel (T_3 and T_4) are to be measured at the next visit if TSH is abnormal; further follow-up is to be performed as needed (see Section 8.3.4)

[p] Methemoglobin and oxygen saturation will be measured using a provided pulse co-oximetry device; on Day 1, the pre-dose measurement will be made within 1 hour prior to dosing and the post-dose measurement will be made approximately 2.5 hours post-dose.

[q] Oral (sublingual) temperature measurements are preferable; aural temperature is an acceptable alternative. At Visit 2, temperature and respiratory rate will be measured within 1 hour prior to dosing and then hourly after the first dose of study drug until discharge from the study unit. At discharge, subjects (and their caregivers) using any medication with the potential to increase synaptic levels of serotonin will be instructed on signs and symptoms of potential serotonergic toxicity and given a thermometer and diary to measure and record the subject's temperature three times a day (in the morning, afternoon, and evening) for 72 hours. The diary is to be returned to the site at Visit 3.

[r] At Screening and within 1 hour prior to dosing on Day 1, blood pressure and pulse will be measured with subjects in a seated position (for at least 5 minutes) and again 2 minutes after standing; the post-dose measurement on Day 1 is to be made approximately 2 hours post-dose. Thereafter, blood pressure and pulse will be measured with subjects in the seated position only (for at least 5 minutes).

[s] A 12-lead ECG will be obtained in triplicate (within an approximate 2- to 5-minute interval) at the Screening visit and at Visit 2; subsequently (at all other visits or upon early termination), single recordings will be made, unless there are emergent abnormalities deemed clinically significant by the investigator, in which case triplicate ECGs recordings should be obtained. For subjects with well controlled atrial fibrillation at Baseline (heart rate ≤ 84 bpm and appropriate anticoagulation), triplicate ECGs are mandatory after Visit 2. At Visit 2, ECGs will be obtained before administration of the first dose of study drug (dose should be held in response to any significant abnormalities based on local interpretation) and then again approximately 3 hours post-dose. Refer to Section 6.2.2.4 for guidance regarding selected QT interval and ECG abnormalities.

[t] On Day 1 (Visit 2), the Columbia-Suicide Severity Rating Scale (C-SSRS) will be used to evaluate suicidal behavior and suicidal ideation post-dose (prior to discharge from the clinic).

[u] At Visit 2, blood will be collected on two occasions, pre-dose and again approximately 3.5 hours post-dose (only at sites with a refrigerated centrifuge and adequate freezer capacity); time of dose and sample is to be recorded at each collection.

[v] A single blood sample for genotyping will be obtained only from subjects by or for whom legally acceptable informed consent for this is provided; the blood sample may be collected any time after eligibility for randomization and continued participation in the study has been confirmed.

4.5. Randomization and Blinding

Eligible subjects will be randomly allocated to LMTM 200 mg/day or placebo groups in a ratio of 1:1 so that the numbers of subjects assigned will be approximately 90 for each of the LMTM and the placebo groups. (Also see Section 10.2 for a discussion of the sample size.)

The randomization will be stratified by region (3 levels consisting of the Americas, Europe, and Asia/Australia). The randomization list will be generated by the sponsor or designee using blocks of 4.

After signing consent, subjects will be assigned a unique subject identification number (007-CC-SSS-EE), with the first three digits for the study (007), the next two digits for the country, the next three digits for the site, and the last two digits for the sequential order of enrollment at a given site. Subjects who are rescreened will receive a new identification number; the previous number is also to be recorded by the site. After Screening assessments are performed and eligibility has been confirmed based on Baseline assessments, an eligible subject will be randomized. Randomization will be accomplished by the study site at the Baseline visit (Visit 2) using an interactive web or voice response system (IWRS or IVRS) to assign the next sequential randomization number to the eligible subject.

Treatment assignment will remain unknown to the subject and caregiver as well as to the investigator and study site personnel (double-blind) throughout the subject's participation in the study. The randomization list will be maintained in a secure location by individuals who are not

directly involved in the conduct of the study. At the end of the study, after the database is locked and subject populations have been determined, treatment assignments will be unblinded.

Regardless of randomized assignment, subjects in the two treatment groups will receive the same regimen of study drug tablets (one tablet *b.i.d.*), unless a dose reduction or interruption becomes necessary. All study drug tablets (LMTM and placebo) will be indistinguishable from one another in appearance. Study drug will be supplied to subjects in a manner that will allow for twice daily administration without breaking the blind. For further description of study treatment, see Section 6.

Because MT coloration occurs upon exposure to oxygen and treatment with LMTM is thus associated with coloration of urine and/or feces, the placebo group study drug regimen will include a low dose of LMTM (8 mg/day total). (Also see Section 6.1.1 and Section 4.6.)

The blind for an individual subject should not be broken during conduct of the study except in the case of a medical situation for which it is deemed essential to know which treatment the subject has received in order to provide appropriate care. The investigator may in an emergency unblind a specific subject and determine the identity of treatment using the IWRS/IVRS. Instructions regarding treatment identification using the IWRS/IVRS will be available in separate guidance documents. In this case, the medical monitor must be contacted and informed of any unblinding as soon as possible. The date, time, and reason for unblinding must be documented.

If a subject is unblinded, study drug will be discontinued and the subject will be followed until resolution or stabilization of the event. He/she will then be discontinued from the study.

Information about any subject for whom a code break or unblinding occurs will be provided to the DSMB by the Sponsor or designee within 15 days (within 7 days in the event of a fatal event).

Information regarding unblinding treatment allocation in relation to reporting suspected unexpected serious adverse reactions (SUSARs) is provided in Section 8.1.6.

4.6. Study Treatment

The total daily doses planned are LMTM 200 mg/day or placebo. As the placebo contains a small amount of LMTM (4 mg) in order to color the urine and feces, the actual total amounts of MT ingested daily are 200 mg/day and 8 mg/day, respectively.

Subjects will be prescribed a total of two study drug tablets daily throughout the 52-week treatment period, administered as one tablet *b.i.d.* (one tablet in the morning and one tablet in the evening). Reduction of dose by omitting the morning or evening dose may be allowed if the investigator determines this is indicated (*e.g.*, tolerability concerns or laboratory abnormalities). In the event of continued poor tolerance subjects should be withdrawn from the study. Treatment may also be interrupted for up to (maximum duration on any one occasion) 30 days during the first 6 months of treatment and/or 14 days during the second 6 months of treatment if necessary.

Study drug and regimens are further described in Section 6.1 and Section 6.2.

4.7. Concomitant Medication

All concomitant medications and medications administered within the past 3 months will be recorded in the electronic Case Report Form (eCRF) at the Screening visit (Visit 1). While in the

clinic at Baseline (Visit 2; before and after the first dose of study drug is administered), any medications administered will be recorded. "Medication" is used to encompass prescription and over-the-counter drugs or biologics, vitamins used in supra-pharmacologic doses, alternative pharmacotherapies for dementia, medical foods, and for women, forms of contraception. Current and recently used medications will also be evaluated by the investigator for serotonergic potential and date of last dose. At each scheduled outpatient visit (Visits 3-9), during each scheduled telephone contact during the treatment period, and at the follow-up visit (if applicable), any changes to existing concomitant medications and any new concomitant medications shall be reviewed and recorded.

Concomitant medications identified at Screening generally should be maintained at a constant dose for the duration of the study if clinically indicated. The investigator should evaluate any changes in the dose of existing concomitant medications and/or initiation of new concomitant medications, and the medical monitor should be contacted to discuss any concerns as needed. The date of commencement, dose, and date of any change of dose of concomitant medications are to be recorded in the eCRF.

4.7.1. Dementia Medication

Subjects taking an AChEI, memantine, or both may be enrolled if the subject has been taking such medication(s) for ≥ 3 months at Screening, if the current dosage regimen is within the locally approved dose range, if the AChEI and/or memantine total daily dose has remained unchanged for ≥ 6 weeks prior to Screening, and if the dose is not expected to change during a subject's participation in this study (see Inclusion Criterion No. 9 in Section 5.1). The only exception is that tacrine is not allowed. A subject who has had variability in the prescribed AChEI and/or memantine dose during the 6-week period prior to Screening or who is consistently not compliant with the regimen should not be enrolled.

Subjects not being treated with an AChEI or memantine also are eligible for enrollment if it is anticipated that such medication(s) will not be started during participation in this study. However, if such a subject has previously taken an AChEI and/or memantine, the last dose must have been taken ≥ 6 weeks prior to Screening.

Ideally, the AChEI and/or memantine dosage and time of dosing should remain unchanged during participation in the study. Due to lack of availability, change from immediate-release memantine to the extended-release formulation (*e.g.*, from 10 mg twice daily to 28 mg once daily) is acceptable. MT may inhibit cytochrome P450 3A4 (CYP3A4), an enzyme system that is involved in the metabolism of two of the AChEIs allowed in the study, donepezil and galantamine. Because drug-drug interaction studies have not yet been performed with these drugs, it is not known whether systemic exposure to either of these drugs will increase or by how much. Therefore, subjects receiving these drugs should be monitored and if adverse events suggest an increase in systemic exposure, dose adjustment of donepezil or galantamine may be considered. If any change in dose occurs for whatever reason, the subject should continue on study drug unless it is judged to pose a risk to the subject.

Subjects who have been receiving a medical food (*e.g.*, Axona, Souvenaid) or a stable dose of alternative pharmacotherapy for dementia (*e.g.*, Vitamin E, folate [in doses up to 5 mg/day; doses of approximately 1 mg/day in the management of folate deficiency are acceptable], a specific neurocognitive vitamin formulation [such as NeuroVits comprising 20 mg Vitamin B_6, 1 mg Vitamin B_{12}, 0.8 mg folate (see Douaud *et al*., 2013)], ginkgo biloba, hormone replacement

therapy, treatments related to coconut oil, curcumin) may continue such treatment, but the dose of such therapy must have remained stable for ≥ 6 months before randomization and it must remain constant for the duration of the subject's participation in the study. With the exception of folate, starting such therapy during participation in the study must be avoided.

There are no restrictions for other non-pharmacological treatments during the treatment period.

4.7.2. Drugs with Serotonergic Potential

As the oxidized form of MT (MT^{+}) is an inhibitor of MAO A and B *in vitro*, due to the potential for drug interaction resulting in serotonin syndrome (toxicity), recent (within five to seven plasma half-lives) or current treatment with any of the following medications (as identified by FDA in the October 2011 Safety Alert and augmented to include drugs not approved in the United States) should be undertaken only if the benefit is judged to outweigh the risk. A list (not exhaustive) of such medications and their plasma half-lives (majority taken from U.S. approved product labeling) is given below; see the Investigator's Brochure for the most current information and examples of drugs with serotonergic potential. It should be noted that some of these are available without prescription and their use should also be recorded in the eCRF.

In addition to in-clinic observation (all subjects are to be monitored in the clinic for at least 4 hours after the start of double-blind study drug), additional telephone contacts and monitoring will occur for subjects entering the study on serotonergic medication; see Section 6.2.2.2 for further information.

Drugs with serotonergic potential should not be initiated during the study; alternative therapies should be sought. The subject's general practitioner will be informed of this proscription in writing. If such medication must be started, the in-clinic and follow-up procedures described in Section 6.2.2.2 are to be followed.

**Generic Name**<br>**Metabolite (if plasma half-life available)**	**Plasma Half-life**<br>**Range (Mean, if available)**
**Selective Serotonin Reuptake Inhibitors (SSRIs)**	
Citalopram	35 hours (30-50% increase in half-life reported in elderly)
Dapoxetene	Biphasic (initial: 1.5 hours; terminal: 20 hours)
Escitalopram	27-32 hours (50% increase in half-life reported in elderly)
Fluoxetine	Acute: 1-3 days, Chronic: 4-6 days
Norfluoxetine	4-16 days (9.3 days)
Fluvoxamine	15.6 hours
Paroxetine	21 hours
Sertraline	26 hours
N-desmethylsertraline	62-104 hours
Vilazodone	25 hours
**Serotonin Norepinephrine Reuptake Inhibitors (SNRIs)**	
Desvenlafaxine	11 hours
Duloxetine	8-17 hours (12 hours); 4-hour increase in elderly half-life
Milnacipran	6-8 hours
d-milnacipran	8-10 hours
l-enatiomer	4-6 hours
Venlafaxine	3-7 hours
O-desmethylvenlafaxine	9-13 hours
**Tricyclic Antidepressant (TCA)**	
Amitriptyline	9-25 hours
Clomipramine	19-37 hours (32 hours)
Desmethylclomipramine	54-77 hours (69 hours)
Desipramine	12-27 hours
Dosulepin (Dothiepin)	19-33 hours (23-46 hours for "its metabolites")
Doxepin	15.3 hours
Nordoxepin	31 hours
Desmethyldoxepin	28-52 hours
Imipramine	9-28 hours
Nortriptyline	16-93 hours
Protriptyline	55-198 hours
Trimipramine	11-23 hours
**Monoamine Oxidase Inhibitors**	
Isocarboxazid	Unknown
Moclobemide	1-2 hours
Phenelzine	11.6 hours (single-dose)
Selegiline	10 hours
R(-)-N-desmethylselegiline	18-25 hours
R(-)-amphetamine	18-25 hours
R(-)-methamphetamine	18-25 hours
Toloxatone	Unknown
Tranylcypromine	2.5 hours
**Other Psychiatric Medicines with Serotonergic Activity**	
Amoxapine	8 hours
8-hydroxyamoxapine	30 hours
Bupropion	12-30 hours (21 hours)
Hydroxybupropion	15-25 hours (20 hours)
Erythrohydrobupropion	23-43 hours (33 hours)
Threohydrobupropion	24-50 hours (37 hours)
Buspirone	2-3 hours (single dose)
Maprotiline	51 hours
Mirtazapine	20-40 hours
Nefazodone	2-4 hours
Trazodone	10 hours (single dose)

Numerous other non-psychiatric medications also have the potential to interact with the serotonergic system and their use is similarly restricted (*i.e.*, additional monitoring by telephone after the first dose). These include drugs such as:

- MAO inhibitors used for non-psychiatric indications (*e.g.*, rasagiline, isoniazid, procarbazine, linezolid)
- Other medications associated with severe serotonin toxicity (*e.g.*, tramadol, pethidine, fentanyl, chlorphenamine, dextromethorphan, tryptophan, dexamphetamine)
- Lithium, valproate, metoclopromide, propranolol, bromocriptine ($5HT_{1A}$)
- Drugs used for weight loss (*e.g.*, lorcaserin)
- "Triptans" used in the treatment of migraine (sumatriptan, rizatriptan, naratriptan, zolmitriptan, eletriptan, almotriptan, frovatriptan)
- St. John's wort (*Hypericum perforatum*) and *panax ginseng*

Note, dietary supplements containing tryptophan or its metabolite 5 hydroxytryptophan (5-HTP) are considered serotonergic concomitant medications as 5-HTP is a precursor in the biosynthesis of serotonin. Tryptophan is a naturally-occurring protein, however, eating foods containing tryptophan does not significantly increase 5-HTP levels; thus, avoiding dietary sources of tryptophan is not necessary.

In the event of any question, the medical monitor should be consulted.

4.7.3. CYP and P-gp Substrates

Results of a completed drug-drug interaction study indicate that MT is a weak inhibitor of CYP3A4, CYP2C8, and CYP2C19 enzymes (see the Investigator's Brochure for examples of drugs metabolized by these enzymes). The extent to which this occurs within an individual or with a given drug is not known, especially for those drugs with multiple metabolic pathways. Therefore, subjects on drugs known to be metabolized by one or more of these enzymes (especially those that have a narrow therapeutic index) should be closely monitored for adverse events that could suggest an increase in systemic exposure. Dose adjustment of the concomitant medication may be warranted.

LMTM is also a weak inducer of CYP2B6 and the P-glycoprotein (P-gp) transporter (see the Investigator's Brochure for examples of substrates). Coadministration of LMTM with digoxin, a P-gp substrate, was shown to result in decreased concentrations of digoxin. Therefore, it is advisable to obtain a baseline digoxin level in subjects on this drug and to monitor digoxin levels periodically while on study. Any such results obtained from the local laboratory should be entered into the eCRF (together with the time of the prior dose of digoxin and the time of the sample).

4.7.4. Drugs Used to Manage Behavioral Disturbance

Subjects may be treated with antipsychotics (other than clozapine or olanzapine) provided they have been used in a stable dose and regimen for at least 3 months prior to Baseline. There should be no intent to initiate such therapy during the course of the study. Should treatment be initiated, the reason(s) should be clearly documented by indicating one or more of the following reasons: delusions, hallucinations, agitation/aggression, depression, anxiety, elation/euphoria, apathy/indifference, disinhibition, irritability/lability, aberrant motor behavior, nighttime behavior, or appetite/eating change.

"As-needed" use of antipsychotics is to be avoided if possible, but such use does not preclude further participation. Similarly, regular or occasional use of benzodiazepines to manage distress, agitation, *etc.* does not preclude further participation. Where possible, these should not be used within the 12 hours prior to cognitive testing.

4.7.5. Other Medications

Other medications listed in Exclusion Criterion No. 23 (see Section 5.2) are specifically prohibited during participation in this study.

Anxiolytics and/or sedatives/hypnotics may be used as sedation for claustrophobia or agitation or to manage excessive movement during imaging. Regular or occasional benzodiazepines, chloral hydrate, low dose trazodone[4] (50 mg) or zolpidem may be used as needed at bedtime for sleep.

Unless otherwise prohibited, concomitant medications (preferably at stable doses) considered appropriate by the subject's physician are allowable but should be kept to the minimum possible as clinically indicated. If there are questions about whether or not a medication is permitted in the study, the medical monitor should be consulted.

4.7.6. Dietary Tyramine

Historically, MAO inhibitors as a class have been reported to be associated with hypertensive crises caused by ingestion of foods containing high amounts of tyramine (known as a tyramine or "cheese" reaction). While there is a theoretical potential for a tyramine reaction with MT, there have been no reports to date in subjects taking part in TauRx-sponsored studies, even though there have been no dietary restrictions in these studies. Nonetheless, as a precaution, subjects and their caregivers should be advised about this potential while taking LMTM (see the Investigator´s Brochure for examples of tyramine-rich foods and beverages, such as air-dried, aged or fermented meats and cheeses, fava bean pods, non-pasteurized beers, sauerkraut, and most soybean products). They should also be advised to seek medical care immediately in the event of signs or symptoms of hypertensive crisis (sudden onset of severe headache, nausea, stiff neck, tachycardia or palpitations, profuse sweating, and/or confusion) or other sudden or unusual symptoms following ingestion of tyramine-rich foods or beverages.

4.7.7. Contraceptive Measures

As a precautionary measure, women of childbearing potential (*i.e.*, not documented to be post-menopausal for at least 1 year or not having undergone hysterectomy or bilateral salpingectomy or oophorectomy for at least 6 months minimum) must use adequate contraception, with the exception of female subjects in Italy. Examples of adequate contraception include bilateral tubal ligation or occlusion at least 6 months prior to Baseline; use of a barrier method (condom, diaphragm, or cervical/vault cap) with spermicidal foam, gel, film, cream, or suppository; intrauterine device (IUD) or system, or oral or long acting injected or implanted hormonal contraceptives for at least 3 months prior to Baseline; or sexual activity restricted to a vasectomized partner (with the appropriate post-vasectomy documentation of the absence of spermatozoa in the ejaculate). Abstinence is only acceptable as true abstinence when this is in line with the subject's preferred and usual lifestyle; periodic abstinence (*e.g.*, calendar, ovulation,

[4] If trazodone is used in this way, steps must be implemented for monitoring signs and symptoms indicative of potential serotonin toxicity.

symptothermal, post-ovulation methods) and withdrawal are not acceptable methods of contraception). Subjects must be competent to use adequate contraception and must agree to continue to maintain adequate contraceptive measures throughout study participation and until the final off-treatment visit. In Italy, subjects must have avoided a pregnancy for at least 3 months prior to Baseline and must accept to avoid a pregnancy throughout participation in the study. Women of childbearing potential should be encouraged to return to the clinic in the event of a delayed menstrual period to rule out possible pregnancy.

Males should practice abstinence or use acceptable birth control with any female sexual partner of childbearing potential. The risk of drug secretion through the ejaculate is not fully studied. To ensure that the fetus is not exposed to MT through vaginal absorption, male subjects (including men who have had vasectomies) whose partners are pregnant should use condoms for the duration of the study and for an additional 4 days after cessation of study treatment. The investigator must provide appropriate counsel to male subjects regarding this issue.

4.7.8. Folate and Vitamin B_{12}

The manufacturer of the test kits used by the central laboratory for measuring folate and Vitamin B_{12} (see Section 8.3.4) has established the following normal ranges (healthy U.S. males and females aged 18 years and older): 5.9-24.8 ng/mL for folate and 180-914 pg/mL for Vitamin B_{12}. However, these values are not applicable for all geographies as the food in the United States is supplemented with these vitamins. Prior to initiating study drug, subjects with folate levels < 4.0 ng/mL or Vitamin B_{12} levels < 150 pg/mL (*i.e.*, deficient according to WHO Technical Consultation 2008, 2012), should be supplemented. Subjects with folate levels < 4.0 ng/mL may be entered into the study provided they are supplemented (approximately 1 mg/day folate) from Visit 2 onwards. Subjects with Vitamin B_{12} levels < 150 pg/mL should be referred to their primary care physician for evaluation and treatment or the medical monitor consulted. There must be a treatment plan in place for any applicable chronic condition. If a condition is diagnosed that the primary care physician believes cannot be reliably or continuously corrected, the subject should be excluded from the study. If review and correction can be achieved within the 42-day screening window, the subject may be entered into the study; otherwise the subject must be reconsented and rescreened after the deficit has been corrected.

Folate and Vitamin B_{12} are necessary for the production and function of red blood cells. Given the risk of anemia with the use of MT, it is important that subjects not become deficient in folate and/or Vitamin B_{12} while on study. Furthermore, low levels of either could be a confounding factor contributing to cognitive impairment (Allen *et al.*, 2013; Moore *et al.*, 2012; Nachum-Biala and Troen, 2012; O'Leary *et al.*, 2012; Malouf and Grimley, 2008; Malouf and Sastre, 2003). For these reasons, folate and Vitamin B_{12} are to be monitored throughout the study. Subjects should be referred to their primary physician for evaluation of causation and correction or the medical monitor consulted in the event that levels of either become deficient. For Vitamin B_{12}, this evaluation may also include measurement of homocysteine and methylmalonic acid (by a local laboratory) to determine whether or not the low levels are physiologically significant.

Chronic hemolytic anemia depletes folate stores, and it is general medical practice to place all hematology patients with chronic hemolytic anemia on prophylactic folate replacement, with a recommended dose of 1 mg by mouth, per day.

4.8. Data and Safety Monitoring Board / Independent Data Monitoring Committee

Safety will be overseen by a DSMB throughout the duration of study conduct. At any time, the DSMB may recommend that dosing be modified or enrollment stopped due to safety concerns; the DSMB may also request to receive additional data unblinded to the subject level in response to identified safety concerns.

Routine meetings are to be scheduled as determined by the DSMB. *Ad hoc* meetings will be convened if needed in response to safety concerns. This DSMB will also be assessing data from other clinical studies sponsored by TauRx with the same active moiety. The DSMB Charter will describe the composition of the DSMB and safety monitoring details, as well as the frequency of meetings needed as the study progresses.

5. SELECTION OF SUBJECTS AND CRITERIA FOR WITHDRAWAL

5.1. Inclusion Criteria

To be eligible for enrollment in this study, a subject must meet all of the following inclusion criteria:

1. Diagnosis of probable bvFTD according to the International Consensus Criteria for bvFTD (Rascovsky *et al.*, 2007; Section 24.1)
2. Centrally rated frontotemporal atrophy score of 2 or greater, taken as the maximum of right or left frontal or anterior temporal lobes (Kipps *et al.*, 2007) on brain MRI of sufficient quality obtained at Screening or within a maximum of 42 days before Baseline, irrespective of pre-existing structural or functional imaging evidence supporting a diagnosis of bvFTD
3. MMSE ≥20 at the Screening visit
4. Age <80 years at the Screening visit
5. Modified Hachinski ischemic score of ≤4 at the Screening visit
6. Females must meet one of the following:
    - Surgically sterile (hysterectomy, bilateral salpingectomy / oophorectomy) for at least 6 months minimum
    - Have undergone bilateral tubal occlusion / ligation at least 6 months prior
    - Post-menopausal for at least 1 year
    - Using adequate contraception (a barrier method [such as condom, diaphragm, or cervical/vault cap] with spermicidal foam, gel, film, cream, or suppository; intrauterine device [IUD] or system, or oral or long-acting injected or implanted hormonal contraceptives for at least 3 months prior to Baseline; or vasectomized partner [with the appropriate post-vasectomy documentation of the absence of spermatozoa in the ejaculate]) or true abstinence (when this is in line with the preferred and usual lifestyle of

the subject); subjects must be competent to use adequate contraception and to agree to continue to maintain adequate contraception throughout participation in the study
OR
In Italy, have avoided a pregnancy for at least 3 months prior to Baseline and accept to avoid a pregnancy throughout participation in the study

7. Subject and/or, in the case of reduced decision-making capacity, legally acceptable representative(s) consistent with national law is/are able to read, understand, and provide written informed consent in the designated language of the study site
8. Has one or more identified adult caregivers who meet the following criteria:
   - Either lives with the subject or sees the subject on average for ≥ 2 hours/day ≥ 3 days/week, or in the investigator's opinion, the extent of contact is sufficient to provide meaningful assessment of changes in subject behavior and function over time and provide information on safety and tolerability
   - Is willing to provide written informed consent for his/her own participation
   - Is able to read, understand, and speak the designated language at the study site
   - Agrees to accompany the subject to each study visit
   - Is able to verify daily compliance with study drug
9. If currently taking an AChEI (*i.e.*, donepezil, galantamine, or rivastigmine) and/or memantine, at the time of Screening:
   - The subject must have been taking such medication(s) for ≥ 3 months
   - The current dosage regimen and dosage form must be within the locally approved dose range and must have remained stable for ≥ 6 weeks
   - It must be planned that the dosage regimen will remain stable throughout participation in the study

   Subjects not being treated with an AChEI or memantine (for ≥ 6 weeks before Screening) may also be enrolled if initiation of an AChEI or memantine is not planned for the time period during which the subject will be participating in this study
10. Able to comply with the study procedures in the view of the investigator

5.2. Exclusion Criteria

In order to be eligible for this study, a subject must not meet any of the exclusion criteria listed below:

1. Significant CNS disorder other than bvFTD, *e.g.*, Alzheimer's disease, Lewy body dementia, Parkinson's disease, multiple sclerosis, progressive supranuclear palsy, hydrocephalus, Huntington's disease, any condition directly or indirectly caused by Transmissible

Spongiform Encephalopathy (TSE), Creutzfeldt-Jakob Disease (CJD), variant Creutzfeldt-Jakob Disease (vCJD), or new variant Creutzfeldt-Jakob Disease (nvCJD)

2. Other significant intracranial pathology seen on brain MRI scan that would lead to a diagnosis other than probable bvFTD or that puts the subject at risk of ARIA, including:
   - Large confluent white matter hyperintense lesions (*i.e.*, Fazekas score of 3)
   - Other focal brain lesion(s) judged clinically relevant by the investigator
   - A single area of superficial siderosis
   - > 4 Cerebral microhemorrhages (regardless of their anatomical location or diagnostic characterization as "possible" or "definite")
   - Evidence of a prior macrohemorrhage

3. Biomarker evidence of underlying AD pathology as etiology of dementia

4. Expressive language deficits such that the subject is too severely impaired to allow testing at Baseline

5. Meets research criteria (El Escorial) for Amyotrophic Lateral Sclerosis or motor neuron disease (Section 24.2); evidence of mild motor neuron disease on examination is allowed if not expected in investigator’s opinion to interfere with subject’s completion of study but prominent bulbar symptoms, indicating high risk for respiratory compromise, would be exclusionary

6. Meets diagnostic criteria for probable bvFTD but has a proven mutation producing non-tau, non-TDP-43 pathology (*e.g.*, FUS CHMP2B)

7. Clinical evidence or history of any of the following within specified period:
   - Cerebrovascular accident (2 years)
   - Transient ischemic attack (6 months)
   - Significant head injury with associated loss of consciousness, skull fracture or persisting cognitive impairment (2 years)
   - Other unexplained or recurrent loss of consciousness ≥ 15 minutes (2 years)

8. Epilepsy (a single prior seizure is considered acceptable)

9. Rapid eye movement sleep behavior disorder

10. DSM IV-TR criteria met (and not subsequently revised) for any of the following within specified period:
    - Major depressive disorder (current)
    - Schizophrenia (lifetime)
    - Other psychotic disorders, bipolar disorder (within the past 5 years), or substance (including alcohol) related disorders (within the past 2 years)

11. Metal implants in the head (except dental), pacemaker, cochlear implants, or any other non-removable items that are contraindications to MR imaging; MR compatible prosthetics, clips, stents, or any other device proven to be compatible will be allowed

12. Resides in hospital or moderate to high dependency continuous care facility (residence in low grade assisted living facility where there is sufficient autonomy to permit evaluation of activities of behavior and general functioning is allowed so long as it is not mandated by an order issued either by the judicial or the administrative authorities)

13. History of swallowing difficulties (note: study drug should be swallowed whole and MUST NOT be broken, crushed, chewed, or dissolved in fluids prior to ingestion)

14. Pregnant or breastfeeding

15. G6PD deficiency

16. History of significant hematological abnormality or current acute or chronic clinically significant abnormality, including:
    - History of hereditary or acquired methemoglobinemia or Baseline measurement of methemoglobin (MetHb) > 2.0% (confirmed on repeat)
    - History of hemoglobinopathy, myelodysplastic syndrome, hemolytic anemia, or splenectomy
    - Screening hemoglobin value (confirmed upon repeat) below age/sex appropriate lower limit of the central laboratory normal range

    Subjects in whom folate is < 4.0 ng/mL may be entered into the study provided folate supplementation (approximately 1 mg/day) is initiated and maintained for the duration of the study (see Section 4.7.8).

    Subjects in whom Vitamin B_{12} is < 150 pg/mL should be evaluated and supplemented as appropriate prior to the initiation of study drug (see Section 4.7.8). If review and correction can be achieved within the 42-day screening window, the subject may be entered into the study; otherwise the subject must be reconsented and rescreened after the deficit has been corrected.

17. Abnormal serum chemistry laboratory value at Screening deemed to be clinically relevant by the investigator (*e.g.*, those considered to have the potential to increase the risk associated with study participation or administration of investigational product and, in the judgment of the investigator, would make the subject inappropriate for entry into this study). In addition, subjects with either of the following abnormalities must be excluded:
    - Creatinine clearance < 50 mL/min at Screening, estimated by the central laboratory according to the Cockcroft and Gault equation
    - Thyroid stimulating hormone (TSH) above laboratory normal range (subject may be treated and rescreened after 3 months)

18. Clinically significant cardiovascular disease or abnormal assessments (in the opinion of the investigator) such as:
    - Hospitalization for acute coronary syndrome (acute myocardial infarction or unstable angina) or symptoms consistent with angina pectoris, within the 12 months preceding Baseline
    - Signs or symptoms of clinical heart failure within the 12 months preceding Baseline

- Evidence of uncontrolled atrial fibrillation on Screening ECG or history of atrial fibrillation that is not currently controlled (heart rate ≥ 85 bpm and/or inappropriate anticoagulation) or where the QT interval cannot in the opinion of the Investigator be assessed by triplicate ECGs taken within an approximate 2- to 5-minute interval (if better control of the heart rate and/or of anticoagulation can be achieved after adequate treatment, subject may be entered into the study if still within the 42-day window, or else the subject must be reconsented and rescreened). A cardiology consult should be sought for further ECG evaluation (especially in subjects with left bundle branch block) if deemed necessary by the investigator.
- QTcF (based on mean of three triplicate ECGs, QT corrected for heart rate using Fridericia's formula) at Screening > 460 msec in males or > 470 msec in females, or low or flat T waves making measurement of QT interval unreliable
- Recent history of poorly controlled hypertension, systolic blood pressure > 160 mmHg, or diastolic blood pressure > 100 mmHg, after 5 minutes in a seated position at Screening
- Hypotension: systolic blood pressure < 100 mmHg after 5 minutes in a seated position at Screening
- Heart rate < 48 bpm or > 96 bpm by measurement of vital signs (after 5 minutes in a seated position) or by ECG at Screening

19. Preexisting or current signs or symptoms of respiratory failure (*e.g*., caused by chronic obstructive pulmonary disease, bronchial asthma, lung fibrosis, or other disease)
    - Subjects with previously diagnosed moderate to severe sleep apnea not adequately controlled (in the opinion of the investigator) should be excluded
20. Concurrent acute or chronic clinically significant (in the opinion of the investigator) immunologic, hepatic (such as presence of encephalopathy or ascites), or endocrine disease (not adequately treated) and/or other unstable or major disease other than bvFTD
    - Subjects with hepatitis or primary biliary cirrhosis should be excluded
    - Human T-Cell Lymphocytic Virus Type III (HTLV-III), Lymphadenopathy Associated Virus (LAV), any mutants or derivatives of HLTV-III or LAV, any condition associated with Acquired Immunodeficiency Syndrome or similar condition however named
21. Diagnosis of cancer within the past 2 years prior to Baseline (other than basal cell or squamous cell skin cancer or Stage 1 prostate cancer) unless treatment has resulted in complete freedom from disease for at least 2 years
22. Prior intolerance or hypersensitivity to MT-containing drug, similar organic dyes, or any of the excipients
23. Treatment currently or within 3 months before Baseline with any of the following medications (unless otherwise noted; see Section 4.7.4):
    - Tacrine
    - Amphetamine or dexamphetamine
    - Antipsychotics
        - Clozapine, olanzapine (and there is no intent to initiate therapy during the course of the study)

    - Other antipsychotics are allowable provided they have not been initiated within 3 months before Baseline and are used in a stable dose and regimen
- Carbamazepine, primidone
- Drugs for which there is a warning or precaution in the labeling about methemoglobinemia at approved doses (*e.g*., dapsone, local anesthetics such as benzocaine used chronically, primaquine and related antimalarials)

24. Current or prior participation in a clinical trial as follows:
    - Clinical trial of a product for cognition prior to Screening in which the last dose was received within 90 days prior to Screening unless confirmed to have been randomized to placebo
    - A clinical trial of a drug, biologic, device, or medical food in which the last dose was received within 28 days prior to Baseline

Subjects who fail Screening may be rescreened if the inclusion or exclusion criterion that initially led to Screening failure has changed and may render the subject potentially eligible; initial screening data and rescreening data will be captured in the database. The investigator should contact the medical monitor if there are any questions or concerns.

5.3. Discontinuation/Withdrawal

For a discussion of reasons for permanent discontinuation of study medication on the basis of safety, see Section 6.2.2. These include, but are not limited to, elevation in MetHb and/or clinically evident hemolytic anemia (Section 6.2.2.1), serotonin syndrome (Section 6.2.2.2), ARIA (Section 6.2.2.3), prolongation of the QT interval on ECG (Section 6.2.2.4), and decrease in renal function (Section 6.2.2.5).

Subjects may discontinue study drug and withdraw (drop out) from the study at any time for any reason. The caregiver may also withdraw his or her consent at any time for any reason. If a caregiver withdraws his or her consent, the subject must then also be withdrawn if alternative arrangements are not available for the completion of the ACE-R assessment (*e.g.*, telephone or face-to-face interview with an alternate consented caregiver). Furthermore, the investigator also has the right to discontinue trial medication if he or she judges that treatment is no longer appropriate, the subject's clinical condition is worsening, or for an adverse event.

If study drug is discontinued, the reason should be recorded as one of the following:

- AE
- Death
- Lack of efficacy (including progressive disease or worsening of cognitive capacity)
- Lost to follow-up
- Withdrawal by subject or legal representative (or caregiver), including specific reasons wherever possible
- Protocol deviation
- Non-compliance with study drug
- Pregnancy

- Study terminated by sponsor
- Physician decision, including specific reason(s) wherever possible
- ARIA
- Other (specify)

If the reason for discontinuation of study drug is an AE, the principal event associated with discontinuation must be specified and recorded. In this case, reasonable effort must be made to clearly document the outcome. If the reason for premature discontinuation is an SAE, this must be documented and an SAE form must be completed (also see Sections 8.1.4 and 8.1.5).

For subjects who withdraw from the study for reasons other than death, lost to follow-up, or subject or caregiver consent withdrawn, a visit should be scheduled as soon as possible after the last dose of study drug and identified end of treatment safety evaluations performed. For subjects who withdraw consent or when a caregiver withdraws consent without available alternate caregiver, the investigator should request that the reason be specified and the subject have any clinically indicated safety assessments performed.

Subjects withdrawn from the study will not be replaced nor can they be re-enrolled (or enter the open-label trial unless discontinued for lack of efficacy). Finally, such subjects should be encouraged to return for a post-treatment visit approximately 28 days after the last dose of study drug and to continue with study visits until the scheduled end of participation for the subject.

5.4. Termination of the Study

The Sponsor reserves the right to terminate the study for duly justified reasons in accordance with the national laws (*e.g.*, in Germany: §40, section 1 of the Arzneimittelgesetz [AMG]). These reasons include in particular:

- Administrative reasons: *e.g.*, financial reasons
- Interest of subject welfare: *e.g.*, new information or events that result in an unfavorable risk-benefit profile.

6. TREATMENTS ADMINISTERED

6.1. Study Drug

Study drug tablets are formulated as blue film-coated oval tablets. All study drug tablets are visually indistinguishable from one another in order to preserve blinding. The active and inactive ingredients are described below.

6.1.1. Active Ingredient

The active ingredient, MT, is provided as leuco-methylthioninium bis(hydromethanesulfonate) (LMTM, TRx0237). The chemical name is *N,N,N′,N′*-tetramethyl-10*H*-phenothiazine-3,7-diaminium bis(methanesulfonate). The contents of the tablets used in the study are:

- LMTM tablet containing 100 mg (expressed as MT base equivalent)

- Placebo tablet containing 4 mg LMTM (MT base equivalent)

Placebo tablets containing a low amount of LMTM (4 mg) are provided in order to prevent inadvertent unblinding that might otherwise occur because of a known effect of LMTM to cause urine and/or fecal coloration. The small total daily dose, 8 mg/day, is considered unlikely to have significant efficacy in bvFTD or result in adverse effects.

The potential for LMTM to cause urinary and/or fecal coloration (or coloration of other bodily fluids) should be explained to the subject and caregiver and will be described in the informed consent form (ICF). If a subject is known to have incontinence, coloration could adversely affect compliance with study drug unless adequate precautions are taken (*e.g.*, use of incontinence pads). Even with the latter, discoloration in the context of incontinence may prove unacceptable to the subject/caregiver, and subjects should be entered into the study only after careful discussion of this possibility with them. Staining of underclothes and other fabrics is difficult to remove using standard washing products; therefore, subjects and caregivers should be informed about available techniques for washing stained clothing (see the Investigator’s Brochure). The possibility of such staining should be clearly discussed with subjects and their caregivers.

6.1.2. Inactive Ingredients

Tablets also contain the following inactive compendial excipients: mannitol, crospovidone, microcrystalline cellulose, and magnesium stearate.

The film coat of study drug tablets contains polyvinyl alcohol-part hydrolyzed, talc, titanium dioxide, Macrogol PEG 3350, lecithin (soya), as well as non-compendial FD&C blue #2 (indigo carmine aluminum lake).

6.2. Study Regimens

6.2.1. Double-Blind Study Drug (LMTM or Placebo)

Subjects will be randomly assigned to one of two treatment groups as described in Section 4.5: LMTM 200 mg/day or placebo. The planned daily study drug regimen, including the number of tablets and LMTM doses for each of the treatment groups, is summarized in Table 6-1. All LMTM doses cited below represent doses expressed as MT base equivalents. The study supplies are further described in Section 6.3.

Table 6-1 Planned Daily Study Drug Regimen: Tablets and LMTM Dose

Treatment Group	Tablet Contents (amount of MT)	Number of Tablets			Dose of LMTM		
		AM	*PM*	Daily Total	*AM*	*PM*	Daily Total
LMTM 200 mg/day	100 mg	1	1	2	100 mg	100 mg	200 mg
Placebo	4 mg	1	1	2	4 mg	4 mg	8 mg

AM = morning; PM = evening

The first dose of study drug (one tablet) will be administered to subjects at the study site under supervised conditions. Thereafter, study drug tablets will be taken orally twice a day (one tablet each morning and one tablet each evening) on an outpatient basis. Morning and evening doses

generally should be separated from one another by≥ 10 hours. Subjects will be instructed to take each dose of study medication with a full glass of water (see Section 6.4 for discussion of study drug dispensing).

Subjects and caregivers will be instructed that study drug should be swallowed whole and MUST NOT be broken, crushed, chewed, or dissolved in liquids prior to ingestion. If there are swallowing difficulties which prevent taking the medication as instructed, subjects should not be entered into the study. Subjects and caregivers should be warned that if the product is not swallowed immediately and is allowed to dissolve in the mouth, it will cause discoloration of teeth and oral mucosa.

6.2.2. Dose Interruption/ Reduction/ Discontinuation

During the treatment period, reduction of dose may be allowed if the investigator determines this is indicated (*e.g*., tolerability concerns or laboratory abnormalities). The dose reduction is to be by omission of the morning or evening tablet. If one tablet daily is not tolerated, the subject should be withdrawn from the study.

Interruption of study drug administration of up to (maximum duration on any one occasion) 30 days during the first 6 months of treatment and/or 14 days during the second 6 months of treatment and resumption of dosing at a reduced or full dose is also allowed. If after allowable interruptions, the subject does not tolerate re-introduction of the study drug or a clinically significant laboratory abnormality recurs, then study drug should be discontinued.

Guidelines for the monitoring and management of adverse events of special interest are given below. Clinical circumstances requiring additional evaluation of a subject and/or potential interruption or discontinuation of study drug are discussed.

Subjects discontinuing treatment will be encouraged to return for a post-treatment follow-up visit approximately 28 days after the last dose and to continue with study visits until the scheduled end of participation for the subject.

6.2.2.1. Methemoglobinemia and/or Hemolytic Anemia

Any MetHb value recorded as greater than 3.5% should be confirmed by repeat measurement after 1 week. At any time, an elevated value (greater than 2.0%) should be immediately repeated and confirmed; the procedure for immediate repeat measurements is described in Section 8.4. In the event of elevation, the mean of three readings will be used as the basis for safety monitoring decisions. Dose interruptions and/or dose reductions are allowable in order to manage the event.

Elevations in MetHb and/or clinically evident hemolytic anemia are to be managed as summarized in Table 6-2, including an unscheduled visit after 1 week. (See Section 4.7.8 for guidance on folate and Vitamin B_{12}.)

Table 6-2 Monitoring and Management of Elevated Methemoglobin

Methemoglobin	Monitoring	1-Week Follow-up Outcome	Action
>3.5% - < 5.0%	Unscheduled visit after 1 week	≤3.5%	Continue dosing
		Stable >3.5% - ≤4.5%	Continue with weekly monitoring
		Increased by > 1.0%	Interrupt dosing and continue with weekly monitoring until decreases to ≤3.5%[1]; resume dosing at reduced dose (omit the morning or evening dose)
≥5.0%	Discontinue dosing permanently, and continue monitoring until decreases to≤3.5%		

[1] If dosing is held for more than 30 consecutive days during the first 6 months of study or 14 consecutive days during the second 6 months of study, subject should be discontinued (see Section 5.3)

For symptomatic methemoglobinemia or when there are signs and/or symptoms of hemolytic anemia, dosing should be discontinued. In addition to the action taken with the dose, hematology testing (RBC panel as described in Section 8.3.2) should be performed as well as measurement of LDH and total, direct and indirect bilirubin. Haptoglobin values should be obtained within 1 week. If abnormal, repeated measurements should continue to be obtained until normal values are noted or levels stabilize at a level acceptable to the investigator. It is acceptable to perform such repeat testing at a local laboratory provided the results are provided to the site (and recorded in the eCRF). As described in Section 8.3.2, determination of Heinz bodies may also be considered; such testing is to be performed at the local laboratory. If Heinz bodies are present, study medication must be stopped, but hematological indices should be monitored until this resolves. The medical monitor should be contacted to discuss whether or not resumption of dosing is indicated or for any other questions.

In addition to elevated methemoglobin, the signs and symptoms of methemoglobinemia or anemia include:

- Cyanosis, headache, anxiety, exercise intolerance, fatigue, confusion, dizziness, tachypnea, palpitation, dysrhythmia, seizures, and coma

Signs of possible hemolytic anemia include:

- Decrease by 20% from Screening in RBC count and/or hemoglobin
- Abnormal RBCs in peripheral blood smear
- Elevation of reticulocyte count to above laboratory normal range
- Increase in LDH or indirect bilirubin, or low haptoglobin

6.2.2.2. Serotonin Syndrome

Subjects are to be monitored for signs and symptoms indicative of potential serotonin toxicity. All subjects should remain in the clinic for at least 4 hours after the first dose of study drug. While in the clinic, subjects should be evaluated by a medically qualified person by targeted physical and neurological examination and measurement of oral (sublingual) temperature (aural temperature is an acceptable, but less preferable, alternative), respiratory rate, blood pressure,

and pulse. The Serotonin Toxicity (Syndrome) Diagnostic Interview and Rating Guide provides further guidance (see Section 24.7). An assessment is to be completed just prior to discharge from the clinic. If a subject meets any one of four possible criteria for serotonin syndrome, no further study drug should be administered and the subject managed as medically appropriate. Subjects without significant signs and symptoms may be discharged from the study unit.

For subjects entering the study on a serotonergic drug (see Section 4.7.2), subjects and their caregivers should remain in close proximity to a local hospital and remain in telephone contact with the clinic for at least 12 - 14 hours after the first dose of study drug; reimbursement for accommodations will be made available if requested by subjects and/or their caregivers. They shall be provided with monitoring instructions, a diary, and a thermometer at the time of Day 1 discharge from the study unit and instructed in its use. Temperature should be monitored three times a day (in the morning, afternoon, and evening) until 72 hours after the first dose and will be recorded in the diary to be returned at Visit 3 (Week 4). Should any changes be noted, they should call the clinic and return to the clinic if so instructed or seek urgent medical care. In addition, caregivers are to be contacted by telephone 5–7, >7–14, >14–24, 44–52 and 68–76 hours after the first dose of study drug (with a minimum of 1 hour between contacts); if indicated, more frequent contacts will be made, and the site will assume responsibility for clinical review and hospital referral. Instructions for the assessment are provided in the Serotonin Toxicity Telephone Assessment in Section 24.8.

The same procedures apply to any subject newly starting treatment with a serotonergic drug.

Table 6-3 Overview of Monitoring and Management of Serotonin Toxicity (Syndrome)

**Signs and Symptoms of Serotonin Toxicity[1]**	**Monitoring after First Dose and at Each Clinic Visit**	**Action**
**Any one of the following:**<br>Autonomic findings (temperature ≥38 ºC, diaphoresis, shivering, tachypnea/dyspnea, diarrhea, hypertension or hypotension)<br>Cognitive changes (agitation/akathisia, elevated mood, insomnia)<br>Neuromuscular changes (tremor; hyperreflexia; spontaneous, inducible, or ocular clonus; muscle rigidity[2]; hypertonia; dizziness; incoordination; or mydriasis)	Targeted PE and neurological examination, focused on the following:<br>deep-tendon reflexes, clonus, and muscle rigidity; size and reactivity of pupils; dryness of oral mucosa; intensity of bowel sounds; skin color; and presence or absence of diaphoresis	Discontinue study drug<br>Appropriate medical management to be undertaken
	Oral (sublingual) temperature[3]	
	Seated blood pressure and pulse	
	MMSE (Baseline and Weeks 16, 32, and 52) and as needed to respond to changes	

[1] See Section 24.7 for an interview and monitoring guide and rating scale
[2] Muscle rigidity can overwhelm other neuromuscular findings and mask the diagnosis
[3] Oral (sublingual) temperature measurements are preferable; aural temperature is an acceptable alternative (use of alternate measurement is to be noted in eCRF)

6.2.2.3. ARIA

Brain MRI will be performed at Screening or within 42 days before Baseline to confirm eligibility and at Weeks 16, 32, and 52 (± 14 days) or early termination for evaluation of whole brain volume (a primary objective) as well as ventricular volume and atrophy in frontal and

temporal lobes (an exploratory objective). Post-Baseline MRI scans will not be independently evaluated for ARIA at regular intervals (as of implementation of Amendment 6.0); however, if ARIA is suspected and the site submits images to the imaging core laboratory for review, an independent review for ARIA may take place. All investigators should be aware of the imaging manifestations of ARIA and the clinical signs and symptoms that may accompany this phenomenon. Any subject identified as having ARIA meeting the termination criteria listed in Table 6-4 must be permanently discontinued from the study. The subject and caregiver are to be informed of the reason.

Imaging abnormalities consistent with ARIA include the following:

- vasogenic edema, macrohemorrhage
- an area of superficial siderosis
- clinically symptomatic microhemorrhage(s)
- >4 clinically asymptomatic new microhemorrhages

The following signs and symptoms have been reported in trial subjects found to have ARIA: confusion (with or without hallucination) or acute decline in cognition, headache, gait disturbance/ataxia, vomiting or upper gastrointestinal disturbance, and acute development of focal neurological signs. Consideration should be given to treatment with high-dose dexamethasone should symptoms be severe.

Follow up of subjects with ARIA includes repeat scanning after the discontinuation of the study drug in order to evaluate their stability until the imaging abnormalities are resolved or stabilized. Scanning every 6 weeks should be performed and resolution/stabilization should be determined based on three follow-up scans. The follow-up scans will be sent to the imaging core laboratory for evaluation of ARIA and will also be evaluated for whole brain and ventricular volumes as well as atrophy in frontal and temporal lobes.

The following table provides a summary of the appropriate imaging follow-up to be performed in response to ARIA findings.

Table 6-4 Overview of ARIA Findings Management Plan

**Finding Type**	**Symptomatic?**	**Action with Study Drug**	**Recommended Post-Discontinuation Follow Up**
Vasogenic edema	Yes or No	Discontinue study drug	Scan subject every 6 weeks after discontinuation of study drug to evaluate resolution/stabilization (stabilization is to be determined based on three follow-up scans).
Macrohemorrhage (≥10 mm)	Yes or No		
Superficial siderosis	Yes or No		
New microhemorrage > 4 (<10 mm)	Yes or No		
New microhemorrhage ≤ 4 (<10 mm)	Yes		
	No	Subject may stay on study until next safety assessment	Not applicable

Source: TRx-237-007 Imaging Charter (BioClinica)

6.2.2.4. QT Interval and ECG Abnormalities

On Day 1 Baseline, the pre-dose machine-read ECGs should be reviewed to confirm that there are no clinically significant abnormalities (see Exclusion Criterion No. 18) or deviations from the Screening ECG; the average of the three readings should be used for determination of QTcF and heart rate. Dosing may be held subject to eligibility review based on the local interpretation of triplicate ECGs by the investigator or the results of the central read. A cardiology consult should be sought prior to dosing if deemed necessary by the investigator.

Subsequent decisions with respect to the ECG may be made on the basis of the local reading, with cardiology consult if deemed appropriate by the investigator. In the event of concern based on the local interpretation, the ECG should be repeated, preferably in triplicate, and doses interrupted if warranted, pending central reading or local cardiology consult.

For subjects entering the study with controlled atrial fibrillation (*i.e.*, heart rate ≤ 84 bpm and appropriate anticoagulation), triplicate ECGs are mandatory at all visits after Visit 2. Data documented on the eCRF will be the average of the three readings. As ECG readings are done locally, a local cardiologist should be consulted for appropriate evaluation of ECGs if deemed necessary by the investigator.

For subjects entering the study with intraventricular conduction defects, a local cardiologist should be consulted for appropriate evaluation of the ECGs. In subjects with left bundle branch block, a cardiologist's evaluation is strongly recommended.

Treatment should be discontinued if the following treatment-emergent change occurs (on Day 1, based on the means of three pre- and post-dose recordings and single recordings at other time points confirmed by triplicate ECGs in the event of, in the opinion of the Investigator, clinically significant abnormality) without other explanatory cause:

- QTcF interval >500 msec: repeat ECGs should be obtained, in triplicate, within 2 weeks; if confirmed by the mean of the repeat ECGs, study drug should be discontinued pending further evaluation.

Other clinically significant changes in the ECG should be discussed with the medical monitor.

6.2.2.5. Other Safety Reasons Requiring Discontinuation

If renal concerns arise and the calculated creatinine clearance is < 50 mL/min, study drug should be discontinued.

6.3. Packaging, Labeling, and Storage

Double-blind study drug will be packaged, labeled, and distributed to study sites by a designated vendor.

Study drug supplied to subjects will be in individual aluminum blister cards containing a 1-week supply of drug (14 tablets). An appropriate number of blister cards will be contained within single cardboard cartons with sufficient supplies until the next scheduled study visit at which dispensing is planned (see Section 6.4). One additional 14-tablet blister card will be included to allow for delays in visit scheduling (or if original medication is lost or damaged).

Study drug package labels will be in compliance with applicable regulatory requirements and will include the statement “Keep out of reach of children”, the cautionary statement “Caution: New Drug – Limited by Federal (United States) law to investigational use” and/or “For clinical trial use only” as appropriate, as well as any other locally mandated statements. Labels will also be translated into the local language.

At a minimum, labels will also include the following information: the name and address of the Sponsor, the study code, a unique identifier, and appropriate contact information. In those jurisdictions where required, a re-test or expiry date will be included.

At the study site, study drug must be stored securely (*e.g.*, locked area) and at a temperature not more than 30°C. The temperature at which study drug is stored at the study site will be recorded daily. The packaging protects the study drug from light and moisture.

Subjects and caregivers should also be provided with information about required storage conditions. Study drug should be ingested immediately after removal from the blister card.

6.4. Dispensing

With the exception of the first dose administered in the clinic, all dosing will be on an outpatient basis. Study drug will be first dispensed to subjects/caregivers on Day 1 (Baseline, Visit 2) if it is determined that the subject can be discharged from the clinic; enough study drug will be dispensed to last the subject until Visit 4. This will occur after subjects have been observed at the site for at least 4 hours post-dose.

Thereafter, study drug will be dispensed beginning at the Week 8 visit (Visit 4) and at each subsequent study visit during the treatment period, except for the Week 52 (Visit 9) or early termination visit, after which administration of study drug under this protocol will cease. Subjects should bring all study medication, including the overage blister card(s), with them to every study visit for compliance assessment.

Subjects and caregivers will be provided with information about storage conditions and taking study drug, including instructions indicating that study drug must be used only as described in this protocol. They will also be informed that tablets should be swallowed whole and not broken, crushed, chewed, or dissolved in fluids prior to ingestion. If there are swallowing difficulties which prevent taking the medication as instructed, subjects should not be entered into the study. Subjects and caregivers should be warned that if the product is not swallowed immediately and is allowed to dissolve in the mouth, it will cause discoloration of teeth and oral mucosa. In the event of interruption or dose reduction, the subjects and caregivers will be provided with updated dosing instructions.

6.5. Compliance

At each post-Baseline visit during the treatment period, the number of tablets dispensed to the subject/caregiver will be recorded.

At the Week 4 visit (Visit 3) and at each study visit thereafter during the treatment period, the subject/caregiver will bring all unused study drug (provided at the previous study visit, or, in the case of the Week 8 visit [Visit 4], provided at Baseline [Visit 2]) to the study site. The number of tablets (all tablets remaining in unopened blister card plus any tablets that have been removed from blister card) will be counted and recorded by study site staff. Empty blister cards and packaging should also be returned by the subject/caregiver to the study site.

Subject compliance with prescribed study drug will also be assessed at each visit by questioning the subject and caregiver. Any apparent discrepancies between the number of tablets taken and the number of tablets which should have been taken since the last visit will be discussed with the subject and caregiver.

Compliance data, including dates of any dose deviations and/or interruptions, and any other pertinent information, will be recorded in the source documentation and on the appropriate field of the eCRF.

If during participation in the study a subject's compliance is determined to be < 80% or > 120% (taking into consideration allowable reductions in the number of tablets taken or dose interruptions), the subject and caregiver should be reeducated about taking study drug properly and the clinical research associate should be informed promptly. If compliance problems are recurrent, the investigator should inform the clinical research associate and contact the medical monitor to determine the course of action.

6.6. Accountability

The investigator or designee will keep a record of all study drug received, and of all study drug dispensed to and returned by subjects.

The investigator will ensure that the supplied study drug will be used only for administration to subjects enrolled in this study and for no other purpose.

The study drug accountability record will be checked by a study monitor at monitoring visits.

All unused and returned study drug will be either returned to the sponsor or designee or disposed of after study completion according to provided instructions.

7. ASSESSMENT OF EFFICACY

Efficacy will be measured at Screening/Baseline and at scheduled visits during the treatment period (see Table 4-1 in Section 4.4). Baseline efficacy assessments may be made on the day before randomization and dosing if necessary.

7.1. Raters

Efficacy instruments will be completed by assessors/raters who are not involved in the assessment of safety parameters that could result in unblinding, generally in the local language. Information regarding validation in each geographic region/language is on file with the Sponsor. Each time an efficacy instrument is administered, the name of the person administering the instrument and the date and time of administration will be recorded. For a given subject, raters should remain constant throughout the study if possible. Investigators and other raters will be trained and their proficiency in administering applicable efficacy instruments will be evaluated and documented. If there is to be a change in rater, the new rater cannot proceed until his or her proficiency in administering applicable efficacy instruments has been evaluated and documented. It is important that every effort be made to keep Rater 2 (who administers the Modified ADCS-CGIC) constant throughout the study. If a change is to be made, a per-subject handover must be arranged by way of at least one joint Rater 2 assessment involving both the new and the old Rater 2.

The following are additional rater requirements with respect to specific instruments:

- For the ACE-R and MMSE, the rater does not need to be blinded to other efficacy results. This rater will be termed Rater 1. This rater will also rate the additional items needed to compute ACE-III, the FRS, FAQ, and will be permitted to rate the UPDRS Parts II and III, C-SSRS and Serotonin Toxicity Assessment.
- The Modified ADCS-CGIC will be performed by an experienced qualified rater who remains blinded throughout the study to results from the ACE-R and all other efficacy results, as well as to all safety assessment results. The Modified ADCS-CGIC rater therefore will be a separate rater, termed Rater 2, who does not perform any other efficacy or safety ratings.

The baseline ACE-R and MMSE questionnaires will be assessed by an independent evaluator at Worldwide Clinical Trials (WCT). The screening assessments will be reviewed by WCT to ensure that subjects meet the inclusion criteria. As necessary, adjudication will occur for any subject where assessments fall outside of the inclusion criteria.

Results of efficacy assessments will be recorded in source documents in a manner such that required assessor blinding is maintained.

Efficacy assessments should be performed at approximately the same time of day throughout the study for a given subject, if possible. The sequence of subject and caregiver assessments is given in Table 7-1. The UPDRS Parts II and III (activities of daily living and motor function) are discussed in Section 8.8 as these evaluations will provide information pertinent to safety measures.

Table 7-1 Overview of Efficacy Assessments: Sequence of Testing by Rater

	Sequence	Subject		Caregiver	
		Rater 1	Rater 2	Rater 1	Rater 2
Screening and Week 52[1]	1	MMSE (~20 minutes)	-	-	-
Baseline and Weeks 16, 32, 52[1]	1	ACE-R (~30-40 minutes)	-	-	Modified ADCS-CGIC (~30 minutes)
	2	-	-	FRS (~20-25 minutes)	-
	3	-	Modified ADCS-CGIC (~30 minutes)	-	-
	4	-	-	FAQ (~10-15 minutes)	-

Note: The UPDRS Parts II and III (discussed in Section 8.8) will also provide information pertinent to efficacy measures and will be performed after the efficacy assessments; the UPDRS assessments require a total of approximately 30 minutes to complete.
[1] Or early termination

7.2. Instruments

Efficacy will be measured at Baseline (Visit 2) and after 16, 32, and 52 weeks on double-blind study drug (See Table 4-1 in Section 4.4). Baseline efficacy assessments may be made on the day

before randomization and dosing if necessary. Efficacy instruments include the following: ACE-R and ACE-III (cognition, the latter in an exploratory fashion), Modified ADCS-CGIC (global impression of change relative to Baseline), and FRS and FAQ (as functional measures). Modified ADCS-CGIC will also be rated at an earlier time point, after 8 weeks on study drug. The UPDRS Parts II and III (activities of daily living and motor function) will provide information pertinent to both efficacy and safety measures; the order in which this evaluation should be administered relative to the other efficacy scales is provided in this section, however the UPDRS is described in detail in Section 8.8.

MMSE will be performed separately at Screening; Baseline, Week 16 and Week 32 scores will be obtained in the context of the ACE-R. MMSE scores will be reported both separately and included in the ACE-R at Week 52 (end of study or early termination). Imaging-based efficacy assessments are discussed in Section 9.

Efficacy instruments will be completed by qualified, trained assessors/raters who have no access to safety assessment results (with the exception of the UPDRS Parts II and III, C-SSRS, and Serotonin Toxicity Assessment, which may be rated by Rater 1). Furthermore, the Modified ADCS-CGIC will be performed by a separate rater who remains blinded throughout the study to results from all other efficacy assessments. Results of efficacy assessments will be recorded in source documents in a manner such that required assessor blinding is maintained.

For a given subject, raters should remain constant throughout the study if possible. Each time an efficacy instrument is administered, the name of the person administering the instrument and the date and time of administration will be recorded.

Efficacy assessments should be performed at approximately the same time of day throughout the study for a given subject, if possible.

The order in which the efficacy instruments should be administered at each applicable visit is shown. As it may be difficult for subjects to cooperate with the approximate 90-minute duration required for the completion of all efficacy scales, at a minimum, every effort should be made to complete the primary efficacy ratings (ACE-R and FAQ). (Parenthetical reference is to whether the subject or caregiver is evaluated first for applicable scales for which both subject and caregiver are evaluated):

- Subject: ACE-R (and additional items from ACE-III), Modified ADCS-CGIC (caregiver first), UPDRS Parts II and III
- Caregiver: Modified ADCS-CGIC (caregiver first), FRS, FAQ, UPDRS Part II

7.2.1. Addenbrooke's Cognitive Examination

The Addenbrooke's Cognitive Examination (ACE) was developed as a brief (15- to 20-minute) test of cognitive function with the aim of early detection of dementia and differentiation between dementia subtypes (Mathuranath *et al.*, 2000; Larner, 2007). The ACE is divided into five cognitive domains: attention and orientation; memory; verbal fluency; language; and visuospatial function and includes all items from the MMSE (Folstein *et al.*, 1975). The ACE has been shown to be sensitive to decline in FTD over a 1- to 2-year period (Kipps *et al.*, 2008).

The ACE was revised to improve its administration and sensitivity and the revised instrument (ACE-R) has been shown to have very good psychometric properties (Mioshi *et al.*, 2006). The revised test measures performance in executive function (a core frontal lobe feature of the

disease) in addition to the five cognitive domains. The revised ACE-R is available in four parallel versions and is scored from 0 (impaired) to 100 (unimpaired), with 18 points for attention and orientation, 26 for memory, 14 for fluency, 26 for language, and 16 for visuospatial. It is partially validated or widely accepted in several languages, including English, German, and Spanish (Alexopoulos *et al*., 2010; Torralva *et al*., 2011). Although not formally validated, a French version is also in common use. A copy of the scale is provided in Section 24.4.

As noted above, the ACE-R incorporates the 30-point MMSE amongst its assessments. The MMSE (Folstein *et al.*, 1975) was originally developed to differentiate between psychiatric subjects with functional and organic conditions, to quantify the level of cognitive impairment, and to monitor changes over time. The MMSE subsequently has become a widely-used and extensively-validated cognitive test demonstrating satisfactory reliability, validity, and change sensitivity under a wide variety of conditions (Tombaugh and McIntyre, 1992). In an epidemiological study (Mukaetova-Ladinska *et al.*, 2000), *pre-mortem* MMSE scores have been correlated with *post-mortem* Braak stage (based on the spread of tau pathology through the brain). MMSE is also sensitive to decline in bvFTD (Knopman *et al*., 2008).

For purposes of study eligibility, the MMSE score will be performed separately at the Screening visit. For purposes of staging, the MMSE score will be reported separately (in addition to its inclusion in the ACE-R) at Week 52 (or early termination).

A further revised version of the ACE has been released as the ACE-III with the aim of excluding MMSE items for copyright reasons and is expected to provide the only ACE going forward (Neuroscience Research Australia, updated in November 2012). However, as ACE-R provides the only source of data available to determine expected rate of placebo decline, ACE-R is used as the primary outcome variable in the present study. Additional items will be tested in order to permit exploratory computation of ACE-III scores for future reference. The ACE-III is still scored from 0 to 100 based on the same five cognitive domains with the same total scores ascribed to each, but excluding certain MMSE items which are subject to copyright restriction. The main differences are in the Attention Domain (the subject is no longer asked to spell WORLD backwards), in the Language Domain (the three-stage command has been replaced by a short graded test of grammatical comprehension, two images in the “Naming” have been replaced, and some words and phrases in the “repetition” have been replaced), and in the Visuospatial Domain (the overlapping pentagons have been replaced by overlapping infinity symbols). A copy of the ACE-III scale is provided in Section 24.5. As the ACE-III and ACE-R are highly correlated (correlation coefficient 0.99; Hsieh *et al.*, 2013), in the few instances where only ACE-III may have been obtained at baseline, the change in total score (out of 100) from baseline ACE-III to subsequent ACE-R will be used to compute the change in ACE-R.

In order to allow computation of both ACE-R and ACE-III scores in this study, after completion of the ACE-R, the subjects will be asked to perform those additional tests which permit computation of the exploratory ACE-III outcome measure. The additional ACE-III items and the original version in the ACE-R are summarized below. Other minor changes intended for clarity in the instructions will not be implemented.

	ACE-R<br>(Version A; 2005)	ACE-III<br>(Version A; 2012)
Attention	**Orientation**<br>Ask: Which:<br>Building<br>Floor<br>Town<br>County<br>Country	**Attention**<br>Ask: Which:<br>No. / Floor<br>Street / Hospital<br>Town<br>County<br>Country
Language	**Language – Comprehension**<br>Show written instruction:<br>Close your eyes<br><br>[Score 0-1]<br><br>Three-stage command:<br>"Take the paper in your right hand. Fold the paper in half. Put the paper on the floor."<br><br>[Score 0-3]	**Language**<br>Place a pencil and a piece of paper in front of the subject. As a practice trial, ask the subject to "Pick up the pencil and then the paper." If incorrect, score 0 and do not continue further.<br><br>If the subject is correct on the practice trial, continue with the following three commands below.<br>• Ask the subject to "Place the paper on top of the pencil."<br>• Ask the subject to "Pick up the pencil but not the paper"<br>• Ask the subject to "Pass me the pencil after touching the paper"<br>Note: place the pencil and paper in front of the subject before each command.<br><br>[Score 0-3]
	**Language – Writing**<br>Ask the subject to make up a sentence and write it in the space below: Score 1 if sentence contains a subject and a verb (see guide for examples)<br><br>[Score 0-1]	**Language**<br>Ask the subject to write two (or more) complete sentences about his/her last holiday/weekend/Christmas. Write in complete sentences and do not use abbreviations. Give 1 point if there are two (or more) complete sentences about the one topic; and give another 1 point if grammar and spelling are correct.<br><br>[Score 0-2]
	**Language - Repetition**<br>Ask the subject to repeat: "hippopotamus," "eccentricity," "unintelligible," "statistician"<br><br>Score 2 if all correct; 1 if 3 correct; 0 if 2 or less.<br><br>Ask the subject to repeat: "Above, beyond and below"<br><br>Ask the subject to repeat: "No ifs, ands or buts"	**Language**<br>Ask the subject to repeat: "caterpillar," "eccentricity," "unintelligible," "statistician"<br><br>Score 2 if all are correct; 1 if 3 are correct; and score 0 if 2 or less are correct.<br><br>Ask the subject to repeat: "All that glitters is not gold"<br><br>Ask the subject to repeat: "A stitch in time saves nine"
	**Language - Naming**<br>Ask the subject to name the following	**Language**<br>Ask the subject to name the following

	ACE-R (Version A; 2005)	ACE-III (Version A; 2012)
	pictures (pictures are of:<br><br>pencil<br>watch<br>kangaroo<br>penguin<br>anchor<br>camel<br>harp<br>rhinoceros<br>barrel<br>crown<br>alligator<br>accordion<br><br>[Score 0-2] pencil + watch<br>[Score 0-10]	pictures (pictures are of:<br><br>spoon<br>book<br>kangaroo<br>penguin<br>anchor<br>camel<br>harp<br>rhinoceros<br>barrel<br>crown<br>alligator<br>accordion<br><br>[Score 0-12]
Visuospatial	**Visuospatial Abilities**<br>Overlapping pentagons: Ask the subject to copy this diagram:<br><br>(image)<br><br>(remaining section identical)	**Visuospatial Abilities**<br>Infinity Diagram: Ask the subject to copy this diagram:<br><br>(image)<br><br>(remaining section identical)

In computing the ACE-III score, the following ACE-R item will not be included:

	ACE-R (Version A; 2005)	ACE-III (Version A; 2012)
Attention	**Attention & Concentration**<br>"Serial 7s"<br>Ask: Could you please spell WORLD for me? Then ask him/her to spell it backwards.	**Attention**<br>"Serial 7s"<br>Not done

7.2.2. Functional Activities Questionnaire (FAQ)

The Functional Activities Questionnaire (FAQ) was developed to provide operational descriptions of various levels of function, independent of education, socio-economic status and intelligence (Pfeffer *et al.*, 1982). This assessment is based on interview with the informant/caregiver, and is not a patient interview. The informant rates the subject on 10 complex, higher-order activities, including money management, cooking, shopping, recreation, awareness and memory, and ability to use transportation. The FAQ takes only 10 minutes to complete. For each activity, four levels ranging from dependence (scored 0) to independence (scored 3) are specified. For activities not normally undertaken by the person, the informant must specify whether the person would be unable to undertake the task if required (scored 1) or could do so if required (0). The total score is the sum of individual item scores; higher scores reflect greater dependency. Sum scores range from 0-30. The FAQ has been shown to be sensitive to change in FTD (Knopman *et al.*, 2008; Boxer *et al.*, 2013).

7.2.3. Modified Alzheimer's Disease Cooperative Study - Clinical Global Impression of Change (Modified ADCS-CGIC)

The fundamental construct that underpins the validity of global impressions of change is the skilled clinician's ability to detect worthwhile clinical change as opposed to clinically irrelevant change. These scales have been used extensively in clinical trials and several different versions are available. The Alzheimer Disease Cooperative Study - Clinical Global Impression of Change (ADCS-CGIC) was developed for the National Institute of Aging's ADCS Instrument Development Project and allows a clinician to rate clinically relevant global changes using an organized but unstructured format. It translates two domains (cognitive and functional) into a global assessment. The instrument has been shown to have good reliability, validity and change sensitivity (Schneider *et al.*, 1997). The ADCS-CGIC has been used in randomized controlled trials evaluating efficacy of cholinesterase inhibitor drugs and has been shown to be sensitive to their effects.

A modified version of the ADCS-CGIC developed to assess the relevant domains of behavior and cognition in FTD subjects will be used, which has been shown to be sensitive to change over 1 year in a simulated clinical trial (Knopman *et al.*, 2008). At each time point, the caregiver is evaluated first, and then the subject evaluation is performed. It is estimated that the assessment will require approximately 20 minutes of subject and 20 minutes of caregiver time. A copy of the scale is provided in Section 24.6.

At Baseline, a clinician blinded to other aspects of the study rates 15 areas in three domains based on an interview with the subject and caregiver, to be documented in notes in sufficient detail to serve as a reference for post-Baseline assessments. The three domains are:

- mental and cognitive state
- behavior
- social and daily functioning

This process is repeated at follow-up (Weeks 16, 32, and 52 [or early termination]) and subsequently a change rating is made on a 7-point Likert scale. The modified ADCS-CGIC should only be referred to Baseline and not to the rest of the visits. That is, the only change that is scored at any given visit is the change from Baseline, and not from any other preceding visit.

In order to ensure that change score is based on the comparison between the Baseline visit and the current visit, the Rater 2 will only have access to the Baseline assessment notes for the purpose of rating subsequent change. Rater 2 will not have access to notes from any intermediate visits other than the Baseline visit at any subsequent assessment. It is therefore particularly important that every effort be made for the Rater 2 to remain constant throughout the study. If a change is to be made, a per-subject handover must be arranged by way of at least one joint Rater 2 assessment involving both the new and the old Rater 2.

The change is to be scored as:

1 = marked improvement
2 = moderate improvement
3 = minimal improvement
4 = no change

5 = minimal worsening
6 = moderate worsening
7 = marked worsening

7.2.4. Frontotemporal Dementia Rating Scale (FRS)

The Frontotemporal Dementia Rating Scale (FRS) was developed from the Cambridge Behavioural Inventory and the Disability Assessment in Dementia as a tool to stage dementia severity in FTD (Mioshi *et al.*, 2010). This assessment is based on interview with the informant/caregiver, and is not a patient interview. The FRS is a 30-item questionnaire assessing the following areas: behavior, outing and shopping; household chores and telephone; finances and correspondence; medications; meal preparation and eating; and self-care and mobility. It is rated with a caregiver and takes approximately 20 minutes to complete. A percentage score is derived with higher scores representing less severe dementia. The FRS is also able to identify six severity stages in FTD (very mild to profound) and has been shown to be sensitive to decline in bvFTD. In addition to English, it is available in French and Spanish.

8. ASSESSMENT OF SAFETY

Safety will be assessed over time by means of adverse events (AEs), laboratory tests of blood (*e.g.*, hematology and chemistry panels, Vitamin B_{12} levels, folate levels, and TSH), urine (*e.g.*, urinalysis), methemoglobin and oxygen saturation measurement, vital sign measurement, ECGs, physical and neurological examinations, serotonin toxicity assessments, and assessment of the potential for suicide or self-harm (C-SSRS).

Assessments to be performed in the clinic are described in the subsections that follow. At intervening times (after 12, 20, and 28 weeks ± 7 days), caregivers of subjects are to be contacted by telephone and an unscheduled visit is to take place if needed in response to a safety concern. Similarly, an unscheduled visit may become necessary (with in-clinic monitoring) if a drug with serotonergic potential is to be initiated (see Section 4.7.2). Imaging-based safety assessments are discussed in Section 9.

Over the course of the entire study (including the post-treatment follow-up visit), the total volume of blood collected from each subject will not exceed approximately 200 mL.

8.1. Adverse Events

An AE is any unfavorable or unintended sign, symptom, or disease, whether or not considered related to the study treatment. This also includes events resulting from medication error or inappropriate use. AE recording will begin at the time the ICF is signed. Thereafter, AEs will be ascertained by asking the subject (and caregiver) how the subject has been since the last visit. A clinical abnormality, laboratory test value abnormality, or imaging abnormality that the investigator deems to be clinically significant should be recorded as an AE.

Every attempt should be made to describe the AE in terms of a diagnosis. Once a clear diagnosis has been made, individual signs and symptoms shall not be recorded unless they represent atypical or extreme manifestations of the diagnosis, in which case they should be reported as separate events. Events leading up to a diagnosis should be retained. If a clear diagnosis cannot be established, each sign and symptom must be recorded individually. Consistent with this, signs or symptoms indicated by the Serotonin Toxicity (Syndrome) Diagnostic Interview and Rating

Guide (In-Clinic) (Section 24.7) or Serotonin Toxicity Telephone Assessment (Section 24.8) that are judged to represent an AE should be recorded as such. However, if any of the criteria for serotonin syndrome are met (see Serotonin Syndrome Worksheet in Section 24.7), the AE is to be recorded as "Serotonin Syndrome", rather than the individual terms.

All AEs must be fully recorded in the source documents and in the eCRF, regardless of whether or not the event is considered related to study drug.

For all AEs, the investigator must pursue and obtain information adequate both to determine the outcome of the AE and to assess whether it meets the criteria for classification as an AE of special interest (Section 8.1.3) or as a Serious Adverse Event (SAE) requiring immediate notification (See Sections 8.1.4 and 8.1.5).

Follow-up of an AE, even after the final dose of study drug, is required if the AE or its sequelae persist. Follow-up is required, including beyond the scheduled final off-treatment visit if needed, until the AE or its sequelae resolve or stabilize at a level acceptable to the investigator.

For each AE, information recorded will include the following: the date when the AE started, the date when the AE stopped (or whether it remained ongoing), the intensity of the AE (see Section 8.1.1), the relationship of the AE to study drug (see Section 8.1.2), action taken with regard to study drug (none, dose reduction, interrupted, or discontinued), other drug therapy (no change, new medication, altered medication, or both of the latter), outcome, and whether or not the AE was considered an SAE (see Section 8.1.4). On Day 1, a separate AE form will be used, and will include time of dose and time of AE onset.

8.1.1. Intensity

The intensity (severity) of each AE will be assessed by the investigator and graded as mild, moderate, or severe, as follows:

- Mild
  An AE that is easily tolerated by the subject, causes minimal discomfort and does not interfere with everyday activities
- Moderate
  An AE that is sufficiently discomforting to interfere with normal everyday activities
- Severe
  An AE that prevents normal everyday activities

8.1.2. Relationship to Study Drug

The investigator will make a judgment considering whether or not, in his or her opinion, each AE is related to the study drug according to classifications described here. However, even if the investigator feels that there is no relationship to the study drug, the AE should be reported nevertheless. For each AE, the relationship or association (causality) of the AE to study drug will be assessed by the investigator and characterized as not related, unlikely related, possibly related, or related as follows:

- Not related
  If there is a confirmed cause of the AE (other medical condition, other therapy) which does not involve the study drug

- Unlikely related
  If the temporal association between the AE and the study drug is such that the AE is not likely to be related to the study drug
- Possibly related
  If the AE shows a reasonable temporal association to study drug administration but could be due to the subject's clinical state or other therapies administered
- Related
  If the AE shows a reasonable temporal association to study drug administration and cannot be explained by the known characteristics of the subject's clinical state

8.1.3. Adverse Events of Special Interest

Several adverse events are of special interest because additional steps are to be taken by the investigator to assess and manage them. The medical monitor shall be informed (either directly by the laboratory, the core imaging laboratory or the site, depending on the nature of the event). AESIs include:

- Methemoglobin values > 3.5% (confirmed on repeat result; see Section 8.4), signs or symptoms consistent with methemoglobinemia or hemolytic anemia, or observation of Heinz bodies. For purposes of this study, only post-randomization methemoglobin values meeting these criteria would be considered an AESI and whether or not this is considered an SAE depends on medical and scientific judgment.
- A case meeting any one of the four criteria for serotonin syndrome outlined in Section 24.7 is a medically significant event and thus will be reported and handled as an SAE (see Section 8.1.6)
- Any possible case of ARIA (see Section 6.2.2.3) is a medically significant event and thus will be reported to the Sponsor and the procedure will be as for an SAE (see Section 8.1.6); asymptomatic subjects with ≤4 new microhemorrhages need not be handled as an SAE. All investigators should be notified of the possible occurrence of ARIA, its imaging manifestations, and the clinical signs and symptoms that may accompany this phenomenon. They should also be told of the measures to be taken should ARIA occur.

See Section 6.2.2 for additional details regarding assessment and dose interruption, reduction, or discontinuation in response to any of these events.

Each AESI should be recorded as for any other adverse event.

8.1.4. Serious Adverse Events

An SAE is defined as any event that:

- Results in death (including suicide)
- Is life-threatening
- Results in inpatient hospitalization or prolongation of existing inpatient hospitalization
  - Planned admissions for respite care are not to be considered an SAE (the medical monitor should be contacted for confirmation regarding whether or not an admission for respite care should be considered planned or unplanned). Unplanned admissions

for respite care will constitute an SAE unless it is as a result of caregiver needs that are independent of the subject's condition.

- o An admission or prolongation of existing hospitalization because the subject does not want to be discharged, or because the caregiver is unable or unwilling to care for the subject, is not to be considered an SAE.
- o Admissions to a hospital that were planned or anticipated before the start of the study for an unrelated pre-existing medical condition are not to be considered an SAE.

- Results in persistent or significant disability or incapacity
- Is a congenital anomaly or birth defect
- Other emergent medically significant event that requires immediate medical or surgical intervention. For purposes of this study, the following will be considered medically significant events and thus reported to the Sponsor and the procedure will be as for SAEs whether or not they meet any other criteria for being an SAE:
    - o Any post-randomization case of suicidal ideation, intent, or action or self-injurious behavior (see Section 8.10)
    - o Possible post-randomization cases of serotonin toxicity and ARIA (see Section 8.1.3)

Medical and scientific judgment should be exercised in deciding whether an event is serious and whether expedited reporting is appropriate in other situations, such as important medical events that may not be immediately life-threatening or result in death or hospitalization but may jeopardize the subject or may require intervention to prevent one of the outcomes listed in the definitions above.

All SAEs must be reported on the eCRF. An assessment should be made by the investigator of whether the event is study drug-related, *i.e.*, is ‘causally’ related to the study drug.

8.1.5. Pregnancy

Pregnancy is to be considered an immediately reportable event. This includes pregnancy of a female subject or a female sexual partner of a male subject.

Subjects who become pregnant during the clinical study should discontinue study drug immediately and contact the investigator.

Subjects should be instructed to notify the investigator of a pregnancy either during the treatment period of the study or within 3 months after the last dose of study drug. Whenever possible, a pregnancy should be followed to term, any premature terminations reported, and the status of the mother and child should be reported to the Sponsor after delivery.

Although the pregnancy is not considered to be an AE or SAE, any pregnancy complications should be recorded as AEs or SAEs (if applicable). Any pregnancy should be followed through delivery for the observation of any SAE. Therefore, regardless of whether or not a pregnancy is actually considered an SAE, a pregnancy form should be completed for all pregnancies. Pregnancies should initially be reported in the Pregnancy Notification Form, Part I and sent by e-mail to the following address:

drugsafety@wwctrials.com.

When the outcome of the pregnancy is known, site personnel will complete the Pregnancy Notification Form, Part II and e-mail it to the following address:

drugsafety@wwctrials.com.

All data related to pregnancy, pregnancy outcome, and SAE associated with pregnancy will be recorded in a safety database maintained by personnel responsible for pharmacovigilance at Worldwide Clinical Trials.

8.1.6. Reporting Requirements and Timeframes

The requirements for reporting SAEs and pregnancies are described below. A Safety Management Plan will be implemented to further describe and document the process for safety reporting.

8.1.6.1. Investigator Reporting of SAEs and Pregnancy to Sponsor

If any of the adverse events are SAEs as defined by this protocol (see Section 8.1.4) or a pregnancy (see Section 8.1.5), special procedures will be followed. All such events will be reported to the Sponsor designee, Worldwide Clinical Trials, immediately (and not exceeding 24 hours following knowledge of the event) and followed by follow-up reports as soon as possible, whether or not the events are deemed study drug-related.

Serious Adverse Events must be reported by entering the SAE information in the AE/SAE section of the Electronic Data Capture (EDC) system. The information provided in the EDC system should be as complete as possible, but must contain the following minimum fields:

- Subject number
- Brief description of the SAE (diagnosis or signs/symptoms)
- Serious criteria
- Causality assessment
- Assessment of the intensity of the event

WCT Drug Safety will receive notification of the initial SAE *via* an e-mail alert generated from the EDC system. In the event of any temporary disruption of the EDC system, an alternative SAE reporting mechanism will be available to site personnel; in this instance, a paper SAE Report Form will be available. Site personnel will complete the paper SAE report form, scan and e-mail it within 24 hours to the following address:

drugsafety@wwctrials.com

Site personnel must complete the AE/SAE section with the SAE information as soon as the EDC system becomes available. Serious adverse events that are ongoing should be followed until resolved or stabilized to a level acceptable to the investigator.

The investigator is obliged to provide additional information as requested by the medical monitor. In general, this will include a description of the event in sufficient detail to allow for a complete medical assessment of the case and independent determination of possible causality. In the case of a subject death, a summary of available autopsy findings, if performed, must be submitted as soon as possible to the contract research organization. However, any supporting

information provided should not reveal a subject's identity beyond the agreed study identifier. The investigator should ensure that information reported is accurate and consistent.

Information not available at the time of the initial report (*e.g.*, an end date for the AE, laboratory values received after the report, or hospital discharge summary) must be documented on a follow-up form. All follow-up information must be reported in the same timelines as initial information.

Any SAEs considered related to the study drug and discovered by the investigator at any interval after the study must also be reported to the Sponsor within 24 hours following knowledge of the event.

8.1.6.2. Sponsor Reporting of SUSARs to Regulatory Authorities

SUSARs are adverse events that are believed to be related to an investigational medicinal product and are both unexpected (*i.e.*, the nature or severity is not expected from the information provided in the Investigator's Brochure) and serious. As stated in the EU 'CT-3' Communication from the Commission (2011/C 172/01) and the US Code of Federal Regulations (21 CFR 312.32), for there to be a reasonable possibility of a causal relationship between the event and study drug there must be facts (evidence) or arguments to suggest a causal relationship. Final assessment of expectedness for purposes of regulatory reporting is the responsibility of the Sponsor.

It is the responsibility of the Sponsor to determine whether a reported SAE fits the classification of a SUSAR and to notify the investigator of the decision as soon as possible.

Requirements and unblinding procedures for SUSAR reporting are described below. All SUSAR reporting, whether determined following unblinding during study conduct or apparent only after the study has ended, will adhere to European Directives 2001/20/EC, 21 CFR 312.32 of the U.S. Code of Federal Regulations, Health Canada Food and Drug Regulation C.05.014, and other regions as applicable.

8.1.6.2.1. Unblinding Treatment Allocation

Generally, only SUSARs for which the treatment allocation of the subject is unblinded should be reported by the Sponsor to the pertinent regulatory authorities.

When an event may be a SUSAR, the blind should be broken only for that specific subject. The blind should be maintained for individuals responsible for the ongoing conduct of the study (*e.g.*, management, monitors and investigators) and those responsible for data analysis and interpretation of results at the conclusion of the study (*e.g.*, biometrics personnel).

Unblinded information should only be accessible to those who need to be involved in the safety reporting to pertinent regulatory authorities, independent ethics committees/independent review boards (IECs/IRBs) and DSMBs, or individuals performing ongoing safety evaluations during the trial.

8.1.6.2.2. Fatal or Life-threatening SUSARs

It is the responsibility of the Sponsor to report fatal or life-threatening SUSARs to the pertinent regulatory authorities as soon as possible, but no later than 7 calendar days (or earlier if locally mandated). These will be reported to the DSMB in parallel.

8.1.6.2.3. Other SUSARs

It is the responsibility of the Sponsor to report other SUSARs to the pertinent regulatory authorities as soon as possible, but no later than 15 calendar days (or earlier if locally mandated). These will be reported to the DSMB in parallel.

8.1.6.3. Reporting to IECs/IRBs

The IEC/IRB will be notified of any SUSARs according to local regulations and within the designated timeframe.

The investigator (or prospectively agreed to designee) is required to notify the IEC/IRB of any fatal SUSAR as soon as possible but no later than 7 calendar days after he/she first became aware of the reaction (or earlier if locally mandated).

The investigator (or prospectively agreed to designee) is required to notify the IEC/IRB of any other SUSAR as soon as possible but no later than 15 calendar days after he/she first became aware of the reaction (or earlier if locally mandated).

8.2. Urgent Safety Measures

The Sponsor and investigator may take appropriate urgent safety measures in order to protect the subjects of the clinical study against any immediate hazard to their health or safety.

The Sponsor and the Medical Monitor or designated deputy will be notified of any urgent safety measures taken by the investigator or qualified designee and advised of their responsibility to notify the licensing authority. The investigator or qualified designee will notify the IEC/IRB.

If these measures are taken, the Sponsor or investigator shall immediately give written notice to the pertinent regulatory authorities and IEC/IRB of the measures taken and the circumstances giving rise to those measures. In any event, the written notice shall be no later than 7 days from the date the measures are taken.

8.3. Clinical Laboratory Tests

Scheduled blood and urine samples for hematology and chemistry panels, Vitamin B_{12} levels, folate levels, and urinalysis will be obtained at each visit during the study, including Screening, Baseline (pre-dose), and approximately 4, 8, 16, 24, 32, 42, and 52 weeks after Baseline (or upon early termination). Blood for measurement of TSH will be obtained at Screening and after 24 and 52 weeks (or upon early termination), with a thyroid hormone panel obtained in the event of abnormality (and further follow up as needed). Clinical laboratory testing will be repeated at the post-treatment follow-up visit if applicable (Visit 10).

A trained and authorized person will collect biological (blood and urine) samples from the subject. Blood samples will be collected by venipuncture from a suitable vein.

Kits with supplies for the collection of blood and urine samples will be provided to each study site before the study initiation visit. The kits will be labeled to identify the clinical study, and will include specimen labels, pre-printed laboratory requisition forms, all supplies needed for specimen collection and shipping, instructions for collection and preparation of specimens, and pre-printed forms to expedite shipment.

A protocol-specific laboratory manual will be provided to each study site. The laboratory manual will include contact details, lists of the contents of collection kits, shipment schedule of

collection kits, and detailed guidelines and recommendations for completing laboratory requisition and for specimen collection, preparation, storage, and transportation.

A central laboratory will process all scheduled laboratory blood samples unless otherwise noted. Data will be transferred electronically for inclusion in the database. Investigators are to enter the results of any testing performed at local laboratories (and the corresponding normal ranges) into the eCRF.

8.3.1. Serum Chemistry

The blood volume for each chemistry panel will be approximately 3.5 mL. Blood samples collected for serum chemistry panels are to be destroyed 1 week after testing has been completed. The chemistry panel will include the following analytes:

Sodium	Gamma-glutamyl transpeptidase (GGT)
Potassium	Alanine transaminase (ALT)
Calcium	Aspartate transaminase (AST)
Albumin	Glucose (random, not fasted)
Total protein	Creatinine*
Blood urea nitrogen, urea	Phosphate
Total and direct (conjugated) bilirubin (with indirect bilirubin calculated as the difference)	Triglycerides
Alkaline phosphatase	Cholesterol (total)
Lactate dehydrogenase (LDH)	Uric acid
Creatine kinase (CK)	Chloride

* Creatinine clearance is to be estimated by the central laboratory using the Cockcroft-Gault equation

8.3.2. Hematology

The blood volume for each hematology panel will be approximately 2.0 mL. Blood samples collected for hematology panels are to be destroyed 3 days after testing has been completed. The hematology panel will include the following analytes:

Hematocrit	White blood cells (WBC) and absolute and percent differential (neutrophils, lymphocytes, monocytes, eosinophils, basophils); any abnormal cells will be noted
Hemoglobin	
Mean corpuscular hemoglobin (MCH)	
Mean corpuscular hemoglobin concentration (MCHC)	
Red blood cells (RBC) and any immature forms	Platelet count
RBC morphology	Mean cell volume (MCV)
Reticulocyte count and percent	

If the Investigator is concerned on the basis of significant hematological abnormalities that Heinz bodies may be present, a blood sample may be sent to the local laboratory for assessment and screening of Heinz bodies; blood samples should be as fresh as possible at the time of slide preparation. If Heinz bodies are present, study medication must be stopped (see Section 6.2.2.1)

and hematological indices monitored until this resolves. Another blood sample will be obtained within 1 week and the percent of erythrocytes with Heinz bodies will be recorded if Heinz body determination is considered to be indicated by the Investigator.

8.3.3. Urinalysis

Urine samples for urinalysis will be analyzed by the central laboratory using the iQ 200 Systems by IRIS (International Remote Imaging Systems, Inc.). The instrument combines all analyses into one system. Specific gravity is determined by refractometry. Flow microscopy is used to measure RBCs, WBCs, WBC clumps, hyaline casts, unclassified casts, squamous epithelial cells, non-squamous epithelial cells, bacteria, yeast, crystals, mucus, and sperm. A dual wavelength (to adjust for potential colorimetric interference) dipstick will be used for the following chemistries: pH, glucose, ketones, protein, bilirubin, blood, nitrites, and urobilinogen. Color and turbidity will also be reported. These samples will be destroyed 1 week after testing.

Because MT potentially interferes with colorimetric measurements, extra samples will be prepared. If ≥ 2+ protein is reported by dipstick or results cannot be reported due to interference, an unpreserved sample will be analyzed for microalbumin by nephelometry; these samples will be discarded within 2 weeks of receipt. An additional sample will be stored frozen at ≤ -20°C if needed for further analysis to be performed as needed (*e.g.*, sodium concentration and/or renal biomarkers). These frozen samples will be stored for the period of the study, expected to be approximately 28 months.

8.3.4. Other Laboratory Tests

Vitamin B_{12} and folate will be analyzed by the central laboratory using Beckman Coulter Access® Immunoassay Systems which are based on competitive-binding immunoenzymatic assays. Folate levels can be measured accurately over the analytical range of 1.0 to 24.8 ng/mL (2.27 to 56.2 nmol/L) and Vitamin B_{12} over the range of 50 to 1500 pg/mL (37 to 1107 pmol/L). Plasma levels are quantitated by chemiluminescence. Blood samples collected for measurement of Vitamin B_{12} and folate will be destroyed 1 week after testing has been completed.

Haptoglobin will be measured at Screening (Visit 1) only[5]; separate blood samples will be obtained for this purpose and will be destroyed 1 week after testing has been completed.

A serum pregnancy test for qualitative testing for the beta subunit of human chorionic gonadotropin (β-hCG) will be obtained for all women of childbearing potential at Screening, Baseline (pre-dose), and each subsequent study visit (or upon early termination), including the 4-week post-treatment follow-up visit if applicable (Visit 10). In the event of a borderline result, testing will be repeated and if the result is still borderline, quantitative testing for β-hCG will be performed.

TSH will be measured at Screening and after 24 and 52 weeks on treatment (or upon early termination); blood samples collected for this purpose will be destroyed 1 week after testing has been completed. In the event of elevation, free triiodothyronine (T_3) and thyroxine (T_4) are to be measured at the next visit. If abnormal, the subject is to be further evaluated (including possibly by measuring thyroxine-binding globulin [TBG] and T_3 uptake; such testing should be undertaken at an unscheduled visit). T_3 uptake samples should be shipped ambient to the central

[5] Haptoglobin may also be measured in response to signs or symptoms of methemoglobinemia or hemolytic anemia.

laboratory; samples for TBG measurement should be shipped frozen (-20 ºC) to the central laboratory and will be forwarded to ARUP Laboratories in Salt Lake City, UT, USA. Blood samples collected for measurement of TBG and T_3 uptake will be destroyed after a storage period of 3 months from the time of sample collection at the study site.

Subjects are to be screened for G6PD deficiency at Screening (Visit 1) using a commercially available kit supplied to the sites for on-site testing, or by using a suitable laboratory. Blood samples obtained for measurement of G6PD should be immediately discarded after testing has been completed.

8.3.5. Review of Laboratory Results

Laboratory results from each study visit will be assessed in a timely manner. The investigator or an authorized physician sub-investigator must interpret the laboratory findings and confirm their review.

The clinical significance of all laboratory values which are outside the laboratory normal reference range should be noted and commented upon by the investigator.

Abnormal values which are considered by the investigator to be clinically significant, taking the age of the subject into account, should be documented as an AE, unless accounted for by a pre-existing medical condition detailed in the subject's medical history. The diagnosis associated with a clinically significant laboratory abnormality generally should be recorded as the underlying abnormality or diagnosis (*e.g.*, renal insufficiency) if there is sufficient overall information to permit a diagnosis to be made. Otherwise, the observed deviation in the laboratory result should be recorded (*e.g.*, elevated creatinine).

The investigator must review and assess laboratory results that represent potential AESIs, *i.e.*, methemoglobin and other hematology panel results including hemoglobin, RBC, reticulocytes, or the appearance of Heinz bodies in the RBCs. If any of these suggest a potential increase in methemoglobin levels, or hemolytic anemia, treatment interruption should be considered (see Section 6.2.2.1). In the event of borderline or deficient folate values not corrected by folate replacement, subjects should be referred to their primary care physician for evaluation and correction or the medical monitor consulted. In the event of borderline or deficient Vitamin B_{12} values (see Section 4.7.8), subjects should be referred to their primary care physician for evaluation and correction or the medical monitor consulted.

Additional tests and other evaluations required to establish the significance or etiology of an abnormal result or to monitor the course of an AE may be obtained from the central laboratory. If a local laboratory needs to be used in some circumstances, the data and corresponding normal ranges will be recorded in the eCRF, entered directly from a laboratory slip. When clinically indicated, the medical monitor should be informed in such circumstances. In particular, if a clinically significant abnormal result is observed that is not resolved by the following visit, repeated tests should be performed until resolution of the abnormality.

8.4. Pulse Co-oximetry

Methemoglobin and oxygen saturation will be measured at Screening, Baseline (within 1 hour prior to dosing and approximately 2.5 hours post-dose), and every subsequent visit thereafter (approximately 4, 8, 16, 24, 32, 42, and 52 weeks after Baseline, or upon early termination). Testing will be repeated at the post-treatment follow-up visit if applicable (Visit 10).

The measurements will be performed using a handheld pulse co-oximeter (model Rad-57) provided by the Masimo Corporation, Irvine CA (FDA 510(k) Number – K042536). The device uses variable wavelengths to measure methemoglobin. Because this is a light-emitting device, it should be left on only so long as needed to obtain the recording(s) (20 seconds is recommended).

Initially, the device is to be applied to the index, middle, or ring fingers. Any methemoglobin values recorded as greater than 2.0% should be confirmed; two immediate repeat measurements should be obtained such that a total of three methemoglobin readings are obtained on three different fingers at a single visit. The mean of the three readings will be calculated automatically in the clinical database and used as the basis for safety monitoring decisions.

Additional monitoring is necessary for any mean methemoglobin values recorded as greater than 3.5% (see Section 6.2.2.1).

All individual and calculated mean readings will be captured in the clinical database and included in the eCRF.

In a validation study (Barker *et al.*, 2006) performed in comparison to standard co-oximetry, methemoglobin was reported with a bias of 0 and a precision of ±0.45%. Consideration was initially given to using standard co-oximetry to perform the methemoglobin measurement; however, in comparison to standard co-oximetry for which interference is well-documented (Gourlain *et al.*, 1997), pulse co-oximetry allows for less interference from other substances in the blood. The reading is performed quickly, and eliminates the issue of processing whole blood samples as well as any problems with sample degradation.

8.5. Vital Sign Measurements

In the clinic, measurements of the vital signs described below will be made by a trained and authorized person.

8.5.1. Temperature and Respiratory Rate

Temperature and respiratory rate will be recorded at Screening, Baseline (Visit 2), and every study visit thereafter (approximately 4, 8, 16, 24, 32, 42, and 52 weeks after Baseline), including early termination and any follow-up visit (if applicable). Oral (sublingual) temperature measurements are preferable; aural temperature is an acceptable alternative (this alternate means of measuring temperature is to be recorded in the eCRF).

At Visit 2 (Day 1), temperature and respiratory rate will be recorded within 1 hour prior to dosing and hourly while subjects are in the clinic, *i.e.*, for at least 4 hours.

Following discharge, subjects who are receiving serotonergic medications and their caregivers will be given a thermometer for continued measurement at home. Temperature measurements are to be performed three times a day (in the morning, afternoon, and evening) until approximately 72 hours after the first dose. Each measurement will be recorded in a diary provided by the site, to be returned to the site at Visit 3 (after 4 weeks). Given that caregivers will be contacted by telephone 5-7, >7-14, >14-24, 44-52, and 68-76 hours after the first dose of study drug (with a minimum of 1 hour between contacts) and queried for the presence of signs and symptoms of serotonin toxicity in the subject, they will be instructed to have the diary available during these telephone contacts.

Respiratory rate will be observed over 15 seconds and recorded in breaths/minute.

8.5.2. Blood Pressure and Pulse

Blood pressure and pulse will be recorded at Screening and Baseline and at each study visit, *i.e.*, after 4, 8, 16, 24, 32, 42, and 52 weeks (or at the final study visit) and at the follow-up visit if applicable (Visit 10). In addition, at Visit 2 (Day 1), measurements will be recorded within 1 hour prior to dosing and approximately 2 hours post dose. If authorized by the medical monitor, vital sign measurements may be made locally at the non-efficacy visits occurring after 4, 8, 24, and 42 weeks for subjects who live at great distance from the study site. Arrangements will be made to enter such data into the eCRF.

At Screening and on Day 1, blood pressure and pulse shall be measured with subjects in a seated position for at least 5 minutes, and again 2 minutes after standing; thereafter, they will be measured with subjects in the seated position only (for at least 5 minutes).

A manual or automated sphygmomanometer will be used to measure systolic and diastolic blood pressure. Blood pressure results will be recorded in mmHg. Pulse rate will be measured in the radial artery for 60 seconds and will be recorded as beats/minute. If possible, blood pressure and pulse rate preferably should be measured in the same arm at each visit.

8.5.3. Weight and Height

Weight will be measured at Screening (Visit 1), Baseline (Visit 2), Week 8, Week 16, Week 24, Week 32, Week 42, and Week 52 (or early termination). If applicable, weight will also be recorded at the off-treatment follow-up visit (Visit 10). Weight will be measured while the subject is clothed with shoes off and recorded in kilograms (kg).

Height will be measured in centimeters at Screening (Visit 1).

8.6. Electrocardiography

At Screening (Visit 1) 12-lead electrocardiogram (ECG) recordings will be obtained in triplicate (within an approximate 2- to 5- minute interval) with the subject in a supine position, suitably rested (for at least 5 minutes). At Baseline (Visit 2), 12-lead ECGs will be performed before administration of the first dose of study drug; the results should be reviewed by the investigator to confirm that the subject may be dosed (see Exclusion Criterion No. 18). The ECGs are to be repeated approximately 3 hours post-dose. At both of these Visit 2 time points, ECG measurement will include triplicate recordings (within an approximate 2- to 5-minute interval). Data entered in the eCRFs will be the average of the three readings. The confirmation of the decision regarding the subject’s eligibility for the study is to be based on the local read. A local cardiology consult should be sought if the investigator deems it necessary. For evaluation of subjects with left bundle branch block, a cardiology consult is strongly recommended. In circumstances where a subsequent central reading reports a significantly different QTcF compared with the local read, the discrepancy and further evaluation is to be addressed by medical monitors on a case to case basis.

Subsequently, 12-lead ECGs will be obtained at each study visit, *i.e.*, after 4, 8, 16, 24, 32, 42, and 52 weeks (or upon early termination) and at follow-up if applicable (Visit 10); these ECG measurements will be obtained as a single recording, unless there are emergent abnormalities deemed clinically significant by the Investigator, in which case triplicate ECGs should be obtained.

All 12-lead ECGs, including those performed at Screening, will be of at least 10-second duration. For subjects with well controlled atrial fibrillation (*i.e.*, heart rate ≤ 84 bpm and appropriate anticoagulation), triplicate ECGs are mandatory at all visits.

Electrocardiograms will be acquired according to instructions provided by a centralized ECG reading facility where the ECGs will be centrally assessed. At a minimum, interval data (Fridericia corrected QT interval, QTcF), ventricular rate, and overall interpretation will be reported for each ECG. Data will be transferred electronically for inclusion in the database. Investigators are to enter the machine-read results and any clinical interpretations for the Screening and Baseline (Day -1) ECGs into the eCRF.

Central reading will not be available in subjects with uncontrolled atrial fibrillation (heart rate > 84 bpm) or for subjects with intraventricular conduction defects; therefore, a local ECG reading must be performed. It is recommended that a local cardiologist be involved for correct interpretation and measurements of ECG data, especially in subjects with left bundle branch block.

8.7. Physical and Neurological Examinations

A complete physical and neurological examination will be conducted by the investigator or a physician sub-investigator at Screening (Visit 1). The complete physical examination is to consist of evaluation of the skin, head, eyes (including size and reactivity of the pupils), ears, nose, throat, neck, thyroid, lungs, heart, lymph nodes, abdomen, and extremities (to include deep tendon reflexes, clonus, and muscle rigidity). Any abnormalities noted should be described.

The complete neurological examination is to consist of observation of appearance and behavior (including observation for tremor and abnormal movements) and evaluation of the following: speech, cranial nerves (2 – 12), motor (muscle strength), muscle tone, sensory abnormalities, coordination, gait, and tendon reflexes. At Screening, the primary aim of this assessment is to exclude neurological disorders other than the condition of interest. Any abnormalities noted should be described.

At Visit 2 (Day 1), targeted examinations (as described below) are to be recorded within 1 hour before and approximately 3 hours after administration of the first dose of study drug (and after completion of ECG measurements); these are to be repeated as needed for subjects who remain in clinic longer than 4 hours. Thereafter, targeted examinations are to be performed at each subsequent visit (or upon early termination), including the 4-week post-treatment follow-up visit if applicable (Visit 10).

The targeted examinations are to be focused on, but not limited to, evaluating subjects for potential serotonin toxicity and also as clinically indicated (*e.g.*, targeted to any changes in medical history, in the event of an AE that requires such follow up, or any reported change in the subject's physical condition). At a minimum, they are to include evaluation of deep tendon reflexes, clonus, and muscle rigidity; size and reactivity of pupils; dryness of oral mucosa; intensity of bowel sounds; skin color; and presence or absence of diaphoresis.

Additional targeted examinations may be performed as clinically indicated (*e.g.*, in the event of an AE that requires such follow up or any reported change in the subject’s physical condition).

Findings will be documented in the subject's medical record and in the eCRF.

8.8. Unified Parkinson's Disease Rating Scale (UPDRS Parts II and III)

The Unified Parkinson's Disease Rating Scale (UPDRS) has been widely used in interventional clinical research and development in Parkinson's Disease and other neurodegenerative diseases (Rascol *et al.*, 2002). While a marked decline over 1 year is not to be expected in subjects with bvFTD, this scale has been selected also as a secondary safety and tolerability outcome in the present trial to identify any motor changes the intervention might induce. There is no theoretical reason nor clinical data to assume any effect on the extrapyramidal system by LMTM.

The original UPDRS scale (Fahn and Elton, 1987) was revised in a new version published in July 2008, developed based on the critique by the Movement Disorder Society Task Force on Rating Scales for Parkinson's Disease. The revised UPDRS version will be used in this study. Two parts will be used, requiring approximately 20 minutes in total. The rating is to be completed by the clinician or other trained health professional.

Part II, rated over the prior week by the caregiver, addresses motor aspects of experiences of daily living, with 13 items (speech; saliva and drooling; chewing and swallowing; eating tasks; dressing; hygiene; handwriting; doing hobbies and other activities; turning in bed; tremor; getting out of bed, a car, or a deep chair; walking and balance; and freezing) rated from 0 ("none") to 4, with descriptive anchors for each.

Part III, the motor examination, assesses the state of the subject at the time of the clinic visit. It has shown sensitivity to change in Progressive Aphasia (Boxer *et al.*, 2009). The motor examination consists of the following factors: speech, facial expression, rigidity, finger tapping, hand movements, hand pronation and supination, toe tapping, leg agility, arising from chair, gait, freezing of gait, postural stability, posture, body bradykinesia, postural tremor of the hands, kinetic tremor of the hands, rest tremor amplitude, and constancy of rest tremor.

The UPDRS Parts II and III are to be rated at Baseline (Visit 2; pre-dose) and Weeks 16, 32, and 52 (or early termination).

8.9. Serotonin Toxicity (Syndrome)

Signs and symptoms of serotonin toxicity are to be rated in the clinic by medically qualified personnel for all subjects at Baseline (Visit 2) and each subsequent visit (or upon early termination), including the follow-up visit if applicable (Visit 10). At Baseline (Visit 2), this will be conducted pre-dose and for at least 4 hours after the first dose of study drug while the subject is in the clinic; an assessment is to be completed just prior to discharge from the clinic. This will be rated using the 20-item Serotonin Toxicity (Syndrome) Diagnostic Interview and Rating Guide described in Section 24.7. Rating is to be based on adverse events query, targeted physical and neurological testing, and vital sign measurements, with the pre-dose MMSE derived from the ACE-R as a reference for changes in level of consciousness.

Upon discharge from the clinic, subjects using serotonergic medication at Baseline will be required to remain in close proximity to a local hospital and remain in telephone contact with the clinic for at least 12 – 14 hours post-dose and reimbursement for accommodations will be made available if requested by the subject and/or caregiver. Caregivers of subjects using serotonergic medication will also be contacted by telephone 5-7, >7-14, >14-24, 44-52, and 68-76 hours post-dose (with a minimum of 1 hour between contacts); if indicated, more frequent contacts will be made. The presence and absence of signs and symptoms of serotonin toxicity are to be assessed using the Serotonin Toxicity Telephone Assessment described in Section 24.8. Possible post-

randomization cases of serotonin toxicity will be considered important medical events and reported as per the procedures for SAEs (see Section 8.1.4 for reporting obligations).

8.10. Columbia-Suicide Severity Rating Scale (C-SSRS)

Suicidal behavior and suicidal ideation will be evaluated using the C-SSRS at Screening (Visit 1); Baseline (Visit 2) post-dose (prior to discharge from the clinic); and at each subsequent visit (or upon early termination), including the 4-week post-treatment follow-up visit if applicable (Visit 10). At each assessment, subjects and their caregivers will be asked to indicate whether or not subjects have had an actual attempt, an interrupted attempt, or an aborted attempt and, if so, the number of attempts since the last assessment and in the last week. Subjects will also be queried regarding five aspects of suicidal ideation and their frequency since the last assessment and in the last week: wish to be dead, non-specific active suicidal thoughts, active suicidal ideation with any method (not plan) without intent to act, active suicidal ideation with some intent to act (without specific plan), and active suicidal ideation with specific plan and intent. Any post-randomization affirmative response will be considered an important medical event and reported as per the procedure for an SAE (see Section 8.1.4 for reporting obligations).

Subjects showing indications of suicidal ideation, intent, or action, or who have engaged in self-injurious behavior, should be referred for psychiatric evaluation.

9. OTHER ASSESSMENTS

9.1. Magnetic Resonance Imaging

Brain MRI plays a significant role in this protocol, not only for eligibility evaluation of the subjects, but also as the principal outcome to support disease modification by LMTM. Change in whole brain and ventricular volumes as well as atrophy in frontal and temporal lobes will be quantified at the imaging core laboratory. The imaging core laboratory will be responsible for image collection, checking of the quality of imaging data, anonymization of the images, pre-processing MRI images, presentation of the data to the independent reader(s) (for MRI eligibility and MRI safety evaluations only), and analysis of the MRI data. The core laboratory will provide SAS datasets to the data management and statistics facility for analysis. All systems and processes used for independent and central reads of this trial will be 21 CFR Part 11 compliant. Before commencement of independent, central evaluations, an independent imaging charter will be developed that will describe in detail the imaging acquisition protocol, image collection procedure, quality assurance procedure, site training procedure, reader training, image evaluation procedures (central determination of subjects' eligibility and efficacy), and a communication plan.

All sites will be prospectively trained about imaging requirements including scanner requirements, image acquisition, image transfer to the core laboratory, and timelines that are critical for this trial.

9.1.1. Site Selection and Qualification

A site imaging technical evaluation questionnaire will be distributed to potential clinical sites to evaluate their technical and personnel capabilities that would include machine description, availability of phantoms (if applicable), onsite availability of modality-specific technologist, site experience in evaluating brain MRI, experience in bvFTD and other dementia trials, *etc.* The

site's capability of producing quality data that is necessary for this trial will also be evaluated by appropriate phantom imaging and/or review of imaging data of the site's first subject. Continuous monitoring of the quality of imaging data will be performed throughout the trial. Technical details of quality assurance procedures will be described in a separate imaging manual.

9.1.2. Brain MRI Acquisition

In this trial, 1.5 and 3.0 Tesla MRI machines will be used to reduce the variability in the imaging data. A separate imaging manual will be developed that will outline imaging methods for brain MRI to harmonize image acquisition across the sites. An MRI scan will be performed at Screening (referred to as Screening/Baseline) and again at Week 16 (Visit 5), Week 32 (Visit 7), and Week 52 (Visit 9) or upon early termination. If needed, MRI scans do not need to occur on the same day as efficacy ratings but should occur within the acceptable window. If the MRI scan cannot be performed due to subject's movement, appropriate sedation prior to scanning is permissible.

The Screening/Baseline MRI scan will be performed, after informed consent has been signed, at the Screening visit or subsequently within 42 days before Baseline (Visit 2). Results of the Screening/Baseline MRI must be available by/at Baseline so that an eligibility determination can be made before a subject is considered for randomization (see Inclusion Criteria, Section 5.1).

If the initial Screening/Baseline MRI scan is not of sufficient quality, then a repeat Screening/Baseline MRI may be required and performed (within 42 days before Visit 2). If the repeat scan cannot be accomplished within the 42-day window, then the patient must be reconsented and rescreened. For subjects who are rescreened for other reasons and an acceptable MRI scan was already completed during the original screening window, the scan should only be repeated if the original MRI scan occurred > 42 days prior to Visit 2. The Screening/Baseline MRI will also be used as a baseline for comparison with the Week 16 (Visit 5), Week 32 (Visit 7), and Week 52 (Visit 9) or early termination visit MRI. The Weeks 16, 32, and 52 (or early termination) MRI scans should be performed within ±14 days of the designated visit and will be evaluated for whole brain and ventricular volumes and atrophy in frontal and temporal lobes. If out of window assessments are undertaken at any of the visits, these will be categorized/labelled according to the intended visit designation, regardless of being out of window.

Post-Baseline MRI scans will not be independently reviewed for ARIA at regular intervals; however, if ARIA is suspected and the site submits images to the imaging core laboratory for review, an independent review for ARIA may take place. If evidence of ARIA is noted meeting the discontinuation criteria listed in Table 6-4, dosing should be permanently discontinued. Guidance for follow up is included in Section 6.2.2.3.

If a subject discontinues study drug and/or withdraws from the study prematurely (before completion of the Week 52 visit), then an early termination visit should be conducted at which time all assessments identified for the Week 52 visit should be performed. If the subject's last MRI scan was performed < 90 days prior to the early termination date, no additional MRI scan is required. If the subject's last MRI scan was performed ≥ 90 days prior to the early termination date, the MRI scan must be repeated as part of the early termination visit assessments within the time window of the early termination visit (*i.e.*, ± 14 days of the last dose of study drug); the allowable time window for this scan may be extended to up to 28 days after the last dose of study drug only with Sponsor/medical monitor approval.

The usual MRI subject preparation and safety procedures will be followed. A total duration of approximately 30 minutes will be required. The positioning of the subject in the head coil will follow manufacturer procedures. The recommended range of acquisition parameters for individual sequences for the sites will be developed once technical evaluation forms from all sites have been reviewed and will be described in the imaging manual. The following MRI methodology will be used:

- T2*-weighted gradient-recalled echo (2-dimensional T2* GRE with slice thickness of 5 mm or less and Echo Time of 20 msec or greater); this sequence enables the detection of microbleeds.
- FLAIR: This sequence allows assessment of other clinically significant focal intracranial pathology and for detection of vasogenic edema; it should be done at all imaging time points.
- Unenhanced T1-weighted, 3-dimensional sequence (*e.g.*, MP RAGE or SPGR) is required for evaluation of whole brain and ventricular volumes.
- For eligibility of the subject in addition to the sequences described above, the following sequences will also be required:
  - T1-weighted 3-dimensional MP RAGE imaging to exclude space occupying lesions.
  - Diffusion-weighted imaging to exclude a recent vascular event.

9.1.3. Image Transfer and Quality Assurance

The imaging manual will outline details of image transfer and timelines. Electronic transfer (DICOM format only) is required for this trial for eligibility reads, evaluation of WBV, exploratory efficacy evaluation of ventricular volumes, and safety evaluations for ARIA. Subject data (age, sex and other details) will be removed before central evaluations to reduce the bias and to comply with Health Insurance Portability Accountability Act (HIPAA) and other privacy requirements. Anonymized MRI images are to be immediately transferred to the imaging core laboratory.

Once the images have been received by the imaging core laboratory, they will undergo standardized quality control evaluation by trained technologists. If there are quality issues, the site will be informed and the site will be asked to resolve the quality issues, including rescan of the subject if needed (as allowed; see Section 9.1.2). Quality issues identified by the independent neuroradiologist will be handled in the same fashion.

9.1.4. Image Evaluation Procedure

All safety and eligibility MRI data will be evaluated centrally by experienced independent neuroradiologists (readers) who are not involved in the clinical conduct of the study and are blinded to subjects' clinical information. Efficacy MRI data (whole brain and ventricular volumes as well as atrophy in frontal and temporal lobes) will be evaluated centrally, as described in Section 9.1.4.2. All readers will be prospectively trained before the beginning of independent evaluations. The analysis methods and evaluation methodology including eCRF details will be described in the Independent Review Charter (IRC).

9.1.4.1. Evaluation of Brain MRI for Subject Eligibility

The reader will evaluate the Screening/Baseline brain MRI images for subject eligibility based on imaging-related inclusion and exclusion criteria (presented in Section 5). If the reader

identifies any quality issues pertaining to the images, the process detailed in Section 9.1.3 will be followed. If there are no quality issues, the reader will proceed with the evaluation.

The reader will also confirm frontotemporal atrophic changes on MRI supportive of a diagnosis of bvFTD. Subjects must have a centrally rated frontotemporal atrophy score of 2 or greater, taken as the maximum of right or left frontal or anterior temporal lobes using the rating method developed by Kipps and colleagues (Kipps *et al.*, 2008). The rating method utilizes two defined coronal images through the frontal and temporal lobes. Three lobar regions on these slices (frontal, anterior temporal and posterior temporal) are rated on a 5-point rating scale (ranging from 0 = normal to 4 = severely abnormal). Subjects must have a frontotemporal atrophy score of 2 or greater on MRI to be eligible for the study, irrespective of whether they have pre-existing structural or functional imaging evidence supporting a diagnosis of bvFTD. Ratings of atrophy of 2 or greater have been shown to predict cognitive decline in bvFTD subjects.

Subjects will be evaluated at baseline only for presence and severity of white matter disease based on Fazekas score (Fazekas *et al*., 1987), rated on a 4-point rating scale as summarized below:

0 = None or a single punctate white matter hyperintense lesion

1 = Multiple punctate lesions

2 = Beginning confluency of lesions (bridging)

3 = Large confluent lesions

Results will be communicated to the sites or designee within 5 business days of image receipt (and resolution of any imaging quality queries) by the core laboratory either confirming imaging eligibility or not confirming eligibility for enrollment (the site radiologist may request re-review based on additional clinical/radiological information; see Section 9.1.5).

9.1.4.2. MRI Evaluation for Efficacy

The goal of evaluation of MRI is to show that LMTM can retard the rate of brain atrophy 52 weeks after treatment. This will be shown by comparing post-treatment data with the baseline data. The MRI data acquired at the clinical sites will be sent to the imaging core laboratory for evaluation of whole brain and ventricular volumes as well as atrophy in frontal and temporal lobes; if an early termination visit MRI scan is performed, it will be sent to the imaging core laboratory for this evaluation. The whole brain and ventricular volume change evaluations and determination of atrophy in frontal and temporal lobes will be quantified using the BBSI and VBSI (Fox and Freeborough, 1997). Changes in whole brain and ventricular volumes and atrophy in frontal and temporal lobes will be quantified by the imaging core laboratory. These values of whole brain and ventricular volumes as well as atrophy in frontal and temporal lobes will be utilized for statistical analysis.

9.1.5. Site Review

The site radiologist will review the MRI for preliminary determination of eligibility. A re-review by the central reader may be requested on the basis of additional clinical/radiological information known to the site. Final determination of eligibility from an imaging perspective is based on the independent neuroradiologist review.

The site radiologist will also review the MRI for subject management as well as treatment-emergent abnormalities. Post-Baseline MRI scans will not be independently reviewed for ARIA at regular intervals; however, if ARIA is suspected and the site submits images to the imaging core laboratory for review, an independent review for ARIA may take place. If there is evidence of ARIA meeting criteria listed in Table 6-4, treatment should be permanently discontinued. Guidance for follow up is included in Section 6.2.2.3. Efficacy assessments are to continue as scheduled and the subject is to be encouraged to continue with study visits until the scheduled end of participation for the subject.

The investigator will always retain the responsibility for management of the subject.

9.2. MT Concentrations

Provided the site has a refrigerated centrifuge and adequate capability to reliably freeze samples, blood will be collected at Baseline (Visit 2; pre-dose and approximately 3.5 hours post-dose) and, to the extent possible, at each subsequent on-treatment visit through Week 52 (approximately 20 minutes after ECG recordings) for purposes of population pharmacokinetic (PK) analysis of MT concentrations; samples may also be analyzed for other analytes, such as metabolites, depending on method availability. The time of the prior dose and the time of the blood sample will be recorded. All samples will be tested and disposed of in accordance with the terms of the subjects' consent.

Concentration results will not be made available to the study sites during the conduct of the study. However, results for a given subject (if available) may be provided to the DSMB if requested to aid in interpretation of a significant subject safety issue.

The collection, handling, and shipping of blood samples are described below. These are to be analyzed using validated analytical methods. The analytical results will be reported separately for possible inclusion in future population pharmacokinetic analysis.

9.2.1. Procedure for Blood Sample Collection

Blood samples (6 mL) will be collected into a suitable vacutainer (6.0 mL) as defined in the laboratory manual. All 6 mL of whole blood will be centrifuged at 3000 rpm for 10 minutes at *ca* 4°C; the separated plasma (2 × 1-mL and 2 × 0.5-mL samples) will be transferred into four Nunc polypropylene tubes, as defined in the laboratory manual, and stored at *ca* -20 °C until shipment to the central laboratory for storage. The 2 × 0.5-mL samples will serve as back-up samples for analysis of MT concentrations. The complete sample collection and handling procedures to be followed can be found in the laboratory manual.

9.2.2. Packaging, Labeling, and Shipping of Blood Samples

The samples must be labeled with unique IDs; other labeling information will be detailed in the laboratory manual. Labels must remain intact and indelible throughout processing and frozen storage. Samples will be clearly distinguished from the other bioanalytical samples.

Samples are to be transported in insulated containers filled with dry ice. They will be shipped to the central laboratory where they will be stored frozen and shipped in batches to the analytical laboratory. Samples are to be destroyed after a storage period of 3 months following completion of the clinical study report, which is expected to be a period of approximately 4 years.

9.2.3. Analytical Laboratory

Samples are to be analyzed at the following laboratory:

University of Aberdeen GLP Test Facility
Meston Building
Old Aberdeen
Aberdeen
AB24 3UE
Tel: +44 (0) 1224 272945
Fax: +44 (0) 1224 272921

9.3. Genotyping

For subjects who agree and for whom a separate informed consent signed by the subject or legally acceptable representative is obtained to participate in genotyping evaluation of genetic mutations underlying bvFTD (mutations in the coding regions of Tau and TDP-43 genes), a single blood sample will be obtained. This blood sample may be obtained from a subject at any time after eligibility for randomization and continued participation in the study has been confirmed for that subject. A volume of approximately 5 mL is to be collected in a 10 mL K_2 EDTA plastic tube and stored at -20°C or ideally at -70°C in wire racks.

Samples should then be shipped frozen (on dry ice) to the central laboratory (Covance) on the day of collection where they will be stored at -70°C. Samples will be sent in batches (frozen, on dry ice) to the analytical laboratory responsible for performing the analysis (LabCorp Genomic Services, Seattle, WA, USA) and batch shipped for testing; samples will be destroyed at the end of the study.

Genotyping results will not be provided to the study sites or to subjects.

10. STATISTICAL PLAN

A statistical analysis plan (SAP) will be written and finalized prior to database lock and unblinding of treatment codes. Changes from analyses planned in this protocol will be documented in the SAP and/or final study report. The database will be locked after a blinded review of data and all queries are resolved and decisions made about the inclusion or exclusion of any spurious data and the handling of unused or missing data.

10.1. Efficacy Endpoints

10.1.1. Primary Efficacy Endpoints

The primary efficacy endpoints are the following:

- Change from Baseline to Week 52 in the ACE-R
- Either of two co-primary endpoints:
    - Change from Baseline to Week 52 in FAQ
    - Reduction in decline in whole brain volume at Week 52 using change from Baseline as measured by the Brain Boundary Shift Integral (BBSI) by MRI imaging

10.1.2. Secondary Endpoints

Secondary clinical endpoints include the following:

- Modified CGIC
- FRS
- UPDRS Parts II and III

10.1.3. Exploratory Endpoints

Several exploratory analyses are pre-specified (additional *post hoc* analyses may be performed):

- Change from Baseline to Week 52 in the ACE-III
- Early effect on Modified ADCS-CGIC (after 8 weeks of treatment)
- Effect of LMTM on disease modification by reduction in the rate of atrophy in frontal and temporal lobes as well as ventricular volume as evaluated by MRI
- Effect on MMSE at Week 52
- Effect of LMTM in subjects with known genetic mutations associated with bvFTD (mutations in the coding regions of Tau and TDP-43 genes) (in subjects by or for whom legally acceptable informed consent is provided)

Blood will also be collected for purposes of population pharmacokinetic analysis. This will be detailed in a separate SAP and will be reported separately (together with data from other studies).

10.2. Sample Size Calculations

The assumptions and calculations that form the bases of the sample size are presented below. (See Section 10.4 for monitoring of these assumptions and retention, a key factor in analyses).

10.2.1. ACE-R

The ACE-R, a co-primary endpoint, is expected to decline by approximately 13.4 ± 13.8 units (mean ± standard deviation) in untreated/placebo-treated subjects with bvFTD at 12 months. The estimate of placebo decline and standard deviation is based on data from Kipps *et al.* (2008).

The sample size of 90 subjects per treatment arm will provide at least 90% power for detecting a treatment difference of 6.7 units at a two-sided alpha of 0.05. This corresponds to a 50% reduction in the expected decline in subjects randomized to LMTM as compared to placebo.

10.2.2. FAQ

The FAQ, a co-primary endpoint, is expected to decline by approximately 2.9 ± 1.0 units (mean ± standard deviation) in untreated/placebo-treated subjects with bvFTD at 6 months. The estimate of placebo decline and standard deviation is based on data from Boxer, Knopman, and Kaufer *et al*. (2013).

The sample size of 90 subjects per treatment arm will provide at least 90% power for detecting a treatment difference of 2.9 units at a two-sided alpha of 0.05 at 12 months. This corresponds to a 50% reduction in the expected decline in subjects randomized to LMTM as compared to placebo.

10.2.3. Modified ADCS-CGIC

Approximately 75% of subjects randomized to placebo are expected to have a worsening in the Modified ADCS-CGIC, a secondary endpoint. This estimate is based on proportion of subjects declining over 12 months on the Clinical Dementia Rating Scale-Sum of Boxes-Frontotemporal Lobar Degeneration (CDR-SB-FTLD; Knopman *et al.*, 2008). If only 50% of subjects in the treated group decline, then the power to show a statistically significant difference at a two-sided alpha of 0.05 is greater than 90%. If 55% of subjects in the treated group decline, then the power is 80%.

10.2.4. MRI

There are no longitudinal studies quantifying the rate of brain atrophy in bvFTD. Assuming that the rate of decline in whole brain volume as measured by BBSI is comparable to that of AD over 12 months, the loss of volume is estimated as 14,092 mm^3 with a SD of 10,509 mm^3 in untreated/placebo-treated subjects. The estimates of placebo decline and SD are based on an analysis of data from the ADNI database[6] at 12 months provided by BioClinica. Assuming a scan rate of 100%, a study withdrawal rate of 25% over 12 months, and a loss of scan data points for imaging reasons of 10%, the final retention rate for scan-pairs is expected to be 68% (*i.e.*, 61 scan-pairs in each arm). Based on the use of a two-sided test at the 5% level of significance, there is therefore 90% power to detect a scan effect of +6,200 mm^3 or a 44% reduction in rate of brain atrophy relative to that expected in the control arm at 12 months.

10.3. Statistical Analysis

10.3.1. Analysis Populations

All analyses (other than sensitivity analyses to be described in the Statistical Analysis Plan) will be undertaken with subjects included in the randomly assigned treatment group/stratum. The various analysis populations are defined below.

The Intent-to-Treat (ITT) population will include all randomized subjects.

The Modified Intent-to-Treat (MITT) population will include all randomized subjects who take at least one dose of the study drug and have a Baseline and at least one post-Baseline efficacy measurement.

The Per Protocol population will include all MITT population subjects who do not have any major protocol violations. Major protocol violations will be determined prior to unblinding.

The MITT population will be used for the primary efficacy analysis, including sensitivity analyses. The Per Protocol and ITT populations will be used for additional sensitivity analyses of the efficacy endpoints. Otherwise, the MITT population will be used for all other efficacy analyses. Subjects will be included in the treatment group to which they were randomized.

The Safety population will include all randomized subjects who take at least one dose of study drug. For safety analyses, subjects will be analyzed based on the treatment received.

The Imaging MITT population will include all randomized subjects who took at least one dose of study drug and have a Baseline and at least one post-Baseline MRI imaging measurement of adequate quality.

[6] Estimates based on ADNI 1 data downloaded from the ADNI website (https://ida.loni.ucla.edu) on July 22, 2014

10.3.2. Baseline Characteristics and Concomitant Medications

Tabular summaries of demographics, Baseline characteristics, and concomitant medication use will be prepared based on data collected at the Baseline visit (or, if not available, from the Screening visit).

Medical history will be recorded using the Medical Dictionary for Regulatory Activities (MedDRA) version 15.0 or later. Tabular summaries will be prepared of the numbers and percentages of subjects with abnormalities in a given System Organ Classification (SOC). All data, including study eligibility and Screening data, will be listed.

Concomitant medications and recently used medications (anti-dementia and psychiatric drugs used within the prior 3 months) will be coded using the March 2012 (or later) version of the World Health Organization (WHO) drug dictionary. Tabulations will be prepared of all drugs used concomitantly (relative to the first dose of study drug) based on WHO Anatomical Therapeutic Classification (ATC) level 1 term, ATC level 3 term and Preferred Term (generic name) with frequency and percentage of subjects in each dosing arm using each concomitant medication. Separate tabulation will be made of on-treatment changes in psychiatric medications (to be identified by blinded review).

Concomitant medications (inclusive of medical foods and alternative pharmacotherapies including nutritional/vitamin supplements used specifically for dementia) are to be listed with these elements as well as the verbatim drug name. For subjects on digoxin, available plasma concentrations of digoxin obtained from the local laboratory are to be included.

10.3.3. Dosing and Exposure

Total duration of exposure and mean and modal daily dose per subject (including "dose equivalent" for subjects randomized to placebo), will be summarized descriptively by treatment group. Mean, modal, and maximum dose will also be summarized over selected exposure intervals. In addition, tabular summaries of the proportions of subjects with dose interruptions and dose reductions will be prepared for each treatment group.

Listings will encompass dosing, drug accountability, and compliance (percentage of tablets taken relative to intended number).

10.3.4. Efficacy Analyses

10.3.4.1. Primary Efficacy Analyses

Two clinical co-primary efficacy endpoints will be analyzed to support a symptomatic / delay of disability claim: the ACE-R and the Functional Activities Questionnaire (FAQ). One clinical and one imaging efficacy endpoint will be analyzed to support a disease-modifying claim: the ACE-R and the whole brain volume (WBV).

The testing approach for analysis of the co-primary efficacy endpoints is as follows:

Step 1: The ACE-R endpoint will be analyzed using a two-sided test at the alpha=0.05 level of significance.

Step 2: If the analysis of ACE-R is statistically significant, then the WBV and FAQ will be analyzed using the Bonferroni-Holm method (Holm, 1979). Thus, if the smallest of the two

p-values is <0.025 (based on the use of a two-sided test), then the endpoint with the larger p-value will be tested at the alpha=0.05 level of significance (two-sided).

This approach will permit the ACE-R and WBV outcomes to support a disease-modifying claim in the event that the ACE-R and FAQ do not support a symptomatic / delay of disability claim, or *vice versa*.

The primary analysis of the ACE-R will be carried out using a restricted maximum likelihood based repeated measures linear mixed model with an unstructured covariance matrix in which no data will be imputed. The dependent variable will be the ACE-R change from Baseline. The model will include fixed effects for treatment group (two levels), time (three levels, corresponding to Weeks 16, 32, and 52), the treatment group by time interaction, and geographic region (three levels consisting of the Americas, Europe, and Asia/Australia). In addition, the corresponding Baseline ACE-R score will be included as a covariate. The Kenward and Roger method of calculating the denominator degrees of freedom will be used for the tests of fixed effects. Treatment comparisons will be based on the modeled change from Baseline at Week 52. LMTM will be compared to placebo at the alpha=0.05 level of significance (two-sided). All available post-baseline data will be included in the model, with no imputation for assessments for which ACE-R is not available.

The analysis of the FAQ will be carried out using a restricted maximum likelihood based repeated measures linear mixed model with an unstructured covariance matrix in which no data will be imputed. The dependent variable will be the FAQ change from Baseline. The model will include fixed effects for treatment group (two levels), time (three levels, corresponding to Weeks 16, 32, and 52), the treatment group by time interaction, and geographic region (three levels consisting of the Americas, Europe, and Asia/Australia). In addition, the corresponding Baseline FAQ score will be included as a covariate. The Kenward and Roger method of calculating the denominator degrees of freedom will be used for the tests of fixed effects. Treatment comparisons will be based on the modeled change from Baseline at Week 52. All available post-Baseline data will be included in the model, with no imputation for assessments for which FAQ is not available.

Change in whole brain volume (WBV) using the Brain Boundary Shift Integral (BBSI) will be evaluated using a restricted maximum likelihood based repeated measures linear mixed model with an unstructured covariance matrix in which no data will be imputed. The dependent variable will be the change in WBV (using the BBSI) from Baseline. The model will include fixed effects for treatment group (two levels), time (three levels: corresponding to Weeks 16, 32 and 52) and the treatment group by time interaction. In addition, Baseline WBV will be included as a covariate. The Kenward and Roger method of calculating the denominator degrees of freedom will be used for the tests of fixed effects. Treatment comparisons will be based on the modeled change from Baseline at Week 52. All available post-Baseline data will be included in the model.

10.3.4.2. Additional Sensitivity Analyses of Primary Endpoints

10.3.4.2.1. Missing Data

Because the occurrence of missing data could potentially lead to bias, strenuous efforts will be made to obtain all scheduled efficacy assessments from all randomized subjects, regardless of whether or not they remain on study treatment. In addition, multiple sensitivity analyses will be

completed in order to assess the results of the pre-specified primary analyses. These sensitivity analyses will be described in greater detail in the SAP, and are outlined below.

For the ACE-R, additional sensitivity analyses will be carried out using analysis of covariance (ANCOVA) models. The dependent variable will be the ACE-R change from Baseline to Week 52. The model will include treatment group (two levels), geographic region (three levels consisting of the Americas, Europe, and Asia/Australia) as factors and the Baseline ACE-R score as a covariate. Marginal means (least-square means in SAS) for change from Baseline at Week 52 will be presented for each treatment group. The difference between these marginal means, with 95% confidence interval and p-value, will be provided for the LMTM treatment group *versus* placebo.

Missing values will be imputed by "last z-score carried forward" (LZCF) imputation, as follows. First, from all observations of MITT subjects, the mean and standard deviation of change from Baseline will be computed for each combination of treatment arm and visit, without imputation. A z-score for each subject at each visit will be computed, defined by z=(x-m)/s where x is the subject's change from Baseline, and m and s are the mean and standard deviation for that subject's treatment arm and that visit, computed earlier. Missing observations are imputed by x=m+z*s, where z is the carried-forward z-score, and m and s are the mean and standard deviation for the subject's treatment arm at the visit to be imputed, computed earlier.

Similar analyses will be carried out using the ITT population with Imputation Using Drop-out Reason. For example, the LZCF imputation will be applied for subjects who discontinue due to lack of efficacy / disease progression; for subjects who discontinue for any other reason, the last available observation will be carried forward. Other imputation approaches will be specified in the SAP. In addition, multiple imputation methodology will be used for additional sensitivity analyses.

These same types of sensitivity analyses will also be carried out for the FAQ and WBV.

10.3.4.2.2. Alternate Populations

The primary efficacy analyses of ACE-R, FAQ and WBV will be repeated using the Per Protocol population and the ITT population. Missing data will be imputed as for the primary analyses.

10.3.4.2.3. Responder Analyses

Responder analyses will be performed to support the clinical relevance of the primary analyses. In these analyses, any subject who does not provide an assessment at the specified time point for defining response will be considered to be a non-responder.

For the ACE-R, a responder will be any subject who is not worse than the mean of the marginal means of the linear mixed effects model observed in the placebo and treatment arm on the ACE-R scale at the final visit; alternate definitions of response based on different absolute magnitudes of change as well as percent change from baseline will also be evaluated, to be defined in the SAP. The proportion of responders at Week 52 (or final available visit) will be compared for the LMTM treatment group *versus* placebo using the Cochran-Mantel-Haenszel (CMH) test, adjusting for region.

Similar responder analyses will also be completed for the FAQ and WBV.

10.3.4.3. Analyses of Key Secondary Endpoints

In order to control the overall level of significance for the analysis of the other secondary endpoints, a pre-specified fixed sequence of testing will be used. The order of testing will be:

- Modified ADCS-CGIC
- FRS
- UPDRS Parts II and III

Thus, the analysis of the Modified ADCS-CGIC will be considered confirmatory only if at least one of the two sets of primary analyses (symptomatic, disease modifying) is statistically significant. Similarly, the analysis of the FRS as well as the UPDRS Parts II and III will be considered confirmatory only if all previous analyses are statistically significant. Each of these three endpoints will be analyzed using a two-sided test at the alpha=0.05 level of significance.

For the Modified ADCS-CGIC, counts and percent of subjects in each category at each visit will be presented by treatment group. For analysis at Week 52 the CMH mean score test will be performed using modified ridit scores. The analysis will be stratified by geographic region (three levels consisting of the Americas, Europe, and Asia/Australia) and AChEI and/or memantine status at randomization (two levels: current ongoing use or not ongoing use). For subjects with missing data at Week 52, the last available Modified ADCS-CGIC score will be carried forward. The treatment comparison with placebo will be assessed against a significance level of 0.05.

In order to assess the impact of missing data, multiple sensitivity analyses will be carried out, as follows:

- The analysis described above will be repeated using the data from Weeks 16 and 32 with no imputation for missing data.
- The analysis at Week 52 will be repeated with missing data replaced by the subject's worst (least favorable) value from the earlier time points.
- Modified ADCS-CGIC results at Week 52 will be dichotomized as subjects with and without moderate or marked decline. Subjects without moderate decline are those with scores of 1, 2, 3, 4, or 5. Subjects with missing data will be assigned to the "with moderate or marked decline" category. This dichotomized response will be analyzed using the CMH test, stratified by the same factors as described for the primary analysis.
- Modified ADCS-CGIC results at Week 52 will be dichotomized as subjects with and without any decline, *i.e.*, absence of any decline (scores of 1, 2, 3, 4) *versus* decline (scores of 5, 6, 7). Subjects with missing data will be assigned to the "with decline" category. This dichotomized response will be analyzed using the CMH test, stratified by the same factors as described for the primary analysis.
- Additional repeated measures analyses of the Modified ADCS-CGIC dichotomized as described in each of the preceding two bullets (with and without moderate or marked decline, with and without any decline) will be carried out using the data from Weeks 16, 32, and 52. A Generalized Estimating Equation (GEE) model using the logit link function, the binomial variance function, and the unstructured working correlation model will be fit. This model will include the same covariates as described for the primary analysis. All available data will be used, with no imputation for missing data.

The other secondary endpoints will be analyzed using a restricted maximum likelihood based repeated measures linear mixed model as described for the primary analysis (Section 10.3.4.1).

As a supporting sensitivity analysis, Pearson's chi-squared test will be used to assess the association between ACE-R change and WBV change at Week 52 in the Imaging MITT population. As the thresholds for categorizing the two variables cannot be defined *a priori,* they will be defined as follows: an ACE-R threshold for comparison of WBV change will be defined as the mid-point between the marginal means for change from Baseline to Week 52 for the placebo and treatment arm as determined in the primary efficacy analysis for ACE-R indicated above. Using this threshold, the population will be split into ACE-R “decliners” and “non-decliners” groups. Similarly, the WBV change will be split into two groups based on a threshold defined as the mid-point between the marginal means for change from Baseline to Week 52 for the placebo and treatment arm as determined in the efficacy analysis for WBV. Using this threshold, the Imaging MITT population will be split into WBV “decliners” and “non-decliners” groups. The same analysis will also be carried out using only subjects in the treatment group.

In order to answer the question whether change in WBV at Week 32 is associated with clinical non-decliner status at Week 52, Pearson's chi-squared test will also be used. The WBV threshold will be defined as the mid-point between marginal means for change from Baseline to Week 32. The ACE-R threshold at Week 52 is defined as above. This analysis likewise will be carried out using only subjects in the treated group.

10.3.4.4. Subgroup Analyses

Subgroup analyses are to be performed for the primary efficacy variables with subjects categorized by:

- Region (to be defined on the basis of the location of study sites), *i.e.*, geographic region (three levels consisting of the Americas, Europe, and Asia/Australia), and a combination of geographical region and language (four levels consisting of the Americas, Europe English/non-English, and Asia/Australia)
- Demographics (age [< 60 years and ≥ 60 years], race [white and non-white], sex)
- Early responders (based on Week 8 Modified ADCS-CGIC) *versus* later responders
- Kipps stage 2 *versus* >2
- MMSE 20 – 26 *versus* >26
- Subjects using AD drugs *versus* not using such drugs
- Subjects using *versus* those not using medical food (*e.g*., Axona, Souvenaid) or alternative pharmacotherapy or medical food for dementia (*e.g*., Vitamin E, folate [in doses up to 5 mg/day; use of doses of 1 mg/day as supplementation is not included here], a specific neurocognitive vitamin formulation [such as NeuroVits comprising 20 mg Vitamin B_6, 1 mg Vitamin B_{12}, 0.8 mg folate (see Douaud *et al.,* 2013)], ginkgo biloba, hormone replacement therapy, coconut oil, curcumin)

10.3.4.5. Exploratory Efficacy Analyses

All analyses as described for the ACE-R in Section 10.3.4.1 will be repeated using the ACE-III score.

The change in MMSE score from Baseline to Week 52 will be analyzed using an ANCOVA model with effects for treatment group (two levels), treatment group by time interaction, geographic region (three levels consisting of the Americas, Europe, and Asia/Australia) and Baseline MMSE score as covariates. The primary analysis will be based on the MITT population. Missing values will be imputed by "last z-score carried forward" (LZCF), defined as for ACE-R in Section 10.3.4.2.1. The difference between the least-square means, along with 95% confidence interval and p-value, will be provided for the LMTM treatment group *versus* placebo.

A linear mixed effects model will also be fit for ACE-R, FAQ, and WBV that includes fixed effects for treatment group and the interaction between treatment group and time (where nominal visit time after Baseline will be treated as a continuous variable). From this model, the statistical significance of the effect of treatment on the rate of decline will be reported. Tests of the significance of inclusion of cubic terms of the time effects will be carried out in each treatment group and overall. If the cubic terms are non-significant, a reduced model will be fit in which the time effects are parameterized as quadratic functions. In this model, assessments of the rate of change in each group will be carried out.

The same analysis as for WBV will be carried out for atrophy in frontal and temporal lobes, as well as ventricular volume, as evaluated by MRI.

Additional exploratory analyses, including based on presence or absence of genetic mutations, may be described in the SAP.

10.3.4.6. Pooling of Small Study Sites

Because the study will include a large number of study sites, relative to the total number of subjects, the effect of study site will not be included in the statistical analysis models. However, region will be included as a factor.

10.3.4.7. Missing Data

Unless otherwise specified, efficacy data missing for an entire outcome scale or for the majority of the scale will not be imputed for analysis. Instead, it will be assumed that the data are close to missing at random after accounting for the terms in the model. However, missing items within a scale may be imputed if some items of the scale are present. Further details for each individual endpoint will be given in the SAP.

Early termination efficacy assessments will be allocated to the corresponding window for a target week.

10.3.5. Safety Analysis

Safety results will be summarized using the safety population. The analysis of safety will include summaries of AEs, laboratory tests of blood and urine, pulse co-oximetry testing, vital signs and weight, ECGs, physical and neurological examinations, suicidality assessment, serotonin toxicity assessments, and cases identified with ARIA.

10.3.5.1. Adverse Events

Adverse events will be coded to a System Organ Class (SOC) and preferred term using MedDRA (version 15.0 or later). Adverse events will be regarded as treatment-emergent (TEAE) if they start on or after the first dose of study drug administration or if they were present prior to

the first date of study drug administration and increased in severity or relationship to study drug while on study treatment.

Tabular summaries are described below, with the number and percentage of subjects reporting each type of event presented by treatment group. If a subject reports the same preferred term more than once, it is counted only once within that category. Further, for a given tabulation, the preferred term will only be counted once in its worst severity, greatest relationship to treatment, and worst action taken.

Pre-treatment AEs, those present from the time of consenting until the first date of study drug administration, will be presented separately.

An overall summary table of TEAEs will be produced showing the number and percent of subjects with the following: TEAE, severe TEAE, TEAE related to study drug, serious TEAE, TEAE with outcome of death, and TEAE leading to interruption, dose reduction, or discontinuation from the study. In addition, the summary table will include the number of TEAEs, severe TEAEs, TEAEs related to study drug, and serious TEAEs.

Separate summaries of incidences (number and percentage of subjects) of all individual TEAEs and the subsets of drug-related TEAEs, TEAEs that are severe in intensity, serious TEAEs, and TEAEs leading to change in dose will be provided.

Certain TEAEs, identified by a blinded data review, will be grouped and summarized. These will include gastrointestinal and urinary tract events, falls, rashes, and possible serotonin syndrome. Details are to be defined in the SAP.

The subset of TEAEs that have an onset, increase in severity, or increase in relationship to study drug more than 7 days after the last dose of study drug will be considered post-treatment. An overall summary table of the number and percent of subjects in each treatment group with post-treatment AEs will be prepared.

Subgroup analyses will be performed in which the effects of demographics (sex, age, race/ethnicity) on selected group events will be evaluated. Other subgroups of interest may also be identified.

Interactions between MT and selected concomitant medications will be investigated for selected grouped events using Cox's proportional hazard models.

All AEs will be presented in a data listing. In addition, listings also will be provided for SAEs, AEs leading to death, AEs leading to discontinuation of study drug, AEs leading to dose reduction and/or interruption, and any other AEs that are of special interest (to be defined prior to database lock).

10.3.5.2. ARIA

Summaries of ARIA will be based on the subset of subjects who were followed for ARIA (prior to implementation of Amendment 6.0). At each scheduled time point, ARIA data will be summarized descriptively by treatment group. The proportions of subjects who meet case definitions for ARIA, *i.e.*, vasogenic edema, macrohemorrhages, microhemorrhages, or an area of superficial siderosis, will be tabulated by treatment group. Likewise, number of microhemorrhages will be tabulated by treatment group.

All identified cases of ARIA will be listed, including those identified by the site. All recorded information will be included in listings.

10.3.5.3. Laboratory Tests of Blood and Urine

Descriptive statistics will be based on central laboratory results as described below. Laboratory results that are obtained from laboratories other than the central laboratory will not be included in tabular summaries. They will, however, be listed separately. Normal ranges will be provided by the central laboratory and each local laboratory used for testing parameters other than Heinz bodies.

The central laboratory will calculate and report creatinine clearance using the Cockcroft-Gault equation. Estimated glomerular filtration rate (eGFR) will be calculated based on the Modification of Diet in Renal Disease (MDRD) Study, to be further described in the SAP.

Categorical laboratory parameters will be summarized by treatment group for each target week using counts and percent of subjects in each result category. Continuous laboratory parameters will be summarized by treatment group for each target week using descriptive statistics for both the original values and the change from Baseline. Visit windows for each target week will be used (see SAP for details). For each analyte, Baseline values will be restricted to those subjects in the safety population for whom there is at least one post-Baseline value.

Selected parameters for which normal ranges differ may additionally be summarized by gender, age, and country and/or region.

Box and whisker plots will be presented for selected parameters including hemoglobin, methemoglobin, reticulocytes, neutrophils, and liver function tests.

Shift tables will be provided cross tabulating the number of subjects who are low, normal, or high at Baseline (with respect to the normal range) against results at each target week.

Potentially clinically significant ranges will be defined in the SAP for selected parameters, and the number and percent of subjects meeting these criteria summarized. Tabular summaries will include only those subjects in whom the values represent a treatment-emergent worsening.

Listings of laboratory parameters will be presented. Listings will flag results above and below the normal range as well as those that meet criteria for being potentially clinically significant (whether or not a treatment-emergent worsening). Separate listings for each hematology, chemistry, and urinalysis parameter will show results that meet criteria for being potentially clinically significant. In addition, for subjects who have one or more result that meets criteria, all laboratory results will be displayed.

10.3.5.4. Vital Signs and Weight

Blood pressure, pulse, temperature, respiratory rate, and weight will be summarized by treatment group for each target visit using descriptive statistics for both the original values and the change from Baseline. Windows for each target visit will be used (see SAP for details).

Potentially clinically significant vital sign changes will be defined for selected parameters, and the number and percent of subjects meeting these criteria will be summarized by treatment group.

Listings of vital sign measurements will be presented; height will be included in the demographic listing as it is measured only once at Screening. Listings will flag results that meet criteria as

being potentially clinically significant. Separate listings for each vital sign parameter will show results that meet criteria for being potentially clinically significant. In addition, for subjects who have one or more result that meets criteria, all corresponding vital sign results will be displayed.

10.3.5.5. Electrocardiogram

ECG results will be provided by the central ECG reader (inclusive of the Screening ECG). Data analyses and summary tables will be based on the central ECG results. The machine-read ECG results and interpretations from Screening and Baseline (Day -1) are to be recorded in the eCRF and included in a separate listing; this also applies to any subsequent local interpretations if used to make dosing or patient management decisions.

ECG interval data will be summarized for Screening and Day 1 pre- and post-dose measurements based on the average of the triplicate measures. All other study visit ECG data [interpretations and interval data based on single measurements (or means of triplicate measurements if performed for clinical reasons)] will be summarized separately.

Electrocardiogram interval and ventricular rate data will be summarized by treatment group using descriptive statistics for both the original values and the change from Baseline. Windows for each target visit will be used (additional details provided in the SAP). Parameters to be analyzed include heart rate (HR) and PR, QRS, QT, and corrected QT (using Fridericia's and Bazett's corrections), and RR intervals. Counts and percent of subjects in each result category will be tabulated. Overall interpretations of abnormality(ies) will also be tabulated, with subjects categorized by whether or not they have treatment-emergent abnormalities.

Subjects are also to be categorized and enumerated on the basis of QTc interval and change from Baseline (mean of triplicate Day 1 pre-dose measurements) as follows:

- QTcB/F outliers (in categories of > 450 to ≤ 480, > 480 to ≤ 500, > 500 msec)
- Change in QTcB/F outliers (in categories of > 30 to ≤ 60, > 60 to < 90, ≥ 90 msec)

All HR, interval data, and interpretations will be listed. Any new finding after Baseline (pre-dose on Day 1) will be categorized into diagnostic groups: rhythm, conduction, hypertrophy, arrhythmia, ischemia, infarction, other.

10.3.5.6. Physical and Neurological Examinations

Pre-treatment physical and neurological examination results will be summarized by body system. Evaluation for subsequent visits will be summarized in a treatment-emergent fashion *i.e.*, presenting the number and percentage of subjects with normal and abnormal observations by body system/parameter evaluated. By-subject listings will detail the abnormality(ies).

10.3.5.7. UPDRS

As the UPDRS Parts II and III provide efficacy and safety information, the total score on each of these parts will be analyzed regardless of the outcome on other variables.

The total score on each part will be analyzed using a restricted maximum likelihood based repeated measures linear mixed model as described for the primary analysis (Section 10.3.4.1).

In addition, the composite of the UPDRS Parts II and III will be evaluated with respect to the extent to which bradykinesia / bradypsychia may interfere in the efficacy assessments.

10.3.5.8. Serotonin Toxicity (Syndrome)

Ratings from the Serotonin Toxicity (Syndrome) Diagnostic Interview and Rating Guide (In-Clinic) and, in those subjects in whom it is applicable, the Serotonin Toxicity Telephone Assessment, will be listed. The total score for each of the four possible diagnostic criteria will be confirmed programmatically.

These data, together with adverse events, vital sign measurements, and physical and neurological examination results, will be reviewed to identify subjects with potential serotonin syndrome. This medical review will be performed prior to database lock and unblinding. The proportion of subjects in each treatment group thus identified will be summarized.

10.3.5.9. Columbia Classification Algorithm of Suicide Assessment (C-CASA)

Results of the C-SSRS will be evaluated and reported according to the Columbia Classification Algorithm of Suicide Assessment (C-CASA) and listed. AE data will also be reviewed prior to database lock and unblinded to identify any additional potential cases.

10.3.6. Concentration of MT

Blood samples for the measurement of MT concentration (including time relative to prior dose) will be summarized using descriptive statistics at each scheduled collection visit. The statistics n (number of subjects with data), mean, median, standard deviation, minimum, maximum, geometric mean and coefficient of variation will be presented at each scheduled collection visit. PK summaries will be based on the PK population.

Separate summaries will also be presented for subjects with and without dose reductions.

Mean concentration will be plotted against time using linear axes.

The PK data will be modeled using a population pharmacokinetic approach. Parameters such as clearance (including weight-adjusted clearance) will be calculated and demographic, concomitant drug, and disease characteristics explored to identify any that are potentially significant covariates. The possibility of a concentration response relationship may also be investigated. This work will be reported separately to the clinical study report and will be subject to a separate analysis plan.

10.3.7. Genotyping

Genotyping results will be listed and summarized descriptively for each treatment.

10.4. Interim Analysis

No interim futility or efficacy analysis is planned in which treatment groups will be compared.

Recruitment and discontinuations will, however, be continuously monitored in a blinded fashion and projections on future dropouts calculated in order to check whether the 20% withdrawal assumption from the sample size calculation holds. Should the overall dropout for the duration of the study be projected to exceed 20%, then the number of subjects to be enrolled may be increased in order to power an analysis at 32 or 52 weeks; see Section 10.2.

In addition, a blinded interim analysis to re-estimate the assumed SD for the change from Baseline to Week 52 in ACE-R may be carried out at some point during the study’s recruitment period. An estimate of the pooled SD would be computed; however, the treatment difference would not be provided to the designated party. Based on the estimated SD at this blinded interim

analysis, the sample size required to provide 90% power to detect a treatment difference of approximately 50% reduction in expected decline would be re-estimated. The sample size may then be increased by a maximum of 33% (*i.e.*, from 180 subjects to 240 subjects). However, the sample size will not be decreased from 180 subjects.

11. REGULATORY

Investigators and all other parties involved in the conduct of the study are responsible for ensuring that the study is conducted at their sites in accordance with the approved protocol and with the principles of the Declaration of Helsinki (most current applicable version), the International Conference on Harmonisation (ICH) Guidelines for Good Clinical Practice (GCP) (CPMP/ICH/135/95, July 1996), and with applicable country and local regulatory requirements and laws.

The Sponsor will be responsible for ensuring that the relevant approval is obtained from the local regulatory authority prior to the start of the study. The relevant documents will be provided to the investigator. The Sponsor or designee will forward any protocol amendments to the regulatory authority and will ensure that SAEs are reported, and progress reports and details of any serious protocol violations are provided as required.

The regulatory authority will be informed should the study be terminated early.

12. APPROVAL OF THE PROTOCOL AND AMENDMENTS

Following authorization by the Sponsor, the final protocol and all related documents (*e.g.*, information sheets and ICFs) will be submitted to the IEC/IRB.

The Sponsor will be responsible for ensuring that regulatory and IEC/IRB approvals are obtained prior to the start of the study. The relevant documents will be provided to the investigator.

Neither the investigator nor the Sponsor will modify this protocol. If modification is necessary, either party must first obtain the concurrence of the other. The party initiating a modification will confirm it in writing, and the investigator will be responsible for informing the IEC/IRB. In case of a substantial amendment, prior approval of the IEC/IRB is required.

TauRx or designee is responsible for submission of a protocol amendment to the regulatory authority. In the event of a substantial amendment, prior approval is required.

13. SERIOUS BREACHES

The investigator and all other parties involved in the conduct of the study will comply with the protocol and ICH GCP. All deviations will be reported to the study monitor.

It is the responsibility of the Sponsor to notify the licensing authority of any serious breach which is likely to affect, to a significant degree, the safety or mental integrity of the subjects of the study or the scientific value of the study.

All serious breaches will be notified to the pertinent regulatory authorities according to the relevant national regulatory requirements. The reporting will be *via* the Sponsor in accordance with TauRx SOPs.

14. INFORMED CONSENT

It is the responsibility of the investigator or a medically trained, medically qualified sub-investigator to obtain informed consent from each subject participating in this study, or his/her representative that is permitted to provide consent in accordance with local legislation. Where required by local law, the person who informs the subject must be a physician.

Potential subjects will be assessed for whether they have capacity to understand the ICF and give consent.

Where possible, fully informed consent will be obtained from the subject. However, subjects entering this study may lack the necessary mental capacity to give fully informed consent. If the potential subject is unable to comprehend the ICF, then one or more legally acceptable representatives will be required to sign the ICF as required by national law. In this situation, and provided that it is permitted by local legislation, the subject's agreement to participate in the study will still be obtained to his/her best level of understanding and recruitment will not proceed if the subject refuses or shows significant distress.

Subjects and/or their legal representative(s) must give written (signed and personally dated) informed consent prior to study entry and before any study specific procedures are undertaken. The identified caregiver(s) for each subject also must provide written consent to his/her own participation as outlined above. Where there is a change of caregiver, the new caregiver must provide written informed consent.

Informed consent can be obtained only after the aims, methods, anticipated benefits, and known potential hazards of the study have been explained to and discussed with the potential subject and caregiver by the investigator. A subject information sheet, providing a written summary of all relevant information, will be given to the potential subject and caregiver prior to written informed consent being obtained. The caregiver will also be given an information sheet. The information sheet will make clear that access to the subject's medical records will be required. It is the responsibility of the investigator to ensure that the potential subject and/or caregiver are aware of this. The investigator will explain to the potential subject and caregiver that they are at liberty to refuse to take part in the study or, should they decide to participate, they may withdraw from the study at any time. Such a decision should not, in any way, impinge on the future management of the subject. The potential subject and caregiver will be allowed as much time as they need to decide whether or not to participate in the study and will be provided with a contact point where further information about the study may be obtained.

The study is being performed in the European Union and the United States, two geographic regions that maintain descriptions of clinical studies on the internet. As required by the U.S. Food and Drug Administration, the ICF must contain the following text: "A description of this clinical trial will be available on http://www.ClinicalTrials.gov, as required by U.S. Law. This Web site will not include information that can identify you. At most, the Web site will include a summary of the results. You can search this Web site at any time." Consistent with European

Union law, the ICF also indicates that information will be on https://www.clinicaltrialsregister.eu. Information regarding other national registries will also be included in the ICF, where applicable.

15. INVESTIGATOR RESPONSIBILITIES

The primary responsibility of all investigators participating in the study is for the well-being and interests of their subjects, including subjects enrolled in this study. The investigator has overall responsibility for the conduct of the trial at his/her study site and may delegate specific duties to appropriately trained members of his/her research team or to other hospital staff, *e.g.*, the pharmacy. Any delegation must be clearly documented in a study site specific delegation list.

The investigator is responsible for the following:

- Performing the study in accordance with ICH GCP
- Ensuring that adequate time and appropriate resources are available to perform the study as described in this protocol
- Ensuring that all persons assisting with the trial are adequately qualified, trained, and informed about the protocol, trial-related duties, and functions
- Maintaining a list of sub-investigators and other appropriately qualified persons to whom duties have been delegated
- Signing an Investigator Agreement to confirm acceptance and willingness to comply with the study protocol
- Maintaining adequate control of study drug and appropriate records of drug disposition
- Maintaining adequate records of each subject's participation

Where local laws require it, national regulatory requirements with regard to the inclusion of subjects who are unable to consent will be followed by the investigators. In particular, in Germany, the risk threshold and degree of burden/distress will be monitored constantly by the investigators in accordance with §41, section 3, of the German Drug Law (AMG).

TauRx and the appointed DSMB will constantly evaluate the risk threshold of this particular study by assessing the safety profile according to Sections 4.8 and 8, as well as assessing the safety profile of other ongoing studies with the same active moiety. Any changes in the risk profile during the course of the study will be communicated to the investigators. In addition, the investigators will review adverse events at each visit in accordance with the schedule of assessments (see Table 4-1), and have the right to reduce the dose, interrupt or discontinue study drug for safety reasons as described in Sections 5.3 and 6.2.2.

The burden/distress associated with participation in this study is addressed in the patient information leaflet. During the study, the investigators should obtain information from the subjects in order to adequately monitor the degree of burden/distress. Subjects are advised that they have the right to discontinue study drug and withdraw from the study at any time for any reason and should inform the investigators accordingly in order to assist the investigators with monitoring activities.

16. CONFIDENTIALITY AND DATA PROTECTION

All study-related documentation is confidential, whether obtained by the investigator or provided by TauRx or their representative.

The investigator must ensure the anonymity of subjects in the trial is maintained on eCRFs, samples, specimens, and other documents leaving the study site and submitted to TauRx or its designees. Subjects must NOT be identified by name, but by an identification code (usually trial number). For all subjects (including subjects who were screened but not enrolled), the investigator must keep a separate log of subject codes, names, and addresses.

To conform to the requirements of EU Directive 95/46/EC, subjects will be explicitly asked to consent to transmission of their data outside the European Economic Area. In the United States, data will be protected consistent with HIPAA.

Confidentiality of the records identifying the subject will be maintained. Representatives of the Sponsor such as monitor(s) or auditor(s), IRB/IEC, and pertinent regulatory authorities will be permitted direct access to these records and other source data/documents as appropriate.

Details of access to the subjects' data will be fully described within the subject and caregiver information sheet. The consequence of the subject's withdrawal of consent with regards to the use of data will also be described.

17. QUALITY ASSURANCE AND CLINICAL MONITORING

Standard operating procedures (SOPs) will be adhered to for all activities relevant to the quality of the study, including protocol compliance, data collection, quality control, and data analyses and reporting.

All aspects of the study will be subject to a Quality Assurance (QA) audit plan. QA audits will be conducted on critical phases during the clinical and reporting phases of the study. These audits will be carried out by QA personnel, independent of the staff involved in the study, according to relevant SOPs.

Clinical monitoring, both primary and secondary, will be performed by trained clinical research personnel. Clinical monitoring is an integral part of controlling and securing of data integrity and subject safety. The first monitoring visit will be scheduled appropriately after the first subject is screened at a site depending on factors that could impact on data reliability, some of which are mentioned below. The average monitoring frequency will be described in a clinical monitoring plan and will depend on a number of factors including subject screening and recruitment rates, site performance, and quality adherence. Regulatory recommendations and guidelines will be followed. Detailed expected monitoring activity will be described in the clinical monitoring plan, which will be modified on an ongoing basis to ensure subject safety and data integrity.

The monitor will ensure compliance with the protocol, adherence to regulatory and ICH obligations, accurate reporting of AEs, maintenance of trial records including drug accountability records, and correct administration of study procedures including supply and storage of study materials. ICFs will be reviewed to verify that they are correctly signed and dated by the subject

and caregiver and the investigator or medically trained sub-investigator. At each monitoring visit, subject data will be reviewed and verified against the medical records.

The monitor will require direct access to laboratory test results and other records needed to verify entries on the eCRF.

The investigator (or his/her designated deputy) agrees to cooperate with the monitor and other clinical research personnel to ensure that any problems detected in the course of these monitoring visits are quickly resolved.

Secondary monitoring of data and/or trial documentation may be carried out by or on behalf of the Sponsor at any stage. Audits of study sites and/or trial processes may be carried out at any stage.

18. DOCUMENTATION

The protocol, its amendments, and any other required documents must be submitted for appropriate regulatory review and approval.

The investigator at each study site must generate and maintain adequate records (medical records, source documents, and eCRFs) to enable the conduct of this study to be fully documented.

Initially, data will be collected on source documents which will then be transcribed to the eCRF. The eCRF may serve as the primary collection medium for any data (to be agreed with the investigator and documented in the Source Data Verification Agreement). Each enrolled subject must have an eCRF completed and this must be reviewed and approved by the investigator.

The documents specified by ICH GCP (*e.g.*, copies of protocols, CRF pages, original copies of test results, reports, drug dispensing logs, correspondence, records of informed consent, and other documents pertaining to the conduct of the study) must be kept on file by the investigator for a minimum of 15 years or for the period of time specified by local law for the preservation of hospital patient documents, whichever is the longest. No study documents should be destroyed without prior written agreement between TauRx and the investigator. Should the site wish to assign the study records to another party, or move them to another location, TauRx must be informed.

A record must be kept of all subjects consenting for the study and subsequently excluded. The reason for non-participation in the study should be recorded.

The following documents must be provided before or at site initiation.

- Protocol and amendments (if applicable) signed and dated by applicable Sponsor representatives, as well as by the investigator
- Regulatory approval (or in absence of document, evidence that study may proceed)
- Signed and dated IEC approval
- Approved subject information sheet, ICF, and advertisement for recruitment (if any)
- eCRFs

- Confidentiality agreement(s)
- Financial disclosure
- Study drug/shipping records
- Signed *curricula vitae* (CV) for personnel who have signed the authorized delegation log (including principal investigator, all sub-investigators, and designated assistants)
- Authorized signature log/delegation list
- Investigator Brochure with signed and dated Investigator Brochure receipt
- Signed and dated clinical trial agreement
- Research and development (or institution) approval, if applicable
- Signed and dated indemnity/insurance statement (if applicable)
- Laboratory reference ranges and accreditation for all applicable laboratories (central and local, as applicable)
- Pharmacy agreement (if any)
- Instructions for handling investigational product
- Sample label
- SAE forms

19. PUBLICATION

Since this is a multiple site study, the community of investigators and delegated individual investigators shall not publish any partial results before the end of the study or before the analysis and publication of the results of the entire study.

The investigator and/or institution shall have the right to publish, display, or otherwise communicate orally, in writing, or electronically (hereafter a "publication") the results of their work conducted under this protocol after 12 months from NDA or equivalent filing, or earlier only with explicit consent of Sponsor in advance and in writing.

Sites and/or investigators must provide the Sponsor with the opportunity to review the contents of any proposed abstract or publication concerning the work, including any results of the study, in advance of publication, and agree to delay the publication if, in the Sponsor's reasonable view, the publication may prejudice the Sponsor's intellectual property. The Sponsor will make every reasonable effort to consider and release each proposed abstract or publication within 60 days of submission. The investigator and/or site will include where possible comments made by the Sponsor. Authorship will be determined by mutual agreement. Access to data will be in accordance with authorship.

20. INDEMNITY, INSURANCE AND COMPENSATION

A clinical trials insurance and product liability insurance policy will be in place to cover the conduct of this study.

21. ADMINISTRATIVE AND FINANCIAL AGREEMENT

Agreed costs for each participating study site will be met by TauRx. For each study site, an agreement will be prepared and signed off by the relevant authority on behalf of the institution (*e.g.*, National Health Service Trust, University) and by TauRx or its designee before the initiation of the trial. Each investigator and subinvestigator must also sign a Form FDA 3455 or its equivalent to disclose any financial arrangements or interests.

Subjects will be reimbursed by TauRx, through the investigator, for reasonable travel costs to and from the study site and accommodation in certain circumstances by prior agreement with the Sponsor.

22. STUDY ADMINISTRATION

This trial will be conducted in compliance with ICH GCP and other applicable regulatory requirements.

Contract Research Organizations and/or independent contract personnel will be contracted to manage and monitor the trial, to provide services for data management and statistical analysis, to provide regulatory advice and services, to handle the reporting of Serious Adverse Events, to provide services for laboratory and PK analysis, to package and distribute the clinical trial supplies, and to provide quality assurance support and services.

Calibration certification for the following equipment used to generate study data will be confirmed: ECG machines, pulse co-oximeters, pharmacy temperature loggers, and pipettes.

23. REFERENCES

Alexopoulos P, Ebert A, Richter-Schmidinger T, *et al*. Validation of the German revised Addenbrooke's cognitive examination for detecting mild cognitive impairment, mild dementia in alzheimer's disease and frontotemporal lobar degeneration. Dement Geriatr Cogn Disord 2010; 29:448-456.

Allen VJ, Methven L, Gosney MA. Use of nutritional complete supplements in older adults with dementia: Systematic review and meta-analysis of clinical outcomes. J Clin Nutr 2013. http://dx.doi.org/10.1016/j.clnu.2013.03.015.

Baddeley TC, McCaffrey JM, Storey J, *et al*. Complex disposition of methylthioninium redox forms determines efficacy in tau aggregation inhibitor therapy for Alzheimer's disease. J Pharmacol Exp Ther 2015; 352:1-9.

Barker SJ, Curry J, Redford D, *et al*. Measurement of carboxyhemoglobin and methemoglobin by pulse oximetry. Anesthesiology 2006; 105(5):892-897.

Boxer AL, Lipton AM, Womack K, *et al*. An Open Label Study of Memantine Treatment in Three Subtypes of Frontotemporal Lobar Degeneration. Alzheimer Dis Assoc Disord 2009; 23(3): 211–217.

Boxer, AL, Knopman, DS, Kaufer, DI, Grossman, M, Onyike, C *et al*. (2013) Memantine in patients with frontotemporal lobar degeneration: a multicentre, randomised, double-blind, placebo-controlled trial. Lancet Neurol. 12:149-156

Conclusions of a WHO Technical Consultation on folate and vitamin B_{12} deficiencies. Food Nutr Bull. The United Nations University, 2008;29(2 Suppl):S238-244.

Douaud G, Refsum H, de Jager CA, *et al*. Preventing Alzheimer's disease-related gray matter atrophy by B-vitamin treatment. PNAS 2013; 110(23): 9523-9528.

Fahn S, Elton RL, UPDRS Development Committee. Unified Parkinson's Disease Rating Scale. In: Fahn S, Marsden CD, Calne DB, Goldstein M, eds. Recent Developments in Parkinson's Disease, Vol 2, 1987. Florham Park: Macmillan Health Care Information, 153-163, 293-304.

Fazekas F, Chawluk JB, Alavi A, *et al*. MR signal abnormalities at 1.5 T in Alzheimer's dementia and normal aging. Am J Roentgenol 1987; 149:351-356.

Folstein MF, Folstein SE, McHugh PR. "Mini-mental State": A practical method for grading the cognitive state of subjects for the clinician. J Psychiatr Res. 1975; 1:189-198.

Fox NC and Freeborough PA. Brain atrophy progression measured from registered serial MRI: Validation and application to Alzheimer's disease. J Mag Res Imaging 1997; 7(6):1069-1075.

Gourlain H, Buneaux F, Borron SW, *et al.* Interference of methylene blue with co-oximetry of hemoglobin derivatives. Clin Chem 1997; 43(6):1078-1080.

Harrington CR, Storey JD, Clunas S, *et al*. Cellular models of aggregation-dependent template-directed proteolysis to characterize tau aggregation inhibitors for treatment of Alzheimer's Disease. J Biol Chem 2015; 290: 10862–75.

Hodges JR, Davies RR, Xuereb JH, *et al.* Clinicopathological correlates in frontotemporal dementia. Ann Neurol 2004 ;56(3):399-406.

Holm, S. A simple sequentially rejective multiple test procedure. Scandinavian Journal of Statistics 1979; 6(2):65–70.

Hsieh S, Schubert S, Hoon C, *et al*. Validation of the Addenbrooke's Cognitive Examination III in Frontotemporal Dementia and Alzheimer's Disease. Dement Geriatr Cogn Disord 2013; 36: 242–250.

Izdes S, Altintas ND, Soykut C. Serotonin syndrome caused by administration of methylene blue to a patient receiving selective serotonin reuptake inhibitors. Anesth Analg 2014;2(9):111-112.

Kipps CM, Davies RR, Mitchell J, *et al.* Clinical significance of lobar atrophy in frontotemporal dementia: Application of an MRI visual rating scale. Dement Geriatr Cogn Disord 2007; 23(5):334-342.

Kipps CM, Nestor PJ, Dawson CE, *et al*. Measuring progression in frontotemporal dementia: Implications for therapeutic interventions. Neurology 2008; 70(22):2046-2052.

Knopman DS, Kramer JH, Boeve BF, *et al*. Development of methodology for conducting clinical trials in frontotemporal lobar degeneration. Brain 2008; 131(pt 11):2957-2968.

Larner AJ. Addenbrooke's Cognitive Examination (ACE) for the diagnosis and differential diagnosis of dementia. Clin Neurol Neurosurg 2007; 109(6):491-494.

Mackenzie IR, Meumann M, Bigio EH, *et al.* Nomenclature and nosology for neuropathologic subtypes of frontotemporal lobar degeneration: an update. Acta Neuropathol 2010; 119(1):1-4.

Mackenzie IR, Neumann M, Cairns NJ, *et al*. Novel types of frontotemporal lobar degeneration: beyond tau and TDP-43. J. Mol. Neurosci 2011; 45(3):402-408.

Malouf R, Grimley Evans J. Folic acid with or without vitamin B12 for the prevention and treatment of healthy elderly and demented people. Cochrane Database Syst Rev 2008; 4:CD004514.

Malouf R, Areosa Sastre A. Vitamin B12 for cognition. Cochrane Database Syst Rev 2003; 3:CD004326.

Mathuranath PS, Nestor PJ, Berrios GE, *et al.* A brief cognitive test battery to differentiate Alzheimer's disease and frontotemporal dementia. Neurol 2000; 55(11):1613-1620.

Melis V, Magbagbeolu M, Rickard JE, *et al*. Effects of oxidized and reduced forms of methylthioninium in two transgenic mouse tauopathy models. Behav Pharmacol 2015 (epub ahead of print).

Mioshi E, Dawson K, Mitchell J, *et al.* The Addenbrooke's Cognitive Examination Revised (ACE-R): a brief cognitive test battery for dementia screening. Int J Geriatr Psychiatry 2006; 21(11):1078-1085.

Mioshi E, Hsieh S, Savage S, *et al.* Clinical staging and disease progression in frontotemporal dementia. Neurology 2010;74(20):1591-1597.

Moore E, Mander A, Ames D *et al*. Cognitive impairment and vitamin B12: a review. Int Psychogeriatr 2012; 24(4):541-556.

Movement Disorder Society Task Force on Rating Scales for Parkinson's Disease. The Unified Parkinson's Disease Rating Scale (UPDRS): status and recommendations. Mov Disord 2003; 18(7):738-750.

Mukaetova-Ladinska EB, Garcia-Siera F, Hurt J, *et al.* Staging of cytoskeletal and β-amyloid changes in human isocortex reveals biphasic synaptic protein response during progression of Alzheimer's disease. Am J Pathology 2000; 157(2): 623-636.

Nachum-Biala Y, Troen AM. B-vitamins for neuroprotection: Narrowing the evidence gap. BioFactors 2012; 38(2):145-150.

Neary D, Snowden JS, Gustafson L, *et al*. Frontotemporal lobar degeneration: A consensus on clinical diagnostic criteria. Neurology 1998; 51(6):1546-1554.

O'Leary F, Allman-Farinelli M, Samman S. Vitamin B_{12} status, cognitive decline and dementia: A systematic review of prospective cohort studies. Br J Nutr 2012; 108(11):1948-1961.

Pfeffer RI, Kurosaki TT, Harrah CH Jr., *et al*. Measurement of functional activities in older adults in the community. J Gerontol 1982; 37(3):323–329.

Rademakers R, Cruts M, van Broeckhoven C. The role of tau (MAPT) in frontotemporal dementia and related tauopathies. Hum Mut 2004; 24(4): 277-295.

Rascol O, Goetz C, Koller W, *et al*. Treatment interventions for Parkinson's disease: an evidence based assessment. Lancet 2002; 359(9317):1589-1598.

Rascovsky K, Hodges JR, Kipps CM, *et al*. Diagnostic criteria for the behavioral variant of frontotemporal dementia (bvFTD): current limitations and future directions. Alzheimer Dis Assoc Disord 2007; 21(4):S14–18.

Rascovsky K, Hodges JR, Knopman D, *et al.* Sensitivity of revised diagnostic criteria for the behavioural variant of frontotemporal dementia. Brain 2011; 134(pt 9):2456-2477.

Schneider LS, Olin JT, Doody RS, *et al*. Validity and reliability of the Alzheimer's Disease Cooperative Study-Clinical Global Impression of Change. Alzheimer Dis Assoc Disord 1997; 11(Suppl 2):S22-32.

Shi J, Shaw CL, Du Plessis D, *et al.* Histopathological changes underlying frontotemporal lobar degeneration with clinicopathological correlation. Acta Neuropathol 2005; 110(5): 501-512.

Tombaugh TN, McIntyre NJ. The Mini-Mental State Examination: A comprehensive review. J Am Geriatr Soc 1992; 40(9):922-935.

Torralva T, Roca M, Gliichgerrct E, *et al*. Validation of the Spanish Version of the Addenbrooke's Cognitive Examination Revised (ACE-R). Neuroligia 2011; 26(6):351-356.

WHO. Serum and red blood cell folate concentrations for assessing folate status in populations. Vitamin and Mineral Nutrition Information System. Geneva: World Health Organization, 2012. (http://apps.who.int/iris/bitstream/10665/75584/1/WHO_NMH_NHD_EPG_12.1_eng.pdf, accessed 3 May 2013)

Wischik CM, Edwards PC, Lai RY, *et al*. Selective inhibition of Alzheimer disease-like tau aggregation by phenothiazines. PNAS 1996; 93(20):11213-11218.

Wischik CM, Staff RT, Wischik DJ, *et al*. Tau aggregation inhibition therapy: an exploratory Phase 2 study in mild or moderate Alzheimer's disease. J Alzheimer's Dis 2015; 44:705-720.

Yamashita M, Nonaka T, Arai T, *et al.* Methylene blue and dimebon inhibit aggregation of TDP-43 in cellular models. FEBS Lett 2009; 583(14): 2419-2424.

24. APPENDICES

24.1. International Consensus Criteria for Probable bvFTD (Rascovsky *et al*., 2007)

Criteria for Probable bvFTD

All of the following symptoms [A-C] must be present to meet criteria.

A. Meets criteria for possible bvFTD (listed below under **Criteria for Possible bvFTD**)

B. Exhibits significant functional decline (by caregiver report or as evidenced by CDR or FAQ scores)

C. Imaging results consistent with bvFTD (one of the following must be present):
  - C.1. Frontal and/or anterior temporal atrophy on CT or MRI
  - C.2. Frontal hypoperfusion or hypometabolism on SPECT or PET

Criteria for Possible bvFTD

Three of the following behavioral / cognitive symptoms [A-F] must be present to meet criteria. These symptoms should occur repeatedly, not just as a single instance.

A. Early behavioral disinhibition (one of the following symptoms must be present):
  - A.1. Socially inappropriate behavior
  - A.2. Loss of manners or decorum
  - A.3. Impulsive, rash or careless actions

B. Early apathy or inertia (one of the following symptoms must be present):
  - B.1. Apathy
  - B.2. Inertia

C. Early loss of sympathy or empathy (one of the following symptoms must be present):
  - C.1. Diminished response to other people’s needs and feelings
  - C.2. Diminished social interest, interrelatedness or personal warmth

D. Early perseverative, stereotyped or compulsive/ritualistic behavior (one of the following symptoms [D.1-D.3] must be present):
  - D.1. Simple repetitive movements
  - D.2. Complex, compulsive or ritualistic behaviors
  - D.3. Stereotypy of speech

E. Hyperorality and dietary changes (one of the following symptoms must be present):
  - E.1. Altered food preferences
  - E.2. Binge eating, increased consumption of alcohol or cigarettes
  - E.3. Oral exploration or consumption of inedible objects

F. Neuropsychological profile: executive/generation deficits with relative sparing of memory and visuospatial functions (all of the following symptoms must be present):
  - F.1. Deficits in executive tasks
  - F.2. Relative sparing of episodic memory
  - F.3. Relative sparing of visuospatial skills

24.2. El Escorial Research Criteria for Diagnosis of Amyotrophic Lateral Sclerosis (Brooks *et al*., 2000)

The diagnosis of amyotrophic lateral sclerosis requires the presence of:

- Evidence of lower motor neuron (LMN) degeneration by clinical, electrophysiological or neuropathologic examination,
- Evidence of upper motor neuron (UMN) degeneration by clinical examination, and
- Progressive spread of symptoms or signs within a region or to other regions, as determined by history or examination,

There must also be absence of:

- Electrophysiological or pathological evidence of other disease processes that might explain the signs of LMN and/or UMN degeneration, and
- Neuroimaging evidence of other disease processes that might explain the observed clinical and electrophysiological signs.

Appropriate history, physical and neurological examinations must be performed to identify clinical evidence of UMN and LMN signs in four regions of the central nervous system (CNS): brainstem, cervical, thoracic, or lumbosacral spinal cord. Examples of UMN and LMN signs by each CNS region are provided in the table below.

	Brainstem	Cervical	Thoracic	Lumbosacral
**Lower motor neuron signs** weakness, atrophy, fasciculations	• jaw, face,<br>• palate,<br>• tongue,<br>• larynx	• neck, arm, hand,<br>• diaphragm	• back,<br>• abdomen	• back, abdomen,<br>• leg, foot
**Upper motor neuron signs** pathologic spread of reflexes, clonus, etc.	• clonic jaw jerk,<br>• gag reflex,<br>• exaggerated snout reflex,<br>• pseudobulbar features,<br>• forced yawning,<br>• pathologic DTRs,<br>• spastic tone	• clonic DTRs,<br>• Hoffmann reflex,<br>• pathologic DTRs,<br>• spastic tone,<br>• preserved reflex in weak wasted limb	• loss of superficial abdominal reflexes,<br>• pathologic DTRs,<br>• spastic tone	• clonic DTRs,<br>• extensor plantar response,<br>• pathologic DTRs,<br>• spastic tone,<br>• preserved reflex in weak, wasted limb

Ancillary tests should be applied as clinically indicated to exclude other disease processes. These tests should include electrodiagnostic, neurophysiological, neuroimaging, or clinical laboratory assessments.

For purposes of the study eligibility, the investigator should answer “yes” or “no” to the following after completing the neurological examination: Does the subject meet research criteria (El Escorial) for Amyotrophic Lateral Sclerosis or motor neuron disease?

24.3. Assessments by Visit

Before participation in this study may begin, the subject and caregiver will each be provided with an information sheet and both will be given as much time as needed to decide whether or not to participate in the study.

Screening Visit (Visit 1, Study Day -42 to Study Day -1)

The following assessments will be performed at Screening:

- Written Informed Consent for participation in the study from the subject (and/or legally acceptable representative[s]) and from the identified caregiver(s) (before any study related procedures may be performed)
- Adverse Events review (after signing of the ICF)
- Inclusion/exclusion criteria review
- Demographic information
- Medical history and clinical interview, including details of diagnosis
- Prior and concomitant medication review (details of current and recent medication [within 3 months], including dose changes)
- Confirmation of bvFTD diagnosis
- MRI (of sufficient quality, with T1, T2*-weighted gradient-recalled echo and FLAIR sequences), to occur within 42 days prior to Baseline (Visit 2)
- MMSE
- Modified Hachinski Ischemic Score
- El Escorial research criteria for Amyotrophic Lateral Sclerosis
- Complete physical examination and neurological examination
- Blood pressure and pulse measured in a seated position for at least 5 minutes and then repeated 2 minutes after standing
- Respiratory rate and preferably oral (sublingual) temperature (aural temperature is an acceptable, but less preferable, alternative)
- Weight and height
- 12-lead electrocardiogram (ECG) measurements performed in triplicate (three ECG tracings within an approximate 2- to 5-minute interval)
- Blood samples for the following:
    - Chemistry panel
    - Hematology panel
    - G6PD, TSH, haptoglobin, vitamin B_{12}, and folate
    - Serum pregnancy test for women of childbearing potential
- Urine sample for urinalysis

- Methemoglobin and oxygen saturation by pulse co-oximetry
- C-SSRS

If a subject is considered potentially eligible for participation in this study, then the subject's primary medical provider will be notified.

Baseline (Visit 2, Day 1)

After the Screening assessments, subjects who are considered eligible for participation in this study will return to the study site within 42 days for Baseline assessments and randomization.

Baseline efficacy assessments may be made on the day before randomization and dosing if necessary.

Before Dose Assessments

The following will be performed prior to dosing:

- Inclusion and exclusion criteria review (to confirm continued eligibility)
- Blood pressure and pulse measured within 1 hour prior to dosing with subject in a seated position for at least 5 minutes and then repeated 2 minutes after standing
- Respiratory rate and preferably oral (sublingual) temperature (aural temperature is an acceptable, but less preferable, alternative) measured within 1 hour prior to dosing
- Weight
- Concomitant medication recording/review
- Adverse event review
- Blood samples for the following Baseline tests:
    - Chemistry panel
    - Hematology panel
    - Serum pregnancy test for women of childbearing potential
    - Vitamin B_{12} and folate
- Urine sample for urinalysis
- Methemoglobin and oxygen saturation by pulse co-oximetry within 1 hour prior to dosing
- 12-lead electrocardiogram measurements performed in triplicate (three ECG tracings within an approximate 2- to 5-minute interval) (dosing may be held subject to eligibility review based on the local interpretation of triplicate ECGs or the results of the central interpretation in the event of deviations from Screening ECG detected at Baseline and considered clinically significant by the investigator)
- Targeted physical and neurological examinations within 1 hour prior to dosing focused on deep-tendon reflexes, clonus, and muscle rigidity; size and reactivity of pupils; dryness of oral mucosa; intensity of bowel sounds; skin color; and presence or absence of diaphoresis.

- Serotonin Toxicity (Syndrome) Diagnostic Interview and Rating Guide (In-Clinic)
- Blood sample for MT concentration (at sites with refrigerated centrifuge and appropriate freezer storage capability)
- Blood sample for genotyping (optional; only from those subjects by or for whom legally acceptable consent is provided); may be collected at any time after eligibility for continued participation in the study has been confirmed
- ACE-R
- ACE-III (additional items)
- Modified ADCS-CGIC
- FRS
- FAQ
- UPDRS Parts II and III

Post Dose Assessments

All subjects are to remain in the clinic for at least 4 hours after the first dose. The following will be performed after randomization and dosing are completed at the times indicated:

- Recording of adverse events (throughout in-clinic observation)
- Respiratory rate and preferably oral (sublingual) temperature (aural temperature is an acceptable, but less preferable, alternative) (hourly until discharge)
- Blood pressure and pulse measured with subject in a seated position for at least 5 minutes and then repeated 2 minutes after standing) (approximately 2 hours post-dose)
- Concomitant medication recording/review
- Methemoglobin and oxygen saturation by pulse co-oximetry (approximately 2.5 hours post-dose)
- 12-lead electrocardiogram measurements performed in triplicate (three ECG tracings within an approximate 2- to 5-minute interval) (approximately 3 hours post-dose)
- Targeted physical and neurological examinations focused on deep-tendon reflexes, clonus, and muscle rigidity; size and reactivity of pupils; dryness of oral mucosa; intensity of bowel sounds; skin color; and presence or absence of diaphoresis (approximately 3 hours post-dose following ECG measurements); these are to be repeated as needed for subjects who remain in clinic longer than 4 hours
- C-SSRS (before discharge from the clinic)
- Blood sample for MT concentration (approximately 3.5 hours post-dose following ECG measurements and physical and neurological examinations); time to be recorded (at sites with refrigerated centrifuge and appropriate freezer storage capability)
- Serotonin toxicity assessment just prior to discharge from the clinic, *i.e.,* approximately 4 hours post-dose

- Any additional assessments required to monitor a treatment-emergent AE to resolution or stabilization

The subject is to be scheduled to return to the clinic for Visit 3 (4 weeks ± 3 days from Baseline). The subject and caregiver will be provided with information about how to correctly take study drug. Study drug will be dispensed to the subject and/or the subject's caregiver. Enough study drug will be dispensed to last until Visit 4; subjects and caregivers will be instructed to bring supplies to the clinic for the Week 4 visit (Visit 3).

At discharge from the study unit, subjects (and their caregivers) using any medication with the potential to increase synaptic levels of serotonin (see Section 4.7.2) will be instructed on signs and symptoms of potential serotonergic toxicity and given a thermometer to measure the subject's temperature three times a day (in the morning, afternoon, and evening) until 72 hours after the first dose of study drug. Each measurement will be recorded in a diary to be returned to the site at Visit 3. They are to remain in close proximity to a local hospital and remain in telephone contact with the clinic for at least 12 – 14 hours post-dose; reimbursement for accommodations will be made available if requested by the subject and/or caregiver.

Post-Dose Telephone Contacts (5–7, >7–14, >14–24, 44–52, and 68–76 Hours after First Dose)

For subjects who are receiving products with serotonergic potential, their caregivers are to be contacted by telephone 5–7, >7–14, >14–24, 44–52, and 68–76 hours after the first dose of study drug (with a minimum of 1 hour between contacts) for assessment of potential serotonin toxicity; if indicated, more frequent contacts will be made. They will be informed to have the temperature diary available for these telephone contacts. Instructions for the phone interview are provided in Section 24.8.

Visit 3 (Week 4 ± 3 Days)

The subjects are to bring all remaining study drug to the clinic to assess compliance. Subjects using any medication with the potential to increase synaptic levels of serotonin are to return their temperature diary to the clinic at this visit.

The following assessments will be performed:

- Vital sign measurements (blood pressure and pulse measured with subject in a seated position for at least 5 minutes)
- Respiratory rate and preferably oral (sublingual) temperature (aural temperature is an acceptable, but less preferable, alternative)
- Methemoglobin and oxygen saturation by pulse co-oximetry
- Targeted physical and neurological examinations focused on deep-tendon reflexes, clonus, and muscle rigidity; size and reactivity of pupils; dryness of oral mucosa; intensity of bowel sounds; skin color; and presence or absence of diaphoresis
- Serotonin Toxicity (Syndrome) Diagnostic Interview and Rating Guide (In-Clinic)
- 12-lead electrocardiogram (single recording, unless there is an emergent abnormality which is of clinical significance in the judgment of the investigator or for subjects with

well controlled atrial fibrillation (heart rate ≤ 84 bpm and appropriate anticoagulation) at Baseline, in which cases triplicate ECG recordings will be obtained)

- Recording of adverse events
- Concomitant medication review and recording (*N.B.*, if initiation of a drug with serotonergic potential is considered, the first dose must be given in the clinic and the monitoring outlined on Day 1 followed, *i.e.*, the subject must remain in the clinic for at least 4 hours with close monitoring, temperature must be measured following discharge at least three times daily with repeated telephone contacts with the clinic until 68–76 hours post-dose.)
- C-SSRS
- Blood samples will be obtained for the following tests:
  - Chemistry panel
  - Hematology panel
  - Serum pregnancy test for women of childbearing potential
  - Vitamin B_{12} and folate
- Blood sample for MT concentration; time to be recorded (at sites with refrigerated centrifuge and appropriate freezer storage capability)
- Urine sample for urinalysis
- Any additional assessments required to monitor a treatment-emergent AE to resolution or stabilization

The subject is to be scheduled to return to the clinic for Visit 4 (8 weeks ± 7 days from Baseline). Since enough study drug was dispensed at Visit 2 to last until Week 8 (Visit 4), the subject and/or the caregiver will not be dispensed study drug during this visit.

Visit 4 (Study Week 8 ± 7 Days)

The subjects are to bring all remaining study drug to the clinic to assess compliance.

The following assessments will be performed:

- Respiratory rate and preferably oral (sublingual) temperature (aural temperature is an acceptable, but less preferable, alternative)
- Blood pressure and pulse measured with subject in a seated position for at least 5 minutes
- Weight
- Blood samples will be obtained for the following tests:
  - Chemistry panel
  - Hematology panel
  - Serum pregnancy test for women of childbearing potential
  - Vitamin B_{12} and folate

- Blood sample for MT concentration; time to be recorded (at sites with refrigerated centrifuge and appropriate freezer storage capability)
- Urine sample for urinalysis
- Methemoglobin and oxygen saturation by pulse co-oximetry
- 12-lead electrocardiogram (single recording, unless there is an emergent abnormality which is of clinical significance in the judgment of the investigator or for subjects with well controlled atrial fibrillation (heart rate ≤ 84 bpm and appropriate anticoagulation) at Baseline, in which cases triplicate ECG recordings will be obtained)
- Targeted physical and neurological examination
- Serotonin Toxicity (Syndrome) Diagnostic Interview and Rating Guide (In-Clinic)
- Recording of adverse events
- Concomitant medication review and recording (*N.B.*, if initiation of a drug with serotonergic potential is considered, the first dose must be given in the clinic and the monitoring outlined on Day 1 followed, *i.e.*, the subject must remain in the clinic for at least 4 hours with close monitoring, temperature must be measured following discharge at least three times daily with repeated telephone contacts with the clinic until 68–76 hours post-dose.)
- C-SSRS
- Modified ADCS-CGIC
- Any additional assessments required to monitor a treatment-emergent AE to resolution or stabilization

The subject is to be scheduled to return to the clinic for Visit 5 (16 weeks ± 7 days from Baseline). The subject and caregiver will be provided with information about how to correctly take study drug. Study drug will be dispensed to the subject and/or the subject’s caregiver. Enough study drug will be dispensed to last until the Week 16 visit (Visit 5).

Subjects’ caregivers are to be contacted by telephone after 12 weeks (± 7 days) for an assessment of safety. An unscheduled visit is to take place if needed in response to a safety concern.

Visit 5 (Study Week 16 ± 7 Days)

The subjects are to bring all remaining study drug to the clinic to assess compliance.

The following assessments will be performed:

- Respiratory rate and preferably oral (sublingual) temperature (aural temperature is an acceptable, but less preferable, alternative)
- Blood pressure and pulse measured with subject in a seated position for at least 5 minutes
- Weight
- Blood samples will be obtained for the following tests:

  - Chemistry panel
  - Hematology panel
  - Serum pregnancy test for women of childbearing potential
  - Vitamin B_{12} and folate

- Blood sample for MT concentration; time to be recorded (at sites with refrigerated centrifuge and appropriate freezer storage capability)
- Urine sample for urinalysis
- Methemoglobin and oxygen saturation by pulse co-oximetry
- 12-lead electrocardiogram (single recording, unless there is an emergent abnormality which is of clinical significance in the judgment of the investigator or for subjects with well controlled atrial fibrillation (heart rate ≤ 84 bpm and appropriate anticoagulation) at Baseline, in which cases triplicate ECG recordings will be obtained)
- Targeted physical and neurological examinations
- Serotonin Toxicity (Syndrome) Diagnostic Interview and Rating Guide (In-Clinic)
- Recording of adverse events
- Concomitant medication review and recording (*N.B.*, if initiation of a drug with serotonergic potential is considered, the first dose must be given in the clinic and the monitoring outlined on Day 1 followed, *i.e.*, the subject must remain in the clinic for at least 4 hours with close monitoring, temperature must be measured following discharge at least three times daily with repeated telephone contacts with the clinic until 68–76 hours post-dose.)
- C-SSRS
- ACE-R
- ACE-III (additional items)
- Modified ADCS-CGIC
- FRS
- FAQ
- UPDRS Parts II and III
- MRI (with T1, T2*-weighted gradient-recalled echo and FLAIR sequences) (a time window of ± 14 days will be allowed)
- Any additional assessments required to monitor a treatment-emergent AE to resolution or stabilization

The subject is to be scheduled to return to the clinic for Visit 6 (24 weeks ± 7 days from Baseline). The subject and caregiver will be provided with information about how to correctly take study drug. Study drug will be dispensed to the subject and/or the subject’s caregiver. Enough study drug will be dispensed to last until the Week 24 visit (Visit 6).

Subjects' caregivers are to be contacted by telephone after 20 weeks (± 7 days) for an assessment of safety. An unscheduled visit is to take place if needed in response to a safety concern.

Visit 6 (Week 24 ± 7 Days)

The subjects are to bring all remaining study drug to the clinic to assess compliance.

The following assessments will be performed:

- Respiratory rate and preferably oral (sublingual) temperature (aural temperature is an acceptable, but less preferable, alternative)
- Blood pressure and pulse measured with subject in a seated position for at least 5 minutes
- Weight
- Blood samples will be obtained for the following tests:
    - Chemistry panel
    - Hematology panel
    - Vitamin B_{12} and folate
    - TSH
    - Serum pregnancy test for women of childbearing potential
- Blood sample for MT concentration; time to be recorded (at sites with refrigerated centrifuge and appropriate freezer storage capability)
- Urine sample for urinalysis
- Methemoglobin and oxygen saturation by pulse co-oximetry
- 12-lead electrocardiogram (single recording, unless there is an emergent abnormality which is of clinical significance in the judgment of the investigator or for subjects with well controlled atrial fibrillation (heart rate ≤ 84 bpm and appropriate anticoagulation) at Baseline, in which cases triplicate ECG recordings will be obtained)
- Targeted physical and neurological examinations
- Serotonin Toxicity (Syndrome) Diagnostic Interview and Rating Guide (In-Clinic)
- Recording of adverse events
- Concomitant medication review and recording (*N.B.*, if initiation of a drug with serotonergic potential is considered, the first dose must be given in the clinic and the monitoring outlined on Day 1 followed, *i.e.*, the subject must remain in the clinic for at least 4 hours with close monitoring, temperature must be measured following discharge at least three times daily with repeated telephone contacts with the clinic until 68–76 hours post-dose.)
- C-SSRS
- Any additional assessments required to monitor a treatment-emergent AE to resolution or stabilization

The subject is to be scheduled to return to the clinic for Visit 7 (32 weeks ± 7 days from Baseline). The subject and caregiver will be provided with information about how to correctly take study drug. Study drug will be dispensed to the subject and/or the subject's caregiver. Enough study drug will be dispensed to last until the Week 32 visit (Visit 7).

Subjects' caregivers are to be contacted by telephone after 28 weeks (± 7 days) for an assessment of safety. An unscheduled visit is to take place if needed in response to a safety concern.

Visit 7 (Week 32 ± 7 Days)

The subjects are to bring all remaining study drug to the clinic to assess compliance.

The following assessments will be performed:

- Respiratory rate and preferably oral (sublingual) temperature (aural temperature is an acceptable, but less preferable, alternative)
- Blood pressure and pulse measured with subject in a seated position for at least 5 minutes
- Weight
- Blood samples will be obtained for the following tests:
  - Chemistry panel
  - Hematology panel
  - Vitamin B_{12} and folate
  - Serum pregnancy test for women of childbearing potential
- Blood sample for MT concentration; time to be recorded (at sites with refrigerated centrifuge and appropriate freezer storage capability)
- Urine sample for urinalysis
- Methemoglobin and oxygen saturation by pulse co-oximetry
- 12-lead electrocardiogram (single recording, unless there is an emergent abnormality which is of clinical significance in the judgment of the investigator or for subjects with well controlled atrial fibrillation (heart rate ≤ 84 bpm and appropriate anticoagulation) at Baseline, in which cases triplicate ECG recordings will be obtained)
- Targeted physical and neurological examinations
- Recording of adverse events
- Serotonin Toxicity (Syndrome) Diagnostic Interview and Rating Guide (In-Clinic)
- Concomitant medication review and recording (*N.B.*, if initiation of a drug with serotonergic potential is considered, the first dose must be given in the clinic and the monitoring outlined on Day 1 followed, *i.e.*, the subject must remain in the clinic for at least 4 hours with close monitoring, temperature must be measured following discharge at least three times daily with repeated telephone contacts with the clinic until 68–76 hours post-dose.)
- C-SSRS

- ACE-R
- ACE-III (additional items)
- Modified ADCS-CGIC
- FRS
- FAQ
- UPDRS Parts II and III
- MRI (with T1, T2*-weighted gradient-recalled echo and FLAIR sequences) (a time window of ± 14 days will be allowed)
- Any additional assessments required to monitor a treatment-emergent AE to resolution or stabilization

The subject is to be scheduled to return to the clinic for Visit 8 (42 weeks ± 7 days from Baseline). The subject and caregiver will be provided with information about how to correctly take study drug. Study drug will be dispensed to the subject and/or the subject's caregiver. Enough study drug will be dispensed to last until the Week 42 visit (Visit 8).

Visit 8 (Week 42 ± 7 Days)

The subjects are to bring all remaining study drug to the clinic to assess compliance.

The following assessments will be performed:

- Respiratory rate and preferably oral (sublingual) temperature (aural temperature is an acceptable, but less preferable, alternative)
- Blood pressure and pulse measured with subject in a seated position for at least 5 minutes
- Weight
- Blood samples will be obtained for the following tests:
    - Chemistry panel
    - Hematology panel
    - Vitamin B_{12} and folate
    - Serum pregnancy test for women of childbearing potential
- Blood sample for MT concentration; time to be recorded (at sites with refrigerated centrifuge and appropriate freezer storage capability)
- Urine sample for urinalysis
- Methemoglobin and oxygen saturation by pulse co-oximetry
- 12-lead electrocardiogram (single recording, unless there is an emergent abnormality which is of clinical significance in the judgment of the investigator or for subjects with well controlled atrial fibrillation (heart rate ≤ 84 bpm and appropriate anticoagulation) at Baseline, in which cases triplicate ECG recordings will be obtained)
- Targeted physical and neurological examinations

- Serotonin Toxicity (Syndrome) Diagnostic Interview and Rating Guide (In-Clinic)
- Recording of adverse events
- Concomitant medication review and recording (*N.B.*, if initiation of a drug with serotonergic potential is considered, the first dose must be given in the clinic and the monitoring outlined on Day 1 followed, *i.e.*, the subject must remain in the clinic for at least 4 hours with close monitoring, temperature must be measured following discharge at least three times daily with repeated telephone contacts with the clinic until 68–76 hours post-dose.)
- C-SSRS
- Any additional assessments required to monitor a treatment-emergent AE to resolution or stabilization

The subject is to be scheduled to return to the clinic for Visit 9 (52 weeks ± 14 days from Baseline). The subject and caregiver will be provided with information about how to correctly take study drug. Study drug will be dispensed to the subject and/or the subject's caregiver. Enough study drug will be dispensed to last until the Week 52 visit (Visit 9).

Visit 9 (Week 52 ± 14 Days) – End of Study (or Early Termination) Visit

The subjects are to bring all remaining study drug to the clinic to assess compliance.

The following assessments will be performed:

- Respiratory rate and preferably oral (sublingual) temperature (aural temperature is an acceptable, but less preferable, alternative)
- Blood pressure and pulse measured with subject in a seated position for at least 5 minutes
- Weight
- Blood samples will be obtained for the following tests:
  - Chemistry panel
  - Hematology panel
  - Vitamin B_{12} and folate
  - TSH
  - Serum pregnancy test for women of childbearing potential
- Blood sample for MT concentration; time to be recorded (at sites with refrigerated centrifuge and appropriate freezer storage capability)
- Urine sample for urinalysis
- Methemoglobin and oxygen saturation by pulse co-oximetry
- 12-lead electrocardiogram (single recording, unless there is an emergent abnormality which is of clinical significance in the judgment of the investigator or for subjects with well controlled atrial fibrillation (heart rate ≤ 84 bpm and appropriate anticoagulation) at Baseline, in which cases triplicate ECG recordings will be obtained)

- Targeted physical and neurological examinations
- Serotonin Toxicity (Syndrome) Diagnostic Interview and Rating Guide (In-Clinic)
- Recording of adverse events
- Concomitant medication review and recording (*N.B.*, if initiation of a drug with serotonergic potential is considered, the first dose must be given in the clinic and the monitoring outlined on Day 1 followed, *i.e.*, the subject must remain in the clinic for at least 4 hours with close monitoring, temperature must be measured following discharge at least three times daily with repeated telephone contacts with the clinic until 68–76 hours post-dose.)
- C-SSRS
- ACE-R (MMSE included and also reported separately)
- ACE-III (additional items)
- Modified ADCS-CGIC
- FRS
- FAQ
- UPDRS Parts II and III
- MRI (with T1, T2*-weighted gradient-recalled echo and FLAIR sequences) (a time window of ± 14 days will be allowed)
- Any additional assessments required to monitor a treatment-emergent AE to resolution or stabilization

Depending on tolerability and response, the subject may be offered the option of entering the open-label extension study. Otherwise, a follow-up visit should be scheduled for approximately 28 days after the last dose of study drug.

Unscheduled Visit

The subjects are to bring all remaining study drug to the clinic to assess compliance.

The following assessments are to be performed as appropriate in response to the safety concern:

- Respiratory rate and preferably oral (sublingual) temperature (aural temperature is an acceptable, but less preferable, alternative)
- Blood pressure and pulse measured with subject in a seated position for at least 5 minutes
- Weight
- Blood samples will be obtained for the following tests:
    - Chemistry panel
    - Hematology panel
    - Vitamin B_{12} and folate
    - Serum pregnancy test for women of childbearing potential

- Blood sample for MT concentration; time to be recorded (at sites with refrigerated centrifuge and appropriate freezer storage capability)
- Urine sample for urinalysis
- Methemoglobin and oxygen saturation by pulse co-oximetry
- 12-lead electrocardiogram (single recording, unless there is an emergent abnormality which is of clinical significance in the judgment of the investigator or for subjects with well controlled atrial fibrillation (heart rate ≤ 84 bpm and appropriate anticoagulation) at Baseline, in which cases triplicate ECG recordings will be obtained)
- Targeted physical and neurological examinations
- Serotonin Toxicity (Syndrome) Diagnostic Interview and Rating Guide (In-Clinic)
- Recording of adverse events
- Concomitant medication review and recording (*N.B.*, if initiation of a drug with serotonergic potential is considered, the first dose must be given in the clinic and the monitoring outlined on Day 1 followed*, i.e.*, the subject must remain in the clinic for at least 4 hours with close monitoring, temperature must be measured following discharge at least three times daily with repeated telephone contacts with the clinic until 68–76 hours post-dose.)
- C-SSRS
- Modified ADCS-CGIC
- Any additional assessments required to monitor a treatment-emergent AE to resolution or stabilization

The subject is to be scheduled to return to the clinic for next scheduled visit or additional unscheduled visits as appropriate.

Visit 10 (28 Days [± 7 Days] Off-treatment) – Safety Follow-up Visit (If Applicable)

The following assessments will be performed in subjects who discontinue early or do not enter the open-label extension study (and any other assessments necessary to follow the resolution of a treatment-emergent adverse event):

- Respiratory rate and preferably oral (sublingual) temperature (aural temperature is an acceptable, but less preferable, alternative)
- Blood pressure and pulse measured with subject in a seated position for at least 5 minutes
- Weight
- Blood samples will be obtained for the following tests:
    - Chemistry panel
    - Hematology panel
    - Vitamin B_{12} and folate
    - Serum pregnancy test for women of childbearing potential

- Urine sample for urinalysis
- Methemoglobin and oxygen saturation by pulse co-oximetry
- 12-lead electrocardiogram (single recording, unless there is an emergent abnormality which is of clinical significance in the judgment of the investigator or for subjects with well controlled atrial fibrillation (heart rate ≤ 84 bpm and appropriate anticoagulation) at Baseline, in which cases triplicate ECG recordings will be obtained)
- Targeted physical and neurological examinations focused on any change in medical history
- Serotonin Toxicity (Syndrome) Diagnostic Interview and Rating Guide (In-Clinic)
- Recording of adverse events
- Concomitant medication review and recording
- C-SSRS

24.4. Addenbrooke's Cognitive Examination-Revised (ACE-R)

ADDENBROOKE'S COGNITIVE EXAMINATION - ACE-R

Final Revised Version A (2005)

Name :
Date of birth :
Hospital no. :

Addressograph

Date of testing: .... /........ /........
Tester's name: ....
Age at leaving full-time education: ....
Occupation: ....
Handedness: ....

ATTENTION & ORIENTATION

ORIENTATION

➢ Ask: What is the	Day	Date	Month	Year	Season	[Score 0-5]
➢ Ask: Which	Building	Floor	Town	County	Country	[Score 0-5]

REGISTRATION

➢ Tell: 'I'm going to give you three words and i'd like you to repeat after me: lemon, key and ball'. After subject repeats, say 'Try to remember them because i'm going to ask you later'. Score only the first trial (repeat 3 times if necessary).

Register number of trials ....

[Score 0-3]

ATTENTION & CONCENTRATION

➢ Ask the subject: ' could you take 7 away from a 100? After the subject responds, ask him or her to take away another 7 to a total of 5 subtractions. If subject make a mistake, carry on and check the subsequent answer (i.e. 93, 84, 77, 70, 63 -score 4)

Stop after five subtractions (93, 86, 79, 72, 65). ....

➢ Ask: 'could you please spell **WORLD** for me? Then ask him/her to spell it backwards:

....

[Score 0-5] (for the best performed task)

MEMORY

MEMORY - Recall

➢ Ask: 'Which 3 words did I ask you to repeat and remember?'

....

[Score 0-3]

MEMORY - Anterograde Memory

➢ Tell: ' I'm going to give you a name and address and I'd like you to repeat after me. We'll be doing that 3 times, so you have a chance to learn it. I'll be asking you later'

Score only the third trial

[Score 0-7]

	1st Trial	2nd Trial	3rd Trial
Harry Barnes	....	....	....
73 Orchard Close	....	....	....
Kingsbridge	....	....	....
Devon	....	....	....

MEMORY - Retrograde Memory

➢ Name of current Prime Minister ....
➢ Name of the woman who was Prime Minister ....
➢ Name of the USA president ....
➢ Name of the USA president who was assassinated in the 1960's ....

[Score 0 -4]

copyright 2000, John R. Hodges

ADDENBROOKE'S COGNITIVE EXAMINATION - ACE-R

Final Revised Version (2005)

VERBAL FLUENCY - Letter 'P' and animals

FLUENCY

➢ **Letters**

Say: 'I'm going to give you a letter of the alphabet and I'd like you to generate as many words as you can beginning with that letter, but not names of people or places. Are you ready? You've got a minute and the letter is P'

[Score 0 - 7]

>17	7
14-17	6
11-13	5
8-10	4
6-7	3
4-5	2
2-3	1
<2	0
total	correct

➢ **Animals**

Say: 'Now can you name as many animals as possible, beginning with any letter?

[Score 0 - 7]

>21	7
17-21	6
14-16	5
11-13	4
9-10	3
7-8	2
5-6	1
<5	0
total	correct

LANGUAGE - Comprehension

LANGUAGE

➢ Show written instruction:

[Score 0-1]

Close your eyes

➢ 3 stage command:

'Take the paper in your right hand. Fold the paper in half. Put the paper on the floor'

[Score 0-3]

LANGUAGE - Writing

➢ Ask the subject to make up a sentence and write it in the space below:

Score 1 if sentence contains a subject and a verb (see guide for examples)

[Score 0-1]

copyright 2000, John R. Hodges

ADDENBROOKE'S COGNITIVE EXAMINATION - ACE-R *Final Revised Version (2005)*

LANGUAGE - Repetition

➢ Ask the subject to repeat: **' hippopotamus'; 'eccentricity; 'unintelligible'; 'statistician'** Score 2 if all correct; 1 if 3 correct; 0 if 2 or less.	[Score 0-2]
➢ Ask the subject to repeat: **'Above, beyond and below'**	[Score 0-1]
➢ Ask the subject to repeat: **'No ifs, ands or buts'**	[Score 0-1]

LANGUAGE - Naming

➢ Ask the subject to name the following pictures:

[Score 0-2] pencil + watch

[Score 0-10]

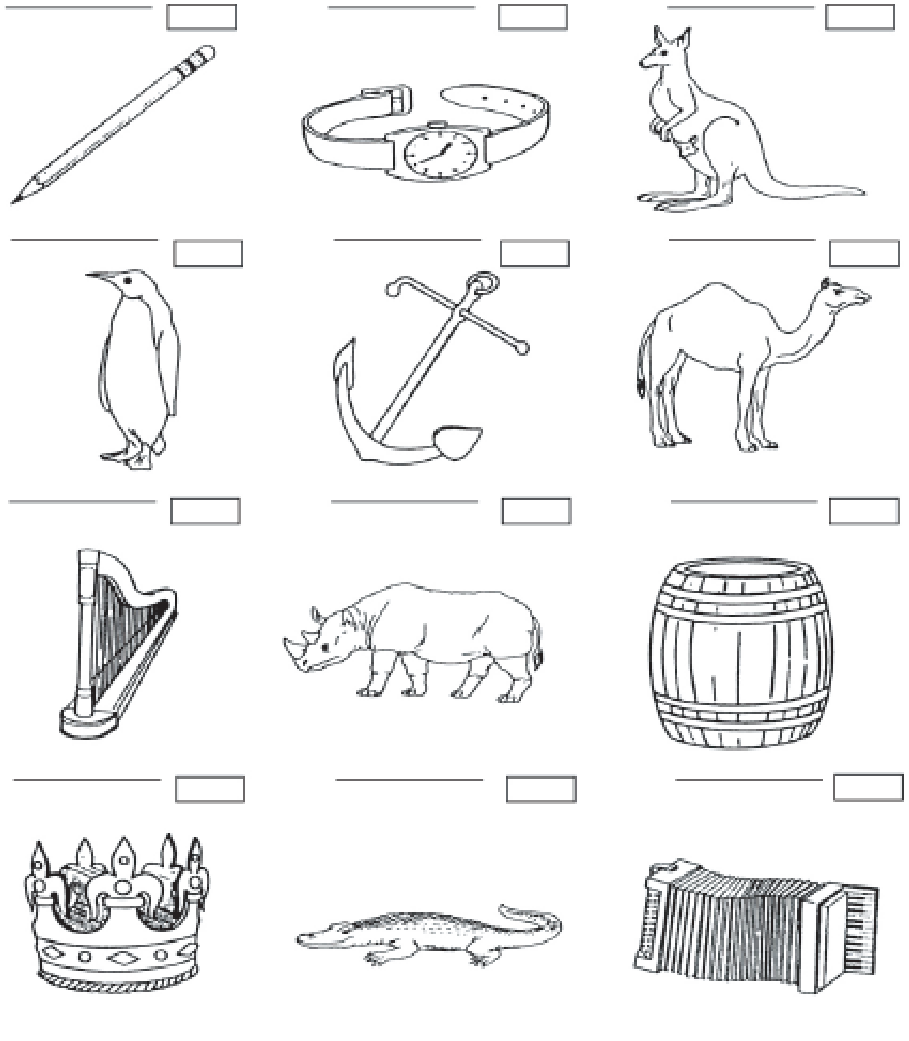

LANGUAGE - Comprehension

➢ Using the pictures above, ask the subject to:

- Point to the one which is associated with the monarchy ______
- Point to the one which is a marsupial ______
- Point to the one which is found in the Antarctic ______
- Point to the one which has a nautical connection ______

[Score 0-4]

LANGUAGE

copyright 2000, John R. Hodges

ADDENBROOKE'S COGNITIVE EXAMINATION - ACE-R *Final Revised Version (2005)*

LANGUAGE - Reading

➢ Ask the subject to read the following words: [Score 1 only if all correct] [Score 0-1]

sew
pint
soot
dough
height

LANGUAGE

VISUOSPATIAL ABILITIES

➢ Overlapping pentagons: Ask the subject to copy this diagram: [Score 0-1]

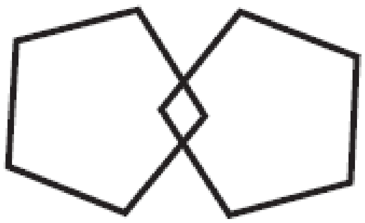

➢ Wire cube : Ask the subject to copy this drawing (for scoring, see instructions guide) [Score 0-2]

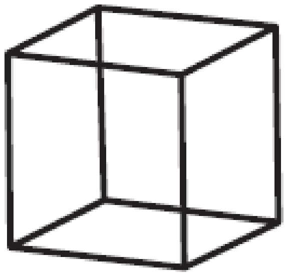

➢ Clock: Ask the subject to draw a clock face with numbers and the hands at ten past five. (for scoring see instruction guide: circle = 1, numbers = 2, hands = 2 if all correct) [Score 0-5]

VISUOSPATIAL

ADDENBROOKE'S COGNITIVE EXAMINATION - ACE-R *Final Revised Version (2005)*

PERCEPTUAL ABILITIES

VISUOSPATIAL

➢ Ask the subject to count the dots without pointing them

[Score 0-4]

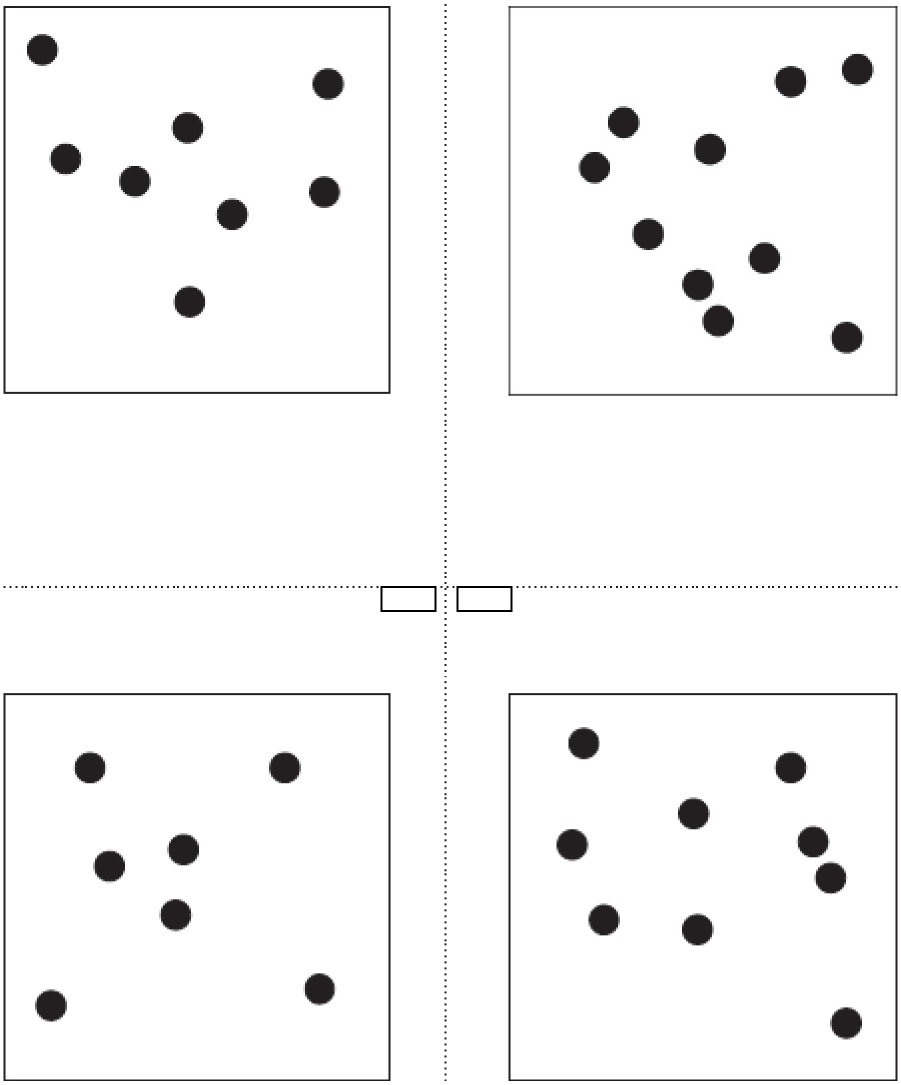

copyright 2000, John R. Hodges

ADDENBROOKE'S COGNITIVE EXAMINATION - ACE-R *Final Revised Version A (2006)*

PERCEPTUAL ABILITIES

➢ Ask the subject to identify the letters [Score 0-4]

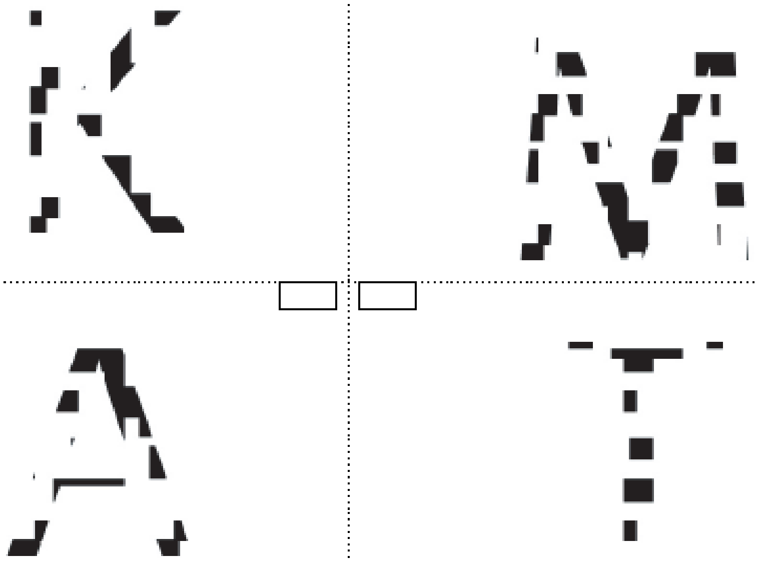

VISUOSPATIAL

RECALL

➢ Ask "Now tell me what you remember of that name and address we were repeating at the beginning"

Harry Barnes	………… …………	[Score 0-7]
73 Orchard Close	……. ………… …………	
Kingsbridge	…………	
Devon	…………	

RECOGNITION

➢ This test should be done if subject failed to recall one or more items. If all items were recalled, skip the test and score 5. If only part is recalled start by ticking items recalled in the shadowed column on the right hand side. Then test not recalled items by telling "ok, I'll give you some hints: was the name X, Y or Z?" and so on. Each recognised item scores one point which is added to the point gained by recalling. [Score 0-5]

Jerry Barnes		Harry Barnes		Harry Bradford		recalled	
37		73		76		recalled	
Orchard Place		Oak Close		Orchard Close		recalled	
Oakhampton		Kingsbridge		Dartington		recalled	
Devon		Dorset		Somerset		recalled	

MEMORY

General Scores

MMSE	/30
ACE-R	/100

Subscores

Attention and Orientation	/18
Memory	/26
Fluency	/14
Language	/26
Visuospatial	/16

SCORE

Normative values based on 63 controls aged 52-75 and 142 dementia patients aged 46-86

Cut-off <88 gives 94% senstivity and 89% specificity for dementia

Cut-off <82 gives 84% sensitivity and 100% specificity for dementia

copyright 2000, John R. Hodges

24.5. Addenbrooke's Cognitive Examination-III (ACE-III)

ADDENBROOKE'S COGNITIVE EXAMINATION – ACE-III
English Version A (2012)

Name:<br>Date of Birth:<br>Hospital No. or Address:	Date of testing: ___/___/___<br>Tester's name:__________<br>Age at leaving full-time education:__________<br>Occupation: __________<br>Handedness: __________

ATTENTION

➢ Ask: What is the	Day	Date	Month	Year	Season	Attention [Score 0-5]
➢ Ask: Which	No./Floor	Street/Hospital	Town	County	Country	Attention [Score 0-5]

ATTENTION

- ➢ Tell: "I'm going to give you three words and I'd like you to repeat them after me: lemon, key and ball." After subject repeats, say "Try to remember them because I'm going to ask you later".
- ➢ Score *only* the first trial (repeat 3 times if necessary).
- ➢ Register number of trials: _____

Attention [Score 0-3]

ATTENTION

- ➢ Ask the subject: "Could you take 7 away from 100? I'd like you to keep taking 7 away from each new number until I tell you to stop."
- ➢ If subject makes a mistake, do not stop them. Let the subject carry on and check subsequent answers (e.g., 93, 84, 77, 70, 63 – score 4).
- ➢ Stop after five subtractions (93, 86, 79, 72, 65): ____ ____ ____ ____ ____

Attention [Score 0-5]

MEMORY

- ➢ Ask: 'Which 3 words did I ask you to repeat and remember?' _______ _______ _______

Memory [Score 0-3]

FLUENCY

➢ **Letters**

Say: "I'm going to give you a letter of the alphabet and I'd like you to generate as many words as you can beginning with that letter, but not names of people or places. For example, if I give you the letter "C", you could give me words like "cat, cry, clock" and so on. But, you can't give me words like Catherine or Canada. Do you understand? Are you ready? You have one minute. The letter I want you to use is the letter "P".

Fluency [Score 0 – 7]

total	correct
≥ 18	7
14-17	6
11-13	5
8-10	4
6-7	3
4-5	2
2-3	1
0-1	0

➢ **Animals**

Say: "Now can you name as many animals as possible. It can begin with any letter."

Fluency [Score 0 – 7]

total	correct
≥ 22	7
17-21	6
14-16	5
11-13	4
9-10	3
7-8	2
5-6	1
<5	0

MEMORY

- Tell: "I'm going to give you a name and address and I'd like you to repeat the name and address after me. So you have a chance to learn, we'll be doing that 3 times. I'll ask you the name and address later."

Score only the third trial.

Memory [Score 0 – 7]

	1st Trial	*2nd Trial*	*3rd Trial*
Harry Barnes<br>73 Orchard Close<br>Kingsbridge<br>Devon	______ ______<br>____ ______ ______<br>____________<br>____________	______ ______<br>____ ______ ______<br>____________<br>____________	______ ______<br>____ ______ ______<br>____________<br>____________

MEMORY

- Name of the current Prime Minister...
- Name of the woman who was Prime Minister ....
- Name of the USA president...
- Name of the USA president who was assassinated in the 1960s...

Memory [Score 0 – 4]

LANGUAGE

- Place a pencil and a piece of paper in front of the subject. As a practice trial, ask the subject to "**Pick up the pencil and then the paper**." If incorrect, score 0 and do not continue further.
- If the subject is correct on the practice trial, continue with the following three commands below.
  - Ask the subject to "**Place the paper on top of the pencil**"
  - Ask the subject to "**Pick up the pencil but not the paper**"
  - Ask the subject to "**Pass me the pencil after touching the paper**"

Note: Place the pencil and paper in front of the subject before each command.

Language [Score 0-3]

LANGUAGE

- Ask the subject to write two (or more) complete sentences about his/her last holiday/weekend/Christmas. Write in complete sentences and do not use abbreviations. Give 1 point if there are two (or more) complete sentences about the one topic; and give another 1 point if grammar and spelling are correct.

Language [Score 0-2]

LANGUAGE

- Ask the subject to repeat: '**caterpillar**'; '**eccentricity**; '**unintelligible**'; '**statistician**'

Score 2 if all are correct; score 1 if 3 are correct; and score 0 if 2 or less are correct.

Language [Score 0-2]

Updated 20/11/2012

LANGUAGE

➢ Ask the subject to repeat: '**All that glitters is not gold**'	**Language** [Score 0-1]
➢ Ask the subject to repeat: '**A stitch in time saves nine**'	**Language** [Score 0-1]

LANGUAGE

➢ Ask the subject to name the following pictures:

Language [Score 0-12]

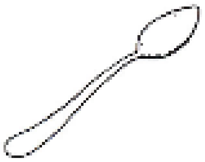
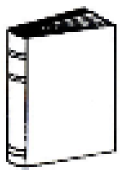
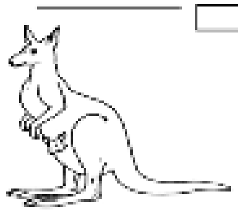
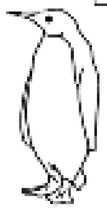
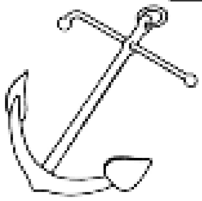
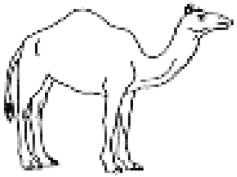
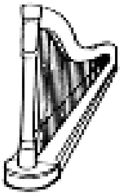
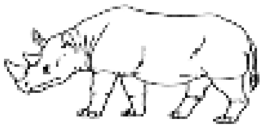
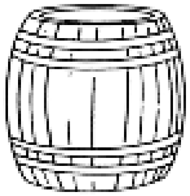
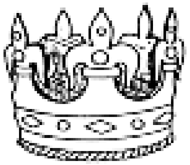
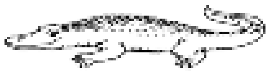
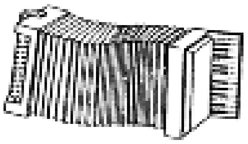

LANGUAGE

➢ Using the pictures above, ask the subject to:

- Point to the one which is associated with the monarchy ....
- Point to the one which is a marsupial ....
- Point to the one which is found in the Antarctic ....
- Point to the one which has a nautical connection ....

Language [Score 0-4]

Updated 20/11/2012

LANGUAGE	
➢ Ask the subject to read the following words: (Score 1 only if all correct)<br><br>**sew**<br>**pint**<br>**soot**<br>**dough**<br>**height**	**Language**<br>[Score 0-1]
**VISUOSPATIAL ABILITIES**	
➢ Infinity Diagram: Ask the subject to copy this diagram	**Visuospatial**<br>[Score 0-1]

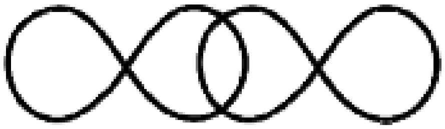

➢ Wire cube: Ask the subject to copy this drawing (for scoring, see instructions guide).	**Visuospatial**<br>[Score 0-2]

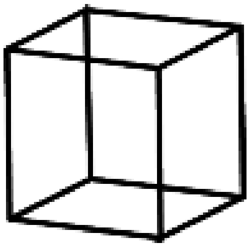

➢ Clock: Ask the subject to draw a clock face with numbers and the hands at ten past five. (For scoring see instruction guide: circle = 1, numbers = 2, hands = 2 if all correct).	**Visuospatial**<br>[Score 0-5]

Updated 20/11/2012

VISUOSPATIAL ABILITIES

- Ask the subject to count the dots without pointing to them

Visuospatial
[Score 0-4]

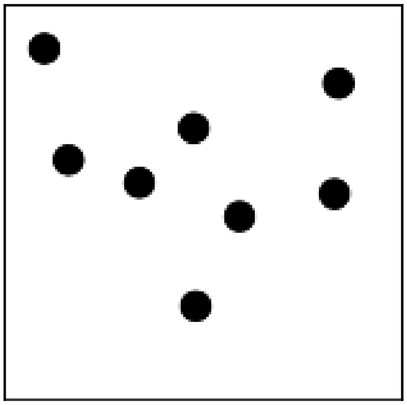

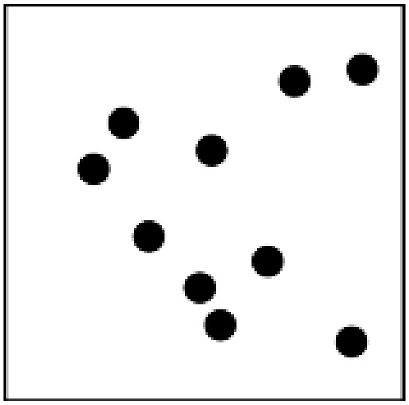

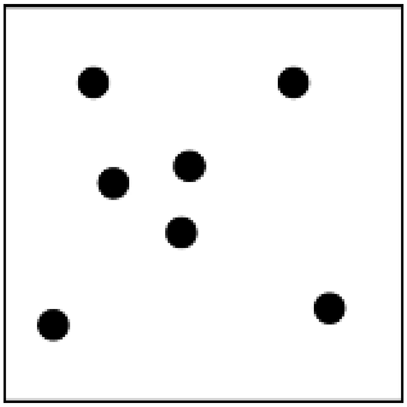

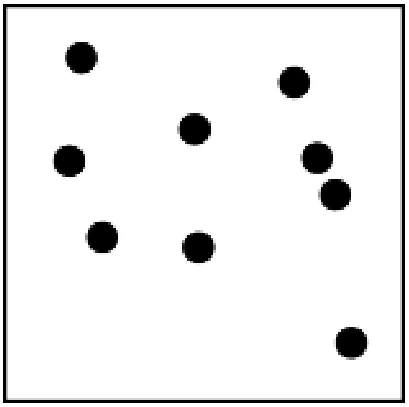

Updated 20/11/2012

VISUOSPATIAL ABILITIES

➢ Ask the subject to identify the letters	**Visuospatial** [Score 0-4] ☐

☐ 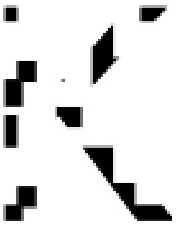 ☐ 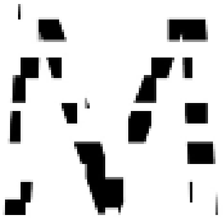

☐ 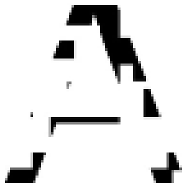 ☐ 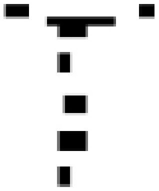

MEMORY

➢ Ask "Now tell me what you remember about that name and address we were repeating at the beginning"

Harry Barnes<br>73 Orchard Close<br>Kingsbridge<br>Devon	....<br>....... ....<br>..<br>..	**Memory** [Score 0-7] ☐

MEMORY

➢ This test should be done if the subject failed to recall one or more items above. If all items were recalled, skip the test and score 5. If only part was recalled start by ticking items recalled in the shadowed column on the right hand side; and then test not recalled items by telling the subject "ok, I'll give you some hints: was the name X, Y or Z?" and so on. Each recognised item scores one point, which is added to the point gained by recalling.	**Memory** [Score 0-5] ☐

Jerry Barnes		Harry Barnes		Harry Bradford		recalled	
37		73		76		recalled	
Orchard Place		Oak Close		Orchard Close		recalled	
Oakhampton		Kingsbridge		Darlington		recalled	
Devon		Dorset		Somerset		recalled	

SCORES

**TOTAL ACE-III SCORE**	/100
**Attention**	/18
**Memory**	/26
**Fluency**	/14
**Language**	/26
**Visuospatial**	/16

Updated 20/11/2012

24.6. Modified ADCS-CGIC

Interview Worksheet for CGIC Baseline Evaluation

The caregiver and subject must be interviewed separately by the clinician who will complete the CGIC. **Interview the caregiver first and then the subject.** The clinician must identify specific areas of deficits, behavior and abilities and record them on the worksheets. This information is to be referred to during subsequent visits at which the CGIC is performed to assist in assessing change occurring in the subject since baseline.

1. Thoroughly review the subject history to determine specific areas of deficits and abilities (*e.g.,* concentration, orientation, memory).
2. Interview the caregiver for a description of what a typical day is like with the subject. Note recent examples as well as specific deficits and abilities.
3. During the interview with the caregiver and subject, identify specific areas of subject behavior that might change and be assessed over time. However, because domains of change cannot be predicted with certainty, a broad overview of the subject's abilities, behavior and personality must be completed at baseline.
4. Ask the caregiver and subject about activities of daily living. Assess the subject's abilities to perform ADLs (*e.g.,* using the telephone, eating ability, ability to use transportation).
5. Ask the caregiver about personality and behavior issues. Using caregiver-supplied information, probe subject's insight into caregiver observations.
6. During the subject interview, note language abilities, affect and behavior.
7. Record any other domains with which you are concerned regarding subject change.

Follow-up Visit

1. Identical queries to baseline should be performed, by same clinician if possible.
2. The goal of the interview at follow-up is to determine whether there has been change since the baseline.
3. A modified CGIC is completed at the follow-up.

Acknowledgment: Derived from ADCS CGIC

Instructions:

The caregiver and subject must be thoroughly interviewed separately by the clinician who will complete the CGIC. **Interview the caregiver first and then the subject**. Refer to the CGIC Baseline Evaluation forms to assist in assessing the change that occurred in the subject.

**RELEVANT HISTORY**	
Background on cognitive disorder<br><br>Recent relevant events<br><br>Clinical illnesses	Caregiver:
	Subject:
**OBSERVATION/EVALUATION**	
Appearance	Caregiver:
	Subject:

Frontotemporal Dementia Multicenter Instrument & MR Study

Mayo Clinic, University of California San Francisco, University of California Los Angeles, University of Washington

MENTAL/COGNITIVE STATE	[structured exam if used:____________]
Attention/Concentration/Arousal/Alertness:<br>Mental focus<br>Engagement in tasks, conversations, events	Caregiver:
	Subject:
Orientation:<br>Time<br>Place<br>Knows way in familiar neighborhoods<br>Able to get to places outside of own neighborhood<br>Understanding of time relationships	Caregiver:
	Subject:
Memory<br>Registration<br>Long term recall/recall for past events<br>Short term recall from mental status (and {optional} a recent event supplied by caregiver)<br>From caregiver:<br>Recall of details of recent events<br>Recall of recent conversations<br>Ability to remember a short list such as for shopping?	Caregiver:
	Subject:

Frontotemporal Dementia Multicenter Instrument & MR Study
Mayo Clinic, University of California San Francisco, University of California Los Angeles, University of Washington

**MENTAL/COGNITIVE STATE continued**	
Language/speech<br>Fluency/expressive/receptive language<br>Comprehension<br>Understanding of word meaning<br>Paraphasia/word finding<br>Loquacious/taciturn<br>Repetition	Caregiver:
	Subject:
Praxis<br>Constructional ability	Caregiver:
	Subject:
Judgment/Problem Solving/Insight<br>Solving household problems<br>Dealing with small financial transactions<br>Dealing with complicated financial affairs<br>Ability to handle emergencies<br>Ability to understand situations<br>Insight into deficits, behavior	Caregiver:
	Subject:

Version date: 3/17/04 Page 4 of 8

**BEHAVIOR**	
Thought content, Hallucinations, Delusions<br>Thought organization<br>Thought appropriateness<br>Auditory/visual hallucinations<br>Misperceptions<br>Systematized/developed<br>Suspiciousness/paranoia<br>Fearfulness<br>Distractability	Caregiver:
	Subject:
Behavior/Mood/Personality<br>Affect/lability<br>Motivation/energy<br>Agitation/aggression<br>Appropriateness<br>Cooperativeness<br>Disinhibited?<br>Personality changes	Caregiver:
	Subject:
Sleep/Appetite<br>Sleep disorder/insomnia<br>Nocturnal activity<br>Hypersomnia, hyposomnia<br>Appetite/weight change	Caregiver:
	Subject:

**BEHAVIOR continued**	
Psychomotor activity<br>Wandering<br>Restlessness<br>Pacing<br>Posture<br>Gait<br>Stereotyped or compulsive actions	Caregiver:
	Subject:
Social behaviors<br>Participation in social interactions<br>Participation in community activities<br>Independent activities outside the home<br>Social appropriateness<br>Empathy towards others	Caregiver:
	Subject:

FUNCTIONING	
Complex Daily activities<br>finances<br>shopping<br>driving<br>household chores<br>hobbies<br>pastimes still engaged in	Caregiver:
	Subject:
Basic functions<br>dressing<br>hygiene/grooming<br>self-feeding<br>mobility<br>bowel/ bladder control	Caregiver:
	Subject:

CLINICIAN NOTES	
Caregiver interview: ______________________________	
Subject interview: ______________________________	

Instructions for making CGIC rating:

Based on your interviews with both the subject and caregiver, indicate your assessment of the overall change, whether or not in your judgment it is entirely due to drug therapy, of the subject's status since baseline.

IMPRESSION OF CHANGE DEFINITIONS:

1. **Marked Improvement** = A substantial improvement.
2. **Moderate Improvement =** A significant improvement.
3. **Minimal Improvement** = A noticeable improvement.
4. **No Change =** Symptoms remain essentially unchanged.
5. **Minimal Worsening** = A noticeable decline.
6. **Moderate Worsening =** A significant decline.
7. **Marked Worsening** = A severe decline.

Impression of Change: ________

24.7. Serotonin Toxicity (Syndrome) Diagnostic Interview and Rating Guide (In-Clinic)

Administration and Scoring

This guide is designed to enable the investigator to elicit clinical information that will allow diagnosis of serotonin toxicity (serotonin syndrome) by four published criteria and to quantify severity of symptoms.

The assessment has three sections: mental state, neurological and autonomic. These sections can be rated in any order and information gathered from the subject's general physical and neurological examination can be used to rate the items with the proviso that the stated conditions are met.

All items, apart from item 4, can be rated and scored in the clinic after the first dose of study medication. Item 4 can only be rated beginning on Day 2.

For each item a decision must be made as to whether the symptom or sign is absent (score 0) or present (score 1).

For items 7, 17, 18, 19 and 20 the physiological measurement is referenced to the physical and neurological examinations, except on Day 1 when they must also be recorded on the serotonin syndrome scoring sheet.

For items 1, 2, 5, 6, 10, 12, 15, 16 and 17 a further symptom severity rating (SSS score) must also be made.

Assessment scores must then be entered into the serotonin syndrome worksheet.

Interpretation

Subjects should be considered a possible case of serotonin toxicity if they meet the criteria for any of the four criteria following exposure to MT.

For possible cases the investigator is recommended to refer to the subject's Baseline score and consider whether any of the positive items are simply due to the dementia. For example, in a subject displaying moderate agitation at Baseline and moderate agitation 3 hours post introduction of MT, it is reasonable to conclude that the observed agitation is not due to serotonin toxicity. Diarrhea on the other hand may be a relatively common AE and should not form the basis for inferring possible serotonin toxicity.

Subjects still meeting criteria for serotonin syndrome after Baseline symptoms have been discounted should be considered probable serotonin syndrome cases.

For all probable cases of serotonin toxicity and for any subject developing pyrexia, possibly due to serotonin toxicity, the advice of a physician with expertise in toxicology must be immediately sought before the subject is allowed to leave the clinic.

A) MENTAL STATE

1) Abnormal Conscious Level (Delirium)

Rate this item positive if the subject on examination displays behavior suggestive of an abnormal conscious level (i.e., clouding, hypervigilance, hypovigilance) supported by an acute deterioration in at least one of the following: attention, thinking, constructional praxis or orientation on cognitive examination.

0 = Symptom absent
1 = Symptom present

SSS score
0 = no change in MMSE orientation
3 = at least 2 point deterioration in MMSE orientation

2) Agitation or Akathisia

Rate this item positive if the subject on examination displays excessive, non-goal directed motor behavior, for example pacing, foot tapping or restlessness, irrespective of whether it is considered to be agitation or akathisia.

0 = Symptom absent
1 = Symptom present

SSS score
0 = None
1 = Slight and intermittent
2 = Moderate (unrest while sitting)
3 = Severe and long lasting (sitting is nearly impossible, subject always feels restless)

3) Elevated Mood

Rate this item positive if any of the following are present on mental state examination: pressured speech, elation, mania or hypomania.

0 = Symptom absent
1 = Symptom present

4) Insomnia

Rate this item positive (beginning on Day 2) if the informant reports the subject to be having more difficulty falling or staying asleep since commencing the study drug.

Symptom not applicable = N/A (use only if informant not available)
0 = Symptom absent
1 = Symptom present

B) NEUROLOGICAL

5) Myoclonus

Rate this item positive if subject on examination displays sudden shock-like muscle jerks. These may occur spontaneously or in reaction to sensory stimulation or movement.

0 = Sign absent
1 = Sign present

SSS score
0 = No myoclonus
1 = Subject or informant reports short episodes of myoclonus
2 = Subject or informant reports repeated episodes; isolated myoclonic jerks are visible
3 = Persistent, visible myoclonic jerks

6) Tremor

Rate this item positive if subject on examination displays clinically relevant tremor at rest, on maintaining posture, or on intention.

0 = Sign absent
1 = Sign present

SSS score
0 = No tremor
1 = Tremor with small amplitude; functioning is not impaired
2 = Tremor with significant amplitude; functioning (*e.g.*, holding a cup) is moderately impaired
3 = Tremor with high amplitude; functioning is severely impaired

7) Mydriasis

Measure pupillary diameter in daylight.

Pupillary diameter = __________mm (OS)

(Where the left and right pupils differ in diameter, each should be recorded and appropriately labeled)

Pupillary diameter = __________mm (OD)

Rate this item positive if subject on examination has a pupillary diameter (in either one or both eyes) of 5 mm or greater.

0 = Enlarged pupil absent
1 = Enlarged pupil present

8) Nystagmus

Rate this item positive if subject displays nystagmus. This should also be elicited by getting the subject to fix on a finger moved rapidly to the midline.

0 = Nystagmus absent
1 = Nystagmus present

9) Clonus

Rate this item positive if there are more than 3 beats of clonus at any site. Clonus must be tested in both upper and lower limbs.

0 = Clonus absent
1 = Clonus present

10) Hyperreflexia

Rate this item positive if reflex is abnormally brisk or associated with clonus, scored as below. Reflexes must be tested in both upper and lower limbs.

0 = Sign absent
1 = Sign present

SSS score
0 = No hyperreflexia
1 = Hyperreflexia without clonus
2 = Hyperreflexia with non-sustained clonus
3= Hyperreflexia with sustained clonus

11) Hypertonia (Rigidity)

Rate this item positive if muscle tone is increased with features of rigidity. Tone must be tested in both the upper and lower limbs.

0 = Rigidity absent
1 = Rigidity present

12) Dizziness

Rate this item positive if the subject reports feeling dizzy or lightheaded. Similarly rate item positive if there is on examination evidence that subject is unsteady or has poor balance whilst standing or walking. When positive, scores are to be assigned as below.

0 = Symptom absent
1 = Symptom present

SSS score
0 = None
1 = Slight and intermittent feelings of dizziness or imbalance

2 = Subject feels dizzy or imbalance most of the time; functioning (moving, standing) is not impaired
3 = Subject always feels dizzy or imbalance; functioning (moving, standing) is affected

13) Incoordination

Rate this item positive if the subject on examination displays lack of control or inaccuracy in voluntary movement. Coordination should be tested in both upper (e.g., finger-nose test) and lower (e.g., heel-shin test) limbs.

0 = Incoordination absent
1 = Incoordination present

C) AUTONOMIC

14) Shivering

Rate this item positive if on examination there is involuntary shaking, trembling, quivering or teeth chattering as if the subject is cold.

0 = Shivering absent
1 = Shivering present

15) Diaphoresis (Sweating)

Rate this item positive if on examination the subject's skin feels moist or beads of perspiration can be seen. This must be rated at rest at normal environmental temperature and scored as below.

0 = Sign absent
1 = Sign present

SSS score
0 = No sweating
1 = Subjective feeling of increased sweating
2 = Moist skin; some beads of perspiration can be seen
3 = Visible beads of perspiration with wet clothes or bed sheet

16) Diarrhea

Rate this item by asking the subject about recent bowel habits.

0 = Symptom absent
1 = Symptom present

SSS score
0 = No diarrhea
1 = Feces with reduced consistency, but normal frequency
2 = Liquid feces and/or frequency 1-3/day

3 = Like 2 but frequency > 3 /day

17) Pyrexia (Fever)

Record oral temperature =__________ºC

0 = Sign absent
1 = Sign present

Rate this item positive if on examination the oral temperature is 38 ºC or above, scored as below.

SSS score
0 = <37 ºC
1= 37-37.9 ºC
2 = 38-38.9 ºC
3 = ≥ 39 ºC

18) Tachycardia

Measure resting heart rate after 5 minutes in a sitting position.

Resting heart rate =__________bpm

Rate this item positive if on examination (or ECG) the heart rate > 96 bpm.

0 = Absent
1 = Present

19) Tachypnea or Dyspnea

Measure resting respiratory rate *after 5 minutes in a sitting position.*

Resting respiratory rate = __________ bpm

Rate this item positive if either:

i) the subject reports dyspnea (difficulty breathing or shortness of breath) or
ii) there is tachypnea on examination (breathing rate of > 20 breaths / minute)

0 = Absent
1 = Positive

20) Hypertension or Hypotension

Measure blood pressure after 5 minutes in a sitting position.

Systolic blood pressure = __________ mmHg
Diastolic blood pressure =__________mmHg

Rate this item positive if on examination either:

i) systolic blood pressure > 160 mmHg, or diastolic blood pressure > 100 mmHg or
ii) systolic blood pressure < 100 mmHg or diastolic blood pressure < 50 mmHg

0 = Absent
1 = Positive

Serotonin Syndrome Worksheet

Item		Sternbach	Hunter	Hegerl	Radomski	
					Major	Minor
1	Abnormal Conscious Level (Delirium)	0 1		0 3	0 1	
2	Agitation or Akathisia	0 1	0 1	0 1 2 3		0 1
3	Elevated Mood				0 1	
4	Insomnia					0 1
5	Myoclonus	0 1	0 1	0 1 2 3	0 1	
6	Tremor	0 1	0 1	0 1 2 3	0 1	
7	Mydriasis					0 1
8	Nystagmus		0 1			
9	Clonus		0 1			
10	Hyperreflexia	0 1	0 1	0 1 2 3	0 1	
11	Hypertonia (Rigidity)		0 1		0 1	
12	Dizziness			0 1 2 3		
13	Incoordination	0 1				0 1
14	Shivering	0 1			0 1	
15	Diaphoresis (Sweating)	0 1	0 1	0 1 2 3	0 1	
16	Diarrhea	0 1		0 1 2 3		0 1
17	Pyrexia (Fever)	0 1	0 1	0 1 2 3	0 1	
18	Tachycardia					0 1
19	Tachypnea or Dyspnea					0 1
20	Hypertension or Hypotension					0 1
	**Total Score**					
	**Interpretation**	**Score of 3 or above = case**	**See Algorithm below**	**Score of 6 or above = case**	**Score of 4 major = case<br>Score of 3 major + 2 minor = case**	

Hunter Serotonin Toxicity Criteria - a case is diagnosed if any of the criteria a-e are met.

a) Myoclonus (item 5)
b) Tremor (item 6) and hyperreflexia (item 10)
c) Nystagmus (item 8) and either agitation (item 2) or diaphoresis (item 15)
d) Clonus (item 9) and either agitation (item 2) or diaphoresis (item15)
e) Hypertonia (item 11), temperature >38°C (item 17), and either nystagmus (item 8) or clonus (item 9)

24.8. Serotonin Toxicity Telephone Assessment

The assessment should be administered to the subject's caregiver *via* the telephone.

Has subject (S) had any new or worsening symptoms since starting ….?

Describe

____
____
____
____
____
____
____

1) Has S appeared more confused since starting …..?

2) Has S appeared more restless or agitated since starting…….?

3) Has S appeared excited or manic since starting …..?

4) Has S been having more difficulty falling or staying asleep since starting …?

5) Has S appeared more jumpy or jerky since starting ….?

6) Has S appeared more tremulous since starting ……?

7) Has S developed any unusual eye movements since starting…?

8) Has S become stiff or rigid since starting ……?

9) Has S complained more of feeling dizzy or lightheaded since starting ….?

10) Has S developed any problems with balance since starting …?

11) Has S become more clumsy or uncoordinated since starting …?

12) Has S started to shiver or tremble since starting ….?

13) Has S appeared or felt more sweaty since starting …..?

14) Has S developed diarrhea (frequent or loose motions) since starting ….?

15) Has S had a temperature above 37 °C since starting……….?

16) Has S complained of feeling breathless or appeared breathless since starting ….?

<u>Interpretation</u>

If a caregiver replies "yes" to any of the above, the subject should be immediately recalled to the clinic and examined for evidence of serotonin toxicity.

24.9. Summary of Changes

24.9.1. Protocol Version 1.1

The final protocol for Study TRx-237-007 (Version 1.0 dated 17 May 2012) has been revised (Version 1.1 dated 17 September 2012) to include non-substantial, administrative changes, as detailed in the table below.

None of these changes impact the study design, study conduct or safety of the subjects.

Summary of Changes	Affected Sections in Revised Protocol (Version 1.1)
Identification of the vendors for brain MRI-related activities; BioClinica, Inc. will be responsible for image acquisition and site interaction, and RadMD, LLC will be responsible for imaging oversight and providing the central blinded readers.	Section 1.2 Responsible Personnel
Pharmacovigilance and medical monitoring are to be performed by a different vendor (Worldwide Clinical Trials); contact details and reporting process for SAEs and pregnancy have been revised accordingly.	Section 1.2 Responsible Personnel<br>Section 8.1.4 Serious Adverse Events<br>Section 8.1.5 Pregnancy<br>Section 8.1.6.1 Investigator Reporting of SAEs and Pregnancy to Sponsor
Modifications have been made to the classifications for relationship of AEs to the study drug; “unrelated” has been changed to “not related”, and “probable” has been changed to “related”.	Section 8.1.2 Relationship to Study Drug
The requirements for Sponsor reporting of SUSARs have been clarified; SUSARs will be reported to the pertinent regulatory authorities and to the DSMB in parallel.	Section 8.1.6.2 Sponsor Reporting of SUSARs to Regulatory Authorities<br>Section 8.1.6.2.2 Fatal or Life-threatening SUSARs<br>Section 8.1.6.2.3 Other SUSARs
A justification for the upper age restriction has been added.	Section 2.4 Rationale for Study

24.9.2. Protocol Version 2.0

The final protocol for Study TRx-237-007 (Version 1.1 dated 17 September 2012) has been revised (Version 2.0 dated 20 September 2012). The majority of the revisions are non-substantial and administrative and/or editorial in nature.

Procedures for which visit windows are now specified or clarified:

- Telephone contacts for evaluation of serotonin toxicity 48 and 72 hours after the first dose (± 4 hours) and between visits (± 14 days)
- MRI (± 14 days)

Revisions that are changes to inclusion/exclusion criteria, dosing and drug supplies, laboratory testing, and other procedures are summarized in the table below. Changes that are substantive clarifications in the event of discrepancies are also included. None of these changes impact the study design or safety of the subjects.

Summary of Changes	Affected Section(s) in Revised Protocol (Version 2.0)
Changes to Inclusion and Exclusion Criteria	
FSH levels will no longer be measured to confirm post-menopausal status.	Synopsis<br>Section 4.4 Schedule of Assessments<br>Section 5.1 Inclusion Criterion No. 6<br>Section 8.3.4 Other Laboratory Tests<br>Section 24.3 Assessments by Visit
Examples of acceptable methods of contraception are provided.	Synopsis<br>Section 5.1 Inclusion Criterion No. 6<br>Section 4.7.5 Contraceptive Measures
The protocol has been modified to cover all national laws regarding informed consent in individuals with reduced decision-making capacity.	Synopsis<br>Section 5.1 Inclusion Criteria<br>Section 14 Informed Consent<br>Section 24.3 Assessments by Visit
Subjects with a history of atrial fibrillation or evidence on ECG are now explicitly excluded; there is no longer a need to exclude subjects on Coumadin derivatives (originally exclusionary as a proxy for atrial fibrillation).	Synopsis<br>Section 5.2 Exclusion Criteria Nos. 17, 22
Subjects with orthostatic hypotension are no longer excluded (MT has not been found to cause orthostasis).	Synopsis<br>Section 5.2 Exclusion Criterion No. 17
Subjects with hypersensitivity to organic dyes similar to methylene blue are to be excluded.	Synopsis<br>Section 5.2 Exclusion Criterion No. 21
Exclusion criteria have been expanded with additional examples of significant CNS disorders (Lewy body dementia), intracranial pathology (large white matter hyperintense lesions), clinically significant hepatic disease (presence of encephalopathy or ascites), and acceptable continuous care facilities.	Synopsis<br>Section 5.2 Exclusion Criteria Nos. 1, 2, 12, 19
Subjects are now allowed to enter study using chlorpromazine, thioridazine, or ziprasidone but not olanzapine (previously allowed).	Synopsis<br>Section 5.2 Exclusion Criterion No. 22<br>Section 4.7.3 Drugs Used to Manage Behavioral Disturbance
Use of primidone has been added as an exclusion.	Synopsis<br>Section 5.2 Exclusion Criterion No. 22

Dosing and Drug Supplies	
It is clarified that the maximum duration of interruption of study drug is to be 30 days.	Synopsis<br>Section 4.1 General Description<br>Section 4.6 Study Treatment
Clarified that study drug is to be permanently discontinued in response to ARIA.	Section 6.2.2.3 ARIA<br>Section 9.1.2 Brain MRI acquisition
Drug is packaged in 8-week supplies and therefore drug will not be dispensed at Visit 3 or supplies collected at Visit 4.	Synopsis<br>Section 4.4 Schedule of Assessments<br>Section 6.4 Dispensing<br>Section 24.3 Assessments by Visit
Statements that study drug should not be frozen, refrigerated, and protected from direct heat have been removed; packing is suitably protective, as supported by stability data.	Section 6.3 Packaging, Labeling, and Storage<br>Section 6.4 Dispensing
Changes to Laboratory Testing	
Direct (conjugated) bilirubin has been added to the serum chemistry panel with indirect bilirubin calculated as the difference from total.	Synopsis<br>Section 4.4 Schedule of Assessments<br>Section 8.3.1 Serum Chemistry<br>Section 24.3 Assessments by Visit
Heinz bodies will no longer be evaluated routinely as the test is not specific to the primary hematologic toxicity (methemoglobinemia). If an Investigator has concerns, a blood sample will be sent to a local lab for measurement of Heinz bodies. Procedures for dose interruption, reduction, and discontinuation based on measurement of methemoglobin and Heinz bodies have been clarified.	Section 6.2.2.1 Methemoglobinemia and/or Hemolytic Anemia<br>Section 8.3.2 Hematology
Folate (previously only measured at Screening) will now also be measured at Baseline (pre-dose), and every subsequent visit thereafter (or upon early termination), including the 4-week post-treatment follow-up visit if applicable (Visit 10).	Synopsis<br>Section 4.4 Schedule of Assessments<br>Section 8 Assessment of Safety<br>Section 8.3 Clinical Laboratory Tests<br>Section 8.3.4 Other Laboratory Tests<br>Section 24.3 Assessments by Visit
TSH (previously only measured at Screening) will now also be measured after 24 and 52 weeks of treatment, with a thyroid hormone panel (T_3 and T_4) obtained in the event of abnormality; further follow-up is to be performed as needed.	
Oxygen content will be measured by pulse co-oximetry in addition to methemoglobin.	Synopsis<br>Section 4.4 Schedule of Assessments<br>Section 8 Assessment of Safety<br>Section 8.4 Pulse Co-oximetry<br>Section 24.3 Assessments by Visit
Other Procedural Changes	
If a caregiver of a subject withdraws his or her consent, the subject must then also be withdrawn if alternative arrangements are not available (*e.g.*, alternate caregiver).	Section 5.3 Discontinuation/Withdrawal<br>Section 14 Informed Consent
Baseline efficacy assessments can be made over 2 days for subject/caregiver convenience (prior to the first dose).	Section 4.4 Schedule of Assessments
The Modified ADCS-CGIC is to be rated in the caregiver first (rather than the subject); the reference point for assessment of change (Baseline visit) has been clarified.	Section 7.1 Instruments<br>Section 7.1.2 Modified Alzheimer's Disease Cooperative Group – Clinical Global Impression of Change (Modified ADCS-CGIC)
Consistent with ICH GCP 4.3, an investigator no longer needs to contact the medical monitor in order to unblind the treatment assignment of a specific subject if needed for managing a medical emergency.	Section 4.5 Randomization and Blinding

It has been made more clear that the same in-clinic and follow-up telephone monitoring procedures are to be followed for subjects starting a drug with serotonergic potential during study.	Section 4.7.2 Drugs with Serotonergic Potential<br>Section 24.3 Assessments by Visit
Only the caregiver (not the subject) is to be contacted by telephone.	Synopsis<br>Section 4.1 General Design<br>Section 4.4 Schedule of Assessments<br>Section 24.3 Assessments by Visit
All ECG recordings (including Screening) will be 10 seconds in duration. Further details and clarification have been provided for holding the dose in response to abnormalities, recording, and analysis of machine-read ECG assessments provided by the central ECG reader.	Section 4.4 Schedule of Assessments<br>Section 6.2.2.4 QT Interval<br>Section 10.3.5.5 Electrocardiogram<br>Section 24.3 Assessments by Visit
The body systems to be included in the neurological examination have been updated.	Section 8.7 Physical and Neurological Examinations
The volume of blood samples for MT drug assay has been increased from 2 mL to 6 mL to allow for analysis of whole blood concentrations in addition to plasma; collection procedures have been modified accordingly and acceptable tubes and labeling updated.	Section 9.2.1 Procedure for Blood Sample Collection<br>Section 9.2.2 Packaging, Labeling, and Shipping of Blood Samples
Miscellaneous	
Public registries where general trial data may be posted have been updated.	Section 14. Informed Consent
An overview of El Escorial research criteria for ALS has been added.	Section 24.2 El Escorial Research Criteria for Diagnosis of Amyotropic Lateral Sclerosis (Brooks *et al.*, 2000)
Editorial changes and clarifications have been made to the serotonin toxicity (syndrome) diagnostic interview and rating guide.	Section 24.6 Serotonin Toxicity (Syndrome) Diagnostic Interview and Rating Guide (In-Clinic)
A more current version of the interview worksheet for the Modified ADCS-CGIC evaluation replaces the previous version.	Section 24.5 Modified ADCS-CGIC

24.9.3. Protocol Version 3.0

The final protocol for Study TRx-237-007 (Version 2.0 dated 20 September 2012) has been revised (Version 3.0 dated 12 December 2012). The majority of the revisions are non-substantial and administrative and/or editorial in nature.

Revisions that are changes to inclusion/exclusion criteria, safety assessments, and other procedures are summarized in the table below. Additional revisions are intended to correct typographical errors, eliminate inconsistencies or add further clarification.

Summary of Changes	Affected Section(s) in Revised Protocol (Version 3.0)
Background	
The expression of the MTC dose has been edited to be in terms of MT rather than the salt, for consistency.	Section 2.2 Nonclinical Data
The discussion of nonclinical data has been updated to include new reproductive toxicity findings; the discussion of contraceptive measures has been updated accordingly.	Section 2.2 Nonclinical Data<br>Section 4.7.6 Contraceptive Measures
Updates have been added to the discussion of clinical pharmacokinetic and safety data.	Section 2.3.1 Pharmacokinetics<br>Section 2.3.3 Safety
Inclusion and Exclusion Criteria	
Clarification that subjects must be less than 70 years of age at Screening to be eligible for enrollment in this study.	Synopsis / Inclusion Criteria<br>Section 2.4 Rationale for Study<br>Section 5.1 Inclusion Criterion No. 4
For exclusion of subjects meeting DSM IV-TR criteria for substance (including alcohol) related disorders, the specified time period has been reduced from within the past 5 years to within the past 2 years (as bvFTD may itself be associated with some degree of alcohol abuse or dependence).	Synopsis / Exclusion Criteria<br>Section 5.1 Exclusion Criterion No. 10
The normal ranges for folate and Vitamin B_{12} established by the manufacturer of the test kits used by the central laboratory are now specified; subjects who are below the normal range at Screening (confirmed upon repeat) are to be excluded. Guidance is provided for interpreting values and a brief description of the central laboratory's analytical method is included.	Synopsis / Exclusion Criteria<br>Section 4.7.7 Folate and Vitamin B_{12}<br>Section 5.2 Exclusion Criterion No. 16<br>Section 8.3.4 Other Laboratory Tests
Reference to Baseline has been removed from the exclusion criteria related to QTcB, blood pressure, and heart rate measurements.	Synopsis / Exclusion Criteria<br>Section 5.2 Exclusion Criterion No. 18
Treatment currently or within 3 months before Baseline with moderate to strong inhibitors of CYP1A2 is no longer included in the criteria for subject exclusion.	Synopsis / Exclusion Criteria<br>Section 5.2 Exclusion Criterion No. 23<br>Section 4.7.2 Drugs with Serotonergic Potential<br>Section 4.7.5 Other Medications
Clarification that the exclusion criteria related to participation in a clinical trial applies to both current and prior participation; clinical trials of medical foods are now included in the criteria.	Synopsis / Exclusion Criteria<br>Section 5.2 Exclusion Criterion No. 24
Safety Assessments / Procedures	
Clarification has been added regarding dosing and study continuation decisions to be made based on ECGs on Day 1.<br><br>Dosing may be held based on local interpretation (using the average of the three readings for QTcB and heart rate) and subsequent eligibility decisions made pending receipt of centrally read results in the event of deviations from Screening ECG detected at Baseline and considered clinically significant. A decision of whether to discontinue the subject or re-schedule the Baseline visit should be	Synopsis / Methodology<br>Section 4.4 Schedule of Assessments<br>Section 6.2.2.4 QT Interval and ECG Abnormalities<br>Section 8.6 Electrocardiography<br>Section 10.3.5.5 Electrocardiogram<br>Section 24.3 Assessments by Visit

Summary of Changes	Affected Section(s) in Revised Protocol (Version 3.0)
made on the basis of the central read; a cardiology consult should be sought if appropriate.<br><br>Subsequent decisions with respect to the ECG may be made on the basis of the central reading, with cardiology consult if appropriate. In addition to the central reading results, local machine-read results from Screening and Baseline are to be recorded in the eCRF and included in a separate listing.	
The C-SSRS will now be applied at Screening (Visit 1), and will not be applied pre-dose at Baseline (Visit 2).	Synopsis / Safety and Tolerability<br>Section 4.4 Schedule of Assessments<br>Section 8.9 Columbia-Suicide Severity Rating Scale (C-SSRS)<br>Section 24.3 Assessments by Visit
Clarification has been added that if dosing is held for more than 30 days in response to methemoglobinemia, the subject should be discontinued.	Section 6.2.2.1 Methemoglobinemia and/or Hemolytic Anemia
Clarification that the UPDRS version used in this study will be the revised version developed based on the critique by the Movement Disorder Society Task Force for Rating Scales for Parkinson's Disease published in July 2008; the description of the scale has been updated accordingly.	Section 8.8 Unified Parkinson's Disease Rating Scale (UPDRS Parts II and III)<br>(previously Section 7.1.5)
MCH, MCHC, and RBC morphology are now specified as analytes to be included in the hematology panel.	Section 8.3.2 Hematology
Clarification that Heinz body determination is not mandated.	Section 8.3.2 Hematology
The reporting and handling requirements for AESIs have been clarified; possible cases of serotonin toxicity and ARIA will be reported to the Sponsor and the procedure will be as for SAEs.	Section 8.1.3 Adverse Events of Special Interest<br>Section 8.1.4 Serious Adverse Events
The measurement of pulse rate has been increased from 30 seconds to 60 seconds.	Section 8.5.2 Blood Pressure and Pulse
The protocol now indicates that the serotonin toxicity assessment requires a qualified medical assessor.	Section 4.4 Schedule of Assessments<br>Section 6.2.2.2 Serotonin Syndrome<br>Section 8.8 Serotonin Syndrome
Other Assessments / Procedures	
Corrected scheduled study days listed for study weeks in treatment period and follow-up visit.	Section 4.1General Description<br>Section 4.4 Schedule of Assessments
Subject identification numbering has been clarified; subjects will be assigned a unique number (007-CC-SSS-EE) with the first three digits for the study (007), the next two digits for the country, the next three digits for the site, and the last two digits for the sequential order of enrollment at a given site.<br><br>Subjects who are re-screened will receive a new identification number; the previous number is also to be recorded by the site. Initial screening data and rescreening data will be captured in the database for subjects who are re-screened.	Section 4.5 Randomization and Blinding<br>Section 5.2 Exclusion Criteria
With respect to concomitant medications, "medication" is now clarified to encompass prescription and over-the-counter drugs or biologics, vitamins used in supra-pharmacologic doses, alternative pharmacotherapies for dementia, medical foods, and for women, forms of contraception. Initiation of folate therapy during participation in the study is no longer specified as to be avoided.	Section 4.7 Concomitant Medication
For subjects who have been receiving a medical food or a stable dose of alternative pharmacotherapy for dementia, it is preferable	Section 4.7.1 Dementia Medication

Summary of Changes	Affected Section(s) in Revised Protocol (Version 3.0)
that the dose of such therapy has remained stable for ≥ 6 months before randomization; examples of medical foods have been added.	
Based on preliminary results from a recent drug-drug interaction study which suggest that MT may inhibit cytochrome P450 3A4 (CYP3A4), additional guidance is provided regarding the monitoring of subjects on drugs known to be metabolized by this enzyme system; examples of these drugs are provided.	Section 4.7.1 Dementia Medication<br>Section 4.7.3 CYP3A4 Substrates (Section added)
For brain MRI scans, 1.5-Tesla and 3.0-Tesla machines will be used.	Section 9.1.2 Brain MRI
New contact provided for sending blood samples intended for analysis of MT concentrations.	Section 9.2.3 Analytical Laboratory
Where local laws require it, national regulatory requirements with regard to the inclusion of subjects who are unable to consent will be followed by the investigators. In particular, in Germany, the risk threshold and degree of burden/distress will be monitored constantly by the investigators in accordance with §41 (3) of the German Drug Law (AMG). Additional detail is now provided regarding the evaluation of the risk threshold by the Sponsor and the appointed DSMB, as well as the monitoring of the degree of burden/distress by the participating investigators.	Section 15 Investigator Responsibilities
Publication procedures have been clarified.	Section 19 Publication
Reference to Clinical Dementia Rating (CDR) has been removed from the worksheet for the Modified ADCS-CGIC evaluation, as CDR is not performed in this study.	Section 24.5 Modified ADCS-CGIC

24.9.4. Protocol Version 4.0

The final protocol for Study TRx-237-007 (Version 3.0 dated 12 December 2012) has been revised (Version 4.0 dated 21 August 2013). Revisions that are modifications or clarifications to the overall protocol / background information; inclusion / exclusion criteria; study drug administration; efficacy, safety, and other assessments / procedures; statistical analysis; and administrative procedures are summarized in the table below.

Other revisions are editorial and/or non-substantial and are intended to add further clarification, eliminate inconsistencies, or correct typographical errors.

Summary of Changes	Affected Sections in Revised Protocol (Version 4.0)
Title Page	
The Title Page has been revised to list the Scotland address (for the convenience of communications) in addition to the Singapore address (the Sponsor's registered address).	Title page
Overall / Background	
A new contact is listed under the responsible personnel for pharmacovigilance. Address and contact details have been added for the Sponsor Global Project Lead, Sponsor Head of Safety and Medical Monitoring, and Central Laboratory locations (in Singapore and the United States). Addresses and contact details have been updated for the personnel responsible for imaging oversight and central blinded readers (RadMD, LLC) and ECGs (BioClinica, Inc.). Contact numbers have been added for the University of Aberdeen GLP Test Facility.	Section 1.2 Responsible Personnel
Study sites will now be included in Argentina; text referring to North America has been changed to "the Americas" accordingly to encompass this new country. Study sites are also planned to be included in Bulgaria, Finland, Italy, and Spain.	Section 1.2 Responsible Personnel<br>Section 4.2 Population<br>Section 10.3.4.1 Primary Efficacy Analyses
Clarified that subjects who complete the treatment period may (rather than will) be offered an opportunity to subsequently receive treatment with LMTM in a separate open-label extension study, as this may be dependent upon other factors such as tolerability and response.	Synopsis<br>Section 4.1 General Description<br>Section 4.3 Duration<br>Section 24.3 Assessments by Visit
The number of study sites to be included in the study has increased.	Synopsis<br>Section 4.2 Population
Updated background information for clinical pharmacokinetics of MT.	Section 2.3.1 Pharmacokinetics
Objectives	
An exploratory objective/endpoint has been added to evaluate the effect of LMTM as assessed by the change from Baseline on ACE-III to permit comparison of ACE-R and ACE-III total scores.	Synopsis<br>Section 3.3 Exploratory<br>Section 4.4 Schedule of Assessments<br>Section 7.2 Instruments<br>Section 7.2.1 Addenbrooke's Cognitive Examination<br>Section 10.1.3 Exploratory Endpoints<br>Section 10.3.4.5 Exploratory Efficacy Analyses<br>Section 24.3 Assessments by Visit<br>Section 24.5 Addenbrooke's Cognitive Examination - III (ACE-III) *(Section added)*

An exploratory objective has been added to determine the effect of LMTM in subjects with known genetic mutations associated with bvFTD.	Synopsis<br>Section 3.3 Exploratory<br>Section 4.4 Schedule of Assessments<br>Section 9.3 Genotyping<br>Section 10.3.4.5 Exploratory Efficacy Analyses<br>Section 10.3.7 Genotyping<br>Section 24.3 Assessments by Visit
Inclusion / Exclusion Criteria	
The age limit has been increased to <80 years at screening.	Synopsis<br>Section 2.4 Rationale for Study<br>Section 5.1 Inclusion Criterion No. 4
Added clarification to inclusion criterion to specify adequate contraception for female subjects in Italy (*i.e.*, accept to avoid a pregnancy for at least 3 months prior to Baseline and throughout participation in the study). Clarified that female subjects are required to be competent to use adequate contraception and to agree to maintain adequate contraception throughout participation in the study.	Synopsis<br>Section 4.7.7 Contraceptive Measures<br>Section 5.1 Inclusion Criterion No. 6
Added clarification to inclusion criterion to specify adequate informed consent for subjects in Germany (*i.e.*, subjects must be able to provide their own written informed consent).	Synopsis<br>Section 5.1 Inclusion Criterion No. 7<br>Section 14 Informed Consent
Caregiver inclusion criterion has been revised such that subjects may have <u>one or more</u> identified caregivers who meet certain criteria, including living with or seeing subjects for certain hours/days of week <u>or</u> the investigator deems the extent of contact sufficient to detect meaningful change.	Synopsis<br>Section 5.1 Inclusion Criterion No. 8
Additional examples of significant CNS disorders other than bvFTD have been included.	Synopsis<br>Section 5.2 Exclusion Criterion No. 1
Added clarification to exclusion criterion to specify that residence in low grade assisted living facility is allowed so long as it is not mandated by an order issued either by the judicial or the administrative authorities.	Synopsis<br>Section 5.2 Exclusion Criterion No. 12
Study drug may not be dissolved in fluids prior to ingestion and the exclusion criterion has been updated accordingly.	Synopsis<br>Section 5.2 Exclusion Criterion No. 13
Exclusion criterion has been modified to no longer allow subjects with Screening hemoglobin (confirmed upon repeat) below age/sex appropriate lower limit of the central laboratory normal range to be treated and rescreened to correct the deficit.	Synopsis<br>Section 5.2 Exclusion Criterion No. 16
Exclusion criterion has been modified for folate and Vitamin B_{12} and supplementation guidelines provided if values are below < 4.0 ng/mL and/or < 150 pg/mL, respectively prior to initiating drug. If appropriate Vitamin B_{12} levels can be achieved within the 42-day screening window, the subject may be entered into the study; otherwise the subject must be reconsented and rescreened after the deficit has been corrected. Additional guidance is provided if these levels become deficient during the study.	Synopsis<br>Section 4.7.8 Folate and Vitamin B_{12}<br>Section 5.2 Exclusion Criterion No. 16<br>Section 8.3.5 Review of Laboratory Results
Exclusion criterion has been modified for abnormal serum chemistry to exclude subjects with creatinine clearance <30 mL/min at Screening; subjects whose creatinine clearance reaches <30 mL/min on-study are to be discontinued.	Synopsis<br>Section 5.2 Exclusion Criterion No. 17<br>Section 6.2.2.5 Other Safety Reasons Requiring Discontinuation

Exclusion criterion has been modified to clarify that evidence of atrial fibrillation on Screening ECG or history of atrial fibrillation is exclusionary only if not currently controlled or where QT interval cannot in the opinion of the investigator be assessed by the triplicate ECGs. It is clarified that this determination may be made on the basis of either the local interpretation or the central read. If better control of the heart rate can be achieved after adequate treatment, the subject may be entered into the study if still within the 42-day window, or else the subject must be reconsented and rescreened. Additional guidance is provided regarding a cardiology consult. QTcF is to be used and based on the mean of three triplicate ECGs. The exclusionary limit for males has been raised to >460 msec.	Synopsis<br>Section 4.4 Schedule of Assessments<br>Section 5.2 Exclusion Criterion No. 18<br>Section 6.2.2.4 QT Interval and ECG Abnormalities<br>Section 8.6 Electrocardiography<br>Section 10.3.5.5 Electrocardiogram<br>Section 24.3 Assessments by Visit
Subjects with hepatitis are to be excluded from the study. Exclusion criterion has been expanded to include additional examples of concurrent acute or chronic clinically significant immunologic diseases other than bvFTD.	Synopsis<br>Section 5.2 Exclusion Criterion No. 20
Treatment currently or within 3 months before Baseline with anxiolytics and/or sedatives/hypnotics is no longer included as exclusion criteria. Guidance has been clarified regarding subjects' use of these types of medications during participation in the study. Amphetamine and dexamphetamine have been added as excluded medications.	Synopsis<br>Section 4.7.4 Drugs Used to Manage Behavioral Disturbance<br>Section 4.7.5 Other Medications<br>Section 5.2 Exclusion Criterion No. 23
Exclusion criterion regarding current/prior participation in a clinical trial of a product for cognition within the 3 months prior to Screening (unless randomized to placebo) now includes all clinical trials rather than only Phase 3 trials.	Synopsis<br>Section 5.2 Exclusion Criterion No. 24
Study Drug Administration	
Instructions for dosing interruption have been revised to specify that interruption of dosing may be allowed for up to (maximum duration on any one occasion) 30 days during the first 6 months of treatment and/or 14 days during the second 6 months of treatment.	Synopsis<br>Section 4.1 General Description<br>Section 4.6 Study Treatment<br>Section 6.2.2 Dose Interruption/ Reduction/ Discontinuation<br>Section 6.2.2.1 Methemoglobinemia and/or Hemolytic Anemia
Study drug should not be dissolved in fluids prior to ingestion. Subjects should not be enrolled if there are swallowing difficulties which prevent taking the medication as instructed and subjects/caregivers should be warned regarding discoloration of teeth and oral mucosa if the product is not swallowed immediately.	Synopsis<br>Section 5.2 Exclusion Criterion No. 13<br>Section 6.2.1 Double-Blind Study Drug (LMTM or Placebo)<br>Section 6.4 Dispensing
Subjects should be entered into the study only after careful discussion of the possibility that discoloration in the context of incontinence may prove unacceptable to the subject/caregiver.	Section 6.1.1 Study Drug
Efficacy Assessments / Procedures	
Clarified that the UPDRS will provide information pertinent to both efficacy and safety; moved discussion of UPDRS-related assessments and analyses to safety sections as this scale is now listed under safety assessments in Section 8.	Section 4.4 Schedule of Assessments<br>Section 7.2 Instruments<br>Section 10.3.5.7 UPDRS (Section added)
Clarified the roles and qualifications of efficacy raters or assessors.	Section 7.1 Raters<br>Section 7.2.2 Modified Alzheimer's Disease Cooperative Study - Clinical Global Impression of Change (Modified ADCS-CGIC)

Safety Assessments / Procedures	
Background information regarding safety has been updated to provide more detail regarding the potential risk of MAO inhibition and the theoretical risk of increased serotonin levels with oral LMTM (or its concomitant use with serotonergic drugs). Added potential risk caused by ingestion of foods containing high amounts of tyramine, symptoms of a hypertensive crisis and examples of tyramine-rich foods and beverages to be avoided. Tryptophan or its metabolites need not be avoided despite serotonergic potential.	Section 2.3.3 Safety<br>Section 2.4 Rationale for Study<br>Section 4.7.2 Drugs with Serotonergic Potential<br>Section 4.7.6 Dietary Tyramine
MRI scans of the brain obtained at Screening/Baseline are no longer required to be contrast-enhanced.	Section 4.4 Schedule of Assessments<br>Section 9.1.2 Brain MRI Acquisition
The telephone contacts with caregivers of subjects receiving serotonergic medication are now specified to occur with a minimum of 1 hour between contacts. Subjects are to remain in close proximity to a local hospital and in telephone contact with the clinic for at least 12 to 14 hours post-dose.	Synopsis / Methodology<br>Section 2.4 Rationale for Study<br>Section 4.1 General Description<br>Section 4.4 Schedule of Assessments<br>Section 6.2.2.2 Serotonin Syndrome<br>Section 8.5.1 Temperature and Respiratory Rate<br>Section 8.9 Serotonin Toxicity (Syndrome)<br>Section 24.3 Assessments by Visit
Clarified the criteria for subject discontinuation based on ARIA findings and added summary of the appropriate imaging follow-up to be performed in response to ARIA findings (to be consistent with the Imaging Charter).	Synopsis<br>Section 4.4 Schedule of Assessments<br>Section 6.2.2.3 ARIA<br>Section 8.1.3 Adverse Events of Special Interest<br>Section 9.1.5 Site Review
ECGs will be obtained in triplicate at the Screening visit (within a 2- to 5-minute interval with the subject in a supine position, suitably rested for at least 5 minutes) and at Visit 2; subsequently (at all other visits or upon early termination), single recordings will be made, unless there is an emergent abnormality which is of clinical significance in the judgment of the investigator, in which case triplicate ECG recordings should be made. Eligibility, dosing, and monitoring guidance have been provided for subjects with atrial fibrillation and in subjects with intraventricular conduction blocks, as appropriate.	Synopsis<br>Section 4.4 Schedule of Assessments<br>Section 6.2.2.4 QT Interval and ECG Abnormalities<br>Section 8.6 Electrocardiography<br>Section 24.3 Assessments by Visit
Methemoglobin and oxygen saturation will be measured within 1 hour before administration of the first dose of study drug, rather than approximately 1 hour prior to dosing.	Synopsis<br>Section 4.4 Schedule of Assessments<br>Section 24.3 Assessments by Visit
Clarified the timing of clinical laboratory tests in the schedule of assessments and added footnote to indicate that an unscheduled visit is to take place if needed in response to a safety concern.	Section 4.4 Schedule of Assessments
Additional examples of alternative pharmacotherapy for dementia that may be continued during participation in the study are included.	Section 4.7.1 Dementia Medication<br>Section 10.3.2 Baseline Characteristics and Concomitant Medications<br>Section 10.3.4.4 Subgroup Analyses
Updated guidance regarding monitoring of concomitant medications is provided based on preliminary results from a recently completed drug-drug interaction study which indicate that MT is a weak inhibitor of CYP3A4, CYP2C8, and CYP2C19, as well as a weak inducer of CYP2B6 and the P-gp transporter. The protocol now refers to the Investigator's Brochure for examples of drugs metabolized	Section 4.7.3 CYP and P-gp Substrates<br>Section 10.3.2 Baseline Characteristics and Concomitant Medications

by these enzymes or substrates of P-gp.	
Added clarification that any subject newly starting a serotonergic drug during the study should be subjected to the same monitoring procedures	Section 6.2.2.2 Serotonin Syndrome
Clarified procedures for repeat testing and treatment discontinuation in response to treatment-emergent QT interval and ECG abnormalities. Guidelines for discontinuing treatment due to ECG abnormalities have been revised: QTc interval prolongation text has been removed and QTc interval values have been modified to >500 msec in either sex.	Section 6.2.2.4 QT Interval and ECG Abnormalities
Included estimated total blood volume to be collected for each subject over the course of the study.	Section 8 Assessment of Safety
Clarified that asymptomatic subjects with≤ 4 new microhemorrhages need not be handled as an SAE.	Section 8.1.3 Adverse Events of Special Interest
SAE definition now indicates that deaths include suicides, and other medically significant events have been clarified.	Section 8.1.4 Serious Adverse Events
Given that all subjects will receive some amount of LMTM, treatment allocation will not be unblinded for this study; guidance for Sponsor reporting of SUSARs to regulatory authorities has been revised accordingly.	Section 8.1.6.2 Sponsor Reporting of SUSARs to Regulatory Authorities<br>Section 8.1.6.2.1 Unblinding Treatment Allocation
Updated the estimated blood volume to be collected for each hematology panel (decreased from 3.0 mL to 2.0 mL).	Section 8.3.2 Hematology
Because MT potentially interferes with colorimetric measurements, the protocol has been updated to require extra samples. Information regarding storage, analysis, and discarding of samples have been added..	Section 8.3.3 Urinalysis
For G6PD deficiency screening, use of a local laboratory is permitted.	Section 8.3.4 Other Laboratory Tests
Added guidance regarding use of pulse co-oximeter for measurement of methemoglobin and oxygen saturation, *i.e.*, it should be left on only so long as needed to obtain the recording(s).	Section 8.4 Pulse Co-Oximetry
In the Serotonin Toxicity (Syndrome) Diagnostic Interview and Rating Guide, the rating for mydriasis has been updated to indicate that where the left and right pupils differ in diameter, each should be recorded and appropriately labeled.	Section 24.7 Serotonin Toxicity (Syndrome) Diagnostic Interview and Rating Guide (In-Clinic)
Statistical Analyses	
An additional sensitivity analysis has been added: repeated measures analysis of the ADCS-CGIC using the Generalized Estimating Equation (GEE) model.	Section 10.3.4.2.3 Responder Analyses
A responder has been defined as anyone with no change (score of 4) or improvement (scores between 1 and 3).	Section 10.3.4.2.3 Responder Analyses
For statistical summaries of ARIA data, the number of microhemorrhages will be tabulated by treatment group.	Section 10.3.5.2 ARIA
Estimated glomerular filtration rate (eGFR) will be calculated based on the Modification of Diet in Renal Disease (MDRD) Study, to be further described in the SAP.	Section 10.3.5.3 Laboratory Tests of Blood and Urine
Any local interpretation of ECG used to make dosing or patient management decisions is now specified as to be included in the eCRF. Parameters to be included for statistical analyses now also include RR and uncorrected QT intervals.	Section 10.3.5.5 Electrocardiogram

Interim analyses may assess projected dropout rate and assumed SD for the change from Baseline to Week 52 in ACE-R.	Section 10.4 Interim Analysis
Other Procedures / Miscellaneous	
In discussion of reasons for discontinuation of study drug, it is now clarified that "lack of efficacy" includes worsening of cognitive capacity, and that in Germany this includes loss of the ability to give consent.	Section 5.3 Discontinuation/Withdrawal
Clarified reasons for termination of the study; the Sponsor reserves the right to terminate the study for duly justified reasons in accordance with the national laws.	Section 5.4 Termination of the Study
Specified that if a repeat Screening/Baseline MRI scan cannot be accomplished within the required 42-day window, then the subject must be reconsented and rescreened.	Synopsis<br>Section 4.4 Schedule of Assessments<br>Section 5.2<br>Section 9.1.2 Brain MRI Acquisition
Specified that if a subject discontinues early, the early termination visit should include an MRI scan if one has not been performed within 90 days prior.	Section 4.4 Schedule of Assessments<br>Section 9.1.2 Brain MRI Acquisition
Sample storage periods prior to destruction for clinical laboratory testing (*i.e.*, hematology, chemistry, urinalysis, Vitamin B_{12}, folate, TSH, TBG, T_3 uptake, haptoglobin, and G6PD) and pharmacokinetic analysis have been added.	Section 8.3.1 Serum Chemistry<br>Section 8.3.2 Hematology<br>Section 8.3.3 Urinalysis<br>Section 8.3.4 Other Laboratory Tests<br>Section 9.2.2 Packaging, Labeling, and Shipping of Blood Samples
Updated clinical laboratory sample shipping conditions and recipients of shipments for T_3 uptake samples and TBG samples; added clarification regarding serum pregnancy testing and procedures for borderline results.	Section 8.3.4 Other Laboratory Tests
Specified that for site radiologist review of MRI results for subject management and preliminary determination of eligibility, as well as for treatment-emergent abnormalities, a re-review by the central reader may be requested on the basis of additional clinical/radiological information known to the site.	Section 9.1.4.1 Evaluation of Brain MRI for Subject Eligibility<br>Section 9.1.5 Site Review
Samples collected for pharmacokinetic analysis are to be destroyed after a storage period of 3 months following completion of the clinical study report, which is expected to be a period of approximately 4 years; removed contact listed for analytical laboratory due to changes in personnel.	Section 9.2.2 Packaging, Labeling, and Shipping of Blood Samples<br>Section 9.2.3 Analytical Laboratory
Details regarding storage and shipment of genotyping samples have been provided.	Section 9.3 Genotyping
In addition to the investigator, all other parties involved in the conduct of the study are also responsible for ensuring that the study is conducted at their sites in accordance with the approved protocol and with the principles of the Declaration of Helsinki, ICH GCP, and with applicable country and local regulatory requirements and laws.	Section 11 Regulatory<br>Section 13 Serious Breaches
Specified timing and criteria of clinical monitoring visits.	Section 17 Quality Assurance and Clinical Monitoring
Subjects may be reimbursed for accommodation by prior agreement with the Sponsor.	Section 21 Administrative and Financial Agreement

Pipettes have been added as equipment for which calibration certification will be confirmed.	Section 22 Study Administration
New references added or removed as applicable to changes in the body of the protocol; information for previously cited references corrected.	Section 23 References

24.9.5. Protocol Version 4.1

The final protocol for Study TRx-237-007 (Version 4.0 dated 21 August 2013) has been revised (Version 4.1 dated 9 September 2013) to remove the requirement that subjects in Germany must be able to provide their own written informed consent in order to be eligible for enrollment in this study as this was inadvertently included in error in Version 4.0.

Other revisions are editorial and are intended to add further clarification or correct typographical errors.

The affected sections for these changes are detailed in the table below.

Summary of Changes	Affected Sections in Revised Protocol (Version 4.1)
The protocol has been modified to no longer require that subjects in Germany must be able to provide their own written informed consent in order to be eligible for enrollment in this study. In the case of subjects in Germany with reduced decision-making capacity, it is acceptable for one or more legally acceptable representatives to provide written informed consent.	Synopsis<br>Section 5.1 Inclusion Criterion No. 7<br>Section 5.3 Discontinuation/Withdrawal<br>Section 14 Informed Consent
Clarified that blood will be collected for purposes of genetic analyses to be performed only for those subjects by or for whom separate legally acceptable informed consent is provided.	Synopsis<br>Section 3.3 Exploratory<br>Section 10.1.3 Exploratory Endpoints
In the schedule of assessments (Table 4-1), corrections have been incorporated for pulse co-oximetry at Visit 2 (post-dose) to denote that this can only be performed by Rater 3, as well as for the Modified ADCS-CGIC at Visit 4 to denote that this can only be performed by Rater 2.	Section 4.4 Schedule of Assessments

24.9.6. Protocol Version 5.0

The final protocol for Study TRx-237-007 (Version 4.1 dated 9 September 2013) has been revised (Version 5.0 dated 20 November 2013) to address comments received from participating Member States in the Voluntary Harmonisation Procedure to restore the exclusionary creatinine clearance to <50 mL/min and to unblind treatment allocation for SUSAR reporting to the pertinent regulatory authorities, as well as to include other clarifications and administrative revisions. The affected sections for these changes are detailed in the table below.

Additional revisions are editorial and are intended to correct typographical errors or eliminate inconsistencies.

Summary of Changes	Affected Sections in Revised Protocol (Version 5.0)
Administrative	
Address and telephone number have been updated for one of the protocol signatories (Statistician).	Section 1.1 Protocol Approval
Only the WCT Global Lead Medical Monitor is identified in the tabular summary of responsible personnel rather than each of the WCT Regional Medical Monitors.	Section 1.2 Responsible Personnel
The locations of study sites are now presented by region rather than country; text referring to "North America" has been revised to "the Americas", and text referring to "Australasia" has been revised to "Asia/Australia" to more accurately reflect the intended countries to be included in this study. Where appropriate, references to "European Union" have been revised to "Europe" so that the region is not restricted to European Union member states.	Section 1.2 Responsible Personnel<br>Synopsis<br>Section 4.2 Population<br>Section 4.5 Randomization and Blinding<br>Section 10.3.4.1 Primary Efficacy Analyses
Exclusion Criteria	
Exclusion criterion has been modified to restore the exclusionary creatinine clearance to <50 mL/min at Screening as in Protocol Version 3.0 (rather than <30 mL/min); if renal concerns arise and calculated creatinine clearance reaches <50 mL/min during the study, study drug should be discontinued.	Synopsis<br>Section 5.2 Exclusion Criterion No. 17<br>Section 6.2.2.5 Other Safety Reasons Requiring Discontinuation
Exclusion criterion regarding current or prior participation in a clinical trial of a product for cognition has been clarified to specify that the last dose must have been received more than 90 days prior (where previously it stated 3 months relative to prior study participation).	Synopsis<br>Section 5.2 Exclusion Criterion No. 24
Safety Assessments / Procedures	
Treatment allocation will be unblinded for SUSAR reporting by the Sponsor to the pertinent regulatory authorities; guidance for SUSAR reporting and unblinding treatment allocation has been revised accordingly.	Section 8.1.6.2 Sponsor Reporting of SUSARs to Regulatory Authorities<br>Section 8.1.6.2.1 Unblinding Treatment Allocation
For G6PD deficiency screening performed outside the site, any suitable laboratory may be used (not restricted to a "local" laboratory).	Section 8.3.4 Other Laboratory Tests

24.9.7. Protocol Version 5.1

The final protocol for Study TRx-237-007 (Version 5.0 dated 20 November 2013) has been revised (Version 5.1 dated 15 May 2014) to update the list of responsible personnel for the study, clarify the exclusion criterion regarding drugs associated with methemoglobinemia, and to include modifications and/or clarifications to safety and other assessments/procedures as summarized in the table below.

Additional revisions are editorial and/or intended to add minor clarification.

Summary of Changes	Affected Sections in Revised Protocol (Version 5.1)
Administrative / Background	
The Sponsor's Global Project Lead and the contact listed for Pharmacovigilance have been updated.	Section 1.2 Responsible Personnel
The background discussion of clinical data has been updated to reflect the cut-off for the most current Development Safety Update Report for LMTM (October 2013).	Section 2.3.3 Safety
Exclusion Criteria	
Exclusion criterion regarding the use of drugs associated with methemoglobinemia has been clarified to indicate that drugs for which there is a warning or precaution in the labeling about methemoglobinemia at approved doses are exclusionary.	Synopsis<br>Section 5.2 Exclusion Criterion No. 23
Safety Assessments / Procedures	
In cases where ARIA is noted, scans are to be repeated every 6 weeks to follow resolution/stabilization of ARIA; it is clarified that stabilization is to be based on at least three follow-up scans.	Synopsis<br>Section 4.4 Schedule of Assessments<br>Section 6.2.2.3 ARIA<br>Section 9.1.2 Brain MRI Acquisition<br>Section 9.1.5 Site Review
Methemoglobin readings are to be repeated in response to values that are > 2.0%. The procedure for obtaining repeat readings has been clarified; specifically, two immediate repeat measurements should be obtained such that a total of three readings are obtained one on each of three different fingers (index, middle, ring) at a single visit. The mean of the three readings will be calculated automatically in the clinical database and used as the basis for safety monitoring decisions. All individual and calculated mean readings will be captured in the clinical database and included in the eCRF.	Section 6.2.2.1Methemoglobinemia and/or Hemolytic Anemia<br>Section 8.4 Pulse Co-oximetry
The description of methemoglobin values that are to be considered AESIs has been clarified to indicate that only those values emergent after screening are to be considered AESIs, and whether or not the values are considered SAEs depends on medical and scientific judgment.	Section 8.1.3 Adverse Events of Special Interest
Other medically significant events that are emergent after screening are to be considered SAEs; with respect to suicidality, serotonin toxicity and ARIA, only post-randomization cases are to be reported as per the procedure for SAEs.	Section 8.1.4 Serious Adverse Events<br>Section 8.9 Serotonin Toxicity (Syndrome)<br>Section 8.10 Columbia-Suicide Severity Rating Scale (C-SSRS)

Summary of Changes	Affected Sections in Revised Protocol (Version 5.1)
Additional guidance is provided regarding assessment and screening of Heinz bodies to indicate that blood samples should be as fresh as possible at the time of slide preparation.	Section 8.3.2 Hematology
Other Assessments / Procedures	
Clarification has been added that the C-SSRS and Serotonin Toxicity Assessment may be rated by Rater 1 (in addition to the safety rater) and that the UPDRS Parts II and III may be rated by any qualified rater (with the exception of raters administering the Modified ADCS-CGIC, which must be independently rated).	Synopsis<br>Section 4.4 Schedule of Assessments<br>Section 7.1 Raters<br>Section 7.2 Instruments
The procedure for blood sample collection for analysis of MT concentrations has been modified. A suitable vacutainer, as defined in the laboratory manual, will be used for blood sample collection (no longer specified in the protocol as a glass sodium heparin vacutainer). All 6 mL of whole blood will now be centrifuged; the separated plasma (2 × 1-mL and 2 × 0.5-mL samples) will be transferred into four Nunc polypropylene tubes. The 2 × 0.5-mL samples will serve as back-up samples for analysis of MT concentrations.	Section 9.2.1 Procedure for Blood Sample Collection
Additional detail is provided regarding the genetic mutations underlying bvFTD to be evaluated as an exploratory objective (*i.e.*, mutations in the coding regions of Tau and TDP-43 genes). The analytical laboratory responsible for the analysis (LabCorp Genomic Services, Seattle, WA, USA) is now identified.	Synopsis<br>Section 3.3 Exploratory<br>Section 9.3 Genotyping<br>Section 10.1.3 Exploratory Endpoints
The description of quality assurance and clinical monitoring has been revised to refer to the clinical monitoring plan for further details regarding the average monitoring frequency.	Section 17 Quality Assurance and Clinical Monitoring

24.9.8. Protocol Version 6.0

The final protocol for Study TRx-237-007 (Version 5.1 dated 15 May 2014) has been revised (Version 6.0 dated 18 June 2014) to no longer require routine MRI monitoring for evaluation of ARIA. This is based on correspondence received from the U.S. Food and Drug Administration (FDA) indicating that development programs for non-antibody compounds are no longer required to institute serial clinical and MRI monitoring for this purpose.

Additional modifications are included with regards to concomitant medications (to allow subjects taking memantine to change from immediate-release memantine to the extended release formulation) and study drug storage temperature conditions (increased to not more than 30°C).

The revisions and affected sections are summarized in the table below.

Additional revisions are editorial and intended to add minor clarification.

Summary of Changes	Affected Sections in Revised Protocol (Version 6.0)
Brain MRI scans will no longer be routinely evaluated for ARIA based on correspondence received from FDA indicating that serial clinical and MRI monitoring is no longer required for this purpose. Post-Baseline brain MRI scans will be performed at Weeks 16, 32, and 52 (or upon early termination) to evaluate change in whole brain volume as an exploratory objective. Guidance is provided regarding follow-up in case evidence of ARIA is noted; if the site submits images to the imaging core laboratory for review, an independent review for ARIA may take place.	Synopsis<br>Section 2.4 Rationale for Study<br>Section 4.1 General Description<br>Section 4.4 Schedule of Assessments<br>Section 6.2.2.3 ARIA<br>Section 9.1 Magnetic Resonance Imaging<br>Section 9.1.2 Brain MRI Acquisition<br>Section 9.1.3 Image Transfer and Quality Assurance<br>Section 9.1.4.2 Evaluation of Brain MRI for ARIA (Section deleted)<br>Section 9.1.4.2 MRI Evaluation for Efficacy<br>Section 9.1.5 Site Review<br>Section 10.3.5.2 ARIA
Based on correspondence from the manufacturer, immediate-release memantine will be discontinued in August 2014; therefore, subjects will be allowed to change to the extended-release formulation.	Section 4.7.1 Dementia Medication
The study drug storage temperature conditions have been modified based on recent satisfactory stability updates; study drug is to be stored at not more than 30°C (previously indicated as to be stored below 25°C).	Section 6.3 Packaging, Labeling, and Storage

24.9.9. Protocol Version 7.0

The final protocol for Study TRx-237-007 (Version 6.0 dated 18 June 2014) has been revised (Version 7.0 dated 29 June 2015) to include modifications and/or clarifications to administrative and background information, study objectives and efficacy/statistical analyses, safety assessments and other procedures. The revisions and affected sections are summarized in the table below.

Additional revisions are included which are editorial and/or intended to add minor clarification.

Summary of Changes	Affected Sections in Revised Protocol (Version 7.0)
Administrative / Background	
The registered address (in Singapore) for the Sponsor has changed and has been updated accordingly.	Title Page
The Sponsor's Global Project Lead has changed and has been updated accordingly. Additional information has also been updated including responsible personnel for Pharmacovigilance (new contact at WCT) and Imaging (addresses and/or telephone numbers have been updated for BioClinica and RadMD, and BioClinica is now identified as responsible for analyses of MRI data).	Section 1.2 Responsible Personnel
The background discussion of clinical data has been updated to reflect the most current Investigator's Brochure and Development Safety Update Report for LMTM.	Section 2 Background<br>Section 2.3.2 Efficacy<br>Section 2.3.3 Safety<br>Section 2.4 Rationale for Study<br>Section 23 References
Objectives and Efficacy / Statistical Analyses	
The primary, secondary, and exploratory endpoints and statistical analyses have been modified. Symptomatic effect as reflected by the FAQ and disease-modifying effect based on reduction in decline in WBV by MRI imaging are now primary endpoints, the Modified ADCS-CGIC is now a secondary endpoint, and disease modification by reduction in the rate of atrophy in frontal and temporal lobes as well as ventricular volume by MRI imaging has been added as an exploratory endpoint. An Imaging MITT population (for analysis of change in WBV) is now defined and will include all randomized subjects who took at least one dose of study drug and have a Baseline and at least one post-Baseline MRI imaging measurement of adequate imaging quality. Sample size calculations are now also discussed for FAQ and MRI.	Synopsis<br>Abbreviations<br>Section 2.4 Rationale for Study<br>Section 3 Objectives<br>Section 4.4 Schedule of Assessments<br>Section 7.2 Instruments<br>Section 9.1 Magnetic Resonance Imaging<br>Section 9.1.2 Brain MRI Acquisition<br>Section 9.1.3 Image Transfer and Quality Assurance<br>Section 9.1.4 Image Evaluation Procedure<br>Section 9.1.4.2 MRI Evaluation for Efficacy<br>Section 10.1 Efficacy Endpoints (and all subsections)<br>Section 10.2.2 FAQ (Section added)<br>Section 10.2.4 MRI (Section added)<br>Section 10.3.1 Analysis Populations<br>Section 10.3.4.1 Primary Efficacy Analyses<br>Section 10.3.4.2 Additional Sensitivity Analyses of Primary Endpoints (and all subsections)<br>Section 10.3.4.3 Analyses of Key Secondary Endpoints<br>Section 10.3.4.5 Exploratory Efficacy Analyses<br>Section 23 References
As the ACE-III and ACE-R are highly correlated, in the few instances where only ACE-III may have been obtained at Baseline, the change in total score (out of 100) from Baseline ACE-III to subsequent ACE-R will be used to compute the change in ACE-R.	Section 4.4 Schedule of Assessments<br>Section 7.2.1 Addenbrooke's Cognitive Examination

Summary of Changes	Affected Sections in Revised Protocol (Version 7.0)
Clarification has been added regarding post-treatment TEAEs, which include the subset of TEAEs that have an onset, increase in severity, or increase in relationship to study drug more than 7 days after the last dose of study drug.	Section 10.3.5.1 Adverse Events
Safety and Other Assessments / Procedures	
For ECG recordings to be obtained in triplicate, clarification has been added to indicate that the 2- to 5-minute interval is approximate.	Synopsis<br>Section 4.4 Schedule of Assessments<br>Section 5.2 Exclusion Criterion No. 18<br>Section 8.6 Electrocardiography<br>Section 24.3 Assessments by Visit
Aural temperature measurement is now specified to be an acceptable (but less preferable) alternative to oral (sublingual) temperature measurement; this alternate means of measuring temperature is to be recorded in the eCRF.	Synopsis<br>Section 4.4 Schedule of Assessments<br>Section 6.2.2.2 Serotonin Syndrome<br>Section 8.5.1 Temperature and Respiratory Rate<br>Section 10.3.5.4 Vital Signs and Weight<br>Section 24.3 Assessments by Visit
Independent neuroradiologists (readers) are now specified to be responsible for MRI eligibility and MRI safety evaluations only. Efficacy MRI data (whole brain and ventricular volumes as well as atrophy in frontal and temporal lobes) will be evaluated centrally and will not require assessment by an independent reader. Clarification has also been added that the results of reader assessments of MRI images (for evaluation of subject eligibility or ARIA) will be communicated to sites within 5 business days of image transfer to the imaging core laboratory (and resolution of any quality issues/queries).	Synopsis<br>Section 4.4 Schedule of Assessments<br>Section 9.1 Magnetic Resonance Imaging<br>Section 9.1.4 Image Evaluation Procedure<br>Section 9.1.4.1 Evaluation of Brain MRI for Subject Eligibility<br>Section 9.1.4.2 MRI Evaluation for Efficacy
The allowable time window for Visit 9 is now ± 14 days (previously ± 7 days), and the follow-up visit is to occur +28 days after the last on-treatment visit (rather than +29 days as previously indicated).	Section 4.1 General Description<br>Section 4.4 Schedule of Assessments<br>Section 24.3 Assessments by Visit
Guidance has been added regarding categorization/labeling of assessments or visits that are performed outside of the stipulated time window and regarding unscheduled visits.	Section 4.4 Schedule of Assessments<br>Section 9.1.2 Brain MRI Acquisition
Guidance regarding contraceptive measures now also indicates women of childbearing potential should be encouraged to return to the clinic in the event of a delayed menstrual period to rule out possible pregnancy.	Section 4.7.7 Contraceptive Measures
The discussion of AE recording has been modified to specify that when describing an AE in terms of a diagnosis, events leading up to a diagnosis should be retained.	Section 8.1 Adverse Events
Clarification has been added for evaluation of sites' technical and personnel capabilities for imaging.	Section 9.1.1 Site Selection and Qualification

24.9.10. Protocol Version 8.0

The final protocol for Study TRx-237-007 (Version 7.0 dated 29 June 2015) has been revised (Version 8.0 dated 7 July 2015) to include updates to responsible personnel as well as modifications to the procedures for quality assurance and clinical monitoring. The revisions and affected sections are summarized in the table below.

Summary of Changes	Affected Sections in Revised Protocol (Version 8.0)
The Sponsor's Head of Safety and Medical Monitoring has changed for this study and has been updated accordingly.	Section 1.2 Responsible Personnel
The Sponsor has appointed Exp-e-Data (UK) Ltd to provide and manage a secure data repository for the upload (as read-only PDFs) and storage of completed rating scales / questionnaires. The uploaded PDFs will be reviewed for the purpose of secondary monitoring to help assure data integrity.	Section 1.2 Responsible Personnel<br>Section 17 Quality Assurance and Clinical Monitoring
The Sponsor has appointed The University of Aberdeen Institute for Complex Systems and Mathematical Biology (ICSMB): (i) to automate reading of PDFs uploaded to the secure server and (ii) to assist with the provision of information to track progress of monitoring and data verification for Sponsor oversight.	Section 1.2 Responsible Personnel<br>Section 17 Quality Assurance and Clinical Monitoring
The Sponsor has appointed the Institute for Clinical Pharmacodynamics (ICPD) to undertake pharmacokinetic analysis.	Section 1.2 Responsible Personnel
